# FAM171B as a Novel Biomarker Mediates Tissue Immune Microenvironment in Pulmonary Arterial Hypertension

**DOI:** 10.1155/2022/1878766

**Published:** 2022-09-22

**Authors:** Lai-Hao Qu, Wen-Juan Luo, Zhi-Guo Yan, Wen-Pan Liu

**Affiliations:** ^1^Cardiothoracic Surgery, The First People's Hospital of Kunming City & Ganmei Affiliated Hospital of Kunming Medical University, Kunming, 650000 Yunnan Province, China; ^2^Department of Thoracic Surgery, Kunming Yan'an Hospital Affiliated to Kunming Medical University, Kunming, 650000 Yunnan Province, China; ^3^Department of Cardiology, The First People's Hospital of Kunming City & Ganmei Affiliated Hospital of Kunming Medical University, Kunming, 650000 Yunnan Province, China

## Abstract

The purpose of this study was to uncover potential diagnostic indicators of pulmonary arterial hypertension (PAH), evaluate the function of immune cells in the pathogenesis of the disease, and find innovative treatment targets and medicines with the potential to enhance prognosis. Gene Expression Omnibus was utilized to acquire the PAH datasets. We recognized differentially expressed genes (DEGs) and investigated their functions utilizing R software. Weighted gene coexpression network analysis, least absolute shrinkage and selection operators, and support vector machines were used to identify biomarkers. The extent of immune cell infiltration in the normal and PAH tissues was determined using CIBERSORT. Additionally, the association between diagnostic markers and immune cells was analyzed. In this study, 258DEGs were used to analyze the disease ontology. Most DEGs were linked with atherosclerosis, arteriosclerotic cardiovascular disease, and lung disease, including obstructive lung disease. Gene set enrichment analysis revealed that compared to normal samples, results from PAH patients were mostly associated with ECM-receptor interaction, arrhythmogenic right ventricular cardiomyopathy, the Wnt signaling pathway, and focal adhesion. FAM171B was identified as a biomarker for PAH (area under the curve = 0.873). The mechanism underlying PAH may be mediated by nave CD4 T cells, resting memory CD4 T cells, resting NK cells, monocytes, activated dendritic cells, resting mast cells, and neutrophils, according to an investigation of immune cell infiltration. FAM171B expression was also associated with resting mast cells, monocytes, and CD8 T cells. The results suggest that PAH may be closely related to FAM171B with high diagnostic performance and associated with immune cell infiltration, suggesting that FAM171B may promote the progression of PAH by stimulating immune infiltration and immune response. This study provides valuable insights into the pathogenesis and treatment of PAH.

## 1. Introduction

Abnormally high blood pressure in the pulmonary arteries is the hallmark of pulmonary arterial hypertension (PAH), a disease or physiological condition with multiple known and unidentified factors [1]. It is characterized by thickening of the intima of the pulmonary artery wall, resulting in abnormal hemodynamics and increased pulmonary resistance [2]. In addition, PAH is a life-threatening cardiovascular illness that can lead to impaired heart function and increased mortality [3]. Over the past few decades, the prevalence of PAH has been reported to range from 15 to 60 cases per million people per year. Significant progress has been made in uncovering the pathophysiology of PAH, as well as identifying prognostic biomarkers and alternative treatments [4]. However, the molecular mechanism underlying PAH has not been elucidated. Angiotropic and hyperplastic drugs, such as PDE-5 inhibitors, endothelin receptor antagonists (ERAs), and prostacyclin receptor agonists can increase exercise endurance and heart function in PAH patients [5–7]. However, the efficacy of the treatment of PAH still needs to be improved for a better prognosis for patients [8]. Thus, it is important to identify effective biomarkers for PAH, study its pathogenesis, and develop targeted therapies.

With the rapid development of gene sequencing technology and bioinformatics research methods, it is now possible to investigate the underlying causes of numerous diseases by carefully assessing the potential changes in gene expression between aberrant and paired normal tissues [4]. However, only a few studies have used machine learning approaches to uncover biomarkers for PAH [9, 10]. These techniques include least absolute shrinkage and selection operator (LASSO) logistic regression, support vector machine-recursive feature elimination (SVM-RFE), random forest (RF), and weighted gene coexpression network analysis (WGCNA). As a regression analysis technique, LASSO analysis sets the coefficients of less significant variables to zero by applying an L1-penalty (lambda) to screen for significant variables and construct the best classification model [11]. The SVM-RFE analysis is a supervised machine learning technique for classifying data points by maximizing the margin between distinct classes in a high-dimensional space [12]. The RF analysis is a nonparametric approach for carrying out classification under supervision [13]. RF encompasses decision trees derived from subdivided datasets. In this study, a single RF classification model was trained and analyzed to identify descriptors capable of discriminating PAH samples from general samples. In addition, this method, represented as WGCNA, is used to investigate gene expression patterns within samples. Genes with consistent expressing modes were subjected to the clustering process, and the relationship between the module and a specific characteristic or phenotype was determined [14]. Consequently, these four machine learning techniques are widely used to identify diagnostic markers and forecast models with high precision and understandability.

In this study, we aimed to reanalyze the datasets previously published by Mura et al. [15], Stearman et al. [16], and Zhao et al. [17], which included the GSE113439, GSE117261, and GSE53408 datasets, respectively. In addition, two sets of microarray mRNA expression data were combined to find genes that were expressed differently. We used differentially expressed genes (DEGs) for functional enrichment analysis and different machine learning approaches for biomarker identification and investigated the diagnostic value of biomarker expression in PAH patients. Finally, we determined the proportion of immune cell infiltration in PAH using the CIBERSORT tool. In the future, we intend to use PAH patient data in the GEO database to conduct bioinformatics research for the determination of biomarkers and specific immune cells associated with PAH, with an ultimate goal to develop drugs that target these biomarkers and immune cells to delay or reverse PAH and improve patient outcomes.

## 2. Materials and Methods

### 2.1. Data Selection

The GSE113439, GSE117261, and GSE53408 microarray datasets were retrieved from the Gene Expression Omnibus (GEO) database (http://www.ncbi.nlm.nih.gov/geo/), containing data for lung tissues collected from 11 normal subjects and 15 PAH patients; 25 normal subjects and 58 PAH patients; and 11 normal subjects and 12 PAH patients, respectively. The GSE113439, GSE117261, and GSE53408 datasets were derived from the GPL6244 platform. We validated our results using GSE53408 expression profiling.  Table 1 provides a complete summary of these datasets.

### 2.2. Data Preprocessing and DEGs Screening

The probes were converted into gene symbols by making use of the probe annotation files that were given by the researchers. Based on the annotated file of each dataset, unmapped probes were eliminated. Multiple probes correspond to the same gene, and the average of this gene in all samples was used for subsequent analyses.

Batch effects were removed from the GSE113439 and GSE117261 datasets using the “sva” function in R [18]. As these datasets contain similar platforms, data can be merged. Principal component analysis (PCA) plots were used on the training matrices to highlight the influence of between-sample rectification. These plots were created before and after the “PCA” function was used to eliminate the interbatch effect [19]. The “limma” function [20] was used to filter DEGs and the “ggplot2” function [21] to show differential gene expression. DEGs were considered statistically significant when adjusted *P* < 0.05 and |log2FC| was >0.5.

### 2.3. Functional Enrichment Analysis

Using the “clusterProfiler” function in R, we investigated DEG enrichment in Disease Ontology (DO), Kyoto Encyclopedia of Genes and Genomes (KEGG), and Gene Ontology (GO) terms (22). In a previous study, researchers used Metascape (http://metascape.org) to conduct pathway enrichment analyses and annotate biological processes to explain the information included in each gene [22]. This study analyzed the DEGs from the training dataset using Metascape's GO and pathway enrichment methods to determine the most important functional biological keywords and signaling pathways. Statistical significance was determined based on the number of enriched genes being ≥3 and *P* < 0.01. Additionally, all the important phrases were categorized according to their membership similarity, and the most enriched term from each cluster was chosen as the representative term. By using the ClusterProfiler function and the “c5.go.v7.4.symbols.gmt” and “c2.cp.kegg.v7.0.symbols.gmt.” datasets, a gene set enrichment analysis (GSEA) of the genomic array was carried out.

### 2.4. Feature Selection Using the Random Forest Model

The DEGs obtained were analyzed using the randomForest function in R [23]. First, the average rate of model miscalculation across all genes was determined. The optimal number of variables for the binary tree in the node was 3, and the best random forest tree count was 500. Random forest models were developed, and the dimensional importance value was computed using the decreasing accuracy method (Gini coefficient method). Disease-specific genes were determined as those with a significance value greater than 2 and ranked among the top three.

### 2.5. Feature Selection Using the LASSO Regression Model

LASSO is a method used to carry out gene selection and classification through regression analysis. The glmnet function in the R package [24] was used to establish a logistic LASSO regression model using 258 DEGs to identify significant prospective gene combinations consistently related to PAH. Ten-fold cross-validation was employed in this study to define tuning parameters, and the partial likelihood of deviance fulfilled the minimal criterion.

### 2.6. Feature Selection Using the SVM Classifier Model

The feature selection approach is an effective method for extracting useful data from available gene datasets [25]. SVM is a supervised learning model used to accurately categorize data points by optimizing the distance between two hyperplanes [26]. SVM-RFE is a well-known feature selection approach that has shown significant and increasing applicability in a high-dimensional data analysis. Feature selection methods are superior to many other feature selection algorithms in terms of data overfitting and classification accuracy and are useful in a variety of fields, including microarray gene expression [27, 28].

### 2.7. Key Module Identification Using WGCNA

The system biology approach, WGCNA, was used to generate gene coexpression networks to investigate gene-gene relationships [29]. First, genes with a variance of over 25% across samples in the integrated dataset were entered into the WGCNA platform. Second, outlier samples were eliminated to confirm the reliability of the network construction outcomes. Third, adjacency was determined using the pick-Soft-Threshold function obtained using the soft thresholding power, which was generated through coexpression similarity. After transforming the adjacency matrix into a topological overlap matrix (TOM), the associated dissimilarity (1-TOM) was measured. Fourth, modules were identified using a combination of hierarchical clustering and a dynamic tree-cut algorithm. We employed average connection hierarchical clustering by minimum genome size (50) to identify genes with similar expression patterns in gene modules [30]. Fifth, for modules related to clinical features, module membership (MM) and gene significance (GS) were calculated. Finally, the correlation between MM and GS of important modules is shown. Furthermore, genes in the modules were evaluated using the information included in the modules. We identified the most important key module associated with PAH by assessing the *P* value and Pearson's correlation coefficient of module eigengenes (MEs) and the disease feature associated with each module. MM denoted the association between MEs and gene expression profiles. Then, the GS of the modules, which represents the association between genetic markers and disease characteristics, was determined. Genes with high MM and GS values in the critical module were significantly associated with disease characteristics. We set MM>0.55 and GS>0.55 as the filter criteria for selecting important genes in the critical module after its selection.

### 2.8. Screening and Verification of Biomarkers

Next, intersecting genes identified using the four different methods were chosen for subsequent analyses. The GSE53408 was used as validation sets for the comprehensive assessment of the efficacy of critical diagnostic markers. The datasets mentioned above were employed to validate differences in diagnostic markers expression between samples collected from normal subjects and PAH patients. Diagnostic effectiveness was then assessed by calculating the receiver operating characteristic (ROC) according to the area under the curve (AUC), which provided insight into the algorithm's predictive potential. A value of *P* < 0.05 indicated two-sided statistical significance.

### 2.9. Immune Cell Infiltration Analysis

Using CIBERSORT with the merged matrix, we evaluated immune cell infiltration. Afterwards, PCA was performed on the results using the ggplot2 function in R and a 2D PCA map was produced. The “corrplot” function was used to plot correlated data. Correlations between 22 distinct infiltrating immune cell types were determined using the “corrplot” function [31]. We constructed violin plots using the “ggplot2” function to illustrate variations in immune cell infiltration.

### 2.10. Interaction between Immune Cells and Biomarkers

The Spearman's rank correlation test, performed with the help of the R program, was used to investigate the potential significance of a link between infiltrating immune cells and newly discovered biomarkers. Correlations were shown through a chart approach using the “ggplot2” function.

### 2.11. Statistical Analysis

The moderate *t-*test was performed to filter DEGs, while Fisher's exact test was used to evaluate GO and KEGG annotation enrichments. Wilcoxon's test was conducted to determine immune cell counts. The statistical analysis was done in the R program (version 4.1.1).

## 3. Results

### 3.1. Analysis Process

**Figure 1:**
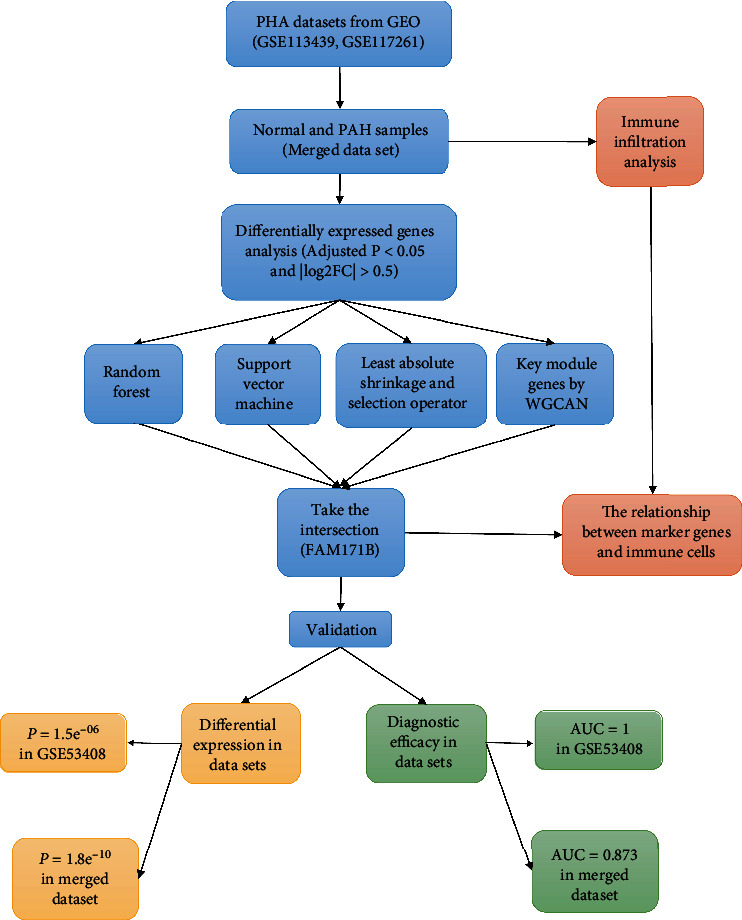
Workflow for the study.

The workflow of this study is shown in Figure 1.

### 3.2. Data Processing and DEG Selection

**Figure 2:**
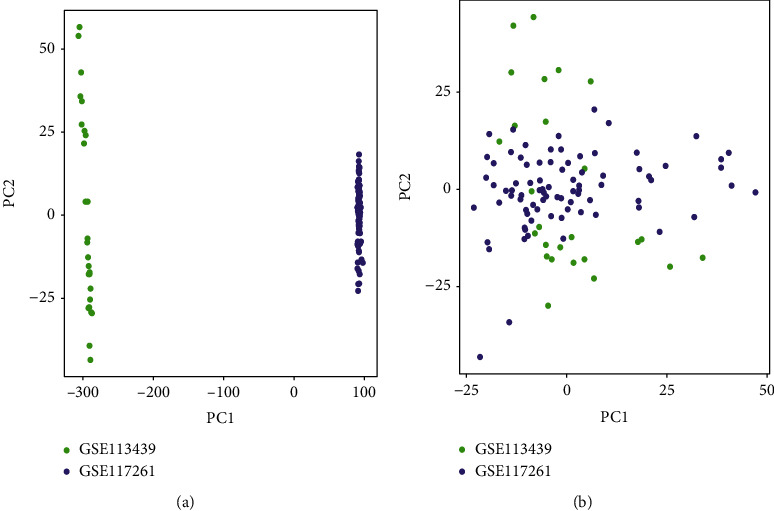
Principal component analysis (PCA) analysis of gene expression datasets. The scatter plots' points depict samples based on the top two principal components (PC1 and PC2) of gene expression profiles without and with batch effect removal. (a) PCA cluster plot of GSE113439 and GSE117261 before sample correction and batch effect removal. (b) PCA cluster plot of GSE113439 and GSE117261 after sample correction and batch effect removal. The colors denote samples from two distinct datasets, respectively. Each dot represents a sample; green represents a sample from GSE113439; purple represents a sample from GSE117261.

**Figure 3:**
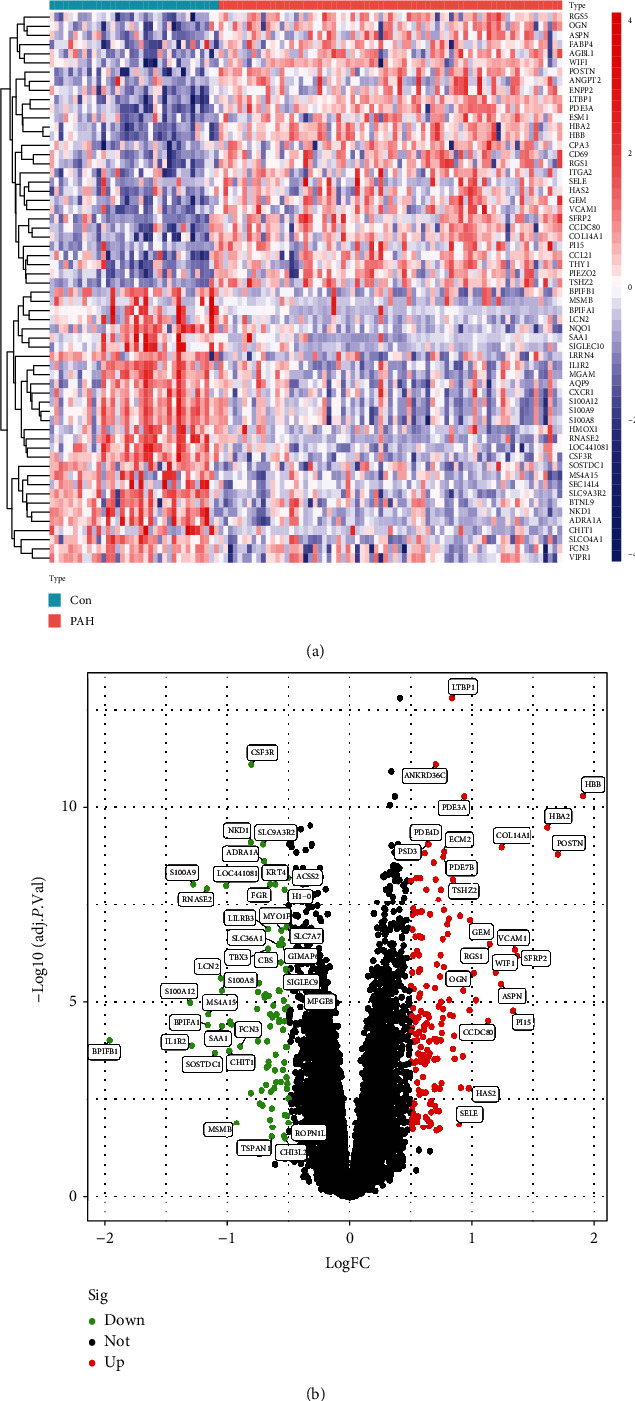
Visualizing the results of differential genes. (a) Clustering heat map of the genes exhibiting significantly differential expression PAH versus normal samples. Statistically significant DEGs were defined as |log2Foldchange| > 0.5 and adjusted *P* value<0.05. Cyan represents PAH samples; reddish-orange represents normal samples. (b) Volcano map of DEGs; red represents upregulated differential genes, black represents no significant difference in genes, and the green represents downregulated differential genes. DEGs: differentially expressed genes; PAH: pulmonary arterial hypertension.

Expression matrices for the GSE113439 and GSE117261 datasets were merged, which included 27 normal samples and 22 PAH samples. Next, normalization and batch effect removal were performed, and a 2D PCA plot was used to represent the dataset before and after batch effect removal (Figures 2(a) and 2(b)). After data preparation, using the R software, we identified 258 DEGs in the normalized data, as illustrated by the heat and volcano maps shown in Figures 3(a) and 3(b). DEGs obtained by differential analysis of PAH and normal samples, which included 169 upregulated and 89 downregulated genes are shown in Supplementary Table 1.  Table 2 displays the top 20 most upregulated and downregulated genes.

### 3.3. Functional Correlation Analysis

**Figure 4:**
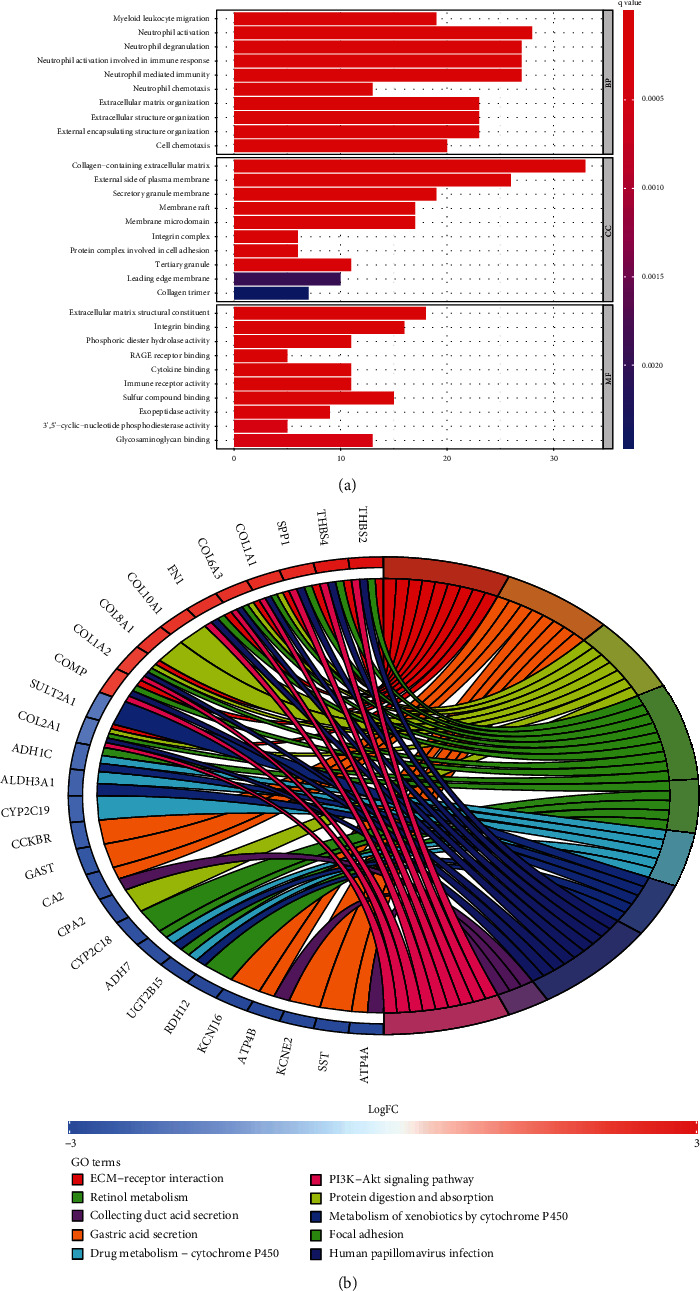
Functional enrichment analyses of DEGs. (a) Gene Ontology (GO) enrichment analyses of DEGs. The *x*-axis shows the number of genes enriched on the terms, and the *y*-axis shows the pathway terms. The *q*-value of each term is colored according to the legend. BP: biological process; CC: cellular component; MF: molecular function; (b) Kyoto Encyclopedia of Genes and Genomes (KEGG) enrichment analyses of DEGs. The *q* value of each term is colored according to the legend. The different colors represent different pathway terms.

**Figure 5:**
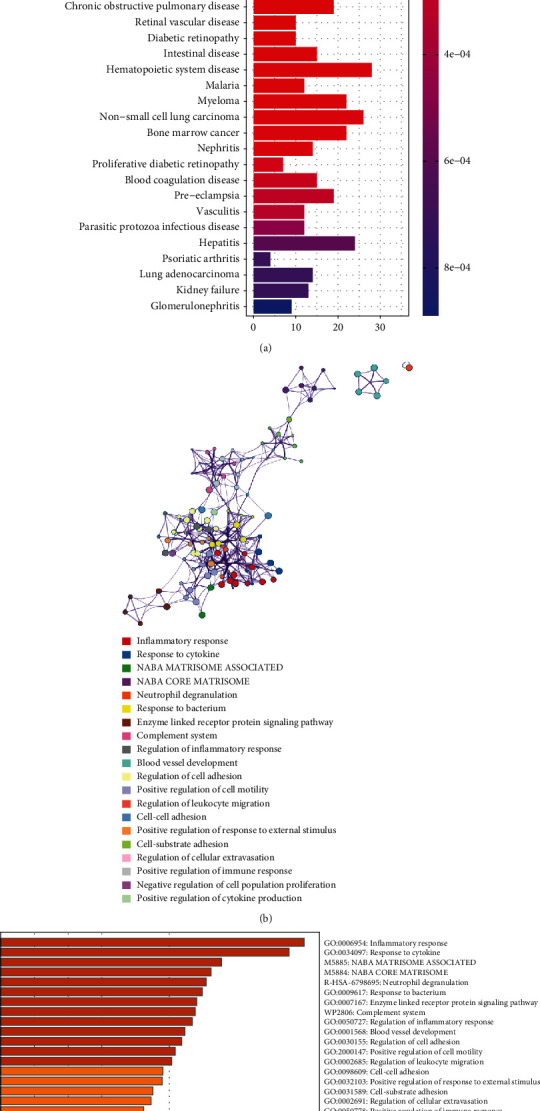
Disease Ontology (DO) and Metascape analyses of DEGs. (a) DO enrichment analysis was performed on DEGs. The *x*-axis shows the number of genes enriched on the terms, and the *y*-axis shows the pathway terms. The *q* value of each term is colored according to the legend. DEGs are differentially expressed genes. (b) The network of the top 20 enriched term clusters. The color indicates cluster identification, the thickness of the edge indicates the similarity score, and terms with a similarity score> 0.3 are connected by an edge. (c) The top 20 clusters are shown in a heat map. Color is used to indicate cluster identification: the lower the *P* value, the darker the color.

**Figure 6:**
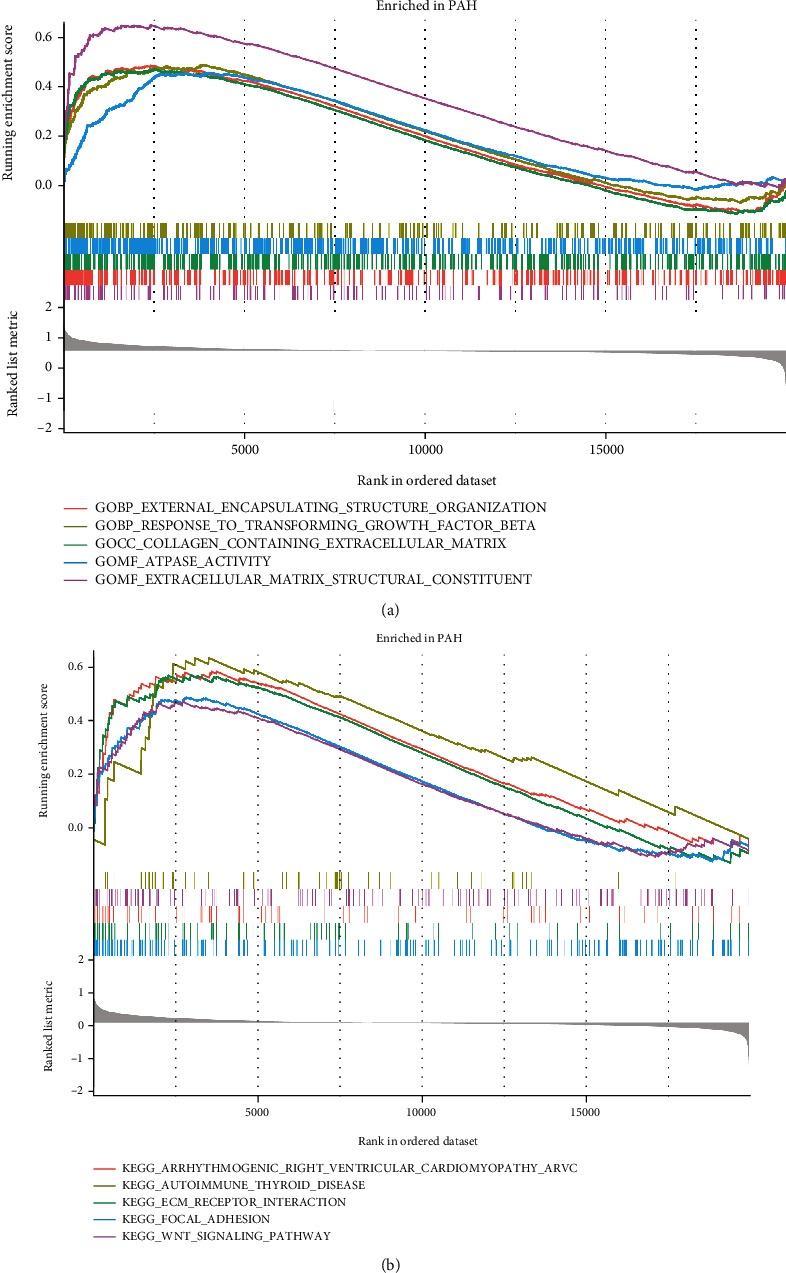
LogFC values were calculated for all genes, and gene set enrichment analysis (GSEA) analysis was performed based on logFC using “c2.cp.kegg.v7.4.symbols.gmt” and “c5.go.v7.4.symbols.gmt” in the PAH samples. (a) Analysis of the GO pathway terms for all genes enriched in the PAH samples using GSEA. (b) Analysis of the KEGG pathway terms for all genes enriched in the PAH samples using GSEA. PAH: pulmonary arterial hypertension.

The results of the GO enrichment analysis of DEGs are mainly presented in the following aspects: biological process (BP): ribosome biogenesis, mitotic nuclear division regulation, and mitotic cytokinesis; cellular component (CC): preribosome and centriole; and molecular function (MF): DNA-dependent ATPase and DNA helicase activity (Figure 4(a); Supplementary Table 2). DEGs were abundant in eukaryotes, melanoma, hypertrophic cardiomyopathy, and dilated cardiomyopathy, according to the KEGG analysis (Figure 4(b); Supplementary Table 3).  Figure 5(a) illustrates the findings of the DO analysis (Supplementary Table 4). DEGs were most related to osteoarthritis, lung disorders including chronic obstructive pulmonary disease and obstructive lung disease, and cardiovascular diseases including arteriosclerosis, atherosclerosis, myocardial infarction, and coronary artery disease. To further comprehend the functional and metabolic pathways connected with these DEGs, an enrichment analysis was conducted utilizing Metascape to uncover the top 20 clusters with the highest significant enrichment (Figures 5(b) and 5(c); Supplementary Table 5). The results of Metascape enrichment are mainly manifested in the inflammatory response, response to cytokines, and response to bacteria. GSEA results suggested that in PAH samples, immune response inactivation and adaptive immune responses dominated GO biological processes (Figure 6(a); Supplementary Table 6). And the enrichment pathway in KEGG mainly includes the chemokine signaling pathway, cytokine–cytokine receptor interaction pathways, and hematopoietic cell pathways (Figure 6(b); Supplemental Table 6). These findings suggest that the immune response significantly influences the development of PAH.

### 3.4. Random Forest-Identified Key Genes

**Figure 7:**
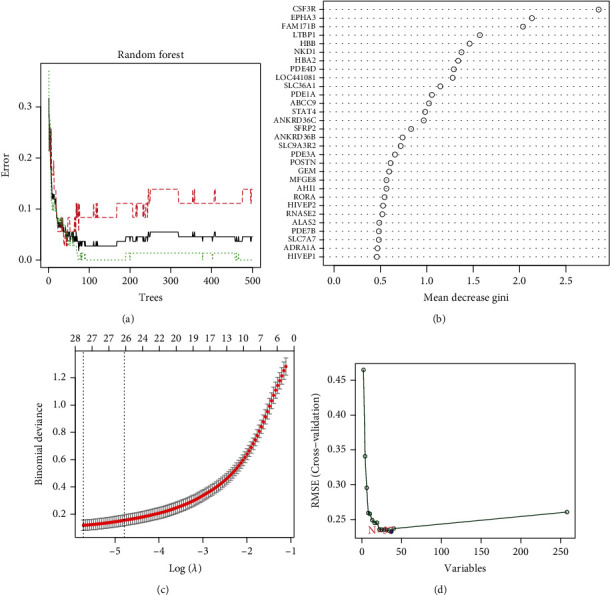
Construct multiple machine learning models based expression of DEGs. (a) The effect of the decision tree number on the error rate. The *x*-axis denotes the number of decision trees, while the *y*-axis shows the error rate. When approximately 100 decision trees are used, the error rate is generally steady. (b) The results of Gini coefficient method in a random forest classifier. The *x*-axis displays the genetic variable, and the *y*-axis the significance index. (c) Fine-tuning the least absolute shrinkage and selection operator (LASSO) model's feature selection. LASSO regression was used to narrow down the DEGs, resulting in the discovery of 28 variables as potential markers for PAH. The ordinate represents the value of the coefficient, the lower abscissa represents log(*λ*), and the upper abscissa represents the current number of nonzero coefficients in the model. (d) A plot illustrating the process of selecting biomarkers using the support vector machine-recursive feature elimination (SVM-RFE) technique. The SVM-RFE technique was used to identify a subset of 37 characteristics from the DEGs. DEGs: differentially expressed genes; PAH: pulmonary arterial hypertension.

A random forest filter was then used to narrow down the 258 DEGs. After determining the optimal parameter, mtry (the optimal number of variables in the binary tree in a node), we performed recurrent random forest classification on all possible values of 1–258 variables and evaluated the average error rate of the model. The average error rate when all variables were chosen is shown in Figure 7(a). Then, we chose 3 as the variable number parameter. The number of variables, as well as the out-of-band error, was kept to a minimum. Finally, we determined the relationship between the model error and the number of decision trees using 500 trees as the model's parameters (Figures 7(a) and 7(b)), which showed a steady error in the model. After that, we calculated the variable significance of the output results (Gini coefficient approach) throughout the random forest model building process in terms of decreasing accuracy and decreasing mean square error. Next, we selected three genes with importance greater than 2 (*CSF3R*, *EPHA3*, and *FAM171B*) as prospective genes for subsequent investigations.

### 3.5. Selection of Significant Genes by Using the LASSO Regression Model

To construct a LASSO regression model, 258 DEGs between the two groups were chosen. Next, the best suitable log (*λ*) (=28) values were determined through 10-fold cross-validation (Figure 7(c)). Finally, 28 genes with nonzero coefficients were identified (*LTBP1*, *CSF3R*, *ANKRD36C*, *HBB*, *HBA2*, *NKD1*, *PDE4D*, *HIVEP1*, *POSTN*, *ADRA1A*, *FAM171B*, *BICC1*, *H1-0*, *RGS5*, *AHCYL2*, *FZD7*, *RGS1*, *WIF1*, *LRRN4*, *PI15*, *CD14*, *ACE2*, *C5*, *BPIFB1*, *SOSTDC1*, *IL13RA2*, *FAM107A*, and *TFPI2*) and used for subsequent analyses.

### 3.6. Selection of Significant Genes by Using the SVM-RFE Model

A total of 37 genes (*LTBP1*, *FAM171B*, *TSHZ2*, *CSF3R*, *NT5E*, *EPHA3*, *HBB*, *HBA2*, *STAT4*, *ANKRD36C*, *PDE7B*, *ADRA1A*, *PDE3A*, *ECM2*, *AHCYL2*, *NKD1*, *SLC9A3R2*, *WIF1*, *HIVEP2*, *PSD3*, *ALAS2*, *LOC441081*, *KRT4*, *H1-0*, *FGR*, *ABCC9*, *AHI1*, *GEM*, *SFRP2*, *C5*, *RORA*, *BICC1*, *IL13RA2*, *PDE4D*, *FZD7*, *POSTN*, and *COL14A1*) with the lowest root mean square error were fitted into the SVM classifier by the SVM-RFE method (Figure 7(d)).

### 3.7. Gene Coexpression Network and Module Identification

**Figure 8:**
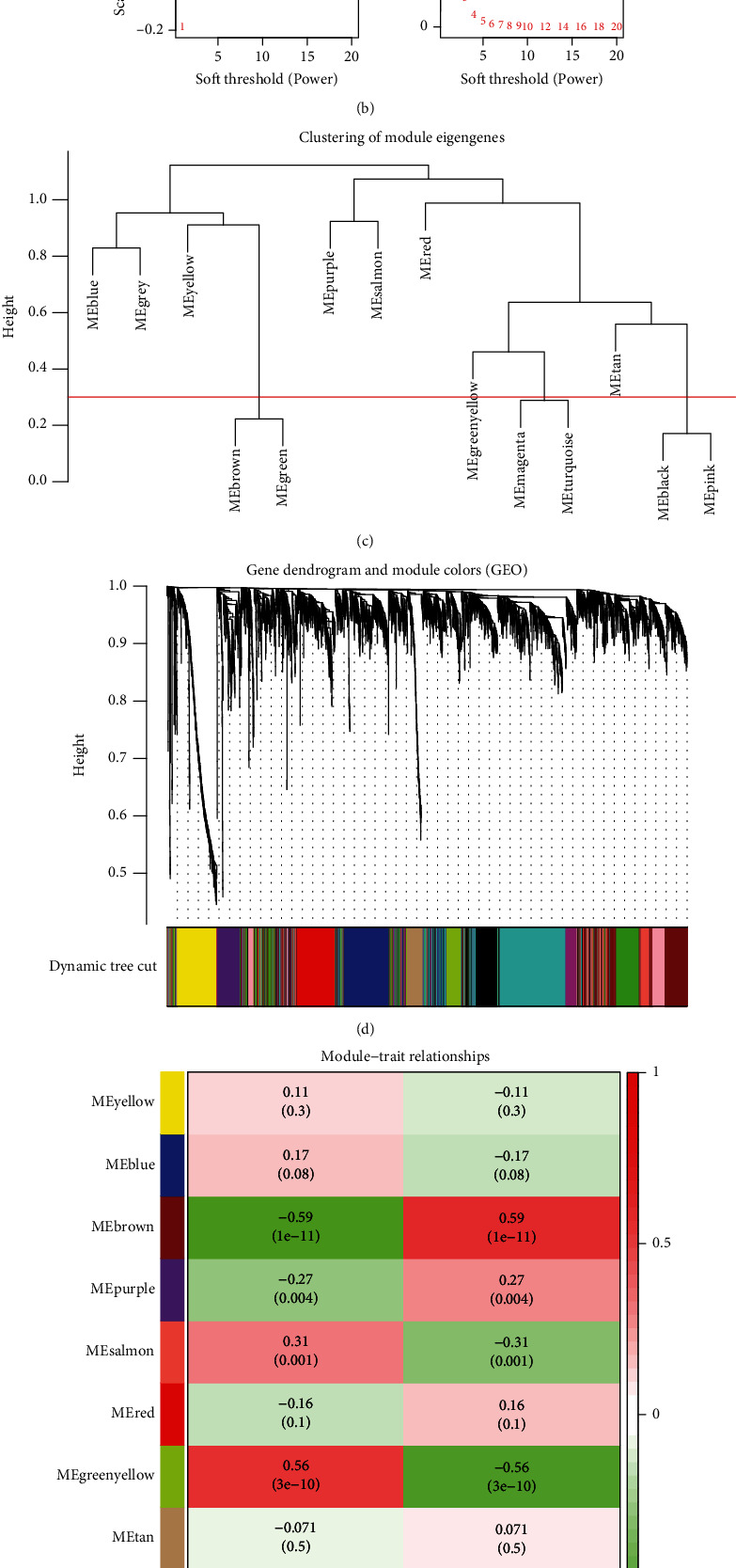
Analysis of the weighted coexpression network in merged dataset. (a) Sample clustering of merged data set. The samples were classified into two clusters that were significantly distinct. Cluster 1 was chosen for further analysis. (b) Selection of optimal thresholds. The threshold is 5. (c) The threshold was set to 0.3 and minimum number of genes per module to 50 to merge modules that are similar in the cluster tree. (d) Different modules are produced and shown in different colors by aggregating genes with strong correlations into a same module. (e) Analysis of correlations between modules and PAH. The brown module was significantly correlated with PAH (*r* = 0.59; *P* = 1e − 11) and with normal samples (*r* = −0.59; *P* = 1e − 11). (f) Correlation plot between MM (*x*-axis) and GS (*y*-axis) of genes contained in the brown module. PAH: pulmonary arterial hypertension.

First, genes were ordered from the largest to smallest in terms of variance, and the top 25% (4992) of these genes were selected for subsequent investigations. Second, the flashClust function in R was used to carry out a cluster analysis, with a threshold of 65 and one outlier sample identified and eliminated (Figure 8(a)). Cluster 1 contained 108 samples, which we intended to maintain. Third, the “pickSoftThreshold” mechanism of the WGCNA software package was used to filter values from the power parameter range of 1-20. In this research, we created a scale-free network with a soft threshold of a power of 5 (scale − free R2 = 0.85) (Figure 8(b)). The threshold was set at 0.3, and the minimum gene number per module was set at 50, enabling the merging of similar modules in the cluster tree (Figure 8(c)). As shown in Figure 8(d), we found 11 modules containing genes with similar coexpression characteristics. The colors used to differentiate the modules were chosen at random. As compared to the other modules, the module eigengene (ME) of the brown model showed the most significant positive correlation and relationship with PAH (*r* = 0.59; *P* = 1e − 11) (Figure 8(e); Supplementary Table 7). Thus, the brown module comprised 921 genes. In addition, we evaluated the correlation between gene MMs and GSs in the brown module. As expected, significant positive correlations were discovered between the MMs and GSs of brown module genes (cor = 0.48, *P* = 3e − 54) (Figure 8(f) and Supplementary Table 8). In the brown module, 27 essential genes (*ABCC9*, *AHI1*, *ANKRD36*, *ANKRD36B*, *ANKRD36C*, *ARHGAP21*, *CACNA2D1*, *ECM2*, *FAM171B*, *FRMD4B*, *GLT8D2*, *JMY*, *KLF12*, *LUC7L3*, *MACF1*, *N4BP2*, *NBEAL1*, *NT5E*, *PHIP*, *PNISR*, *RORA*, *RPS6KA5*, *RUFY3*, *SHPRH*, *WIF1*, ZNF483, and *ZNF711*) were identified for subsequent analyses.

### 3.8. Biomarker Screening and Verification

**Figure 9:**
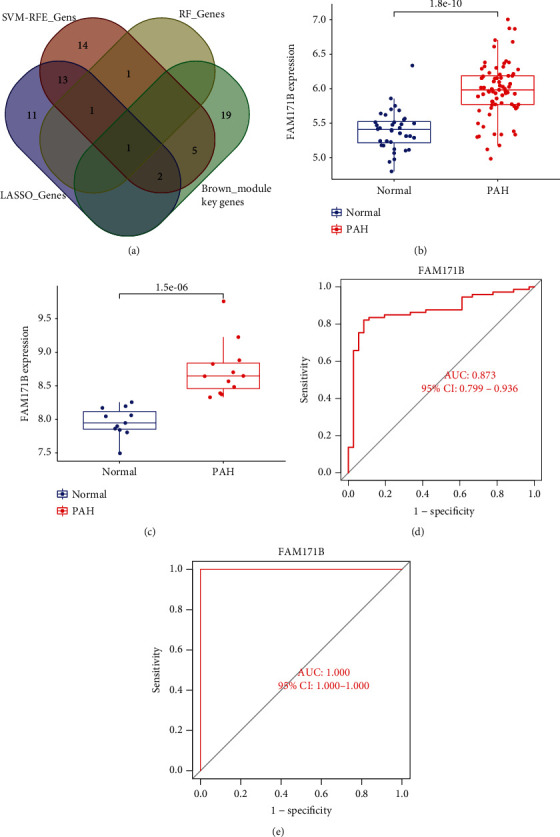
Screening and verification of diagnostic markers. (a) Venn diagram showing overlapping marker with RF, Lasso, SVM-RFE, and WGCNA. FAM171B mRNA expression is significantly higher in PAH samples than in normal samples, (b) the combined dataset (*P* = 1.8e − 10) and (c) the GSE53408 (*P* = 1.5e − 06). ROC curves were constructed using publicly available data to assess the diagnostic accuracy of FAM171B for PAH. (d) The combined dataset had an AUC of 0.873. (e) GSE53408 had an AUC of 1. ROC: receiver operating characteristic; AUC: area under the ROC curve; PAH: pulmonary arterial hypertension.

A diagnosis-related gene was generated by merging the genes identified using the four approaches (Figure 9(a)). The difference in FAM171B expression between normal and PAH samples in the combined dataset and GSE53408 dataset were *P* = 1.8e − 10 (Figure 9(b)) and *P* = 1.5e − 06 (Figure 9(c)), respectively. We generated ROC curves for the combined dataset, and the GSE53408 dataset found that their ROC AUCs were 0.873 (Figure 9(d)) and 1 (Figure 9(e)), respectively. Although the small sample quantity may have influenced the ROC values, these results demonstrate that FAM171B helps to distinguish PAH samples from normal samples.

### 3.9. Immune Cell Infiltration Analysis Findings

The combined data matrices of the GSE113439 and GSE117261 datasets were analyzed using CIBERSORT, and the findings of this analysis are shown in Supplementary Table 9.

**Figure 10:**
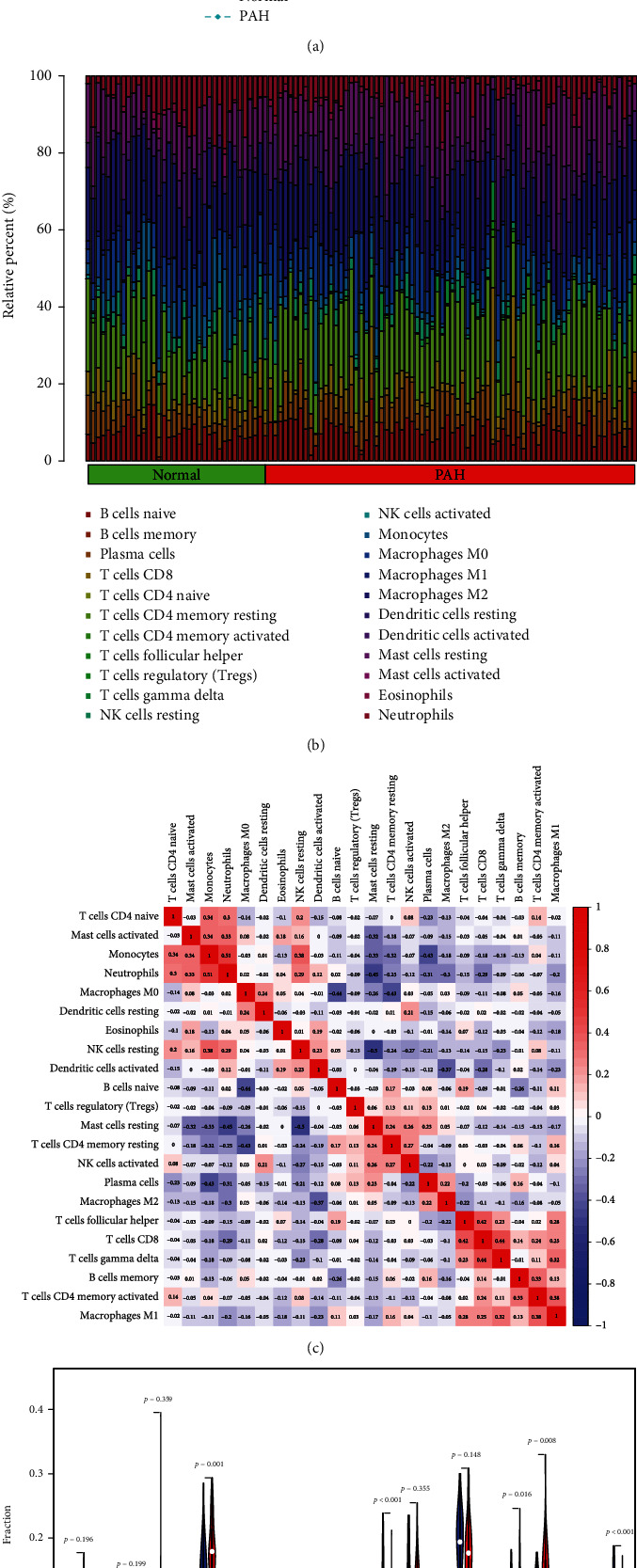
Comparing the composition of immune cell infiltration in the normal and PAH samples by using the combined data matrix of GSE113439 and GSE117261 and visualized the results. (a) PCA cluster plot of immune cell infiltration between normal and PAH samples. (b) The heat map of the 22 subpopulations of immune cells. (c) Correlation heat map of 22 types of immune cells. The size of the colored squares represents the strength of the correlation: red represents a positive correlation; blue represents a negative correlation. The redder the color, the stronger the correlation. (d) Violin diagram of the proportion of 22 types of immune cells. (The normal samples are marked as blue color and PAH samples marked as red color. *P* values <0.05 were considered as statistically significant.) PAH: pulmonary arterial hypertension; PCA: principal component analysis.

PCA analysis was used to determine the difference between PAH and healthy samples. The PCA cluster analysis revealed a statistical difference between the two groups' immune cell infiltration (Figure 10(a)). Using the data matrix derived from the combined GSE113439 and GSE117261 datasets, we assessed the infiltrating immune cell composition in PAH and healthy samples (Figure 10(b)). According to our results, the percentage of CD4 naïve T cells (*P* < 0.05), resting NK cells (*P* < 0.05), monocytes (*P* < 0.05), and neutrophils (*P* < 0.05) was substantially higher in healthy samples than in PAH samples. In PAH tissues, however, the fraction of resting CD4 memory T cells (*P* < 0.05), activated dendritic cells (*P* < 0.05), and resting mast cells (*P* < 0.05) was considerably greater than in healthy samples (Figure 10(c)). In addition, the interaction across 22 immune cells was studied (Figure 10(d)). Naïve CD4 T cells showed significant association with monocytes (*r* = 0.34), neutrophils (*r* = 0.3), and resting NK cells (*r* = 0.2) and a significantly inverse relationship with activated dendritic cells (*r* = −0.15) and resting mast cells (*r* = −0.07). Monocytes showed significant association with neutrophils (*r* = 0.51) and resting NK cells (*r* = 0.38) and a significantly inverse relationship with resting mast cells (*r* = −0.33), activated dendritic cells (*r* = −0.03), and resting memory CD4 T cells (*r* = −0.32). Neutrophils showed significant association with resting NK cells (*r* = 0.29) and activated dendritic cells (*r* = 0.12) and a significantly inverse relationship with resting mast cells (*r* = −0.45) and resting memory CD4 T cells (*r* = −0.25). Resting NK cells showed significant association with activated dendritic cells (*r* = 0.23) and a significantly inverse relationship with resting mast cells (*r* = −0.5) and resting memory CD4 T cells (*r* = −0.24). Activated dendritic cells showed significant association with resting mast cells (*r* = −0.04) and resting memory CD4 T cells (*r* = −0.19). Resting mast cells are significantly associated with resting memory CD4 T cells (*r* = 0.24).

### 3.10. Correlation between FAM171B and Infiltrating Immune Cells

**Figure 11:**
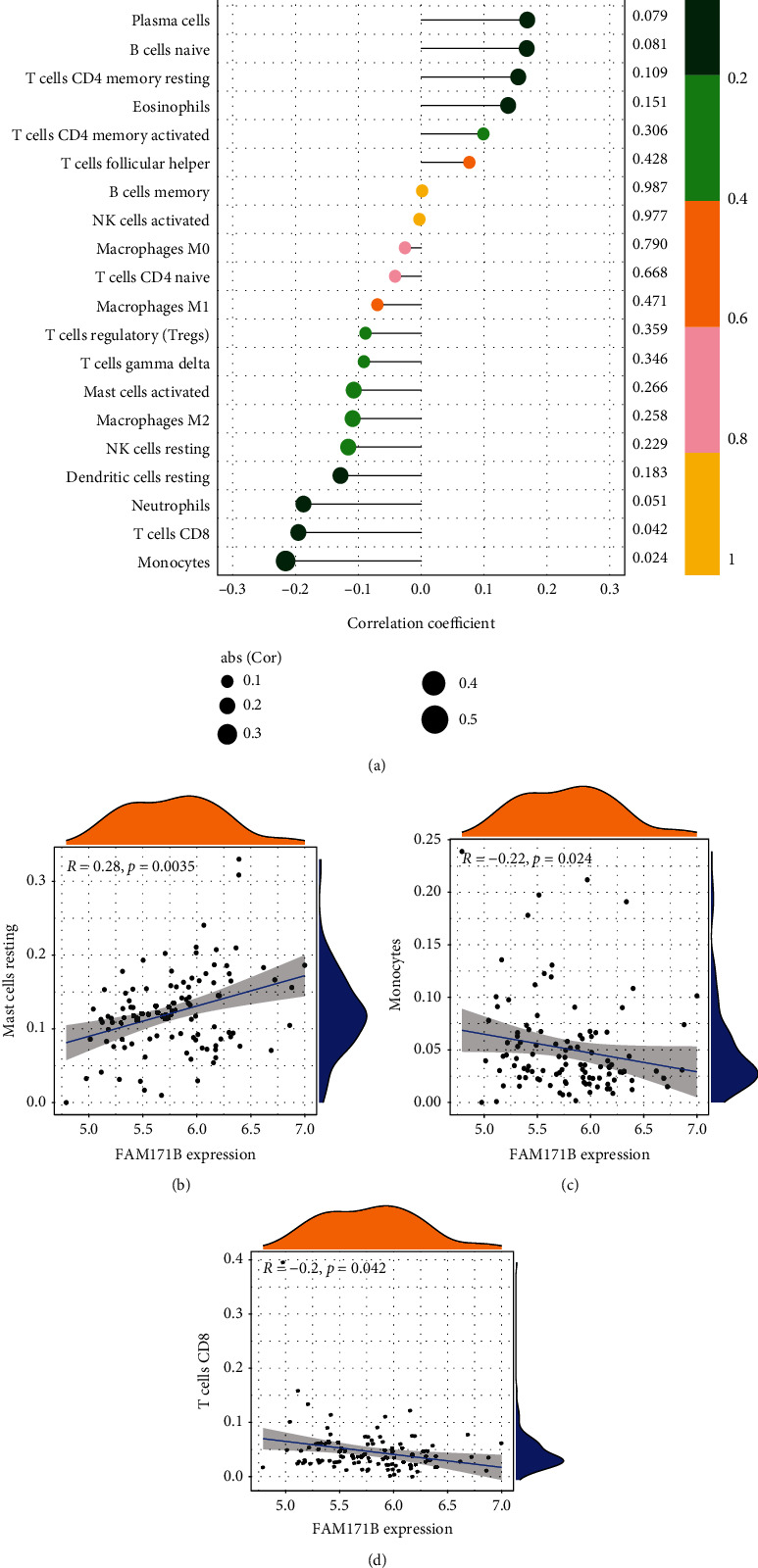
Visualization of the results of immune cell infiltration and FAM171B correlation analysis based on the combined data matrix of GSE113439 and GSE117261. (a) Correlation between FAM171B and infiltrating immune cells. The size of the dots represents the strength of the correlation between genes and immune cells: the larger the dots, the stronger the correlation and vice versa. The color of the dots represents the *P* value: the greener the color, the lower the *P* value, and the yellower the color, the larger the *P* value. (*P* < 0.05 was considered statistically significant.) The correlation analysis in the expression of FAM171B and mast cells resting (b), monocytes(c), and CD8 T cells (d).

We evaluated the relationship between the immune infiltration outcomes and FAM171B. As shown in Figure 11(a), FAM171B was strongly connected with resting mast cells (*r* = 0.28, *P* = 0.0035; Figure 11(b)) and negatively associated with CD8 T cells (*r* = −0.2, *P* = 0.042; Figure 11(c)) and monocytes (*r* = −0.22, *P* = 0.024; Figure 11(d)). Supplementary Table 10 shows the relationship between FAM171B and immune cells.

## 4. Discussion

PAH causes shear stress, endothelial damage in the artery wall, and unfavorable pulmonary vascular reconstruction over time. A distinctive feature of PAH is pulmonary artery remodeling caused by an imbalance of vascular wall proliferation and apoptosis; however, the precise mechanism by which PAH occurs remains unknown [32]. Consequently, it is essential to explore the biological processes underlying the incidence and progression of PAH to allow earlier identification and treatment of the disease, improve the prognosis of the condition, and develop effective strategies for reversing the disease process [33].

By comparing gene expression between PAH and normal samples, we identified 258 significant DEGs, including 169 upregulated and 89 downregulated DEGs. These DEGs were subsequently analyzed by GO and Metascape function-related enrichment analyses. These genes exhibited significant correlations with immune responses and inflammatory signals (e.g., neutrophil activation during the immune response, myeloid leukocyte migration, and neutrophil activation). KEGG analysis revealed that genes involved in the coagulation cascades and complement, NF-*κ*B signaling, chemokine signaling, and ECM–receptor interactions were enriched. The functional enrichment analysis results confirmed further that inflammation and immunity play a role in the occurrence and progression of PAH. Irrespective of the etiology or type of PAH, inflammation usually occurs in the lungs of patients suffering from the disease, with immune cell infiltration [34]. Recruited immune cells produce localized and circulating cytokines, which cause alterations in the pulmonary vascular system; these include interleukin (IL)-1, IL-2, IL-4, IL-8, and IL-12p70, tumor necrosis factor (TNF)-*α*, macrophage inflammatory protein-1*α*, and the chemokines, CXC3L1 (fractalkine), CCL5 (RANTES), and CCL2 [35, 36]. In patients with PAH, a rise in the levels of serum inflammatory markers is a prognostic indicator of disease severity and patient survival [35]. Inflammatory indicators, such as CCL2, CCL5, and fractalkine, have been associated with severe PAH [37]. In the context of PAH, IL-6 is an indicator of right ventricular failure, and investigations on humans and animals have revealed an elevation in IL-6 levels during PAH [38]. In addition, alterations in immunological processes significantly contribute to PAH by inducing inflammatory cell recruitment, pulmonary vasculature remodeling, and autoimmune reactions [39]. In the PAH model, NF-*κ*B signaling is activated, and sevoflurane can modulate NF-*κ*B signaling by inhibiting p-I*κ*B, p-p65, and p65 levels, reducing pulmonary fibrosis, and preventing PAH [40]. TLR/NF-*κ*B pathway inhibition may also benefit PAH patients, reducing inflammatory and immune responses and pulmonary vascular remodeling [41]. Cytokines IL-1*β*, IL-6, and TNF-*α* are involved in PAH-related modifications of the pulmonary artery wall [42]. The TLR family is pattern recognition receptors that recognize microbial fragments and activate the NF-*κ*B pathway. Decreased TLR3 expression is associated with endothelial cell death and changes in the pulmonary artery wall [43]. These data support the notion that inflammation and immune responses play a role in PAH development.

In the last 20 years, several different machine learning strategies and feature extraction algorithms have been widely applied for diagnosing and predicting diseases [44–49]. Most of these studies apply machine learning methods to simulate the progression of malignancy and find significant characteristics that are then used in a categorization scheme. According to the results of our study and those of other researchers [50–56], this was the first study in which analytical methods for identifying PAH biomarkers use many machine learning approaches, including RF, Lasso, SVM-RFE, and WGCNA. Akter et al. [57] suggest that merging different machine learning algorithms may boost prediction performance and construct highly accurate diagnostic models. Thus, using the four machine learning approaches enabled us to identify potentially significant biomarkers critical for the evaluation of PAH. Finally, in this study, FAM171B was selected and shown to be accurate for in-depth verification, confirming our prediction and proving its feasibility through the integration approach.

FAM171B is a protein yet to be identified, and its function is unknown. A group of researchers reported on a mutant mouse with gastroschisis that had a mutation in *Slit3*, as well as an extra point mutation in Fam171A1, a related family member that has 35% amino acid identity with FAM171B [58]. In addition to gastroschisis, this mutant mouse was found to have a double-outlet right ventricle with an atrioventricular septal defect, atrioventricular septal defect, and ventricular noncompaction [59]. Furthermore, FAM171B is a member of the Fam171b protein family, a family of secreted proteins with high and selective expression levels in the brain; however, its function has not yet been determined. Owing to these traits, *Fam171b* is one of 106 genes known as the “core brain ignorome” [60]. Only a few studies have demonstrated that this gene is involved in developing congenital heart disease; however, its precise function in the illness's progression remains unknown. Since the cardiopulmonary vascular system is closely related to PAH, it is likely to become a potential therapeutic target for reversing or delaying PAH progression.

We utilized CIBERSORT to evaluate immune cellular components in PAH and normal samples and discovered that PAH-associated biological processes are strongly connected to several immune cell types. This investigation showed that resting memory CD4 T cells, activated dendritic cells, and resting mast cells are considerably expressed in PAH samples. However, resting NK cells, monocytes, and neutrophils are significantly expressed in normal samples. In addition, it was discovered that *FAM171B* is substantially expressed in PAH tissues. The correlation analysis revealed that resting mast cells were significantly associated with *FAM171B*, whereas CD8 T cells and monocytes were negatively associated with *FAM171B*, indicating that high *FAM171B* expression was closely associated with the extent of infiltration of resting mast cells and CD8 T cells. These results prove that the high resting mast cell counts reported in PAH tissues and the high monocyte counts observed in normal tissues may be connected to FAM171B. Therefore, the results of this analysis indicate that FAM171B and many inflammatory cell types are involved in the process of PAH; this supports the need for further research into PAH molecular pathways.

IL-5, IL-4, and IL-13, as well as antibodies (particularly IgE), are produced by CD4^+^ T_H_2 cells [61]. Several studies using animal models have investigated T_H_2 immune responses as causative factors for PAH. For instance, T_H_2 responses, which include antigen sensitization and subsequent antigen challenge, may result in smaller pulmonary artery muscularization due to interactions between CD4^+^ cells and IL-13 [62]. Hypoxia induces the resistin-like alpha protein, which is associated with vascular remodeling [63]. The T_H_2 immune response also induces this protein. Dendritic cells (DCs) essential for activating naïve T cells are crucial antigen-presenting cells in the immune function. The ability of DCs to develop into many cell types, including endothelial cells (ECs), may play a significant function in the pathophysiology of vascular diseases [64]. The accumulation of immature DCs in altered pulmonary arteries in experimental and clinical PAH tissue samples suggests that they may play a role in the immunopathology of PAH [65]. Antibodies in the serum of patients with PAH and collagen vascular disease directed against fibroblasts and endothelial cells, in addition to nuclear antigens, may be a contributing factor in the formation of these antigen-presenting cells [66]. Wang et al. [67] found that PAH patients had a lower proportion of monocyte-derived DCs in their peripheral blood, suggesting the involvement of the T_H_1 immune response in the pathogenesis of PAH. During PAH, mast cells secrete the vascular endothelial growth factor, which may induce dysfunction in angiogenesis [68]. In addition, during PAH, perivascular mast cells produce chymase [69]. As chymase can induce localized angiotensin II production, endothelin activation, and matrix metalloprotease activation, it may be involved in vascular remodeling and vasomotor tone regulation. In PAH-associated fibrosis, mast cell chymase may be a significant target for the therapy of immune cell- and autoantibody-associated pulmonary hypertension [70]. The levels of total serum tryptase in PAH samples were significantly greater than those in control samples [71], indicating high mast cell counts or enhanced mast cell activation. Thus, multiple studies have identified the important role of immune cell infiltration in PAH.

There were a few problems with this research. First, increasing the number of individuals represented in the sample and filling out all genetic data will make the illness analysis and prediction more reliable. Second, to give reliable evidence for the development of targeted therapeutic medicines, the potential marker genes and pathways discovered in this study need to be confirmed in additional research. In the end, investigating the protein expression levels of marker genes may provide more proof of the possible roles that marker genes play in PAH. Additional research is necessary to validate the biological function for our results.

## 5. Conclusions

Overall, FAM171B has strong diagnostic utility and is associated with immune cell infiltration for PAH. We also discovered that resting memory CD4 T cells, activated dendritic cells, and resting mast cells may all play a role in the development and progression of PAH. Furthermore, FAM171B was significantly associated with resting mast cells and negatively associated with CD8 T cells and monocytes. These immune cells possibly affect PAH development, and further research into their action may help identify immunotherapeutic targets and improve immunomodulation-based PAH treatment.

## Figures and Tables

**Table 1:** Characteristics of three datasets.

Datasets	PAH	Tissue source	Normal	Tissue source	Platform
GSE113439	15	6 patients with idiopathic PAH, 4 patients with PAH secondary to connective tissue disease, 4 patients with PAH secondary to congenital heart disease, and 1 patient with chronic thromboembolic pulmonary hypertension	11	Tissue flanking lung cancer resections	GPL6244
GSE117261	58	Patients with PAH at transplant	25	Patients who do not have an appropriate recipient but still meet physiologic standards	GPL6244
GSE53408	12	The recipient's lung at the time of lung transplantation	11	Normal tissue of cancer patients undergoing surgery	GPL6244

PAH: pulmonary arterial hypertension.

**Table 2:** The top 20 up- and downregulated DEGs in PAH and normal samples.

Genes	Log2FC	AveExpr	*T*	*P* value	Adj. *P* value	*B*
Upregulated						
*HBB*	1.905556883	9.856392202	8.817638633	2.05E-14	5.16E-11	22.29719033
*POSTN*	1.706056477	9.004745628	7.883631545	2.56E-12	1.65E-09	17.65365235
*HBA2*	1.623246487	9.995273661	8.395393375	1.84E-13	3.35E-10	20.18546164
*SFRP2*	1.364599703	6.835061904	6.259692192	7.73E-09	6.77E-07	9.956619921
*VCAM1*	1.348484359	6.33270595	6.359079782	4.83E-09	4.68E-07	10.40761494
*PI15*	1.332181179	5.338197295	5.364027131	4.59E-07	1.68E-05	6.054507121
*COL14A1*	1.239026153	7.436785148	8.01864568	1.28E-12	1.03E-09	18.31809232
*ASPN*	1.234775416	7.13255928	5.807955926	6.29E-08	3.53E-06	7.949732975
*WIF1*	1.190095167	9.744898457	5.996873555	2.64E-08	1.78E-06	8.780077328
*RGS1*	1.141220379	7.504991123	6.443957755	3.22E-09	3.37E-07	10.79526605
*CCDC80*	1.12913297	8.080546692	5.183353767	1.01E-06	3.04E-05	5.30766633
*ENPP2*	1.028785935	9.494957558	5.544595076	2.06E-07	8.96E-06	6.815449702
*OGN*	1.012257941	7.501144893	5.987771459	2.75E-08	1.84E-06	8.739767044
*GEM*	0.983757223	7.038971522	6.81826404	5.28E-10	8.31E-08	12.53033356
*ESM1*	0.978601315	5.94799635	5.374557277	4.38E-07	1.63E-05	6.098491469
*HAS2*	0.971047451	6.507270998	3.905308804	0.000163209	0.001579558	0.510130208
*PDE3A*	0.937415523	7.215983585	8.816355258	2.07E-14	5.16E-11	22.29074747
*AGBL1*	0.928098439	6.426958445	5.706068912	1.00E-07	5.14E-06	7.507594521
*FABP4*	0.924223274	8.40626485	4.525883107	1.54E-05	0.000241578	2.725479162
*ANGPT2*	0.907969049	5.841628396	3.918091772	0.000155808	0.001526427	0.553359741
Downregulated						
*CSF3R*	-0.802339405	7.69132216	-9.322132247	1.46E-15	7.90E-12	24.83851182
*AQP9*	-0.806618791	7.940157165	-3.800645776	0.000237754	0.002124247	0.160190529
*NKD1*	-0.807008288	6.232544998	-8.187270593	5.40E-13	7.70E-10	19.15162494
*FCN3*	-0.894325549	10.56417571	-4.710864558	7.29E-06	0.000136457	3.42917004
*MSMB*	-0.923641782	4.535954481	-3.117142176	0.002333392	0.012605115	-1.939453915
*MGAM*	-0.963945588	5.678217693	-5.117611008	1.33E-06	3.74E-05	5.039674765
*SLCO4A1*	-0.975611085	7.118272517	-5.164935589	1.09E-06	3.21E-05	5.232380522
*CHIT1*	-0.981420709	5.221009621	-4.628773964	1.02E-05	0.000177714	3.114574322
*LOC441081*	-1.005779498	7.09466789	-7.367729041	3.48E-11	9.93E-09	15.14314687
*S100A8*	-1.041186811	10.0442234	-5.703814773	1.01E-07	5.14E-06	7.497859186
*SAA1*	-1.041252274	4.232431929	-5.085585452	1.53E-06	4.18E-05	4.909874548
*LCN2*	-1.051025788	6.391003715	-5.901638768	4.10E-08	2.48E-06	8.35982061
*SOSTDC1*	-1.098927062	6.477777826	-4.589229089	1.19E-05	0.000201156	2.964336963
*MS4A15*	-1.151439894	6.595835828	-5.303669318	5.97E-07	2.05E-05	5.803351239
*BPIFA1*	-1.157491285	4.694038015	-5.103986002	1.41E-06	3.91E-05	4.984392023
*RNASE2*	-1.163968826	5.60043777	-7.328408686	4.24E-11	1.18E-08	14.95389119
*S100A9*	-1.275518557	7.549020869	-7.393186829	3.07E-11	9.17E-09	15.26585058
*IL1R2*	-1.287495193	7.121522058	-4.727398557	6.82E-06	0.000129741	3.492972276
*S100A12*	-1.300940357	7.131519115	-5.502100612	2.49E-07	1.04E-05	6.635105221
*BPIFB1*	-1.955118819	6.8396727	-4.829346556	4.48E-06	9.46E-05	3.889569936

## Data Availability

The datasets used and analyzed during the current study are all available from the corresponding author.

## Supplementary Materials

Supplementary Materials Supplementary Table 1: The results of differentially expressed genes (DEGs). Supplementary Table 2: Gene Ontology (GO) enrichment analysis results of differentially expressed genes (DEGs). Supplementary Table 3: Kyoto Encyclopedia of Genes and Genomes (KEGG) enrichment analysis results of differentially expressed genes (DEGs). Supplementary Table 4: Disease Ontology (DO) enrichment analysis results of differentially expressed genes (DEGs). Supplementary Table 5: Metascape function analysis results of differentially expressed genes (DEGs). Supplementary Table 6: results of Gene Set Enrichment Analysis (GSEA) of gene expression matrix. Supplementary Table 7: results of all genes in brown module. Supplementary Table 8: results of key genes in brown module. Supplementary Table 9: results of analyzing the combined data matrix of GSE113439 and GSE117261 using CIBERSORT. Supplementary Table 10: results of the correlation of FAM171B with immune cells.
