# Supplementary material for: FAM171B as a Novel Biomarker Mediates Tissue Immune Microenvironment in Pulmonary Arterial Hypertension
Source: Mediators Inflamm. 2022 Sep 22;2022:1878766. doi: 10.1155/2022/1878766 (PMC9553458; doi:10.1155/2022/1878766)
Supplement: Supplementary Materials — Supplementary Table 1: The results of differentially expressed genes (DEGs). Supplementary Table 2: Gene Ontology (GO) enrichment analysis results of differentially expressed genes (DEGs). Supplementary Table 3: Kyoto Encyclopedia of Genes and Genomes (KEGG) enrichment analysis results of differentially expressed genes (DEGs). Supplementary Table 4: Disease Ontology (DO) enrichment analysis results of differentially expressed genes (DEGs). Supplementary Table 5: Metascape function analysis results of differentially expressed genes (DEGs). Supplementary Table 6: results of Gene Set Enrichment Analysis (GSEA) of gene expression matrix. Supplementary Table 7: results of all genes in brown module. Supplementary Table 8: results of key genes in brown module. Supplementary Table 9: results of analyzing the combined data matrix of GSE113439 and GSE117261 using CIBERSORT. Supplementary Table 10: results of the correlation of FAM171B with immune cells. [file 1878766.f1.zip › Supplementary Table1.docx]

| Gene | logFC | AveExpr | t | P.Value | adj.P.Val | B |
| --- | --- | --- | --- | --- | --- | --- |
| LTBP1 | 0.835256222 | 8.503645587 | 10.1791311 | 1.60E-17 | 1.60E-13 | 29.17750725 |
| CSF3R | -0.802339405 | 7.69132216 | -9.322132247 | 1.46E-15 | 7.90E-12 | 24.83851182 |
| ANKRD36C | 0.703122236 | 8.574638772 | 9.307094539 | 1.58E-15 | 7.90E-12 | 24.76255412 |
| HBB | 1.905556883 | 9.856392202 | 8.817638633 | 2.05E-14 | 5.16E-11 | 22.29719033 |
| PDE3A | 0.937415523 | 7.215983585 | 8.816355258 | 2.07E-14 | 5.16E-11 | 22.29074747 |
| HBA2 | 1.623246487 | 9.995273661 | 8.395393375 | 1.84E-13 | 3.35E-10 | 20.18546164 |
| NKD1 | -0.807008288 | 6.232544998 | -8.187270593 | 5.40E-13 | 7.70E-10 | 19.15162494 |
| PSD3 | 0.637080388 | 6.193440608 | 8.117937145 | 7.71E-13 | 8.66E-10 | 18.80842988 |
| SLC9A3R2 | -0.706269683 | 7.48012134 | -8.116828217 | 7.76E-13 | 8.66E-10 | 18.80294602 |
| PDE4D | 0.644278208 | 7.760884525 | 8.092191508 | 8.80E-13 | 8.66E-10 | 18.68115614 |
| COL14A1 | 1.239026153 | 7.436785148 | 8.01864568 | 1.28E-12 | 1.03E-09 | 18.31809232 |
| ECM2 | 0.772243434 | 6.188436109 | 7.953469436 | 1.79E-12 | 1.33E-09 | 17.99699951 |
| HIVEP1 | 0.612706559 | 7.982053735 | 7.914405728 | 2.19E-12 | 1.46E-09 | 17.80485626 |
| POSTN | 1.706056477 | 9.004745628 | 7.883631545 | 2.56E-12 | 1.65E-09 | 17.65365235 |
| PDE7B | 0.76394745 | 5.49316045 | 7.857723321 | 2.92E-12 | 1.82E-09 | 17.52647177 |
| ADRA1A | -0.696894856 | 4.814355108 | -7.799531042 | 3.93E-12 | 2.38E-09 | 17.24120505 |
| NT5E | 0.685878344 | 5.984762386 | 7.779310437 | 4.36E-12 | 2.56E-09 | 17.14221056 |
| FAM171B | 0.562048353 | 5.787403913 | 7.512463268 | 1.68E-11 | 6.36E-09 | 15.84248612 |
| ACSS2 | -0.501950186 | 7.94019674 | -7.511708863 | 1.69E-11 | 6.36E-09 | 15.83883021 |
| ANKRD36B | 0.545184682 | 8.377045881 | 7.50351165 | 1.76E-11 | 6.45E-09 | 15.7991129 |
| ANKRD36 | 0.578076304 | 7.994451045 | 7.501835945 | 1.78E-11 | 6.45E-09 | 15.79099534 |
| BICC1 | 0.701731163 | 6.558868812 | 7.481401 | 1.97E-11 | 6.96E-09 | 15.69204685 |
| TSHZ2 | 0.843259173 | 5.534909506 | 7.479536871 | 1.99E-11 | 6.96E-09 | 15.68302455 |
| AHI1 | 0.502414728 | 7.049378477 | 7.446149802 | 2.35E-11 | 7.62E-09 | 15.52154858 |
| FGR | -0.648925331 | 7.715379579 | -7.39747499 | 3.00E-11 | 9.17E-09 | 15.28653211 |
| S100A9 | -1.275518557 | 7.549020869 | -7.393186829 | 3.07E-11 | 9.17E-09 | 15.26585058 |
| KRT4 | -0.605339722 | 4.870543729 | -7.392470611 | 3.08E-11 | 9.17E-09 | 15.26239667 |
| LOC441081 | -1.005779498 | 7.09466789 | -7.367729041 | 3.48E-11 | 9.93E-09 | 15.14314687 |
| RNASE2 | -1.163968826 | 5.60043777 | -7.328408686 | 4.24E-11 | 1.18E-08 | 14.95389119 |
| STAT4 | 0.625849577 | 6.213188059 | 7.298170093 | 4.93E-11 | 1.26E-08 | 14.80856907 |
| ABCC9 | 0.745300005 | 7.975881897 | 7.298160365 | 4.93E-11 | 1.26E-08 | 14.80852235 |
| H1-0 | -0.531542478 | 8.149474752 | -7.296261271 | 4.98E-11 | 1.26E-08 | 14.79940209 |
| EPHA3 | 0.724351304 | 5.130515419 | 7.140938452 | 1.08E-10 | 2.37E-08 | 14.05615569 |
| ANTXR1 | 0.520707904 | 9.052568173 | 6.998714622 | 2.18E-10 | 4.19E-08 | 13.38045563 |
| HIVEP2 | 0.768779894 | 7.756657674 | 6.976106011 | 2.44E-10 | 4.55E-08 | 13.27349261 |
| MATN2 | 0.589182949 | 5.576902329 | 6.955163913 | 2.70E-10 | 4.98E-08 | 13.17452707 |
| RORA | 0.55373714 | 7.796392682 | 6.942593924 | 2.87E-10 | 5.17E-08 | 13.11517806 |
| GLT8D2 | 0.65656936 | 6.510096678 | 6.906756417 | 3.43E-10 | 5.91E-08 | 12.94619091 |
| ATP2B1 | 0.516067028 | 8.444429022 | 6.901643444 | 3.51E-10 | 6.00E-08 | 12.92210799 |
| RGS5 | 0.901928999 | 9.324054234 | 6.883769131 | 3.83E-10 | 6.43E-08 | 12.83796985 |
| PDE1A | 0.810225685 | 5.278416375 | 6.843416812 | 4.67E-10 | 7.58E-08 | 12.64832733 |
| GEM | 0.983757223 | 7.038971522 | 6.81826404 | 5.28E-10 | 8.31E-08 | 12.53033356 |
| HMCN1 | 0.797915545 | 7.995173014 | 6.755024057 | 7.19E-10 | 1.05E-07 | 12.23441548 |
| SLC7A7 | -0.514838367 | 8.03954599 | -6.70912905 | 8.99E-10 | 1.25E-07 | 12.02034056 |
| LILRB3 | -0.664982035 | 6.094281231 | -6.672874966 | 1.07E-09 | 1.40E-07 | 11.85164835 |
| PDCD4 | 0.515055287 | 8.493323339 | 6.659812503 | 1.14E-09 | 1.44E-07 | 11.79095853 |
| MYO1F | -0.55644171 | 6.961487215 | -6.640742663 | 1.25E-09 | 1.52E-07 | 11.70244459 |
| MACC1 | 0.800589461 | 7.302299557 | 6.588814019 | 1.61E-09 | 1.87E-07 | 11.46194315 |
| AHCYL2 | 0.569473013 | 9.3725548 | 6.559198149 | 1.85E-09 | 2.12E-07 | 11.32513179 |
| FZD7 | 0.538208121 | 6.138443683 | 6.548830755 | 1.95E-09 | 2.20E-07 | 11.27730032 |
| FREM1 | 0.572020321 | 5.370417117 | 6.547676055 | 1.96E-09 | 2.20E-07 | 11.27197491 |
| GGTA1 | 0.590706799 | 6.297449423 | 6.534399447 | 2.09E-09 | 2.32E-07 | 11.21077243 |
| RASGRP1 | 0.760340075 | 6.361105302 | 6.515332638 | 2.29E-09 | 2.51E-07 | 11.12297022 |
| S100A4 | -0.552354529 | 7.71661969 | -6.507448787 | 2.38E-09 | 2.58E-07 | 11.08669712 |
| RGS1 | 1.141220379 | 7.504991123 | 6.443957755 | 3.22E-09 | 3.37E-07 | 10.79526605 |
| GIMAP6 | -0.546776404 | 6.917123784 | -6.435016594 | 3.36E-09 | 3.44E-07 | 10.75432434 |
| SLC36A1 | -0.578935079 | 5.884713409 | -6.430701477 | 3.43E-09 | 3.50E-07 | 10.7345742 |
| TBX3 | -0.666844646 | 6.856448803 | -6.375194598 | 4.47E-09 | 4.42E-07 | 10.4810395 |
| VCAM1 | 1.348484359 | 6.33270595 | 6.359079782 | 4.83E-09 | 4.68E-07 | 10.40761494 |
| LUM | 0.551314409 | 10.57769826 | 6.340027414 | 5.28E-09 | 5.05E-07 | 10.32091271 |
| PLCB4 | 0.568047133 | 6.3883545 | 6.277529334 | 7.10E-09 | 6.45E-07 | 10.03732314 |
| SULF1 | 0.742135941 | 6.823594142 | 6.276610375 | 7.14E-09 | 6.45E-07 | 10.0331628 |
| ARID5B | 0.519244403 | 8.839458997 | 6.267461598 | 7.45E-09 | 6.60E-07 | 9.991759296 |
| SFRP2 | 1.364599703 | 6.835061904 | 6.259692192 | 7.73E-09 | 6.77E-07 | 9.956619921 |
| CBS | -0.561407002 | 5.43831295 | -6.160736432 | 1.23E-08 | 9.87E-07 | 9.510829905 |
| CFH | 0.712565218 | 8.83240725 | 6.125246335 | 1.45E-08 | 1.11E-06 | 9.351760775 |
| INHBA | 0.768112568 | 6.098707583 | 6.086722231 | 1.74E-08 | 1.30E-06 | 9.179588207 |
| SIGLEC9 | -0.519451813 | 5.477276806 | -6.053750101 | 2.03E-08 | 1.48E-06 | 9.032643241 |
| UACA | 0.575993918 | 7.934058148 | 6.051782296 | 2.05E-08 | 1.49E-06 | 9.023885638 |
| MFGE8 | -0.51030179 | 8.153231726 | -6.024687075 | 2.32E-08 | 1.60E-06 | 8.903440625 |
| CNTN1 | 0.564926718 | 5.230990233 | 6.009069955 | 2.50E-08 | 1.71E-06 | 8.834138459 |
| ZFPM2 | 0.512478625 | 6.941089374 | 5.997668717 | 2.63E-08 | 1.78E-06 | 8.783600278 |
| WIF1 | 1.190095167 | 9.744898457 | 5.996873555 | 2.64E-08 | 1.78E-06 | 8.780077328 |
| OGN | 1.012257941 | 7.501144893 | 5.987771459 | 2.75E-08 | 1.84E-06 | 8.739767044 |
| PDGFD | 0.737710175 | 5.02079863 | 5.925991659 | 3.66E-08 | 2.26E-06 | 8.466966688 |
| LCN2 | -1.051025788 | 6.391003715 | -5.901638768 | 4.10E-08 | 2.48E-06 | 8.35982061 |
| NQO1 | -0.738924635 | 6.852149243 | -5.826913826 | 5.77E-08 | 3.32E-06 | 8.032448452 |
| ASPN | 1.234775416 | 7.13255928 | 5.807955926 | 6.29E-08 | 3.53E-06 | 7.949732975 |
| XAF1 | 0.627092311 | 8.533659368 | 5.779211815 | 7.17E-08 | 3.92E-06 | 7.824584949 |
| DPYSL3 | 0.502316702 | 7.384079907 | 5.778602302 | 7.19E-08 | 3.92E-06 | 7.821934695 |
| FAT3 | 0.697750806 | 5.272295535 | 5.746649595 | 8.32E-08 | 4.45E-06 | 7.683203808 |
| MALL | 0.525700254 | 9.460361543 | 5.730480183 | 8.95E-08 | 4.70E-06 | 7.61315364 |
| ITGAM | -0.580778341 | 6.998833672 | -5.707445299 | 9.94E-08 | 5.13E-06 | 7.513539954 |
| AGBL1 | 0.928098439 | 6.426958445 | 5.706068912 | 1.00E-07 | 5.14E-06 | 7.507594521 |
| S100A8 | -1.041186811 | 10.0442234 | -5.703814773 | 1.01E-07 | 5.14E-06 | 7.497859186 |
| GLT1D1 | -0.571287669 | 4.887782522 | -5.694726265 | 1.05E-07 | 5.28E-06 | 7.458627779 |
| LRRN4 | -0.749135806 | 7.18797263 | -5.677394029 | 1.14E-07 | 5.57E-06 | 7.383903652 |
| PLCB1 | 0.577952982 | 6.555728309 | 5.650823596 | 1.28E-07 | 6.22E-06 | 7.269587053 |
| PHGDH | -0.684221123 | 5.924575521 | -5.637255593 | 1.36E-07 | 6.48E-06 | 7.21132295 |
| SIGLEC10 | -0.695301673 | 5.712068344 | -5.622267187 | 1.46E-07 | 6.87E-06 | 7.147046948 |
| CCN5 | -0.660698036 | 6.41027262 | -5.616943053 | 1.49E-07 | 6.99E-06 | 7.124237257 |
| CDON | 0.569613538 | 6.045189416 | 5.590768808 | 1.68E-07 | 7.63E-06 | 7.012271624 |
| PROK2 | -0.694481472 | 5.868240781 | -5.590531795 | 1.68E-07 | 7.63E-06 | 7.011259052 |
| PIEZO2 | 0.813499326 | 7.511715858 | 5.546573983 | 2.05E-07 | 8.90E-06 | 6.82386672 |
| ENPP2 | 1.028785935 | 9.494957558 | 5.544595076 | 2.06E-07 | 8.96E-06 | 6.815449702 |
| TCN2 | -0.53764156 | 6.757647238 | -5.536500994 | 2.14E-07 | 9.17E-06 | 6.781039793 |
| ABCG2 | 0.746973709 | 5.957582416 | 5.51419243 | 2.36E-07 | 9.96E-06 | 6.686344229 |
| S100A12 | -1.300940357 | 7.131519115 | -5.502100612 | 2.49E-07 | 1.04E-05 | 6.635105221 |
| CA4 | -0.622527409 | 5.889600205 | -5.456490019 | 3.05E-07 | 1.23E-05 | 6.442394819 |
| BMP6 | 0.725419927 | 7.202760794 | 5.44342439 | 3.24E-07 | 1.29E-05 | 6.387356469 |
| AFF2 | -0.506670762 | 5.641520501 | -5.429155765 | 3.45E-07 | 1.35E-05 | 6.327335413 |
| RASEF | 0.52045826 | 5.612541803 | 5.410404228 | 3.74E-07 | 1.44E-05 | 6.248592312 |
| SEC14L4 | -0.754356005 | 5.160539856 | -5.396060575 | 3.99E-07 | 1.50E-05 | 6.188463448 |
| SHMT2 | -0.527621057 | 7.437252083 | -5.390909043 | 4.08E-07 | 1.53E-05 | 6.166890276 |
| MXRA5 | 0.763476889 | 6.500568112 | 5.390420795 | 4.09E-07 | 1.53E-05 | 6.164846239 |
| ESM1 | 0.978601315 | 5.94799635 | 5.374557277 | 4.38E-07 | 1.63E-05 | 6.098491469 |
| PI15 | 1.332181179 | 5.338197295 | 5.364027131 | 4.59E-07 | 1.68E-05 | 6.054507121 |
| PLPPR4 | 0.58337952 | 5.255536127 | 5.356240449 | 4.75E-07 | 1.73E-05 | 6.022013979 |
| ZNF521 | 0.617679605 | 6.172232151 | 5.305756551 | 5.92E-07 | 2.04E-05 | 5.81200903 |
| MS4A15 | -1.151439894 | 6.595835828 | -5.303669318 | 5.97E-07 | 2.05E-05 | 5.803351239 |
| ADORA3 | -0.649318717 | 5.460381505 | -5.301029505 | 6.04E-07 | 2.07E-05 | 5.792404191 |
| LILRA1 | -0.597435449 | 5.359669692 | -5.299210628 | 6.09E-07 | 2.08E-05 | 5.784863327 |
| SNORD114-2 | 0.63694382 | 4.128817973 | 5.294103212 | 6.23E-07 | 2.11E-05 | 5.763696579 |
| CD69 | 0.820552193 | 7.79389906 | 5.288313516 | 6.39E-07 | 2.15E-05 | 5.739716606 |
| MYH10 | 0.508502415 | 9.08524018 | 5.274385569 | 6.78E-07 | 2.27E-05 | 5.682091985 |
| CCL21 | 0.842460516 | 8.344516023 | 5.27269633 | 6.83E-07 | 2.28E-05 | 5.675109062 |
| CD14 | -0.630645244 | 8.786023667 | -5.250556809 | 7.52E-07 | 2.45E-05 | 5.583710491 |
| ABCA8 | 0.645907463 | 7.911345039 | 5.250185103 | 7.54E-07 | 2.45E-05 | 5.582177902 |
| ALAS2 | 0.564568143 | 4.784492839 | 5.24920809 | 7.57E-07 | 2.46E-05 | 5.578149863 |
| SECISBP2L | 0.529085772 | 9.300888883 | 5.237252236 | 7.97E-07 | 2.55E-05 | 5.528893838 |
| IGF1 | 0.761160511 | 6.75524236 | 5.234657449 | 8.06E-07 | 2.57E-05 | 5.518212499 |
| GFRA1 | 0.503827552 | 5.526586472 | 5.23330176 | 8.11E-07 | 2.58E-05 | 5.5126331 |
| ITGB6 | 0.533965125 | 9.013607957 | 5.227434127 | 8.32E-07 | 2.64E-05 | 5.488494409 |
| C3AR1 | -0.573765253 | 8.592926231 | -5.202074009 | 9.28E-07 | 2.87E-05 | 5.38435027 |
| CCDC80 | 1.12913297 | 8.080546692 | 5.183353767 | 1.01E-06 | 3.04E-05 | 5.30766633 |
| RNF182 | -0.530339704 | 5.239020519 | -5.173439536 | 1.05E-06 | 3.12E-05 | 5.267121228 |
| SLCO4A1 | -0.975611085 | 7.118272517 | -5.164935589 | 1.09E-06 | 3.21E-05 | 5.232380522 |
| TMEM45B | -0.576912487 | 6.363313561 | -5.163062089 | 1.10E-06 | 3.23E-05 | 5.224731409 |
| ACE2 | 0.53851449 | 4.702579921 | 5.15849999 | 1.12E-06 | 3.27E-05 | 5.206112247 |
| C5 | 0.62770829 | 7.434695049 | 5.134597285 | 1.24E-06 | 3.54E-05 | 5.10872021 |
| KIT | 0.749980105 | 8.090309395 | 5.125722116 | 1.29E-06 | 3.64E-05 | 5.072627414 |
| MGAM | -0.963945588 | 5.678217693 | -5.117611008 | 1.33E-06 | 3.74E-05 | 5.039674765 |
| BPIFA1 | -1.157491285 | 4.694038015 | -5.103986002 | 1.41E-06 | 3.91E-05 | 4.984392023 |
| FAM13C | 0.611166661 | 6.744478996 | 5.098644715 | 1.45E-06 | 3.99E-05 | 4.962744376 |
| SAA1 | -1.041252274 | 4.232431929 | -5.085585452 | 1.53E-06 | 4.18E-05 | 4.909874548 |
| CXCR2 | -0.6475612 | 5.743660602 | -5.08465484 | 1.53E-06 | 4.18E-05 | 4.906110149 |
| PDLIM3 | 0.66906253 | 6.911354891 | 5.075887443 | 1.59E-06 | 4.31E-05 | 4.87066594 |
| LGALSL | 0.51120858 | 8.995047524 | 5.052452983 | 1.76E-06 | 4.67E-05 | 4.77610995 |
| WEE1 | 0.608658511 | 6.337607773 | 5.031547874 | 1.92E-06 | 5.01E-05 | 4.691985814 |
| PLAC8 | -0.544132479 | 8.418615603 | -5.026625581 | 1.96E-06 | 5.08E-05 | 4.6722092 |
| CFAP69 | 0.505606491 | 5.36500455 | 4.986199034 | 2.33E-06 | 5.80E-05 | 4.510236971 |
| TRPC6 | 0.568763359 | 7.028573099 | 4.984372613 | 2.35E-06 | 5.82E-05 | 4.502938381 |
| GBP5 | 0.752393019 | 5.840899278 | 4.965298843 | 2.54E-06 | 6.15E-05 | 4.426816714 |
| RGS13 | 0.539633604 | 5.679987525 | 4.947170023 | 2.74E-06 | 6.55E-05 | 4.354634864 |
| LRRC36 | 0.551837248 | 5.690346963 | 4.942519849 | 2.80E-06 | 6.64E-05 | 4.33614628 |
| CPA3 | 0.853856006 | 8.915208073 | 4.917459494 | 3.11E-06 | 7.21E-05 | 4.236696723 |
| FGFR2 | 0.599004553 | 7.900946387 | 4.889886815 | 3.49E-06 | 7.84E-05 | 4.127644983 |
| ACADL | 0.690432517 | 7.891962406 | 4.856708242 | 4.00E-06 | 8.75E-05 | 3.996936304 |
| MME | 0.709116534 | 8.820421744 | 4.840337443 | 4.28E-06 | 9.16E-05 | 3.932651298 |
| SEMA3D | 0.744741436 | 6.475711351 | 4.830562959 | 4.46E-06 | 9.44E-05 | 3.894334835 |
| CNKSR3 | 0.552998833 | 6.3652258 | 4.829379495 | 4.48E-06 | 9.46E-05 | 3.889698956 |
| BPIFB1 | -1.955118819 | 6.8396727 | -4.829346556 | 4.48E-06 | 9.46E-05 | 3.889569936 |
| EPHA4 | 0.529367769 | 6.645816555 | 4.819790055 | 4.66E-06 | 9.76E-05 | 3.852161925 |
| SULT1B1 | -0.673803688 | 5.653325627 | -4.811950719 | 4.82E-06 | 0.000100388 | 3.821511059 |
| CXCR1 | -0.74884973 | 6.053454694 | -4.811442473 | 4.83E-06 | 0.000100389 | 3.819524981 |
| C7 | 0.540042808 | 10.47801047 | 4.810847876 | 4.84E-06 | 0.000100427 | 3.817201642 |
| ITK | 0.709211496 | 6.283537326 | 4.809235344 | 4.87E-06 | 0.000100825 | 3.810901734 |
| NR1D2 | 0.648366255 | 7.694862919 | 4.806517432 | 4.93E-06 | 0.000101082 | 3.800286347 |
| OVOS2 | 0.528193811 | 4.987260901 | 4.802870695 | 5.00E-06 | 0.000101986 | 3.786049295 |
| IL1R2 | -1.287495193 | 7.121522058 | -4.727398557 | 6.82E-06 | 0.000129741 | 3.492972276 |
| FCN3 | -0.894325549 | 10.56417571 | -4.710864558 | 7.29E-06 | 0.000136457 | 3.42917004 |
| LILRA3 | -0.520135689 | 4.947741537 | -4.702927203 | 7.53E-06 | 0.000140155 | 3.398592928 |
| LILRB2 | -0.514069364 | 6.324748377 | -4.701451336 | 7.58E-06 | 0.000140869 | 3.392911165 |
| HGF | 0.581396131 | 6.847212629 | 4.698285623 | 7.68E-06 | 0.000142167 | 3.380727807 |
| ITGA2 | 0.843116217 | 8.082332406 | 4.66652071 | 8.73E-06 | 0.000157327 | 3.258778147 |
| PAMR1 | 0.632742225 | 5.164620889 | 4.64663661 | 9.47E-06 | 0.000167689 | 3.182718299 |
| CHIT1 | -0.981420709 | 5.221009621 | -4.628773964 | 1.02E-05 | 0.000177714 | 3.114574322 |
| SOSTDC1 | -1.098927062 | 6.477777826 | -4.589229089 | 1.19E-05 | 0.000201156 | 2.964336963 |
| SLC18A2 | 0.652668827 | 5.916478928 | 4.584212008 | 1.22E-05 | 0.000204041 | 2.945337833 |
| SLC7A2 | 0.560128132 | 8.023031649 | 4.578930919 | 1.24E-05 | 0.000208234 | 2.925353974 |
| ACOXL | 0.568251436 | 7.115631435 | 4.574158108 | 1.27E-05 | 0.000211192 | 2.90730675 |
| FPR1 | -0.603657082 | 7.305543118 | -4.554183564 | 1.37E-05 | 0.000223529 | 2.831915085 |
| BCHE | 0.602277261 | 5.443975383 | 4.548583028 | 1.40E-05 | 0.000226379 | 2.810816355 |
| FABP4 | 0.924223274 | 8.40626485 | 4.525883107 | 1.54E-05 | 0.000241578 | 2.725479162 |
| CXCL12 | 0.644209308 | 8.365761678 | 4.523096773 | 1.55E-05 | 0.000243455 | 2.715024221 |
| SELP | 0.602436248 | 7.063530997 | 4.477864792 | 1.86E-05 | 0.000279518 | 2.545915967 |
| ABCB1 | 0.715783023 | 5.10862794 | 4.475033834 | 1.88E-05 | 0.000282027 | 2.535370404 |
| THY1 | 0.856956796 | 6.346132713 | 4.474168319 | 1.89E-05 | 0.000282569 | 2.532147198 |
| CA2 | 0.583558759 | 7.526915572 | 4.467143408 | 1.94E-05 | 0.000289005 | 2.506001947 |
| MS4A2 | 0.691528277 | 5.595688394 | 4.461968796 | 1.98E-05 | 0.000293219 | 2.486761096 |
| RFTN2 | 0.631543386 | 6.491500041 | 4.433714806 | 2.21E-05 | 0.000320368 | 2.381973595 |
| SYNPO2 | 0.580511925 | 7.532079127 | 4.424647993 | 2.29E-05 | 0.00033007 | 2.348443882 |
| BMP5 | 0.545867702 | 7.350898474 | 4.424125957 | 2.30E-05 | 0.000330509 | 2.346514797 |
| F11 | 0.500552812 | 5.878018175 | 4.422669111 | 2.31E-05 | 0.000332165 | 2.341132125 |
| HIF3A | -0.662118157 | 6.613833322 | -4.420718673 | 2.33E-05 | 0.000333756 | 2.333927668 |
| HCK | -0.533262066 | 7.521714621 | -4.41127388 | 2.42E-05 | 0.000344618 | 2.299071862 |
| CA12 | 0.531822293 | 5.416993558 | 4.408528041 | 2.44E-05 | 0.0003476 | 2.288948063 |
| GXYLT2 | 0.500384464 | 6.78858442 | 4.405116203 | 2.48E-05 | 0.000351275 | 2.276374818 |
| PDE8B | 0.614348144 | 6.078769628 | 4.376489296 | 2.77E-05 | 0.000383282 | 2.171145149 |
| CR1 | -0.662296886 | 5.955927475 | -4.351990638 | 3.05E-05 | 0.000413643 | 2.081469283 |
| ANPEP | -0.678583843 | 6.970951366 | -4.318856018 | 3.46E-05 | 0.000460324 | 1.960741613 |
| TRAT1 | 0.540119725 | 5.485562053 | 4.274662923 | 4.11E-05 | 0.000531556 | 1.800731414 |
| RIPOR2 | -0.545284384 | 6.563785569 | -4.271510001 | 4.16E-05 | 0.000536068 | 1.789359992 |
| CCR1 | -0.609602812 | 5.761725763 | -4.261221535 | 4.32E-05 | 0.000553017 | 1.752294614 |
| IL13RA2 | 0.597933776 | 4.539108792 | 4.129249037 | 7.14E-05 | 0.000823212 | 1.282519681 |
| ITGAX | -0.515648231 | 7.437614646 | -4.121699011 | 7.34E-05 | 0.000841825 | 1.255966175 |
| VSIG4 | -0.562031016 | 9.067644101 | -4.03087382 | 0.000103019 | 0.001099346 | 0.939311551 |
| FAM107A | -0.599314238 | 7.697213401 | -4.02510745 | 0.000105241 | 0.001118869 | 0.91938212 |
| BIRC3 | 0.552824789 | 8.134039461 | 4.018381527 | 0.00010789 | 0.00113915 | 0.896162869 |
| TM4SF18 | 0.750473381 | 7.106777383 | 4.005584366 | 0.000113108 | 0.001179893 | 0.852063516 |
| GPR146 | -0.508474978 | 6.482956128 | -3.992856465 | 0.000118537 | 0.001226905 | 0.808305864 |
| VNN2 | -0.682761233 | 6.893368301 | -3.974699029 | 0.000126714 | 0.001296752 | 0.746060264 |
| ADAMTS9 | 0.692596378 | 8.269431148 | 3.945480895 | 0.000141014 | 0.001414103 | 0.646339759 |
| ANGPT2 | 0.907969049 | 5.841628396 | 3.918091772 | 0.000155808 | 0.001526427 | 0.553359741 |
| TFPI2 | 0.701124968 | 5.2346119 | 3.911030708 | 0.000159855 | 0.001555397 | 0.529467506 |
| LRRC32 | -0.61407734 | 7.162012603 | -3.90781636 | 0.000161731 | 0.001570585 | 0.518601939 |
| HAS2 | 0.971047451 | 6.507270998 | 3.905308804 | 0.000163209 | 0.001579558 | 0.510130208 |
| AREG | 0.735632236 | 8.264570717 | 3.895520189 | 0.000169102 | 0.001621653 | 0.4770986 |
| AKR1C2 | -0.512194272 | 6.062432875 | -3.89264053 | 0.000170873 | 0.001634719 | 0.467393058 |
| EGR1 | 0.692591353 | 9.472749629 | 3.884944262 | 0.000175696 | 0.001670452 | 0.441480174 |
| MFAP4 | 0.564732038 | 9.872788668 | 3.867833769 | 0.000186887 | 0.001755948 | 0.384008424 |
| BTNL9 | -0.719586702 | 7.345494636 | -3.863158326 | 0.000190061 | 0.001779432 | 0.368337518 |
| ELF5 | -0.642788495 | 5.722619967 | -3.836558339 | 0.00020912 | 0.001915274 | 0.27945358 |
| SERPINE2 | 0.621080348 | 5.769893888 | 3.816186289 | 0.000224933 | 0.002030297 | 0.211694918 |
| STEAP2 | 0.590728089 | 7.603820104 | 3.815504975 | 0.000225481 | 0.002033405 | 0.209433556 |
| TLL1 | 0.605060545 | 5.294616627 | 3.812804658 | 0.000227666 | 0.002047556 | 0.200473909 |
| AQP9 | -0.806618791 | 7.940157165 | -3.800645776 | 0.000237754 | 0.002124247 | 0.160190529 |
| OLR1 | 0.572111456 | 8.52899395 | 3.769600797 | 0.000265476 | 0.002315639 | 0.05778102 |
| SLCO1A2 | -0.500294633 | 4.518519733 | -3.693851794 | 0.000346574 | 0.00286886 | -0.189389123 |
| IFI44L | 0.665948226 | 7.674751523 | 3.636700795 | 0.000422765 | 0.003337654 | -0.373303827 |
| FRAS1 | 0.511870344 | 6.19806211 | 3.604432754 | 0.000472527 | 0.003639846 | -0.476156778 |
| VIPR1 | -0.734464804 | 8.801150052 | -3.580114249 | 0.000513638 | 0.003887523 | -0.553196555 |
| COLEC10 | 0.532268992 | 4.678808737 | 3.57531822 | 0.000522134 | 0.003933938 | -0.568341798 |
| SFRP4 | 0.656203987 | 5.748800845 | 3.572230314 | 0.000527675 | 0.003968746 | -0.578084562 |
| HMOX1 | -0.710995155 | 7.184310252 | -3.535715138 | 0.00059755 | 0.004363822 | -0.692791879 |
| COL6A6 | 0.699412321 | 7.739490695 | 3.523102666 | 0.000623651 | 0.004524642 | -0.732195916 |
| TIMP4 | -0.635295804 | 5.369527919 | -3.469325358 | 0.000747483 | 0.005227409 | -0.898953088 |
| PRRX1 | 0.512466423 | 6.661449555 | 3.454590802 | 0.000785253 | 0.005409173 | -0.944286665 |
| CXCL9 | 0.707688689 | 6.141534381 | 3.41986341 | 0.000881496 | 0.005925907 | -1.050521171 |
| ERAP2 | 0.72920483 | 7.184884132 | 3.405468166 | 0.000924553 | 0.006141569 | -1.09430521 |
| ITGB3 | 0.666809728 | 7.02079762 | 3.384786026 | 0.000989883 | 0.006480003 | -1.156951128 |
| COL6A3 | 0.523882088 | 9.338567353 | 3.346964371 | 0.001120698 | 0.007155825 | -1.270715449 |
| ADGRF1 | -0.530413801 | 4.823152047 | -3.322727941 | 0.001212864 | 0.007598359 | -1.343072079 |
| MUC5B | -0.509897816 | 4.798313152 | -3.303199786 | 0.001292239 | 0.008002742 | -1.40106145 |
| COL15A1 | 0.561668323 | 5.621638003 | 3.283853923 | 0.001375644 | 0.008425189 | -1.458234832 |
| ROBO2 | 0.620932727 | 6.448366575 | 3.270583441 | 0.001435739 | 0.008734298 | -1.497294886 |
| MEDAG | 0.629892322 | 5.840565785 | 3.243254762 | 0.001567302 | 0.009318777 | -1.577325566 |
| CCL5 | 0.51089866 | 7.815664896 | 3.235670436 | 0.001605761 | 0.009507895 | -1.599438242 |
| TNFAIP3 | 0.524722299 | 8.570577496 | 3.218249958 | 0.001697456 | 0.009927182 | -1.650067834 |
| SERPINA3 | -0.649501301 | 8.188218095 | -3.204149812 | 0.001775224 | 0.010246927 | -1.690882672 |
| GZMK | 0.646606046 | 6.425101739 | 3.172343346 | 0.001963001 | 0.011052817 | -1.782407525 |
| FAP | 0.552771349 | 5.195828617 | 3.145774917 | 0.002133854 | 0.011775715 | -1.858280002 |
| PTGS2 | 0.66156493 | 7.443348765 | 3.141116682 | 0.002165198 | 0.011899352 | -1.871528139 |
| OLFM4 | -0.50019009 | 4.267330876 | -3.130900776 | 0.002235442 | 0.012214785 | -1.900525294 |
| MSMB | -0.923641782 | 4.535954481 | -3.117142176 | 0.002333392 | 0.012605115 | -1.939453915 |
| DPT | 0.50832369 | 7.559915222 | 3.10473047 | 0.002425157 | 0.01297446 | -1.974449041 |
| CPB2 | 0.664604357 | 7.964232904 | 3.071295934 | 0.002689274 | 0.014045 | -2.068138086 |
| SELE | 0.893271298 | 5.488958932 | 3.064901597 | 0.002742727 | 0.014227405 | -2.085959335 |
| THBS2 | 0.510966033 | 7.105666426 | 3.021908312 | 0.003128458 | 0.015733703 | -2.204972901 |
| EDN1 | 0.646621819 | 7.730108474 | 3.014595907 | 0.003198852 | 0.016031194 | -2.225074206 |
| DEPP1 | 0.601183812 | 8.73938281 | 2.998575821 | 0.003358214 | 0.0166661 | -2.26896872 |
| CD163 | -0.629063088 | 9.192533298 | -2.991181745 | 0.003434212 | 0.016921885 | -2.289161614 |
| ANKRD1 | 0.733365423 | 9.193185101 | 2.958648761 | 0.003787817 | 0.018320627 | -2.37750628 |
| ITGBL1 | 0.515423127 | 7.039525783 | 2.950020774 | 0.003887067 | 0.018648655 | -2.400798468 |
| IDO1 | 0.707044977 | 7.199082613 | 2.913603907 | 0.004332924 | 0.020241263 | -2.498472101 |
| ROPN1L | -0.535897106 | 5.964880594 | -2.771010341 | 0.006568474 | 0.027909373 | -2.870899855 |
| TSPAN1 | -0.63583098 | 6.977195565 | -2.751457778 | 0.006946125 | 0.029111359 | -2.920709695 |
| CHI3L2 | -0.529506254 | 7.233259457 | -2.704599371 | 0.007932936 | 0.032330883 | -3.038832856 |
