# Supplementary material for: FAM171B as a Novel Biomarker Mediates Tissue Immune Microenvironment in Pulmonary Arterial Hypertension
Source: Mediators Inflamm. 2022 Sep 22;2022:1878766. doi: 10.1155/2022/1878766 (PMC9553458; doi:10.1155/2022/1878766)
Supplement: Supplementary Materials — Supplementary Table 1: The results of differentially expressed genes (DEGs). Supplementary Table 2: Gene Ontology (GO) enrichment analysis results of differentially expressed genes (DEGs). Supplementary Table 3: Kyoto Encyclopedia of Genes and Genomes (KEGG) enrichment analysis results of differentially expressed genes (DEGs). Supplementary Table 4: Disease Ontology (DO) enrichment analysis results of differentially expressed genes (DEGs). Supplementary Table 5: Metascape function analysis results of differentially expressed genes (DEGs). Supplementary Table 6: results of Gene Set Enrichment Analysis (GSEA) of gene expression matrix. Supplementary Table 7: results of all genes in brown module. Supplementary Table 8: results of key genes in brown module. Supplementary Table 9: results of analyzing the combined data matrix of GSE113439 and GSE117261 using CIBERSORT. Supplementary Table 10: results of the correlation of FAM171B with immune cells. [file 1878766.f1.zip › Supplementary Table10.docx]

| Gene | Cell | cor | pvalue |
| --- | --- | --- | --- |
| FAM171B | B cells naive | 0.167991845 | 0.080822 |
| FAM171B | B cells memory | 0.001521066 | 0.987475867 |
| FAM171B | Plasma cells | 0.168807339 | 0.079343939 |
| FAM171B | T cells CD8 | -0.195505514 | 0.041764994 |
| FAM171B | T cells CD4 naive | -0.041490585 | 0.668385429 |
| FAM171B | T cells CD4 memory resting | 0.154415717 | 0.108828979 |
| FAM171B | T cells CD4 memory activated | 0.098971047 | 0.305886378 |
| FAM171B | T cells follicular helper | 0.076639055 | 0.428316084 |
| FAM171B | T cells regulatory (Tregs) | -0.088688748 | 0.35910516 |
| FAM171B | T cells gamma delta | -0.091154969 | 0.345845427 |
| FAM171B | NK cells resting | -0.116118878 | 0.229203797 |
| FAM171B | NK cells activated | -0.002836324 | 0.976648651 |
| FAM171B | Monocytes | -0.21578167 | 0.024405365 |
| FAM171B | Macrophages M0 | -0.025804508 | 0.78997323 |
| FAM171B | Macrophages M1 | -0.069752487 | 0.471081738 |
| FAM171B | Macrophages M2 | -0.109100176 | 0.258345639 |
| FAM171B | Dendritic cells resting | -0.128445773 | 0.183164719 |
| FAM171B | Dendritic cells activated | 0.186841319 | 0.051731804 |
| FAM171B | Mast cells resting | 0.277842647 | 0.003546737 |
| FAM171B | Mast cells activated | -0.107394828 | 0.266341749 |
| FAM171B | Eosinophils | 0.138370291 | 0.151319717 |
| FAM171B | Neutrophils | -0.187378371 | 0.051171842 |
