# Supplementary material for: FAM171B as a Novel Biomarker Mediates Tissue Immune Microenvironment in Pulmonary Arterial Hypertension
Source: Mediators Inflamm. 2022 Sep 22;2022:1878766. doi: 10.1155/2022/1878766 (PMC9553458; doi:10.1155/2022/1878766)
Supplement: Supplementary Materials — Supplementary Table 1: The results of differentially expressed genes (DEGs). Supplementary Table 2: Gene Ontology (GO) enrichment analysis results of differentially expressed genes (DEGs). Supplementary Table 3: Kyoto Encyclopedia of Genes and Genomes (KEGG) enrichment analysis results of differentially expressed genes (DEGs). Supplementary Table 4: Disease Ontology (DO) enrichment analysis results of differentially expressed genes (DEGs). Supplementary Table 5: Metascape function analysis results of differentially expressed genes (DEGs). Supplementary Table 6: results of Gene Set Enrichment Analysis (GSEA) of gene expression matrix. Supplementary Table 7: results of all genes in brown module. Supplementary Table 8: results of key genes in brown module. Supplementary Table 9: results of analyzing the combined data matrix of GSE113439 and GSE117261 using CIBERSORT. Supplementary Table 10: results of the correlation of FAM171B with immune cells. [file 1878766.f1.zip › Supplementary Table2.docx]

| ONTOLOGY | ID | Description | GeneRatio | pvalue | p.adjust | qvalue | geneID | Count |
| --- | --- | --- | --- | --- | --- | --- | --- | --- |
| BP | GO:0097529 | myeloid leukocyte migration | 19/240 | 5.00E-11 | 8.63E-08 | 6.47E-08 | CSF3R/S100A9/PDGFD/S100A8/PLCB1/S100A12/CCL21/C3AR1/C5/KIT/SAA1/CXCR2/CXCR1/CXCL12/RIPOR2/CCR1/CXCL9/CCL5/EDN1 | 19 |
| BP | GO:0042119 | neutrophil activation | 28/240 | 5.24E-11 | 8.63E-08 | 6.47E-08 | HBB/FGR/S100A9/RNASE2/LILRB3/SIGLEC9/LCN2/ITGAM/S100A8/S100A12/CD14/C3AR1/MGAM/CXCR2/PLAC8/MME/CXCR1/LILRA3/LILRB2/CHIT1/FPR1/CR1/ANPEP/ITGAX/OLR1/CCL5/SERPINA3/OLFM4 | 28 |
| BP | GO:0043312 | neutrophil degranulation | 27/240 | 1.36E-10 | 1.28E-07 | 9.61E-08 | HBB/FGR/S100A9/RNASE2/LILRB3/SIGLEC9/LCN2/ITGAM/S100A8/S100A12/CD14/C3AR1/MGAM/CXCR2/PLAC8/MME/CXCR1/LILRA3/LILRB2/CHIT1/FPR1/CR1/ANPEP/ITGAX/OLR1/SERPINA3/OLFM4 | 27 |
| BP | GO:0002283 | neutrophil activation involved in immune response | 27/240 | 1.56E-10 | 1.28E-07 | 9.61E-08 | HBB/FGR/S100A9/RNASE2/LILRB3/SIGLEC9/LCN2/ITGAM/S100A8/S100A12/CD14/C3AR1/MGAM/CXCR2/PLAC8/MME/CXCR1/LILRA3/LILRB2/CHIT1/FPR1/CR1/ANPEP/ITGAX/OLR1/SERPINA3/OLFM4 | 27 |
| BP | GO:0002446 | neutrophil mediated immunity | 27/240 | 2.55E-10 | 1.68E-07 | 1.26E-07 | HBB/FGR/S100A9/RNASE2/LILRB3/SIGLEC9/LCN2/ITGAM/S100A8/S100A12/CD14/C3AR1/MGAM/CXCR2/PLAC8/MME/CXCR1/LILRA3/LILRB2/CHIT1/FPR1/CR1/ANPEP/ITGAX/OLR1/SERPINA3/OLFM4 | 27 |
| BP | GO:0030593 | neutrophil chemotaxis | 13/240 | 6.43E-10 | 3.53E-07 | 2.65E-07 | CSF3R/S100A9/S100A8/S100A12/CCL21/C3AR1/SAA1/CXCR2/CXCR1/RIPOR2/CXCL9/CCL5/EDN1 | 13 |
| BP | GO:0030198 | extracellular matrix organization | 23/240 | 1.32E-09 | 5.58E-07 | 4.18E-07 | COL14A1/ECM2/POSTN/ANTXR1/VCAM1/LUM/SULF1/SFRP2/ITGAM/ITGB6/CCDC80/ITGA2/ITGAX/ADAMTS9/HAS2/MFAP4/TLL1/ITGB3/COL6A3/COL15A1/FAP/DPT/CPB2 | 23 |
| BP | GO:0043062 | extracellular structure organization | 23/240 | 1.38E-09 | 5.58E-07 | 4.18E-07 | COL14A1/ECM2/POSTN/ANTXR1/VCAM1/LUM/SULF1/SFRP2/ITGAM/ITGB6/CCDC80/ITGA2/ITGAX/ADAMTS9/HAS2/MFAP4/TLL1/ITGB3/COL6A3/COL15A1/FAP/DPT/CPB2 | 23 |
| BP | GO:0045229 | external encapsulating structure organization | 23/240 | 1.52E-09 | 5.58E-07 | 4.18E-07 | COL14A1/ECM2/POSTN/ANTXR1/VCAM1/LUM/SULF1/SFRP2/ITGAM/ITGB6/CCDC80/ITGA2/ITGAX/ADAMTS9/HAS2/MFAP4/TLL1/ITGB3/COL6A3/COL15A1/FAP/DPT/CPB2 | 23 |
| BP | GO:0060326 | cell chemotaxis | 20/240 | 2.53E-09 | 8.33E-07 | 6.24E-07 | CSF3R/S100A9/VCAM1/PDGFD/S100A8/S100A12/CCL21/C3AR1/C5/KIT/SAA1/CXCR2/CXCR1/HGF/CXCL12/RIPOR2/CCR1/CXCL9/CCL5/EDN1 | 20 |
| BP | GO:0030595 | leukocyte chemotaxis | 17/240 | 5.12E-09 | 1.53E-06 | 1.15E-06 | CSF3R/S100A9/S100A8/S100A12/CCL21/C3AR1/C5/KIT/SAA1/CXCR2/CXCR1/CXCL12/RIPOR2/CCR1/CXCL9/CCL5/EDN1 | 17 |
| BP | GO:1990266 | neutrophil migration | 13/240 | 5.90E-09 | 1.62E-06 | 1.21E-06 | CSF3R/S100A9/S100A8/S100A12/CCL21/C3AR1/SAA1/CXCR2/CXCR1/RIPOR2/CXCL9/CCL5/EDN1 | 13 |
| BP | GO:0071621 | granulocyte chemotaxis | 13/240 | 6.51E-09 | 1.65E-06 | 1.24E-06 | CSF3R/S100A9/S100A8/S100A12/CCL21/C3AR1/SAA1/CXCR2/CXCR1/RIPOR2/CXCL9/CCL5/EDN1 | 13 |
| BP | GO:0002691 | regulation of cellular extravasation | 8/240 | 8.34E-09 | 1.90E-06 | 1.43E-06 | PDGFD/PLCB1/CCL21/CXCL12/SELP/THY1/RIPOR2/SELE | 8 |
| BP | GO:0002685 | regulation of leukocyte migration | 16/240 | 8.66E-09 | 1.90E-06 | 1.43E-06 | PDGFD/PLCB1/CCL21/C3AR1/C5/ITGA2/CXCL12/SELP/THY1/BMP5/RIPOR2/CCR1/HMOX1/CCL5/SELE/EDN1 | 16 |
| BP | GO:0050727 | regulation of inflammatory response | 21/240 | 1.01E-08 | 2.07E-06 | 1.55E-06 | NT5E/S100A9/RORA/PDCD4/S100A8/SIGLEC10/S100A12/IGF1/ACE2/SAA1/NR1D2/HGF/ITGA2/FABP4/HCK/BIRC3/CCL5/TNFAIP3/PTGS2/SELE/IDO1 | 21 |
| BP | GO:0097530 | granulocyte migration | 13/240 | 5.53E-08 | 1.07E-05 | 8.04E-06 | CSF3R/S100A9/S100A8/S100A12/CCL21/C3AR1/SAA1/CXCR2/CXCR1/RIPOR2/CXCL9/CCL5/EDN1 | 13 |
| BP | GO:0018108 | peptidyl-tyrosine phosphorylation | 20/240 | 5.87E-08 | 1.07E-05 | 8.06E-06 | ADRA1A/FGR/EPHA3/SFRP2/CNTN1/PDGFD/ENPP2/BMP6/IGF1/KIT/WEE1/FGFR2/EPHA4/ITK/HGF/THY1/HCK/AREG/ITGB3/CCL5 | 20 |
| BP | GO:0018212 | peptidyl-tyrosine modification | 20/240 | 6.70E-08 | 1.16E-05 | 8.72E-06 | ADRA1A/FGR/EPHA3/SFRP2/CNTN1/PDGFD/ENPP2/BMP6/IGF1/KIT/WEE1/FGFR2/EPHA4/ITK/HGF/THY1/HCK/AREG/ITGB3/CCL5 | 20 |
| BP | GO:0002544 | chronic inflammatory response | 6/240 | 9.42E-08 | 1.55E-05 | 1.16E-05 | S100A9/VCAM1/S100A8/CCL5/TNFAIP3/IDO1 | 6 |
| BP | GO:0032102 | negative regulation of response to external stimulus | 20/240 | 1.70E-07 | 2.67E-05 | 2.00E-05 | NT5E/RORA/SIGLEC10/IGF1/C5/SAA1/SEMA3D/EPHA4/NR1D2/HGF/F11/CR1/VSIG4/ANGPT2/SERPINE2/ROBO2/TNFAIP3/FAP/CPB2/EDN1 | 20 |
| BP | GO:0031589 | cell-substrate adhesion | 19/240 | 1.84E-07 | 2.76E-05 | 2.07E-05 | ECM2/POSTN/EPHA3/ANTXR1/FZD7/FREM1/VCAM1/CCL21/ITGB6/CCDC80/ITGA2/THY1/FAM107A/ADAMTS9/ANGPT2/HAS2/ITGB3/OLFM4/ITGBL1 | 19 |
| BP | GO:0032496 | response to lipopolysaccharide | 18/240 | 2.10E-07 | 3.01E-05 | 2.26E-05 | S100A9/PDCD4/VCAM1/S100A8/BMP6/CD14/FGFR2/LILRB2/SELP/HCK/TIMP4/CXCL9/CCL5/TNFAIP3/SELE/EDN1/ANKRD1/IDO1 | 18 |
| BP | GO:0007159 | leukocyte cell-cell adhesion | 19/240 | 2.48E-07 | 3.28E-05 | 2.46E-05 | NT5E/S100A9/VCAM1/S100A8/CCL21/IGF1/LILRB2/CXCL12/SELP/THY1/CR1/RIPOR2/VSIG4/LRRC32/HAS2/OLR1/CCL5/SELE/IDO1 | 19 |
| BP | GO:0045123 | cellular extravasation | 9/240 | 2.49E-07 | 3.28E-05 | 2.46E-05 | VCAM1/PDGFD/PLCB1/CCL21/CXCL12/SELP/THY1/RIPOR2/SELE | 9 |
| BP | GO:0008217 | regulation of blood pressure | 13/240 | 4.48E-07 | 5.68E-05 | 4.26E-05 | HBB/PDE4D/POSTN/ADRA1A/ATP2B1/ACE2/CPA3/MME/ANPEP/HMOX1/ERAP2/PTGS2/EDN1 | 13 |
| BP | GO:0002237 | response to molecule of bacterial origin | 18/240 | 5.03E-07 | 6.14E-05 | 4.61E-05 | S100A9/PDCD4/VCAM1/S100A8/BMP6/CD14/FGFR2/LILRB2/SELP/HCK/TIMP4/CXCL9/CCL5/TNFAIP3/SELE/EDN1/ANKRD1/IDO1 | 18 |
| BP | GO:0071674 | mononuclear cell migration | 13/240 | 1.00E-06 | 0.000118229 | 8.86E-05 | PDGFD/PLCB1/S100A12/CCL21/C3AR1/SAA1/CXCR2/CXCR1/CXCL12/BMP5/RIPOR2/CCR1/CCL5 | 13 |
| BP | GO:0002687 | positive regulation of leukocyte migration | 11/240 | 1.09E-06 | 0.000123376 | 9.25E-05 | PDGFD/CCL21/C3AR1/ITGA2/CXCL12/SELP/THY1/RIPOR2/CCR1/CCL5/EDN1 | 11 |
| BP | GO:0002686 | negative regulation of leukocyte migration | 7/240 | 1.74E-06 | 0.000190594 | 0.000142904 | PLCB1/CCL21/C5/CXCL12/BMP5/RIPOR2/HMOX1 | 7 |
| BP | GO:0006957 | complement activation, alternative pathway | 5/240 | 2.39E-06 | 0.000254486 | 0.000190808 | CFH/C5/C7/CR1/VSIG4 | 5 |
| BP | GO:0050673 | epithelial cell proliferation | 19/240 | 2.59E-06 | 0.000266536 | 0.000199843 | KRT4/FZD7/SULF1/SFRP2/PROK2/BMP6/IGF1/KIT/FGFR2/HGF/CXCL12/BMP5/HAS2/AREG/HMOX1/ITGB3/TNFAIP3/FAP/CPB2 | 19 |
| BP | GO:0001822 | kidney development | 15/240 | 3.22E-06 | 0.000321381 | 0.000240965 | BICC1/AHI1/SULF1/ARID5B/PDGFD/BMP6/FGFR2/MME/EPHA4/CA2/ANGPT2/HAS2/EGR1/FRAS1/ROBO2 | 15 |
| BP | GO:0001819 | positive regulation of cytokine production | 19/240 | 3.50E-06 | 0.000339444 | 0.000254508 | PDE4D/POSTN/FGR/RORA/RASGRP1/LUM/SULF1/PLCB1/CD14/C3AR1/C5/SAA1/GBP5/LILRB2/HGF/EGR1/HMOX1/PTGS2/IDO1 | 19 |
| BP | GO:1990868 | response to chemokine | 9/240 | 4.07E-06 | 0.000372108 | 0.000278999 | CCL21/CXCR2/CXCR1/CXCL12/RIPOR2/CCR1/CXCL9/CCL5/EDN1 | 9 |
| BP | GO:1990869 | cellular response to chemokine | 9/240 | 4.07E-06 | 0.000372108 | 0.000278999 | CCL21/CXCR2/CXCR1/CXCL12/RIPOR2/CCR1/CXCL9/CCL5/EDN1 | 9 |
| BP | GO:0002683 | negative regulation of immune system process | 18/240 | 4.40E-06 | 0.00039159 | 0.000293607 | FGR/LILRB3/INHBA/PLCB1/CCL21/C5/LILRB2/CXCL12/THY1/BMP5/CR1/RIPOR2/IL13RA2/VSIG4/LRRC32/HMOX1/TNFAIP3/IDO1 | 18 |
| BP | GO:0072001 | renal system development | 15/240 | 4.54E-06 | 0.000393449 | 0.000295001 | BICC1/AHI1/SULF1/ARID5B/PDGFD/BMP6/FGFR2/MME/EPHA4/CA2/ANGPT2/HAS2/EGR1/FRAS1/ROBO2 | 15 |
| BP | GO:0050920 | regulation of chemotaxis | 13/240 | 6.23E-06 | 0.000525999 | 0.000394384 | PDGFD/CCL21/C3AR1/C5/SEMA3D/ITGA2/CXCL12/RIPOR2/CCR1/ANGPT2/ROBO2/CCL5/EDN1 | 13 |
| BP | GO:0048660 | regulation of smooth muscle cell proliferation | 11/240 | 6.61E-06 | 0.000544215 | 0.000408041 | PDE1A/PDCD4/OGN/PDGFD/IGF1/FGFR2/ITGA2/HMOX1/CCL5/TNFAIP3/EDN1 | 11 |
| BP | GO:0050730 | regulation of peptidyl-tyrosine phosphorylation | 14/240 | 7.08E-06 | 0.000569281 | 0.000426836 | ADRA1A/SFRP2/CNTN1/PDGFD/ENPP2/BMP6/IGF1/KIT/EPHA4/HGF/THY1/AREG/ITGB3/CCL5 | 14 |
| BP | GO:0048659 | smooth muscle cell proliferation | 11/240 | 7.44E-06 | 0.000583696 | 0.000437644 | PDE1A/PDCD4/OGN/PDGFD/IGF1/FGFR2/ITGA2/HMOX1/CCL5/TNFAIP3/EDN1 | 11 |
| BP | GO:0006959 | humoral immune response | 17/240 | 7.99E-06 | 0.000612241 | 0.000459046 | S100A9/CFH/LCN2/S100A8/S100A12/C3AR1/C5/BPIFA1/BPIFB1/C7/FCN3/CR1/VSIG4/MFAP4/COLEC10/CXCL9/CPB2 | 17 |
| BP | GO:0007160 | cell-matrix adhesion | 13/240 | 8.29E-06 | 0.000619479 | 0.000464473 | ECM2/POSTN/EPHA3/FREM1/VCAM1/CCL21/ITGB6/ITGA2/THY1/FAM107A/ADAMTS9/ITGB3/ITGBL1 | 13 |
| BP | GO:0007229 | integrin-mediated signaling pathway | 9/240 | 8.46E-06 | 0.000619479 | 0.000464473 | FGR/ITGAM/ITGB6/ITGA2/THY1/HCK/ITGAX/ITGB3/ITGBL1 | 9 |
| BP | GO:0045933 | positive regulation of muscle contraction | 6/240 | 9.15E-06 | 0.000655519 | 0.000491495 | ADRA1A/PROK2/ACE2/KIT/ITGA2/EDN1 | 6 |
| BP | GO:0050866 | negative regulation of cell activation | 12/240 | 1.02E-05 | 0.000712364 | 0.000534117 | FGR/INHBA/LILRB2/CR1/RIPOR2/IL13RA2/VSIG4/LRRC32/SERPINE2/HMOX1/TNFAIP3/IDO1 | 12 |
| BP | GO:0002407 | dendritic cell chemotaxis | 5/240 | 1.12E-05 | 0.000766022 | 0.000574348 | CCL21/CXCR2/CXCR1/CCR1/CCL5 | 5 |
| BP | GO:0071675 | regulation of mononuclear cell migration | 9/240 | 1.23E-05 | 0.000828537 | 0.000621221 | PDGFD/PLCB1/CCL21/C3AR1/CXCL12/BMP5/RIPOR2/CCR1/CCL5 | 9 |
| BP | GO:0072503 | cellular divalent inorganic cation homeostasis | 19/240 | 1.33E-05 | 0.000876738 | 0.000657361 | PDE4D/ADRA1A/S100A9/ATP2B1/S100A8/PROK2/CCL21/C3AR1/SAA1/CXCR2/TRPC6/CXCR1/FPR1/CXCL12/THY1/CCR1/CXCL9/CCL5/EDN1 | 19 |
| BP | GO:0001655 | urogenital system development | 15/240 | 1.60E-05 | 0.001031866 | 0.000773673 | BICC1/AHI1/SULF1/ARID5B/PDGFD/BMP6/FGFR2/MME/EPHA4/CA2/ANGPT2/HAS2/EGR1/FRAS1/ROBO2 | 15 |
| BP | GO:0070098 | chemokine-mediated signaling pathway | 8/240 | 1.64E-05 | 0.001036836 | 0.000777399 | CCL21/CXCR2/CXCR1/CXCL12/CCR1/CXCL9/CCL5/EDN1 | 8 |
| BP | GO:1903236 | regulation of leukocyte tethering or rolling | 4/240 | 1.67E-05 | 0.001038336 | 0.000778524 | CCL21/CXCL12/SELP/SELE | 4 |
| BP | GO:0045987 | positive regulation of smooth muscle contraction | 5/240 | 2.05E-05 | 0.001253613 | 0.000939935 | ADRA1A/PROK2/KIT/ITGA2/EDN1 | 5 |
| BP | GO:0014911 | positive regulation of smooth muscle cell migration | 6/240 | 2.14E-05 | 0.001284184 | 0.000962856 | POSTN/PDGFD/IGF1/ITGA2/HAS2/CCL5 | 6 |
| BP | GO:0002695 | negative regulation of leukocyte activation | 11/240 | 2.23E-05 | 0.001292811 | 0.000969324 | FGR/INHBA/LILRB2/CR1/RIPOR2/IL13RA2/VSIG4/LRRC32/HMOX1/TNFAIP3/IDO1 | 11 |
| BP | GO:0072507 | divalent inorganic cation homeostasis | 19/240 | 2.28E-05 | 0.001292811 | 0.000969324 | PDE4D/ADRA1A/S100A9/ATP2B1/S100A8/PROK2/CCL21/C3AR1/SAA1/CXCR2/TRPC6/CXCR1/FPR1/CXCL12/THY1/CCR1/CXCL9/CCL5/EDN1 | 19 |
| BP | GO:1903037 | regulation of leukocyte cell-cell adhesion | 15/240 | 2.29E-05 | 0.001292811 | 0.000969324 | VCAM1/CCL21/IGF1/LILRB2/CXCL12/SELP/THY1/CR1/RIPOR2/VSIG4/LRRC32/HAS2/CCL5/SELE/IDO1 | 15 |
| BP | GO:0009214 | cyclic nucleotide catabolic process | 4/240 | 2.31E-05 | 0.001292811 | 0.000969324 | PDE4D/PDE7B/PDE1A/PDE8B | 4 |
| BP | GO:0003044 | regulation of systemic arterial blood pressure mediated by a chemical signal | 6/240 | 2.44E-05 | 0.001337058 | 0.0010025 | PDE4D/ADRA1A/ACE2/CPA3/MME/EDN1 | 6 |
| BP | GO:0010575 | positive regulation of vascular endothelial growth factor production | 5/240 | 2.48E-05 | 0.001337058 | 0.0010025 | RORA/SULF1/C3AR1/C5/PTGS2 | 5 |
| BP | GO:0007162 | negative regulation of cell adhesion | 14/240 | 2.68E-05 | 0.001424535 | 0.001068089 | POSTN/FZD7/BMP6/CCL21/LILRB2/CXCL12/CR1/RIPOR2/VSIG4/FAM107A/ANGPT2/LRRC32/SERPINE2/IDO1 | 14 |
| BP | GO:0040013 | negative regulation of locomotion | 16/240 | 2.82E-05 | 0.001475842 | 0.001106557 | SULF1/SFRP2/DPYSL3/PLCB1/ADORA3/CCL21/C5/SEMA3D/CXCL12/THY1/BMP5/RIPOR2/ADAMTS9/ANGPT2/HMOX1/ROBO2 | 16 |
| BP | GO:0033002 | muscle cell proliferation | 12/240 | 2.88E-05 | 0.00148117 | 0.001110552 | PDE1A/PDCD4/ZFPM2/OGN/PDGFD/IGF1/FGFR2/ITGA2/HMOX1/CCL5/TNFAIP3/EDN1 | 12 |
| BP | GO:0036336 | dendritic cell migration | 5/240 | 2.96E-05 | 0.001500538 | 0.001125074 | CCL21/CXCR2/CXCR1/CCR1/CCL5 | 5 |
| BP | GO:0045785 | positive regulation of cell adhesion | 17/240 | 3.35E-05 | 0.00166783 | 0.001250506 | ECM2/VCAM1/SFRP2/CCL21/IGF1/CCDC80/SAA1/LILRB2/ITGA2/CXCL12/SELP/THY1/CR1/HAS2/CCL5/OLFM4/SELE | 17 |
| BP | GO:0050901 | leukocyte tethering or rolling | 5/240 | 3.52E-05 | 0.00166783 | 0.001250506 | VCAM1/CCL21/CXCL12/SELP/SELE | 5 |
| BP | GO:0045661 | regulation of myoblast differentiation | 6/240 | 3.53E-05 | 0.00166783 | 0.001250506 | TBX3/PLCB1/CDON/SOSTDC1/RIPOR2/CXCL9 | 6 |
| BP | GO:0033627 | cell adhesion mediated by integrin | 7/240 | 3.61E-05 | 0.00166783 | 0.001250506 | SFRP2/CCL21/ITGB6/ITGA2/ITGB3/CCL5/ITGBL1 | 7 |
| BP | GO:0031099 | regeneration | 11/240 | 3.65E-05 | 0.00166783 | 0.001250506 | POSTN/FZD7/IGF1/EPHA4/HGF/CXCL12/THY1/ANGPT2/HMOX1/PRRX1/CPB2 | 11 |
| BP | GO:0048015 | phosphatidylinositol-mediated signaling | 11/240 | 3.65E-05 | 0.00166783 | 0.001250506 | FGR/PLCB4/PDGFD/PLCB1/IGF1/KIT/HGF/SELP/SERPINE2/CCL5/EDN1 | 11 |
| BP | GO:0031346 | positive regulation of cell projection organization | 15/240 | 3.70E-05 | 0.00166783 | 0.001250506 | S100A9/EPHA3/CNTN1/DPYSL3/ENPP2/CCL21/KIT/EPHA4/HGF/ITGA2/CXCL12/BMP5/RIPOR2/ROBO2/ANKRD1 | 15 |
| BP | GO:0051480 | regulation of cytosolic calcium ion concentration | 15/240 | 3.70E-05 | 0.00166783 | 0.001250506 | PDE4D/ADRA1A/ATP2B1/PROK2/CCL21/C3AR1/SAA1/CXCR2/TRPC6/CXCR1/FPR1/THY1/CCR1/CXCL9/EDN1 | 15 |
| BP | GO:0050731 | positive regulation of peptidyl-tyrosine phosphorylation | 11/240 | 3.83E-05 | 0.001704653 | 0.001278115 | ADRA1A/CNTN1/ENPP2/BMP6/IGF1/KIT/EPHA4/HGF/AREG/ITGB3/CCL5 | 11 |
| BP | GO:0043406 | positive regulation of MAP kinase activity | 12/240 | 4.07E-05 | 0.00177859 | 0.001333552 | RASGRP1/PDGFD/PROK2/S100A12/IGF1/C5/KIT/SAA1/EPHA4/HGF/FPR1/EDN1 | 12 |
| BP | GO:0043405 | regulation of MAP kinase activity | 14/240 | 4.15E-05 | 0.00177859 | 0.001333552 | PDCD4/RASGRP1/SFRP2/PDGFD/PROK2/S100A12/IGF1/C5/KIT/SAA1/EPHA4/HGF/FPR1/EDN1 | 14 |
| BP | GO:0006939 | smooth muscle contraction | 8/240 | 4.16E-05 | 0.00177859 | 0.001333552 | ADRA1A/ATP2B1/TBX3/SULF1/PROK2/KIT/ITGA2/EDN1 | 8 |
| BP | GO:0048017 | inositol lipid-mediated signaling | 11/240 | 4.41E-05 | 0.001861881 | 0.001396002 | FGR/PLCB4/PDGFD/PLCB1/IGF1/KIT/HGF/SELP/SERPINE2/CCL5/EDN1 | 11 |
| BP | GO:0007204 | positive regulation of cytosolic calcium ion concentration | 14/240 | 4.61E-05 | 0.001921505 | 0.001440707 | PDE4D/ADRA1A/PROK2/CCL21/C3AR1/SAA1/CXCR2/TRPC6/CXCR1/FPR1/THY1/CCR1/CXCL9/EDN1 | 14 |
| BP | GO:0022407 | regulation of cell-cell adhesion | 17/240 | 4.75E-05 | 0.001955199 | 0.00146597 | VCAM1/BMP6/CCL21/IGF1/LILRB2/CXCL12/SELP/THY1/CR1/RIPOR2/VSIG4/LRRC32/HAS2/SERPINE2/CCL5/SELE/IDO1 | 17 |
| BP | GO:0048333 | mesodermal cell differentiation | 5/240 | 4.87E-05 | 0.001955199 | 0.00146597 | SFRP2/INHBA/FGFR2/ITGA2/ITGB3 | 5 |
| BP | GO:0050974 | detection of mechanical stimulus involved in sensory perception | 5/240 | 4.87E-05 | 0.001955199 | 0.00146597 | PIEZO2/KIT/ITGA2/CXCL12/SERPINE2 | 5 |
| BP | GO:0001501 | skeletal system development | 18/240 | 5.37E-05 | 0.002131583 | 0.001598219 | FGR/FREM1/TBX3/LUM/SULF1/ARID5B/SFRP2/BMP6/IGF1/ITGB6/KIT/FGFR2/BMP5/HAS2/TLL1/SFRP4/PRRX1/EDN1 | 18 |
| BP | GO:0006874 | cellular calcium ion homeostasis | 17/240 | 5.47E-05 | 0.002143906 | 0.001607459 | PDE4D/ADRA1A/ATP2B1/PROK2/CCL21/C3AR1/SAA1/CXCR2/TRPC6/CXCR1/FPR1/CXCL12/THY1/CCR1/CXCL9/CCL5/EDN1 | 17 |
| BP | GO:0009612 | response to mechanical stimulus | 11/240 | 5.80E-05 | 0.002246415 | 0.001684318 | POSTN/PIEZO2/BMP6/KIT/ITGA2/CXCL12/RIPOR2/ANGPT2/SERPINE2/EDN1/ANKRD1 | 11 |
| BP | GO:0050679 | positive regulation of epithelial cell proliferation | 11/240 | 6.06E-05 | 0.002296782 | 0.001722082 | FZD7/BMP6/IGF1/FGFR2/CXCL12/BMP5/HAS2/AREG/HMOX1/ITGB3/TNFAIP3 | 11 |
| BP | GO:0050886 | endocrine process | 7/240 | 6.06E-05 | 0.002296782 | 0.001722082 | TBX3/INHBA/BMP6/ACE2/CPA3/MME/EDN1 | 7 |
| BP | GO:0045445 | myoblast differentiation | 7/240 | 7.14E-05 | 0.002672207 | 0.002003569 | TBX3/PLCB1/CDON/IGF1/SOSTDC1/RIPOR2/CXCL9 | 7 |
| BP | GO:0050921 | positive regulation of chemotaxis | 9/240 | 7.35E-05 | 0.002719361 | 0.002038923 | PDGFD/CCL21/C3AR1/ITGA2/CXCL12/RIPOR2/CCR1/CCL5/EDN1 | 9 |
| BP | GO:0055074 | calcium ion homeostasis | 17/240 | 7.59E-05 | 0.002753256 | 0.002064337 | PDE4D/ADRA1A/ATP2B1/PROK2/CCL21/C3AR1/SAA1/CXCR2/TRPC6/CXCR1/FPR1/CXCL12/THY1/CCR1/CXCL9/CCL5/EDN1 | 17 |
| BP | GO:0009154 | purine ribonucleotide catabolic process | 5/240 | 7.60E-05 | 0.002753256 | 0.002064337 | PDE4D/PDE7B/NT5E/PDE1A/PDE8B | 5 |
| BP | GO:0052547 | regulation of peptidase activity | 17/240 | 7.80E-05 | 0.002793076 | 0.002094194 | S100A9/ANTXR1/SFRP2/UACA/S100A8/PI15/C5/EPHA4/OVOS2/HGF/CR1/BIRC3/TFPI2/SERPINE2/TIMP4/COL6A3/SERPINA3 | 17 |
| BP | GO:0045663 | positive regulation of myoblast differentiation | 4/240 | 8.53E-05 | 0.003020682 | 0.002264848 | PLCB1/CDON/RIPOR2/CXCL9 | 4 |
| BP | GO:0030336 | negative regulation of cell migration | 14/240 | 9.00E-05 | 0.003154247 | 0.002364993 | SULF1/SFRP2/DPYSL3/PLCB1/ADORA3/CCL21/C5/CXCL12/THY1/BMP5/RIPOR2/ADAMTS9/ANGPT2/HMOX1 | 14 |
| BP | GO:0050678 | regulation of epithelial cell proliferation | 15/240 | 9.48E-05 | 0.003289307 | 0.002466258 | KRT4/FZD7/SULF1/SFRP2/BMP6/IGF1/FGFR2/CXCL12/BMP5/HAS2/AREG/HMOX1/ITGB3/TNFAIP3/CPB2 | 15 |
| BP | GO:0006956 | complement activation | 10/240 | 9.71E-05 | 0.003334011 | 0.002499776 | CFH/C3AR1/C5/C7/FCN3/CR1/VSIG4/MFAP4/COLEC10/CPB2 | 10 |
| BP | GO:0015711 | organic anion transport | 15/240 | 0.000100608 | 0.003417574 | 0.00256243 | HBB/HBA2/SLC7A7/SLC36A1/ABCG2/CA4/SLCO4A1/ACE2/SLC7A2/CA2/CA12/CR1/AQP9/SLCO1A2/EDN1 | 15 |
| BP | GO:0006940 | regulation of smooth muscle contraction | 6/240 | 0.000102223 | 0.003436991 | 0.002576988 | ADRA1A/ATP2B1/PROK2/KIT/ITGA2/EDN1 | 6 |
| BP | GO:0010518 | positive regulation of phospholipase activity | 6/240 | 0.000112385 | 0.003728903 | 0.002795859 | ADRA1A/KIT/FGFR2/ITK/CCL5/SELE | 6 |
| BP | GO:0060021 | roof of mouth development | 7/240 | 0.000113169 | 0.003728903 | 0.002795859 | TBX3/ARID5B/INHBA/ITGB6/LRRC32/FRAS1/PRRX1 | 7 |
| BP | GO:0010810 | regulation of cell-substrate adhesion | 11/240 | 0.000114571 | 0.003737727 | 0.002802475 | ECM2/POSTN/EPHA3/FZD7/CCL21/CCDC80/THY1/FAM107A/ANGPT2/HAS2/OLFM4 | 11 |
| BP | GO:0042110 | T cell activation | 17/240 | 0.0001278 | 0.004128441 | 0.003095424 | RORA/FZD7/RASGRP1/VCAM1/CCL21/IGF1/KIT/ITK/LILRB2/THY1/CR1/RIPOR2/VSIG4/LRRC32/EGR1/CCL5/IDO1 | 17 |
| BP | GO:0014909 | smooth muscle cell migration | 7/240 | 0.000130863 | 0.004186342 | 0.003138837 | POSTN/PDGFD/IGF1/ITGA2/HAS2/ITGB3/CCL5 | 7 |
| BP | GO:0002688 | regulation of leukocyte chemotaxis | 8/240 | 0.00014245 | 0.004395425 | 0.003295603 | CCL21/C3AR1/C5/CXCL12/RIPOR2/CCR1/CCL5/EDN1 | 8 |
| BP | GO:2000146 | negative regulation of cell motility | 14/240 | 0.000143495 | 0.004395425 | 0.003295603 | SULF1/SFRP2/DPYSL3/PLCB1/ADORA3/CCL21/C5/CXCL12/THY1/BMP5/RIPOR2/ADAMTS9/ANGPT2/HMOX1 | 14 |
| BP | GO:0006730 | one-carbon metabolic process | 5/240 | 0.000146351 | 0.004395425 | 0.003295603 | AHCYL2/CA4/SHMT2/CA2/CA12 | 5 |
| BP | GO:0009187 | cyclic nucleotide metabolic process | 5/240 | 0.000146351 | 0.004395425 | 0.003295603 | PDE4D/PDE7B/RORA/PDE1A/PDE8B | 5 |
| BP | GO:0009261 | ribonucleotide catabolic process | 5/240 | 0.000146351 | 0.004395425 | 0.003295603 | PDE4D/PDE7B/NT5E/PDE1A/PDE8B | 5 |
| BP | GO:0071634 | regulation of transforming growth factor beta production | 5/240 | 0.000146351 | 0.004395425 | 0.003295603 | LTBP1/LUM/ITGB6/LRRC32/PTGS2 | 5 |
| BP | GO:0045861 | negative regulation of proteolysis | 14/240 | 0.000147881 | 0.004395425 | 0.003295603 | SFRP2/PI15/C5/EPHA4/OVOS2/IL1R2/HGF/CR1/TFPI2/SERPINE2/TIMP4/COL6A3/SERPINA3/CPB2 | 14 |
| BP | GO:0003073 | regulation of systemic arterial blood pressure | 7/240 | 0.000150738 | 0.004395425 | 0.003295603 | PDE4D/POSTN/ADRA1A/ACE2/CPA3/MME/EDN1 | 7 |
| BP | GO:0014068 | positive regulation of phosphatidylinositol 3-kinase signaling | 7/240 | 0.000150738 | 0.004395425 | 0.003295603 | FGR/PDGFD/IGF1/KIT/HGF/SELP/CCL5 | 7 |
| BP | GO:1903035 | negative regulation of response to wounding | 7/240 | 0.000150738 | 0.004395425 | 0.003295603 | SIGLEC10/EPHA4/F11/SERPINE2/FAP/CPB2/EDN1 | 7 |
| BP | GO:0051250 | negative regulation of lymphocyte activation | 9/240 | 0.000153153 | 0.004426647 | 0.003319013 | FGR/INHBA/LILRB2/CR1/RIPOR2/VSIG4/LRRC32/TNFAIP3/IDO1 | 9 |
| BP | GO:0001666 | response to hypoxia | 14/240 | 0.000157002 | 0.00447331 | 0.003354 | POSTN/RORA/VCAM1/ALAS2/TRPC6/ITGA2/CXCL12/HIF3A/ANGPT2/EGR1/HMOX1/PTGS2/EDN1/ANKRD1 | 14 |
| BP | GO:0022408 | negative regulation of cell-cell adhesion | 10/240 | 0.000159113 | 0.00447331 | 0.003354 | BMP6/CCL21/LILRB2/CXCL12/CR1/RIPOR2/VSIG4/LRRC32/SERPINE2/IDO1 | 10 |
| BP | GO:0014065 | phosphatidylinositol 3-kinase signaling | 9/240 | 0.000160886 | 0.00447331 | 0.003354 | FGR/PDGFD/IGF1/KIT/HGF/SELP/SERPINE2/CCL5/EDN1 | 9 |
| BP | GO:0030509 | BMP signaling pathway | 9/240 | 0.000160886 | 0.00447331 | 0.003354 | HIVEP1/PDCD4/SULF1/SFRP2/BMP6/SOSTDC1/BMP5/EGR1/SFRP4 | 9 |
| BP | GO:0002690 | positive regulation of leukocyte chemotaxis | 7/240 | 0.000161555 | 0.00447331 | 0.003354 | CCL21/C3AR1/CXCL12/RIPOR2/CCR1/CCL5/EDN1 | 7 |
| BP | GO:0019932 | second-messenger-mediated signaling | 13/240 | 0.000163255 | 0.004482713 | 0.00336105 | PDE3A/PDE4D/PDE7B/VCAM1/IGF1/CXCR2/CXCR1/FPR1/SELP/TRAT1/CCR1/SELE/EDN1 | 13 |
| BP | GO:1902042 | negative regulation of extrinsic apoptotic signaling pathway via death domain receptors | 5/240 | 0.000164963 | 0.004492179 | 0.003368148 | SFRP2/HGF/BMP5/HMOX1/TNFAIP3 | 5 |
| BP | GO:0014706 | striated muscle tissue development | 14/240 | 0.000171596 | 0.004634491 | 0.003474851 | ADRA1A/FZD7/TBX3/ZFPM2/CDON/IGF1/FGFR2/NR1D2/BMP5/RIPOR2/ADAMTS9/EGR1/EDN1/ANKRD1 | 14 |
| BP | GO:0051271 | negative regulation of cellular component movement | 14/240 | 0.000176714 | 0.004721364 | 0.003539986 | SULF1/SFRP2/DPYSL3/PLCB1/ADORA3/CCL21/C5/CXCL12/THY1/BMP5/RIPOR2/ADAMTS9/ANGPT2/HMOX1 | 14 |
| BP | GO:0007498 | mesoderm development | 8/240 | 0.000179111 | 0.004721364 | 0.003539986 | TBX3/SFRP2/INHBA/ZFPM2/FGFR2/ITGA2/HCK/ITGB3 | 8 |
| BP | GO:0042476 | odontogenesis | 8/240 | 0.000179111 | 0.004721364 | 0.003539986 | CSF3R/INHBA/ASPN/ITGB6/FGFR2/SOSTDC1/CA2/EDN1 | 8 |
| BP | GO:0071604 | transforming growth factor beta production | 5/240 | 0.00018533 | 0.004846514 | 0.003633821 | LTBP1/LUM/ITGB6/LRRC32/PTGS2 | 5 |
| BP | GO:0060571 | morphogenesis of an epithelial fold | 4/240 | 0.000187141 | 0.004855355 | 0.00364045 | SULF1/FGFR2/SOSTDC1/BMP5 | 4 |
| BP | GO:0060348 | bone development | 10/240 | 0.000188677 | 0.00485696 | 0.003641653 | FREM1/SULF1/SFRP2/BMP6/IGF1/ITGB6/KIT/FGFR2/HAS2/SFRP4 | 10 |
| BP | GO:0015701 | bicarbonate transport | 5/240 | 0.000207559 | 0.005272985 | 0.003953581 | HBB/HBA2/CA4/CA2/CA12 | 5 |
| BP | GO:0050918 | positive chemotaxis | 6/240 | 0.000208039 | 0.005272985 | 0.003953581 | HGF/ITGA2/CXCL12/ANGPT2/COLEC10/CCL5 | 6 |
| BP | GO:0007178 | transmembrane receptor protein serine/threonine kinase signaling pathway | 14/240 | 0.000210282 | 0.005289159 | 0.003965707 | LTBP1/HIVEP1/PDCD4/SULF1/SFRP2/INHBA/ASPN/BMP6/ITGB6/SOSTDC1/BMP5/LRRC32/EGR1/SFRP4 | 14 |
| BP | GO:0090023 | positive regulation of neutrophil chemotaxis | 4/240 | 0.000222338 | 0.005488345 | 0.004115053 | CCL21/C3AR1/RIPOR2/EDN1 | 4 |
| BP | GO:0036293 | response to decreased oxygen levels | 14/240 | 0.00022263 | 0.005488345 | 0.004115053 | POSTN/RORA/VCAM1/ALAS2/TRPC6/ITGA2/CXCL12/HIF3A/ANGPT2/EGR1/HMOX1/PTGS2/EDN1/ANKRD1 | 14 |
| BP | GO:0014066 | regulation of phosphatidylinositol 3-kinase signaling | 8/240 | 0.000223198 | 0.005488345 | 0.004115053 | FGR/PDGFD/IGF1/KIT/HGF/SELP/SERPINE2/CCL5 | 8 |
| BP | GO:0048661 | positive regulation of smooth muscle cell proliferation | 7/240 | 0.00022547 | 0.00550314 | 0.004126147 | PDGFD/IGF1/FGFR2/ITGA2/HMOX1/CCL5/EDN1 | 7 |
| BP | GO:1901136 | carbohydrate derivative catabolic process | 10/240 | 0.000232008 | 0.005621073 | 0.00421457 | PDE4D/PDE7B/NT5E/PDE1A/LUM/OGN/CHIT1/PDE8B/ADAMTS9/CHI3L2 | 10 |
| BP | GO:0034612 | response to tumor necrosis factor | 13/240 | 0.000244681 | 0.005884835 | 0.004412334 | POSTN/RORA/VCAM1/CCL21/CD14/FABP4/BIRC3/HAS2/CCL5/TNFAIP3/SELE/EDN1/ANKRD1 | 13 |
| BP | GO:0010517 | regulation of phospholipase activity | 6/240 | 0.000264803 | 0.00627717 | 0.004706499 | ADRA1A/KIT/FGFR2/ITK/CCL5/SELE | 6 |
| BP | GO:0048332 | mesoderm morphogenesis | 6/240 | 0.000264803 | 0.00627717 | 0.004706499 | TBX3/SFRP2/INHBA/FGFR2/ITGA2/ITGB3 | 6 |
| BP | GO:0006195 | purine nucleotide catabolic process | 5/240 | 0.000286548 | 0.006744102 | 0.005056595 | PDE4D/PDE7B/NT5E/PDE1A/PDE8B | 5 |
| BP | GO:0071772 | response to BMP | 9/240 | 0.000294883 | 0.006842532 | 0.005130396 | HIVEP1/PDCD4/SULF1/SFRP2/BMP6/SOSTDC1/BMP5/EGR1/SFRP4 | 9 |
| BP | GO:0071773 | cellular response to BMP stimulus | 9/240 | 0.000294883 | 0.006842532 | 0.005130396 | HIVEP1/PDCD4/SULF1/SFRP2/BMP6/SOSTDC1/BMP5/EGR1/SFRP4 | 9 |
| BP | GO:0060537 | muscle tissue development | 14/240 | 0.000302324 | 0.006966148 | 0.005223081 | ADRA1A/FZD7/TBX3/ZFPM2/CDON/IGF1/FGFR2/NR1D2/BMP5/RIPOR2/ADAMTS9/EGR1/EDN1/ANKRD1 | 14 |
| BP | GO:0050729 | positive regulation of inflammatory response | 8/240 | 0.000305696 | 0.006977197 | 0.005231365 | S100A9/PDCD4/S100A8/S100A12/ITGA2/FABP4/PTGS2/IDO1 | 8 |
| BP | GO:0014812 | muscle cell migration | 7/240 | 0.000308364 | 0.006977197 | 0.005231365 | POSTN/PDGFD/IGF1/ITGA2/HAS2/ITGB3/CCL5 | 7 |
| BP | GO:0006898 | receptor-mediated endocytosis | 13/240 | 0.000310381 | 0.006977197 | 0.005231365 | HBB/HBA2/AHI1/ITGAM/CCL21/CD14/SAA1/CXCR2/CXCR1/SFRP4/ITGB3/SELE/CD163 | 13 |
| BP | GO:0036363 | transforming growth factor beta activation | 3/240 | 0.000311274 | 0.006977197 | 0.005231365 | LTBP1/ITGB6/LRRC32 | 3 |
| BP | GO:0070555 | response to interleukin-1 | 10/240 | 0.000318567 | 0.007092419 | 0.005317756 | RORA/PLCB1/CCL21/IL1R2/HAS2/EGR1/CCL5/SELE/EDN1/ANKRD1 | 10 |
| BP | GO:0060485 | mesenchyme development | 12/240 | 0.000325881 | 0.007206567 | 0.005403342 | EPHA3/PDCD4/S100A4/SFRP2/ZFPM2/FGFR2/SEMA3D/HGF/BMP5/HAS2/ROBO2/EDN1 | 12 |
| BP | GO:0060193 | positive regulation of lipase activity | 6/240 | 0.00033308 | 0.007316661 | 0.005485889 | ADRA1A/KIT/FGFR2/ITK/CCL5/SELE | 6 |
| BP | GO:0042542 | response to hydrogen peroxide | 8/240 | 0.000338168 | 0.007379227 | 0.0055328 | HBB/HBA2/PDGFD/NQO1/TRPC6/HGF/HMOX1/TNFAIP3 | 8 |
| BP | GO:0048738 | cardiac muscle tissue development | 10/240 | 0.000343983 | 0.007456737 | 0.005590915 | ADRA1A/FZD7/TBX3/ZFPM2/IGF1/FGFR2/BMP5/ADAMTS9/EDN1/ANKRD1 | 10 |
| BP | GO:0030195 | negative regulation of blood coagulation | 5/240 | 0.000350633 | 0.007551204 | 0.005661744 | F11/SERPINE2/FAP/CPB2/EDN1 | 5 |
| BP | GO:0071624 | positive regulation of granulocyte chemotaxis | 4/240 | 0.000356384 | 0.007625219 | 0.005717239 | CCL21/C3AR1/RIPOR2/EDN1 | 4 |
| BP | GO:0045765 | regulation of angiogenesis | 13/240 | 0.000379685 | 0.0080089 | 0.006004916 | SULF1/SFRP2/PROK2/ENPP2/C3AR1/C5/HGF/ITGAX/ADAMTS9/ANGPT2/HMOX1/TNFAIP3/THBS2 | 13 |
| BP | GO:0052548 | regulation of endopeptidase activity | 15/240 | 0.000386391 | 0.0080089 | 0.006004916 | S100A9/SFRP2/UACA/S100A8/C5/EPHA4/OVOS2/HGF/CR1/BIRC3/TFPI2/SERPINE2/TIMP4/COL6A3/SERPINA3 | 15 |
| BP | GO:0050982 | detection of mechanical stimulus | 5/240 | 0.000386469 | 0.0080089 | 0.006004916 | PIEZO2/KIT/ITGA2/CXCL12/SERPINE2 | 5 |
| BP | GO:0071622 | regulation of granulocyte chemotaxis | 5/240 | 0.000386469 | 0.0080089 | 0.006004916 | CCL21/C3AR1/RIPOR2/CCL5/EDN1 | 5 |
| BP | GO:1900047 | negative regulation of hemostasis | 5/240 | 0.000386469 | 0.0080089 | 0.006004916 | F11/SERPINE2/FAP/CPB2/EDN1 | 5 |
| BP | GO:1903038 | negative regulation of leukocyte cell-cell adhesion | 8/240 | 0.000392104 | 0.008074886 | 0.006054391 | CCL21/LILRB2/CXCL12/CR1/RIPOR2/VSIG4/LRRC32/IDO1 | 8 |
| BP | GO:0010975 | regulation of neuron projection development | 15/240 | 0.000405806 | 0.008120368 | 0.006088492 | S100A9/EPHA3/SFRP2/CNTN1/DPYSL3/TRPC6/SEMA3D/EPHA4/HGF/CXCL12/THY1/BMP5/PRRX1/ROBO2/ANKRD1 | 15 |
| BP | GO:0001867 | complement activation, lectin pathway | 3/240 | 0.000411139 | 0.008120368 | 0.006088492 | FCN3/MFAP4/COLEC10 | 3 |
| BP | GO:0002002 | regulation of angiotensin levels in blood | 3/240 | 0.000411139 | 0.008120368 | 0.006088492 | ACE2/CPA3/MME | 3 |
| BP | GO:0002003 | angiotensin maturation | 3/240 | 0.000411139 | 0.008120368 | 0.006088492 | ACE2/CPA3/MME | 3 |
| BP | GO:0006563 | L-serine metabolic process | 3/240 | 0.000411139 | 0.008120368 | 0.006088492 | CBS/PHGDH/SHMT2 | 3 |
| BP | GO:0010454 | negative regulation of cell fate commitment | 3/240 | 0.000411139 | 0.008120368 | 0.006088492 | FZD7/SFRP2/SOSTDC1 | 3 |
| BP | GO:0055076 | transition metal ion homeostasis | 8/240 | 0.000411563 | 0.008120368 | 0.006088492 | S100A9/LCN2/S100A8/ABCG2/BMP6/ALAS2/STEAP2/HMOX1 | 8 |
| BP | GO:0070482 | response to oxygen levels | 14/240 | 0.000438192 | 0.008594311 | 0.006443845 | POSTN/RORA/VCAM1/ALAS2/TRPC6/ITGA2/CXCL12/HIF3A/ANGPT2/EGR1/HMOX1/PTGS2/EDN1/ANKRD1 | 14 |
| BP | GO:1901342 | regulation of vasculature development | 13/240 | 0.000449177 | 0.008757616 | 0.006566289 | SULF1/SFRP2/PROK2/ENPP2/C3AR1/C5/HGF/ITGAX/ADAMTS9/ANGPT2/HMOX1/TNFAIP3/THBS2 | 13 |
| BP | GO:0007596 | blood coagulation | 13/240 | 0.000461749 | 0.008949778 | 0.006710368 | HBB/ZFPM2/SAA1/TRPC6/ITGA2/SELP/F11/TFPI2/SERPINE2/ITGB3/FAP/CPB2/EDN1 | 13 |
| BP | GO:0072523 | purine-containing compound catabolic process | 5/240 | 0.000466365 | 0.00895636 | 0.006715303 | PDE4D/PDE7B/NT5E/PDE1A/PDE8B | 5 |
| BP | GO:1902624 | positive regulation of neutrophil migration | 4/240 | 0.000472784 | 0.00895636 | 0.006715303 | CCL21/C3AR1/RIPOR2/EDN1 | 4 |
| BP | GO:0010466 | negative regulation of peptidase activity | 11/240 | 0.000472961 | 0.00895636 | 0.006715303 | SFRP2/PI15/C5/OVOS2/HGF/CR1/TFPI2/SERPINE2/TIMP4/COL6A3/SERPINA3 | 11 |
| BP | GO:0071559 | response to transforming growth factor beta | 11/240 | 0.000472961 | 0.00895636 | 0.006715303 | LTBP1/PDE3A/POSTN/PDGFD/ASPN/MXRA5/ITGB6/FGFR2/LRRC32/EDN1/ANKRD1 | 11 |
| BP | GO:0071347 | cellular response to interleukin-1 | 9/240 | 0.000510395 | 0.009560871 | 0.007168554 | RORA/PLCB1/CCL21/IL1R2/HAS2/EGR1/CCL5/EDN1/ANKRD1 | 9 |
| BP | GO:0050819 | negative regulation of coagulation | 5/240 | 0.000510687 | 0.009560871 | 0.007168554 | F11/SERPINE2/FAP/CPB2/EDN1 | 5 |
| BP | GO:0007599 | hemostasis | 13/240 | 0.000515069 | 0.009588424 | 0.007189212 | HBB/ZFPM2/SAA1/TRPC6/ITGA2/SELP/F11/TFPI2/SERPINE2/ITGB3/FAP/CPB2/EDN1 | 13 |
| BP | GO:0050817 | coagulation | 13/240 | 0.000529185 | 0.009641922 | 0.007229324 | HBB/ZFPM2/SAA1/TRPC6/ITGA2/SELP/F11/TFPI2/SERPINE2/ITGB3/FAP/CPB2/EDN1 | 13 |
| BP | GO:0002430 | complement receptor mediated signaling pathway | 3/240 | 0.00052947 | 0.009641922 | 0.007229324 | C3AR1/FPR1/CR1 | 3 |
| BP | GO:0070486 | leukocyte aggregation | 3/240 | 0.00052947 | 0.009641922 | 0.007229324 | S100A9/S100A8/HAS2 | 3 |
| BP | GO:0071496 | cellular response to external stimulus | 12/240 | 0.000529647 | 0.009641922 | 0.007229324 | POSTN/ATP2B1/VCAM1/SFRP2/CBS/PIEZO2/BMP6/ITGA2/RIPOR2/FAM107A/HMOX1/ANKRD1 | 12 |
| BP | GO:0010660 | regulation of muscle cell apoptotic process | 6/240 | 0.000546127 | 0.009849848 | 0.007385223 | PDE1A/PDCD4/SFRP2/IGF1/HMOX1/EDN1 | 6 |
| BP | GO:0022612 | gland morphogenesis | 7/240 | 0.000547048 | 0.009849848 | 0.007385223 | TBX3/SULF1/FGFR2/HGF/SOSTDC1/TNFAIP3/CPB2 | 7 |
| BP | GO:0045444 | fat cell differentiation | 10/240 | 0.00055384 | 0.009917958 | 0.00743629 | RORA/ARID5B/SFRP2/ZFPM2/WIF1/PLCB1/PLAC8/FABP4/MEDAG/PTGS2 | 10 |
| BP | GO:0014910 | regulation of smooth muscle cell migration | 6/240 | 0.000583634 | 0.010395002 | 0.007793969 | POSTN/PDGFD/IGF1/ITGA2/HAS2/CCL5 | 6 |
| BP | GO:0071900 | regulation of protein serine/threonine kinase activity | 16/240 | 0.000592217 | 0.010491151 | 0.007866059 | PDCD4/RASGRP1/SFRP2/PDGFD/PROK2/S100A12/IGF1/C5/KIT/SAA1/EPHA4/HGF/FPR1/THY1/TNFAIP3/EDN1 | 16 |
| BP | GO:0006968 | cellular defense response | 5/240 | 0.000608747 | 0.010592003 | 0.007941676 | CXCR2/ITK/LILRB2/TRAT1/CXCL9 | 5 |
| BP | GO:0061756 | leukocyte adhesion to vascular endothelial cell | 5/240 | 0.000608747 | 0.010592003 | 0.007941676 | VCAM1/CCL21/CXCL12/SELP/SELE | 5 |
| BP | GO:0030282 | bone mineralization | 7/240 | 0.000608876 | 0.010592003 | 0.007941676 | FGR/ATP2B1/ASPN/BMP6/IGF1/FGFR2/CCR1 | 7 |
| BP | GO:0034390 | smooth muscle cell apoptotic process | 4/240 | 0.000613983 | 0.010592003 | 0.007941676 | PDE1A/PDCD4/IGF1/EDN1 | 4 |
| BP | GO:0034391 | regulation of smooth muscle cell apoptotic process | 4/240 | 0.000613983 | 0.010592003 | 0.007941676 | PDE1A/PDCD4/IGF1/EDN1 | 4 |
| BP | GO:0071902 | positive regulation of protein serine/threonine kinase activity | 12/240 | 0.00066637 | 0.011396907 | 0.008545177 | RASGRP1/PDGFD/PROK2/S100A12/IGF1/C5/KIT/SAA1/EPHA4/HGF/FPR1/EDN1 | 12 |
| BP | GO:0051917 | regulation of fibrinolysis | 3/240 | 0.000667558 | 0.011396907 | 0.008545177 | F11/FAP/CPB2 | 3 |
| BP | GO:0030449 | regulation of complement activation | 7/240 | 0.00067613 | 0.011483751 | 0.008610291 | CFH/C3AR1/C5/C7/CR1/VSIG4/CPB2 | 7 |
| BP | GO:0070570 | regulation of neuron projection regeneration | 4/240 | 0.000694743 | 0.011679486 | 0.008757049 | EPHA4/HGF/THY1/PRRX1 | 4 |
| BP | GO:0090022 | regulation of neutrophil chemotaxis | 4/240 | 0.000694743 | 0.011679486 | 0.008757049 | CCL21/C3AR1/RIPOR2/EDN1 | 4 |
| BP | GO:0010657 | muscle cell apoptotic process | 6/240 | 0.000708256 | 0.011846209 | 0.008882055 | PDE1A/PDCD4/SFRP2/IGF1/HMOX1/EDN1 | 6 |
| BP | GO:0042493 | response to drug | 13/240 | 0.000725819 | 0.012078655 | 0.009056339 | PDE3A/ADRA1A/SFRP2/INHBA/NQO1/ITGA2/BCHE/ABCB1/HMOX1/TIMP4/CPB2/EDN1/ANKRD1 | 13 |
| BP | GO:0031349 | positive regulation of defense response | 13/240 | 0.000763933 | 0.012649037 | 0.009484 | S100A9/PDCD4/RASGRP1/S100A8/S100A12/GBP5/ITGA2/FABP4/HCK/MUC5B/CCL5/PTGS2/IDO1 | 13 |
| BP | GO:0001837 | epithelial to mesenchymal transition | 8/240 | 0.000776783 | 0.012726349 | 0.009541967 | EPHA3/PDCD4/S100A4/SFRP2/FGFR2/HGF/BMP5/HAS2 | 8 |
| BP | GO:0048762 | mesenchymal cell differentiation | 10/240 | 0.000779421 | 0.012726349 | 0.009541967 | EPHA3/PDCD4/S100A4/SFRP2/FGFR2/SEMA3D/HGF/BMP5/HAS2/EDN1 | 10 |
| BP | GO:0042692 | muscle cell differentiation | 13/240 | 0.000783613 | 0.012726349 | 0.009541967 | ADRA1A/RORA/PDCD4/FZD7/TBX3/CDON/IGF1/KIT/FGFR2/RIPOR2/CXCL9/EDN1/ANKRD1 | 13 |
| BP | GO:0046916 | cellular transition metal ion homeostasis | 7/240 | 0.000787914 | 0.012726349 | 0.009541967 | S100A9/LCN2/S100A8/ABCG2/BMP6/ALAS2/HMOX1 | 7 |
| BP | GO:0051928 | positive regulation of calcium ion transport | 7/240 | 0.000787914 | 0.012726349 | 0.009541967 | ATP2B1/TRPC6/CXCL12/THY1/CCR1/CXCL9/CCL5 | 7 |
| BP | GO:0055072 | iron ion homeostasis | 6/240 | 0.000802134 | 0.012892837 | 0.009666796 | LCN2/ABCG2/BMP6/ALAS2/STEAP2/HMOX1 | 6 |
| BP | GO:0060572 | morphogenesis of an epithelial bud | 3/240 | 0.000826634 | 0.013222136 | 0.009913698 | SULF1/FGFR2/SOSTDC1 | 3 |
| BP | GO:0010574 | regulation of vascular endothelial growth factor production | 5/240 | 0.000846421 | 0.013408445 | 0.010053389 | RORA/SULF1/C3AR1/C5/PTGS2 | 5 |
| BP | GO:0031663 | lipopolysaccharide-mediated signaling pathway | 5/240 | 0.000846421 | 0.013408445 | 0.010053389 | BMP6/CD14/HCK/CCL5/TNFAIP3 | 5 |
| BP | GO:1903039 | positive regulation of leukocyte cell-cell adhesion | 10/240 | 0.000860221 | 0.013561851 | 0.010168409 | VCAM1/CCL21/IGF1/LILRB2/SELP/THY1/CR1/HAS2/CCL5/SELE | 10 |
| BP | GO:0042098 | T cell proliferation | 9/240 | 0.000904749 | 0.014137855 | 0.010600286 | RASGRP1/VCAM1/IGF1/LILRB2/CR1/VSIG4/LRRC32/CCL5/IDO1 | 9 |
| BP | GO:0070664 | negative regulation of leukocyte proliferation | 6/240 | 0.000905338 | 0.014137855 | 0.010600286 | LILRB2/CR1/VSIG4/LRRC32/TNFAIP3/IDO1 | 6 |
| BP | GO:1902041 | regulation of extrinsic apoptotic signaling pathway via death domain receptors | 5/240 | 0.000915328 | 0.014198182 | 0.010645518 | SFRP2/HGF/BMP5/HMOX1/TNFAIP3 | 5 |
| BP | GO:0031348 | negative regulation of defense response | 10/240 | 0.000917819 | 0.014198182 | 0.010645518 | NT5E/RORA/SIGLEC10/IGF1/SAA1/NR1D2/HGF/CR1/VSIG4/TNFAIP3 | 10 |
| BP | GO:0071222 | cellular response to lipopolysaccharide | 9/240 | 0.000972247 | 0.014938182 | 0.011200356 | PDCD4/BMP6/CD14/LILRB2/HCK/CXCL9/CCL5/TNFAIP3/ANKRD1 | 9 |
| BP | GO:0051924 | regulation of calcium ion transport | 10/240 | 0.000978547 | 0.014938182 | 0.011200356 | PDE4D/ATP2B1/GEM/TRPC6/LILRB2/CXCL12/THY1/CCR1/CXCL9/CCL5 | 10 |
| BP | GO:0007588 | excretion | 5/240 | 0.000988323 | 0.014938182 | 0.011200356 | ADRA1A/ABCG2/AQP9/HMOX1/EDN1 | 5 |
| BP | GO:0034113 | heterotypic cell-cell adhesion | 5/240 | 0.000988323 | 0.014938182 | 0.011200356 | VCAM1/LILRB2/THY1/ITGAX/ITGB3 | 5 |
| BP | GO:0045576 | mast cell activation | 5/240 | 0.000988323 | 0.014938182 | 0.011200356 | FGR/S100A12/KIT/IL13RA2/HMOX1 | 5 |
| BP | GO:0019722 | calcium-mediated signaling | 9/240 | 0.001007496 | 0.014992604 | 0.01124116 | VCAM1/IGF1/CXCR2/CXCR1/SELP/TRAT1/CCR1/SELE/EDN1 | 9 |
| BP | GO:0002693 | positive regulation of cellular extravasation | 3/240 | 0.001007876 | 0.014992604 | 0.01124116 | PDGFD/THY1/RIPOR2 | 3 |
| BP | GO:0051238 | sequestering of metal ion | 3/240 | 0.001007876 | 0.014992604 | 0.01124116 | S100A9/LCN2/S100A8 | 3 |
| BP | GO:0003007 | heart morphogenesis | 10/240 | 0.001010124 | 0.014992604 | 0.01124116 | AHI1/PDCD4/TBX3/SFRP2/ZFPM2/FGFR2/BMP5/HAS2/ROBO2/ANKRD1 | 10 |
| BP | GO:0060349 | bone morphogenesis | 6/240 | 0.001018475 | 0.015048767 | 0.01128327 | FREM1/SFRP2/BMP6/FGFR2/HAS2/SFRP4 | 6 |
| BP | GO:0031214 | biomineral tissue development | 8/240 | 0.001038599 | 0.015209707 | 0.01140394 | FGR/ATP2B1/ASPN/BMP6/IGF1/ITGB6/FGFR2/CCR1 | 8 |
| BP | GO:0050728 | negative regulation of inflammatory response | 8/240 | 0.001038599 | 0.015209707 | 0.01140394 | NT5E/RORA/SIGLEC10/IGF1/SAA1/NR1D2/HGF/TNFAIP3 | 8 |
| BP | GO:0006937 | regulation of muscle contraction | 8/240 | 0.001081158 | 0.015762894 | 0.011818709 | PDE4D/ADRA1A/ATP2B1/PROK2/ACE2/KIT/ITGA2/EDN1 | 8 |
| BP | GO:0001990 | regulation of systemic arterial blood pressure by hormone | 4/240 | 0.001093655 | 0.015874855 | 0.011902654 | ACE2/CPA3/MME/EDN1 | 4 |
| BP | GO:0110148 | biomineralization | 8/240 | 0.001125098 | 0.016259647 | 0.012191164 | FGR/ATP2B1/ASPN/BMP6/IGF1/ITGB6/FGFR2/CCR1 | 8 |
| BP | GO:0061448 | connective tissue development | 10/240 | 0.001144901 | 0.016363339 | 0.01226891 | LUM/SULF1/ARID5B/SFRP2/PDGFD/BMP6/BMP5/EGR1/PRRX1/EDN1 | 10 |
| BP | GO:0010573 | vascular endothelial growth factor production | 5/240 | 0.001147172 | 0.016363339 | 0.01226891 | RORA/SULF1/C3AR1/C5/PTGS2 | 5 |
| BP | GO:0042698 | ovulation cycle | 5/240 | 0.001147172 | 0.016363339 | 0.01226891 | INHBA/HAS2/EGR1/TIMP4/ROBO2 | 5 |
| BP | GO:0010566 | regulation of ketone biosynthetic process | 3/240 | 0.001212404 | 0.016928316 | 0.012692519 | BMP6/BMP5/EGR1 | 3 |
| BP | GO:0060412 | ventricular septum morphogenesis | 4/240 | 0.001214097 | 0.016928316 | 0.012692519 | TBX3/ZFPM2/FGFR2/ROBO2 | 4 |
| BP | GO:1904994 | regulation of leukocyte adhesion to vascular endothelial cell | 4/240 | 0.001214097 | 0.016928316 | 0.012692519 | CCL21/CXCL12/SELP/SELE | 4 |
| BP | GO:1903034 | regulation of response to wounding | 8/240 | 0.001217257 | 0.016928316 | 0.012692519 | SIGLEC10/EPHA4/F11/SERPINE2/TNFAIP3/FAP/CPB2/EDN1 | 8 |
| BP | GO:0003018 | vascular process in circulatory system | 10/240 | 0.001217606 | 0.016928316 | 0.012692519 | PDE3A/ADRA1A/ABCC9/ATP2B1/ABCG2/BMP6/ACE2/SLC7A2/ABCB1/EDN1 | 10 |
| BP | GO:0010951 | negative regulation of endopeptidase activity | 10/240 | 0.001217606 | 0.016928316 | 0.012692519 | SFRP2/C5/OVOS2/HGF/CR1/TFPI2/SERPINE2/TIMP4/COL6A3/SERPINA3 | 10 |
| BP | GO:0071677 | positive regulation of mononuclear cell migration | 5/240 | 0.001233328 | 0.017074856 | 0.012802392 | PDGFD/CCL21/CXCL12/CCR1/CCL5 | 5 |
| BP | GO:0007584 | response to nutrient | 8/240 | 0.00126554 | 0.017447505 | 0.013081796 | POSTN/ATP2B1/VCAM1/SFRP2/NQO1/ITGA2/BCHE/HMOX1 | 8 |
| BP | GO:0034308 | primary alcohol metabolic process | 6/240 | 0.001277065 | 0.017533036 | 0.013145926 | ACSS2/BMP6/IGF1/SULT1B1/BMP5/AKR1C2 | 6 |
| BP | GO:0010959 | regulation of metal ion transport | 10/240 | 0.001294037 | 0.017692328 | 0.01326536 | PDE4D/ATP2B1/GEM/TRPC6/LILRB2/CXCL12/THY1/CCR1/CXCL9/CCL5 | 10 |
| BP | GO:0060411 | cardiac septum morphogenesis | 5/240 | 0.001324175 | 0.018029574 | 0.013518221 | TBX3/ZFPM2/FGFR2/BMP5/ROBO2 | 5 |
| BP | GO:0045823 | positive regulation of heart contraction | 4/240 | 0.001343523 | 0.018141533 | 0.013602165 | PDE4D/ADRA1A/ACE2/EDN1 | 4 |
| BP | GO:0048246 | macrophage chemotaxis | 4/240 | 0.001343523 | 0.018141533 | 0.013602165 | C3AR1/C5/SAA1/CCL5 | 4 |
| BP | GO:0032602 | chemokine production | 6/240 | 0.001348915 | 0.018141533 | 0.013602165 | POSTN/S100A9/S100A8/C5/EGR1/HMOX1 | 6 |
| BP | GO:0042129 | regulation of T cell proliferation | 8/240 | 0.001419605 | 0.019014622 | 0.01425679 | VCAM1/IGF1/LILRB2/CR1/VSIG4/LRRC32/CCL5/IDO1 | 8 |
| BP | GO:0060284 | regulation of cell development | 15/240 | 0.001440326 | 0.019072468 | 0.014300162 | PDE3A/POSTN/PLCB1/IGF1/KIT/TRPC6/MME/SEMA3D/EPHA4/CXCL12/THY1/HAS2/SERPINE2/ROBO2/OLFM4 | 15 |
| BP | GO:0001991 | regulation of systemic arterial blood pressure by circulatory renin-angiotensin | 3/240 | 0.001441288 | 0.019072468 | 0.014300162 | ACE2/CPA3/MME | 3 |
| BP | GO:0002523 | leukocyte migration involved in inflammatory response | 3/240 | 0.001441288 | 0.019072468 | 0.014300162 | S100A9/S100A8/SELE | 3 |
| BP | GO:0051047 | positive regulation of secretion | 11/240 | 0.001457419 | 0.019148376 | 0.014357077 | FGR/INHBA/ITGAM/S100A8/BMP6/MYH10/IGF1/CXCL12/CPB2/EDN1/ANKRD1 | 11 |
| BP | GO:0071560 | cellular response to transforming growth factor beta stimulus | 10/240 | 0.001458647 | 0.019148376 | 0.014357077 | LTBP1/PDE3A/POSTN/PDGFD/ASPN/ITGB6/FGFR2/LRRC32/EDN1/ANKRD1 | 10 |
| BP | GO:0071219 | cellular response to molecule of bacterial origin | 9/240 | 0.001468197 | 0.019197254 | 0.014393724 | PDCD4/BMP6/CD14/LILRB2/HCK/CXCL9/CCL5/TNFAIP3/ANKRD1 | 9 |
| BP | GO:0071356 | cellular response to tumor necrosis factor | 11/240 | 0.001497067 | 0.0194061 | 0.014550313 | POSTN/RORA/VCAM1/CCL21/FABP4/BIRC3/HAS2/CCL5/TNFAIP3/EDN1/ANKRD1 | 11 |
| BP | GO:0045807 | positive regulation of endocytosis | 6/240 | 0.001501838 | 0.0194061 | 0.014550313 | AHI1/CCL21/CD14/ITGA2/SFRP4/SELE | 6 |
| BP | GO:0060191 | regulation of lipase activity | 6/240 | 0.001501838 | 0.0194061 | 0.014550313 | ADRA1A/KIT/FGFR2/ITK/CCL5/SELE | 6 |
| BP | GO:0030193 | regulation of blood coagulation | 5/240 | 0.00152056 | 0.019495113 | 0.014617053 | F11/SERPINE2/FAP/CPB2/EDN1 | 5 |
| BP | GO:0042130 | negative regulation of T cell proliferation | 5/240 | 0.00152056 | 0.019495113 | 0.014617053 | LILRB2/CR1/VSIG4/LRRC32/IDO1 | 5 |
| BP | GO:1900046 | regulation of hemostasis | 5/240 | 0.001626408 | 0.020508454 | 0.015376836 | F11/SERPINE2/FAP/CPB2/EDN1 | 5 |
| BP | GO:0008207 | C21-steroid hormone metabolic process | 4/240 | 0.001630718 | 0.020508454 | 0.015376836 | BMP6/BMP5/AKR1C2/EGR1 | 4 |
| BP | GO:0042908 | xenobiotic transport | 4/240 | 0.001630718 | 0.020508454 | 0.015376836 | SLC36A1/ABCG2/ABCA8/ABCB1 | 4 |
| BP | GO:0055023 | positive regulation of cardiac muscle tissue growth | 4/240 | 0.001630718 | 0.020508454 | 0.015376836 | ZFPM2/IGF1/FGFR2/EDN1 | 4 |
| BP | GO:1903524 | positive regulation of blood circulation | 4/240 | 0.001630718 | 0.020508454 | 0.015376836 | PDE4D/ADRA1A/ACE2/EDN1 | 4 |
| BP | GO:0048705 | skeletal system morphogenesis | 9/240 | 0.001672677 | 0.020956162 | 0.015712519 | FGR/FREM1/ARID5B/SFRP2/BMP6/FGFR2/HAS2/SFRP4/PRRX1 | 9 |
| BP | GO:0001707 | mesoderm formation | 5/240 | 0.00173757 | 0.021604875 | 0.016198911 | SFRP2/INHBA/FGFR2/ITGA2/ITGB3 | 5 |
| BP | GO:0002548 | monocyte chemotaxis | 5/240 | 0.00173757 | 0.021604875 | 0.016198911 | S100A12/CCL21/CXCL12/CCR1/CCL5 | 5 |
| BP | GO:0014823 | response to activity | 5/240 | 0.001854202 | 0.022968403 | 0.017221257 | POSTN/BMP6/ITGA2/ANGPT2/EDN1 | 5 |
| BP | GO:2000027 | regulation of animal organ morphogenesis | 8/240 | 0.00190414 | 0.023498657 | 0.017618832 | NKD1/AHI1/FZD7/SULF1/SFRP2/HGF/ROBO2/EDN1 | 8 |
| BP | GO:0002920 | regulation of humoral immune response | 7/240 | 0.001955893 | 0.023855115 | 0.017886097 | CFH/C3AR1/C5/C7/CR1/VSIG4/CPB2 | 7 |
| BP | GO:1902622 | regulation of neutrophil migration | 4/240 | 0.001958004 | 0.023855115 | 0.017886097 | CCL21/C3AR1/RIPOR2/EDN1 | 4 |
| BP | GO:0015669 | gas transport | 3/240 | 0.001976138 | 0.023855115 | 0.017886097 | HBB/HBA2/CA2 | 3 |
| BP | GO:0046885 | regulation of hormone biosynthetic process | 3/240 | 0.001976138 | 0.023855115 | 0.017886097 | BMP6/BMP5/EGR1 | 3 |
| BP | GO:0090026 | positive regulation of monocyte chemotaxis | 3/240 | 0.001976138 | 0.023855115 | 0.017886097 | CXCL12/CCR1/CCL5 | 3 |
| BP | GO:0006879 | cellular iron ion homeostasis | 5/240 | 0.001976463 | 0.023855115 | 0.017886097 | LCN2/ABCG2/BMP6/ALAS2/HMOX1 | 5 |
| BP | GO:2001237 | negative regulation of extrinsic apoptotic signaling pathway | 6/240 | 0.002040522 | 0.024538398 | 0.018398409 | SFRP2/IGF1/HGF/BMP5/HMOX1/TNFAIP3 | 6 |
| BP | GO:0050818 | regulation of coagulation | 5/240 | 0.002104513 | 0.02517287 | 0.018874124 | F11/SERPINE2/FAP/CPB2/EDN1 | 5 |
| BP | GO:0046718 | viral entry into host cell | 7/240 | 0.002122315 | 0.02517287 | 0.018874124 | ITGB6/ACE2/FCN3/ITGA2/CR1/ANPEP/ITGB3 | 7 |
| BP | GO:0034142 | toll-like receptor 4 signaling pathway | 4/240 | 0.002137537 | 0.02517287 | 0.018874124 | ITGAM/CD14/BPIFB1/TNFAIP3 | 4 |
| BP | GO:0043300 | regulation of leukocyte degranulation | 4/240 | 0.002137537 | 0.02517287 | 0.018874124 | FGR/ITGAM/IL13RA2/HMOX1 | 4 |
| BP | GO:2000404 | regulation of T cell migration | 4/240 | 0.002137537 | 0.02517287 | 0.018874124 | CCL21/CXCL12/RIPOR2/CCL5 | 4 |
| BP | GO:0048608 | reproductive structure development | 13/240 | 0.002139121 | 0.02517287 | 0.018874124 | TBX3/SULF1/ARID5B/SFRP2/INHBA/ZFPM2/BMP6/KIT/FGFR2/MME/BMP5/SERPINE2/ROBO2 | 13 |
| BP | GO:0009615 | response to virus | 12/240 | 0.002255681 | 0.026406102 | 0.019798777 | FGR/RNASE2/ABCC9/ITGB6/BPIFA1/FCN3/CXCL12/BIRC3/IFI44L/CXCL9/CCL5/TNFAIP3 | 12 |
| BP | GO:0070661 | leukocyte proliferation | 11/240 | 0.002261421 | 0.026406102 | 0.019798777 | RASGRP1/VCAM1/IGF1/KIT/LILRB2/CR1/VSIG4/LRRC32/CCL5/TNFAIP3/IDO1 | 11 |
| BP | GO:0061458 | reproductive system development | 13/240 | 0.00228064 | 0.026406102 | 0.019798777 | TBX3/SULF1/ARID5B/SFRP2/INHBA/ZFPM2/BMP6/KIT/FGFR2/MME/BMP5/SERPINE2/ROBO2 | 13 |
| BP | GO:0009070 | serine family amino acid biosynthetic process | 3/240 | 0.002283988 | 0.026406102 | 0.019798777 | CBS/PHGDH/SHMT2 | 3 |
| BP | GO:0033630 | positive regulation of cell adhesion mediated by integrin | 3/240 | 0.002283988 | 0.026406102 | 0.019798777 | SFRP2/CCL21/CCL5 | 3 |
| BP | GO:0019730 | antimicrobial humoral response | 7/240 | 0.00229948 | 0.026492261 | 0.019863377 | S100A9/LCN2/S100A8/S100A12/BPIFA1/BPIFB1/CXCL9 | 7 |
| BP | GO:0010863 | positive regulation of phospholipase C activity | 4/240 | 0.002328114 | 0.026635889 | 0.019971067 | ADRA1A/KIT/ITK/SELE | 4 |
| BP | GO:0060421 | positive regulation of heart growth | 4/240 | 0.002328114 | 0.026635889 | 0.019971067 | ZFPM2/IGF1/FGFR2/EDN1 | 4 |
| BP | GO:0000302 | response to reactive oxygen species | 9/240 | 0.002354821 | 0.026749276 | 0.020056082 | HBB/HBA2/PDGFD/NQO1/TRPC6/HGF/HMOX1/TNFAIP3/EDN1 | 9 |
| BP | GO:0003151 | outflow tract morphogenesis | 5/240 | 0.002378615 | 0.026749276 | 0.020056082 | TBX3/SFRP2/ZFPM2/FGFR2/ROBO2 | 5 |
| BP | GO:0006809 | nitric oxide biosynthetic process | 5/240 | 0.002378615 | 0.026749276 | 0.020056082 | HBB/RORA/NQO1/PTGS2/EDN1 | 5 |
| BP | GO:0009166 | nucleotide catabolic process | 5/240 | 0.002378615 | 0.026749276 | 0.020056082 | PDE4D/PDE7B/NT5E/PDE1A/PDE8B | 5 |
| BP | GO:0031100 | animal organ regeneration | 5/240 | 0.002378615 | 0.026749276 | 0.020056082 | HGF/CXCL12/ANGPT2/HMOX1/CPB2 | 5 |
| BP | GO:0002526 | acute inflammatory response | 6/240 | 0.00247325 | 0.027718913 | 0.020783097 | VCAM1/S100A8/SAA1/SERPINA3/PTGS2/CD163 | 6 |
| BP | GO:0035051 | cardiocyte differentiation | 7/240 | 0.002487844 | 0.027787949 | 0.020834859 | ADRA1A/PDCD4/FZD7/TBX3/VCAM1/IGF1/EDN1 | 7 |
| BP | GO:0061045 | negative regulation of wound healing | 5/240 | 0.002524989 | 0.027975119 | 0.020975195 | F11/SERPINE2/FAP/CPB2/EDN1 | 5 |
| BP | GO:0071260 | cellular response to mechanical stimulus | 5/240 | 0.002524989 | 0.027975119 | 0.020975195 | PIEZO2/BMP6/ITGA2/RIPOR2/ANKRD1 | 5 |
| BP | GO:1900271 | regulation of long-term synaptic potentiation | 4/240 | 0.002530071 | 0.027975119 | 0.020975195 | MME/EPHA4/LILRB2/FAM107A | 4 |
| BP | GO:1903532 | positive regulation of secretion by cell | 10/240 | 0.002556349 | 0.028171142 | 0.021122169 | FGR/INHBA/ITGAM/BMP6/MYH10/IGF1/CXCL12/CPB2/EDN1/ANKRD1 | 10 |
| BP | GO:0034446 | substrate adhesion-dependent cell spreading | 6/240 | 0.002591455 | 0.028462814 | 0.021340859 | POSTN/ANTXR1/FZD7/HAS2/ITGB3/OLFM4 | 6 |
| BP | GO:0003081 | regulation of systemic arterial blood pressure by renin-angiotensin | 3/240 | 0.002619962 | 0.02849101 | 0.021362 | ACE2/CPA3/MME | 3 |
| BP | GO:0014821 | phasic smooth muscle contraction | 3/240 | 0.002619962 | 0.02849101 | 0.021362 | TBX3/KIT/EDN1 | 3 |
| BP | GO:0046058 | cAMP metabolic process | 3/240 | 0.002619962 | 0.02849101 | 0.021362 | PDE4D/PDE7B/PDE8B | 3 |
| BP | GO:0001818 | negative regulation of cytokine production | 12/240 | 0.002700722 | 0.02927263 | 0.021948043 | ATP2B1/PDCD4/INHBA/IGF1/IL1R2/HGF/CR1/VSIG4/LRRC32/HMOX1/TNFAIP3/IDO1 | 12 |
| BP | GO:0006953 | acute-phase response | 4/240 | 0.00274374 | 0.029352677 | 0.022008061 | SAA1/SERPINA3/PTGS2/CD163 | 4 |
| BP | GO:0043303 | mast cell degranulation | 4/240 | 0.00274374 | 0.029352677 | 0.022008061 | FGR/KIT/IL13RA2/HMOX1 | 4 |
| BP | GO:1900274 | regulation of phospholipase C activity | 4/240 | 0.00274374 | 0.029352677 | 0.022008061 | ADRA1A/KIT/ITK/SELE | 4 |
| BP | GO:2000107 | negative regulation of leukocyte apoptotic process | 4/240 | 0.00274374 | 0.029352677 | 0.022008061 | CCL21/CXCL12/CCL5/IDO1 | 4 |
| BP | GO:0022409 | positive regulation of cell-cell adhesion | 10/240 | 0.00291469 | 0.030831341 | 0.023116734 | VCAM1/CCL21/IGF1/LILRB2/SELP/THY1/CR1/HAS2/CCL5/SELE | 10 |
| BP | GO:0002279 | mast cell activation involved in immune response | 4/240 | 0.00296945 | 0.030831341 | 0.023116734 | FGR/KIT/IL13RA2/HMOX1 | 4 |
| BP | GO:0002448 | mast cell mediated immunity | 4/240 | 0.00296945 | 0.030831341 | 0.023116734 | FGR/KIT/IL13RA2/HMOX1 | 4 |
| BP | GO:0007157 | heterophilic cell-cell adhesion via plasma membrane cell adhesion molecules | 4/240 | 0.00296945 | 0.030831341 | 0.023116734 | HMCN1/VCAM1/SELP/SELE | 4 |
| BP | GO:1903053 | regulation of extracellular matrix organization | 4/240 | 0.00296945 | 0.030831341 | 0.023116734 | ANTXR1/HAS2/FAP/CPB2 | 4 |
| BP | GO:0031623 | receptor internalization | 6/240 | 0.002971587 | 0.030831341 | 0.023116734 | AHI1/CXCR2/CXCR1/SFRP4/ITGB3/SELE | 6 |
| BP | GO:0046660 | female sex differentiation | 6/240 | 0.002971587 | 0.030831341 | 0.023116734 | TBX3/ARID5B/INHBA/ZFPM2/KIT/ROBO2 | 6 |
| BP | GO:0031639 | plasminogen activation | 3/240 | 0.002984886 | 0.030831341 | 0.023116734 | F11/SERPINE2/CPB2 | 3 |
| BP | GO:0060384 | innervation | 3/240 | 0.002984886 | 0.030831341 | 0.023116734 | VCAM1/SULF1/SERPINE2 | 3 |
| BP | GO:0071676 | negative regulation of mononuclear cell migration | 3/240 | 0.002984886 | 0.030831341 | 0.023116734 | PLCB1/BMP5/RIPOR2 | 3 |
| BP | GO:0120255 | olefinic compound biosynthetic process | 3/240 | 0.002984886 | 0.030831341 | 0.023116734 | BMP6/BMP5/EGR1 | 3 |
| BP | GO:0046209 | nitric oxide metabolic process | 5/240 | 0.003003324 | 0.030924854 | 0.023186849 | HBB/RORA/NQO1/PTGS2/EDN1 | 5 |
| BP | GO:0071216 | cellular response to biotic stimulus | 9/240 | 0.003063551 | 0.031349068 | 0.023504916 | PDCD4/BMP6/CD14/LILRB2/HCK/CXCL9/CCL5/TNFAIP3/ANKRD1 | 9 |
| BP | GO:0097305 | response to alcohol | 9/240 | 0.003063551 | 0.031349068 | 0.023504916 | ATP2B1/VCAM1/INHBA/NQO1/S100A8/CCL21/CD14/FGFR2/AKR1C2 | 9 |
| BP | GO:2001057 | reactive nitrogen species metabolic process | 5/240 | 0.003176378 | 0.032402985 | 0.024295122 | HBB/RORA/NQO1/PTGS2/EDN1 | 5 |
| BP | GO:0010656 | negative regulation of muscle cell apoptotic process | 4/240 | 0.003207524 | 0.032619725 | 0.024457629 | SFRP2/IGF1/HMOX1/EDN1 | 4 |
| BP | GO:0050863 | regulation of T cell activation | 11/240 | 0.003240348 | 0.032852149 | 0.024631896 | VCAM1/CCL21/IGF1/LILRB2/THY1/CR1/RIPOR2/VSIG4/LRRC32/CCL5/IDO1 | 11 |
| BP | GO:0010560 | positive regulation of glycoprotein biosynthetic process | 3/240 | 0.003379535 | 0.034158186 | 0.025611137 | PLCB1/CCL21/IGF1 | 3 |
| BP | GO:0010976 | positive regulation of neuron projection development | 7/240 | 0.003486599 | 0.034918977 | 0.026181564 | S100A9/EPHA3/CNTN1/DPYSL3/HGF/BMP5/ANKRD1 | 7 |
| BP | GO:0044409 | entry into host | 7/240 | 0.003486599 | 0.034918977 | 0.026181564 | ITGB6/ACE2/FCN3/ITGA2/CR1/ANPEP/ITGB3 | 7 |
| BP | GO:0046661 | male sex differentiation | 7/240 | 0.003486599 | 0.034918977 | 0.026181564 | TBX3/ARID5B/SFRP2/INHBA/ZFPM2/BMP6/KIT | 7 |
| BP | GO:0001704 | formation of primary germ layer | 6/240 | 0.003541439 | 0.035066137 | 0.026291901 | FZD7/SFRP2/INHBA/FGFR2/ITGA2/ITGB3 | 6 |
| BP | GO:0003206 | cardiac chamber morphogenesis | 6/240 | 0.003541439 | 0.035066137 | 0.026291901 | TBX3/SFRP2/ZFPM2/FGFR2/BMP5/ROBO2 | 6 |
| BP | GO:1990748 | cellular detoxification | 6/240 | 0.003541439 | 0.035066137 | 0.026291901 | HBB/HBA2/S100A9/NQO1/ABCG2/PTGS2 | 6 |
| BP | GO:2000243 | positive regulation of reproductive process | 5/240 | 0.003543862 | 0.035066137 | 0.026291901 | PDE3A/INHBA/ZFPM2/PLCB1/CFAP69 | 5 |
| BP | GO:0000187 | activation of MAPK activity | 7/240 | 0.003614031 | 0.035546959 | 0.026652412 | PROK2/IGF1/C5/KIT/SAA1/HGF/FPR1 | 7 |
| BP | GO:2001236 | regulation of extrinsic apoptotic signaling pathway | 7/240 | 0.003614031 | 0.035546959 | 0.026652412 | SFRP2/INHBA/IGF1/HGF/BMP5/HMOX1/TNFAIP3 | 7 |
| BP | GO:0003229 | ventricular cardiac muscle tissue development | 4/240 | 0.003722048 | 0.036392129 | 0.027286104 | TBX3/ZFPM2/FGFR2/ADAMTS9 | 4 |
| BP | GO:0048260 | positive regulation of receptor-mediated endocytosis | 4/240 | 0.003722048 | 0.036392129 | 0.027286104 | AHI1/CCL21/SFRP4/SELE | 4 |
| BP | GO:0050672 | negative regulation of lymphocyte proliferation | 5/240 | 0.003738613 | 0.036442755 | 0.027324062 | LILRB2/CR1/VSIG4/LRRC32/IDO1 | 5 |
| BP | GO:0002092 | positive regulation of receptor internalization | 3/240 | 0.003804646 | 0.036442755 | 0.027324062 | AHI1/SFRP4/SELE | 3 |
| BP | GO:0003148 | outflow tract septum morphogenesis | 3/240 | 0.003804646 | 0.036442755 | 0.027324062 | ZFPM2/FGFR2/ROBO2 | 3 |
| BP | GO:0017145 | stem cell division | 3/240 | 0.003804646 | 0.036442755 | 0.027324062 | FZD7/SFRP2/KIT | 3 |
| BP | GO:0042730 | fibrinolysis | 3/240 | 0.003804646 | 0.036442755 | 0.027324062 | F11/FAP/CPB2 | 3 |
| BP | GO:0072012 | glomerulus vasculature development | 3/240 | 0.003804646 | 0.036442755 | 0.027324062 | PDGFD/ANGPT2/EGR1 | 3 |
| BP | GO:2000050 | regulation of non-canonical Wnt signaling pathway | 3/240 | 0.003804646 | 0.036442755 | 0.027324062 | NKD1/SFRP2/SFRP4 | 3 |
| BP | GO:0070663 | regulation of leukocyte proliferation | 9/240 | 0.003826029 | 0.036541354 | 0.02739799 | VCAM1/IGF1/LILRB2/CR1/VSIG4/LRRC32/CCL5/TNFAIP3/IDO1 | 9 |
| BP | GO:0032945 | negative regulation of mononuclear cell proliferation | 5/240 | 0.003940919 | 0.037529846 | 0.028139142 | LILRB2/CR1/VSIG4/LRRC32/IDO1 | 5 |
| BP | GO:0043900 | regulation of multi-organism process | 4/240 | 0.003999127 | 0.03797442 | 0.028472475 | PLCB1/BMP6/CFAP69/TNFAIP3 | 4 |
| BP | GO:0002224 | toll-like receptor signaling pathway | 7/240 | 0.004017661 | 0.038040788 | 0.028522236 | S100A9/ITGAM/S100A8/CD14/BPIFB1/BIRC3/TNFAIP3 | 7 |
| BP | GO:0090257 | regulation of muscle system process | 9/240 | 0.0040381 | 0.038124752 | 0.02858519 | PDE4D/ADRA1A/ATP2B1/PROK2/IGF1/ACE2/KIT/ITGA2/EDN1 | 9 |
| BP | GO:0055006 | cardiac cell development | 5/240 | 0.004150937 | 0.038856074 | 0.029133522 | ADRA1A/PDCD4/TBX3/IGF1/EDN1 | 5 |
| BP | GO:0060291 | long-term synaptic potentiation | 5/240 | 0.004150937 | 0.038856074 | 0.029133522 | MME/EPHA4/LILRB2/FAM107A/SERPINE2 | 5 |
| BP | GO:1901292 | nucleoside phosphate catabolic process | 5/240 | 0.004150937 | 0.038856074 | 0.029133522 | PDE4D/PDE7B/NT5E/PDE1A/PDE8B | 5 |
| BP | GO:0050868 | negative regulation of T cell activation | 6/240 | 0.004188807 | 0.039099483 | 0.029316025 | LILRB2/CR1/RIPOR2/VSIG4/LRRC32/IDO1 | 6 |
| BP | GO:0006700 | C21-steroid hormone biosynthetic process | 3/240 | 0.004260909 | 0.039217023 | 0.029404154 | BMP6/BMP5/EGR1 | 3 |
| BP | GO:0010460 | positive regulation of heart rate | 3/240 | 0.004260909 | 0.039217023 | 0.029404154 | PDE4D/ADRA1A/EDN1 | 3 |
| BP | GO:0050926 | regulation of positive chemotaxis | 3/240 | 0.004260909 | 0.039217023 | 0.029404154 | ITGA2/CXCL12/ANGPT2 | 3 |
| BP | GO:0061437 | renal system vasculature development | 3/240 | 0.004260909 | 0.039217023 | 0.029404154 | PDGFD/ANGPT2/EGR1 | 3 |
| BP | GO:0061440 | kidney vasculature development | 3/240 | 0.004260909 | 0.039217023 | 0.029404154 | PDGFD/ANGPT2/EGR1 | 3 |
| BP | GO:0002886 | regulation of myeloid leukocyte mediated immunity | 4/240 | 0.00428983 | 0.039294906 | 0.029462549 | FGR/ITGAM/IL13RA2/HMOX1 | 4 |
| BP | GO:0051147 | regulation of muscle cell differentiation | 7/240 | 0.004305148 | 0.039294906 | 0.029462549 | PDCD4/FZD7/CDON/IGF1/KIT/FGFR2/EDN1 | 7 |
| BP | GO:2000241 | regulation of reproductive process | 7/240 | 0.004305148 | 0.039294906 | 0.029462549 | PDE3A/SULF1/INHBA/ZFPM2/PLCB1/IGF1/CFAP69 | 7 |
| BP | GO:0008625 | extrinsic apoptotic signaling pathway via death domain receptors | 5/240 | 0.004368828 | 0.039765994 | 0.029815761 | SFRP2/HGF/BMP5/HMOX1/TNFAIP3 | 5 |
| BP | GO:0010811 | positive regulation of cell-substrate adhesion | 6/240 | 0.004543451 | 0.041241518 | 0.03092208 | ECM2/CCL21/CCDC80/THY1/HAS2/OLFM4 | 6 |
| BP | GO:0002090 | regulation of receptor internalization | 4/240 | 0.004594461 | 0.041476028 | 0.031097912 | AHI1/SFRP4/ITGB3/SELE | 4 |
| BP | GO:1905517 | macrophage migration | 4/240 | 0.004594461 | 0.041476028 | 0.031097912 | C3AR1/C5/SAA1/CCL5 | 4 |
| BP | GO:0019218 | regulation of steroid metabolic process | 6/240 | 0.004728846 | 0.041951395 | 0.031454333 | RORA/BMP6/KIT/ACADL/BMP5/EGR1 | 6 |
| BP | GO:0097237 | cellular response to toxic substance | 6/240 | 0.004728846 | 0.041951395 | 0.031454333 | HBB/HBA2/S100A9/NQO1/ABCG2/PTGS2 | 6 |
| BP | GO:0003401 | axis elongation | 3/240 | 0.004748974 | 0.041951395 | 0.031454333 | NKD1/SFRP2/FGFR2 | 3 |
| BP | GO:0010758 | regulation of macrophage chemotaxis | 3/240 | 0.004748974 | 0.041951395 | 0.031454333 | C3AR1/C5/CCL5 | 3 |
| BP | GO:0010955 | negative regulation of protein processing | 3/240 | 0.004748974 | 0.041951395 | 0.031454333 | IL1R2/SERPINE2/CPB2 | 3 |
| BP | GO:0090025 | regulation of monocyte chemotaxis | 3/240 | 0.004748974 | 0.041951395 | 0.031454333 | CXCL12/CCR1/CCL5 | 3 |
| BP | GO:1903020 | positive regulation of glycoprotein metabolic process | 3/240 | 0.004748974 | 0.041951395 | 0.031454333 | PLCB1/CCL21/IGF1 | 3 |
| BP | GO:1903318 | negative regulation of protein maturation | 3/240 | 0.004748974 | 0.041951395 | 0.031454333 | IL1R2/SERPINE2/CPB2 | 3 |
| BP | GO:0030199 | collagen fibril organization | 4/240 | 0.004913319 | 0.043113237 | 0.032325459 | COL14A1/LUM/SFRP2/DPT | 4 |
| BP | GO:0046622 | positive regulation of organ growth | 4/240 | 0.004913319 | 0.043113237 | 0.032325459 | ZFPM2/IGF1/FGFR2/EDN1 | 4 |
| BP | GO:1903409 | reactive oxygen species biosynthetic process | 6/240 | 0.00491975 | 0.043113237 | 0.032325459 | HBB/RORA/NQO1/SLC18A2/PTGS2/EDN1 | 6 |
| BP | GO:0030100 | regulation of endocytosis | 8/240 | 0.004974042 | 0.043295186 | 0.032461881 | AHI1/EPHA3/CCL21/CD14/ITGA2/SFRP4/ITGB3/SELE | 8 |
| BP | GO:0071456 | cellular response to hypoxia | 8/240 | 0.004974042 | 0.043295186 | 0.032461881 | RORA/TRPC6/HIF3A/EGR1/HMOX1/PTGS2/EDN1/ANKRD1 | 8 |
| BP | GO:0090092 | regulation of transmembrane receptor protein serine/threonine kinase signaling pathway | 9/240 | 0.004979932 | 0.043295186 | 0.032461881 | LTBP1/SULF1/SFRP2/INHBA/ASPN/BMP6/SOSTDC1/BMP5/SFRP4 | 9 |
| BP | GO:0045927 | positive regulation of growth | 9/240 | 0.005240102 | 0.045333803 | 0.033990395 | S100A9/SFRP2/ZFPM2/S100A8/PLCB1/IGF1/FGFR2/CXCL12/EDN1 | 9 |
| BP | GO:0002507 | tolerance induction | 3/240 | 0.005269453 | 0.045333803 | 0.033990395 | LILRB2/TNFAIP3/IDO1 | 3 |
| BP | GO:0032703 | negative regulation of interleukin-2 production | 3/240 | 0.005269453 | 0.045333803 | 0.033990395 | CR1/VSIG4/TNFAIP3 | 3 |
| BP | GO:0060603 | mammary gland duct morphogenesis | 3/240 | 0.005269453 | 0.045333803 | 0.033990395 | TBX3/FGFR2/SOSTDC1 | 3 |
| BP | GO:0042180 | cellular ketone metabolic process | 9/240 | 0.005374079 | 0.046113513 | 0.034575007 | SLC7A7/NQO1/BMP6/ACADL/BMP5/AKR1C2/EGR1/PTGS2/IDO1 | 9 |
| BP | GO:0032612 | interleukin-1 production | 6/240 | 0.005526469 | 0.047297966 | 0.035463086 | IGF1/SAA1/GBP5/IL1R2/EGR1/TNFAIP3 | 6 |
| BP | GO:0019216 | regulation of lipid metabolic process | 12/240 | 0.005562371 | 0.047481895 | 0.035600993 | FGR/RORA/BMP6/CCL21/KIT/ACADL/NR1D2/BMP5/EGR1/SERPINA3/PTGS2/ANKRD1 | 12 |
| BP | GO:0010038 | response to metal ion | 11/240 | 0.005604563 | 0.047718437 | 0.035778347 | VCAM1/NQO1/S100A8/BMP6/CD14/KIT/FABP4/CA2/AQP9/HMOX1/EDN1 | 11 |
| BP | GO:0043112 | receptor metabolic process | 7/240 | 0.005795485 | 0.049216813 | 0.0369018 | AHI1/CXCR2/CXCR1/TRAT1/SFRP4/ITGB3/SELE | 7 |
| BP | GO:0043304 | regulation of mast cell degranulation | 3/240 | 0.005822914 | 0.04932263 | 0.03698114 | FGR/IL13RA2/HMOX1 | 3 |
| BP | GO:0070301 | cellular response to hydrogen peroxide | 5/240 | 0.005850345 | 0.049427916 | 0.037060081 | PDGFD/NQO1/TRPC6/HGF/TNFAIP3 | 5 |
| BP | GO:0046328 | regulation of JNK cascade | 6/240 | 0.006186198 | 0.05213177 | 0.039087378 | PDCD4/FZD7/RASGRP1/SFRP2/PLCB1/CCL21 | 6 |
| BP | GO:0010631 | epithelial cell migration | 11/240 | 0.006209663 | 0.052184557 | 0.039126957 | ENPP2/KIT/ITGA2/ADAMTS9/ANGPT2/HAS2/HMOX1/ITGB3/FAP/PTGS2/EDN1 | 11 |
| BP | GO:0036294 | cellular response to decreased oxygen levels | 8/240 | 0.006224137 | 0.052184557 | 0.039126957 | RORA/TRPC6/HIF3A/EGR1/HMOX1/PTGS2/EDN1/ANKRD1 | 8 |
| BP | GO:0031102 | neuron projection regeneration | 4/240 | 0.006336806 | 0.052574747 | 0.039419513 | EPHA4/HGF/THY1/PRRX1 | 4 |
| BP | GO:0006816 | calcium ion transport | 12/240 | 0.006352601 | 0.052574747 | 0.039419513 | PDE4D/ADRA1A/ATP2B1/GEM/CCL21/TRPC6/LILRB2/CXCL12/THY1/CCR1/CXCL9/CCL5 | 12 |
| BP | GO:0033006 | regulation of mast cell activation involved in immune response | 3/240 | 0.006409892 | 0.052574747 | 0.039419513 | FGR/IL13RA2/HMOX1 | 3 |
| BP | GO:0033137 | negative regulation of peptidyl-serine phosphorylation | 3/240 | 0.006409892 | 0.052574747 | 0.039419513 | PDE4D/CNKSR3/HGF | 3 |
| BP | GO:0034694 | response to prostaglandin | 3/240 | 0.006409892 | 0.052574747 | 0.039419513 | CCL21/AKR1C2/EDN1 | 3 |
| BP | GO:0051491 | positive regulation of filopodium assembly | 3/240 | 0.006409892 | 0.052574747 | 0.039419513 | DPYSL3/CCL21/RIPOR2 | 3 |
| BP | GO:0070633 | transepithelial transport | 3/240 | 0.006409892 | 0.052574747 | 0.039419513 | ABCG2/ABCB1/EDN1 | 3 |
| BP | GO:2000406 | positive regulation of T cell migration | 3/240 | 0.006409892 | 0.052574747 | 0.039419513 | CCL21/CXCL12/CCL5 | 3 |
| BP | GO:0055017 | cardiac muscle tissue growth | 5/240 | 0.006414279 | 0.052574747 | 0.039419513 | ADRA1A/ZFPM2/IGF1/FGFR2/EDN1 | 5 |
| BP | GO:0090132 | epithelium migration | 11/240 | 0.006596672 | 0.053703131 | 0.040265553 | ENPP2/KIT/ITGA2/ADAMTS9/ANGPT2/HAS2/HMOX1/ITGB3/FAP/PTGS2/EDN1 | 11 |
| BP | GO:0061041 | regulation of wound healing | 6/240 | 0.006656733 | 0.053703131 | 0.040265553 | F11/SERPINE2/TNFAIP3/FAP/CPB2/EDN1 | 6 |
| BP | GO:0008585 | female gonad development | 5/240 | 0.00671008 | 0.053703131 | 0.040265553 | ARID5B/INHBA/ZFPM2/KIT/ROBO2 | 5 |
| BP | GO:0032663 | regulation of interleukin-2 production | 4/240 | 0.006731095 | 0.053703131 | 0.040265553 | PDE4D/CR1/VSIG4/TNFAIP3 | 4 |
| BP | GO:0015824 | proline transport | 2/240 | 0.006783003 | 0.053703131 | 0.040265553 | SLC36A1/ACE2 | 2 |
| BP | GO:0032341 | aldosterone metabolic process | 2/240 | 0.006783003 | 0.053703131 | 0.040265553 | BMP6/BMP5 | 2 |
| BP | GO:0032342 | aldosterone biosynthetic process | 2/240 | 0.006783003 | 0.053703131 | 0.040265553 | BMP6/BMP5 | 2 |
| BP | GO:0035524 | proline transmembrane transport | 2/240 | 0.006783003 | 0.053703131 | 0.040265553 | SLC36A1/ACE2 | 2 |
| BP | GO:0035860 | glial cell-derived neurotrophic factor receptor signaling pathway | 2/240 | 0.006783003 | 0.053703131 | 0.040265553 | SULF1/GFRA1 | 2 |
| BP | GO:0043301 | negative regulation of leukocyte degranulation | 2/240 | 0.006783003 | 0.053703131 | 0.040265553 | IL13RA2/HMOX1 | 2 |
| BP | GO:0070099 | regulation of chemokine-mediated signaling pathway | 2/240 | 0.006783003 | 0.053703131 | 0.040265553 | CCL5/EDN1 | 2 |
| BP | GO:0072124 | regulation of glomerular mesangial cell proliferation | 2/240 | 0.006783003 | 0.053703131 | 0.040265553 | PDGFD/EGR1 | 2 |
| BP | GO:0072178 | nephric duct morphogenesis | 2/240 | 0.006783003 | 0.053703131 | 0.040265553 | AHI1/EPHA4 | 2 |
| BP | GO:0090030 | regulation of steroid hormone biosynthetic process | 2/240 | 0.006783003 | 0.053703131 | 0.040265553 | BMP6/BMP5 | 2 |
| BP | GO:0002697 | regulation of immune effector process | 13/240 | 0.006796421 | 0.053703131 | 0.040265553 | FGR/RASGRP1/CFH/ITGAM/C3AR1/C5/C7/CR1/IL13RA2/VSIG4/HMOX1/TNFAIP3/CPB2 | 13 |
| BP | GO:0042445 | hormone metabolic process | 8/240 | 0.006932264 | 0.054645475 | 0.040972105 | BMP6/ACE2/CPA3/MME/SULT1B1/BMP5/AKR1C2/EGR1 | 8 |
| BP | GO:0061097 | regulation of protein tyrosine kinase activity | 5/240 | 0.007015305 | 0.055027931 | 0.041258862 | ADRA1A/EPHA4/THY1/AREG/CCL5 | 5 |
| BP | GO:0019835 | cytolysis | 3/240 | 0.007030883 | 0.055027931 | 0.041258862 | C5/C7/CR1 | 3 |
| BP | GO:0042044 | fluid transport | 3/240 | 0.007030883 | 0.055027931 | 0.041258862 | HAS2/AQP9/EDN1 | 3 |
| BP | GO:0032722 | positive regulation of chemokine production | 4/240 | 0.007141294 | 0.055418076 | 0.041551385 | POSTN/C5/EGR1/HMOX1 | 4 |
| BP | GO:0061098 | positive regulation of protein tyrosine kinase activity | 4/240 | 0.007141294 | 0.055418076 | 0.041551385 | ADRA1A/EPHA4/AREG/CCL5 | 4 |
| BP | GO:0010634 | positive regulation of epithelial cell migration | 7/240 | 0.007198463 | 0.055418076 | 0.041551385 | ENPP2/ITGA2/HAS2/HMOX1/ITGB3/PTGS2/EDN1 | 7 |
| BP | GO:0045766 | positive regulation of angiogenesis | 7/240 | 0.007198463 | 0.055418076 | 0.041551385 | SFRP2/C3AR1/C5/HGF/ITGAX/ANGPT2/HMOX1 | 7 |
| BP | GO:0051897 | positive regulation of protein kinase B signaling | 7/240 | 0.007198463 | 0.055418076 | 0.041551385 | CCL21/KIT/FGFR2/HGF/TRAT1/AREG/AKR1C2 | 7 |
| BP | GO:0052126 | movement in host environment | 7/240 | 0.007198463 | 0.055418076 | 0.041551385 | ITGB6/ACE2/FCN3/ITGA2/CR1/ANPEP/ITGB3 | 7 |
| BP | GO:1904018 | positive regulation of vasculature development | 7/240 | 0.007198463 | 0.055418076 | 0.041551385 | SFRP2/C3AR1/C5/HGF/ITGAX/ANGPT2/HMOX1 | 7 |
| BP | GO:0030111 | regulation of Wnt signaling pathway | 11/240 | 0.0072835 | 0.055723317 | 0.041780249 | NKD1/BICC1/FZD7/SULF1/SFRP2/WIF1/FGFR2/SOSTDC1/EGR1/SFRP4/TNFAIP3 | 11 |
| BP | GO:0090130 | tissue migration | 11/240 | 0.0072835 | 0.055723317 | 0.041780249 | ENPP2/KIT/ITGA2/ADAMTS9/ANGPT2/HAS2/HMOX1/ITGB3/FAP/PTGS2/EDN1 | 11 |
| BP | GO:0045088 | regulation of innate immune response | 10/240 | 0.007288847 | 0.055723317 | 0.041780249 | FGR/RASGRP1/GBP5/HCK/CR1/VSIG4/BIRC3/MUC5B/CCL5/TNFAIP3 | 10 |
| BP | GO:0050670 | regulation of lymphocyte proliferation | 8/240 | 0.007502128 | 0.057191385 | 0.042880977 | VCAM1/IGF1/LILRB2/CR1/VSIG4/LRRC32/CCL5/IDO1 | 8 |
| BP | GO:0019229 | regulation of vasoconstriction | 4/240 | 0.007567661 | 0.057191385 | 0.042880977 | ADRA1A/ATP2B1/ACE2/EDN1 | 4 |
| BP | GO:0032623 | interleukin-2 production | 4/240 | 0.007567661 | 0.057191385 | 0.042880977 | PDE4D/CR1/VSIG4/TNFAIP3 | 4 |
| BP | GO:1903961 | positive regulation of anion transmembrane transport | 4/240 | 0.007567661 | 0.057191385 | 0.042880977 | IGF1/ACE2/ABCB1/CA2 | 4 |
| BP | GO:1905330 | regulation of morphogenesis of an epithelium | 4/240 | 0.007567661 | 0.057191385 | 0.042880977 | NKD1/SULF1/HGF/ITGAX | 4 |
| BP | GO:0007517 | muscle organ development | 10/240 | 0.007604752 | 0.057340177 | 0.042992539 | ARID5B/ZFPM2/CDON/IGF1/FGFR2/NR1D2/RIPOR2/EGR1/COL6A3/ANKRD1 | 10 |
| BP | GO:0003279 | cardiac septum development | 5/240 | 0.007654627 | 0.057429725 | 0.04305968 | TBX3/ZFPM2/FGFR2/BMP5/ROBO2 | 5 |
| BP | GO:0007202 | activation of phospholipase C activity | 3/240 | 0.007686346 | 0.057429725 | 0.04305968 | ADRA1A/ITK/SELE | 3 |
| BP | GO:0010453 | regulation of cell fate commitment | 3/240 | 0.007686346 | 0.057429725 | 0.04305968 | FZD7/SFRP2/SOSTDC1 | 3 |
| BP | GO:0016486 | peptide hormone processing | 3/240 | 0.007686346 | 0.057429725 | 0.04305968 | ACE2/CPA3/MME | 3 |
| BP | GO:0051146 | striated muscle cell differentiation | 9/240 | 0.007719814 | 0.057549292 | 0.04314933 | ADRA1A/FZD7/TBX3/CDON/IGF1/RIPOR2/CXCL9/EDN1/ANKRD1 | 9 |
| BP | GO:0001667 | ameboidal-type cell migration | 13/240 | 0.007787664 | 0.057924046 | 0.043430313 | ARID5B/ENPP2/KIT/SEMA3D/ITGA2/ADAMTS9/ANGPT2/HAS2/HMOX1/ITGB3/FAP/PTGS2/EDN1 | 13 |
| BP | GO:0032944 | regulation of mononuclear cell proliferation | 8/240 | 0.007901196 | 0.05863613 | 0.043964219 | VCAM1/IGF1/LILRB2/CR1/VSIG4/LRRC32/CCL5/IDO1 | 8 |
| BP | GO:0051235 | maintenance of location | 10/240 | 0.00793114 | 0.058704808 | 0.044015713 | LTBP1/PDE4D/S100A9/LCN2/S100A8/CCL21/SLC18A2/THY1/CXCL9/ITGB3 | 10 |
| BP | GO:0045598 | regulation of fat cell differentiation | 6/240 | 0.007946083 | 0.058704808 | 0.044015713 | RORA/SFRP2/ZFPM2/WIF1/MEDAG/PTGS2 | 6 |
| BP | GO:0046545 | development of primary female sexual characteristics | 5/240 | 0.007989017 | 0.058889959 | 0.044154535 | ARID5B/INHBA/ZFPM2/KIT/ROBO2 | 5 |
| BP | GO:0032835 | glomerulus development | 4/240 | 0.008010454 | 0.058916171 | 0.044174188 | SULF1/PDGFD/ANGPT2/EGR1 | 4 |
| BP | GO:0002645 | positive regulation of tolerance induction | 2/240 | 0.008221165 | 0.059535688 | 0.04463869 | LILRB2/IDO1 | 2 |
| BP | GO:0042541 | hemoglobin biosynthetic process | 2/240 | 0.008221165 | 0.059535688 | 0.04463869 | INHBA/ALAS2 | 2 |
| BP | GO:0051610 | serotonin uptake | 2/240 | 0.008221165 | 0.059535688 | 0.04463869 | SLC18A2/ITGB3 | 2 |
| BP | GO:0072110 | glomerular mesangial cell proliferation | 2/240 | 0.008221165 | 0.059535688 | 0.04463869 | PDGFD/EGR1 | 2 |
| BP | GO:0097048 | dendritic cell apoptotic process | 2/240 | 0.008221165 | 0.059535688 | 0.04463869 | CCL21/CXCL12 | 2 |
| BP | GO:0098883 | synapse pruning | 2/240 | 0.008221165 | 0.059535688 | 0.04463869 | ITGAM/EPHA4 | 2 |
| BP | GO:2000668 | regulation of dendritic cell apoptotic process | 2/240 | 0.008221165 | 0.059535688 | 0.04463869 | CCL21/CXCL12 | 2 |
| BP | GO:0003298 | physiological muscle hypertrophy | 3/240 | 0.008376706 | 0.059742957 | 0.044794096 | ADRA1A/IGF1/EDN1 | 3 |
| BP | GO:0003301 | physiological cardiac muscle hypertrophy | 3/240 | 0.008376706 | 0.059742957 | 0.044794096 | ADRA1A/IGF1/EDN1 | 3 |
| BP | GO:0032350 | regulation of hormone metabolic process | 3/240 | 0.008376706 | 0.059742957 | 0.044794096 | BMP6/BMP5/EGR1 | 3 |
| BP | GO:0043552 | positive regulation of phosphatidylinositol 3-kinase activity | 3/240 | 0.008376706 | 0.059742957 | 0.044794096 | FGR/CCL21/KIT | 3 |
| BP | GO:0050869 | negative regulation of B cell activation | 3/240 | 0.008376706 | 0.059742957 | 0.044794096 | INHBA/CR1/TNFAIP3 | 3 |
| BP | GO:0061049 | cell growth involved in cardiac muscle cell development | 3/240 | 0.008376706 | 0.059742957 | 0.044794096 | ADRA1A/IGF1/EDN1 | 3 |
| BP | GO:0140448 | signaling receptor ligand precursor processing | 3/240 | 0.008376706 | 0.059742957 | 0.044794096 | ACE2/CPA3/MME | 3 |
| BP | GO:0043491 | protein kinase B signaling | 9/240 | 0.008460619 | 0.060018033 | 0.045000343 | CCL21/IGF1/KIT/FGFR2/HGF/TRAT1/AREG/AKR1C2/CCL5 | 9 |
| BP | GO:0048662 | negative regulation of smooth muscle cell proliferation | 4/240 | 0.00846992 | 0.060018033 | 0.045000343 | PDCD4/OGN/HMOX1/TNFAIP3 | 4 |
| BP | GO:2000401 | regulation of lymphocyte migration | 4/240 | 0.00846992 | 0.060018033 | 0.045000343 | CCL21/CXCL12/RIPOR2/CCL5 | 4 |
| BP | GO:0001889 | liver development | 6/240 | 0.008508994 | 0.06003669 | 0.045014331 | ARID5B/HGF/ITGA2/HMOX1/TNFAIP3/CPB2 | 6 |
| BP | GO:0098754 | detoxification | 6/240 | 0.008508994 | 0.06003669 | 0.045014331 | HBB/HBA2/S100A9/NQO1/ABCG2/PTGS2 | 6 |
| BP | GO:0032872 | regulation of stress-activated MAPK cascade | 7/240 | 0.008589124 | 0.06047257 | 0.045341146 | PDCD4/FZD7/RASGRP1/SFRP2/PLCB1/CCL21/HGF | 7 |
| BP | GO:0019233 | sensory perception of pain | 5/240 | 0.008687986 | 0.061038199 | 0.045765243 | PROK2/MME/ITGA2/CXCL12/EDN1 | 5 |
| BP | GO:0015849 | organic acid transport | 10/240 | 0.008794439 | 0.061654628 | 0.04622743 | SLC7A7/SLC36A1/ABCG2/ACE2/SLC7A2/FABP4/ABCB1/AQP9/SLCO1A2/EDN1 | 10 |
| BP | GO:0003281 | ventricular septum development | 4/240 | 0.008946303 | 0.062321496 | 0.046727434 | TBX3/ZFPM2/FGFR2/ROBO2 | 4 |
| BP | GO:0035914 | skeletal muscle cell differentiation | 4/240 | 0.008946303 | 0.062321496 | 0.046727434 | CDON/NR1D2/EGR1/ANKRD1 | 4 |
| BP | GO:0050922 | negative regulation of chemotaxis | 4/240 | 0.008946303 | 0.062321496 | 0.046727434 | C5/SEMA3D/ANGPT2/ROBO2 | 4 |
| BP | GO:0050890 | cognition | 9/240 | 0.009050798 | 0.062798203 | 0.047084859 | LRRN4/PLCB1/AFF2/KIT/MME/LILRB2/BCHE/FAM107A/ADGRF1 | 9 |
| BP | GO:0060419 | heart growth | 5/240 | 0.009052852 | 0.062798203 | 0.047084859 | ADRA1A/ZFPM2/IGF1/FGFR2/EDN1 | 5 |
| BP | GO:0061008 | hepaticobiliary system development | 6/240 | 0.00909997 | 0.062876858 | 0.047143834 | ARID5B/HGF/ITGA2/HMOX1/TNFAIP3/CPB2 | 6 |
| BP | GO:0031646 | positive regulation of nervous system process | 3/240 | 0.009102356 | 0.062876858 | 0.047143834 | HGF/ITGA2/ITGAX | 3 |
| BP | GO:0003002 | regionalization | 10/240 | 0.00915928 | 0.063137717 | 0.047339421 | NKD1/AHI1/TBX3/SFRP2/ITGAM/CDON/FGFR2/SOSTDC1/ROBO2/EDN1 | 10 |
| BP | GO:0070302 | regulation of stress-activated protein kinase signaling cascade | 7/240 | 0.009354173 | 0.064346554 | 0.048245783 | PDCD4/FZD7/RASGRP1/SFRP2/PLCB1/CCL21/HGF | 7 |
| BP | GO:0007188 | adenylate cyclase-modulating G protein-coupled receptor signaling pathway | 8/240 | 0.009424505 | 0.064531683 | 0.048384589 | PDE4D/ADRA1A/RGS1/FPR1/VIPR1/CXCL9/ADGRF1/EDN1 | 8 |
| BP | GO:2000379 | positive regulation of reactive oxygen species metabolic process | 5/240 | 0.009428159 | 0.064531683 | 0.048384589 | HBB/ITGAM/ACE2/PTGS2/EDN1 | 5 |
| BP | GO:1904888 | cranial skeletal system development | 4/240 | 0.009439839 | 0.064531683 | 0.048384589 | FREM1/FGFR2/COLEC10/PRRX1 | 4 |
| BP | GO:0010565 | regulation of cellular ketone metabolic process | 7/240 | 0.009619942 | 0.06460029 | 0.04843603 | SLC7A7/NQO1/BMP6/ACADL/BMP5/EGR1/PTGS2 | 7 |
| BP | GO:0051216 | cartilage development | 7/240 | 0.009619942 | 0.06460029 | 0.04843603 | LUM/SULF1/SFRP2/BMP6/BMP5/PRRX1/EDN1 | 7 |
| BP | GO:0071453 | cellular response to oxygen levels | 8/240 | 0.009658814 | 0.06460029 | 0.04843603 | RORA/TRPC6/HIF3A/EGR1/HMOX1/PTGS2/EDN1/ANKRD1 | 8 |
| BP | GO:0001768 | establishment of T cell polarity | 2/240 | 0.00978317 | 0.06460029 | 0.04843603 | CCL21/RIPOR2 | 2 |
| BP | GO:0002664 | regulation of T cell tolerance induction | 2/240 | 0.00978317 | 0.06460029 | 0.04843603 | LILRB2/IDO1 | 2 |
| BP | GO:0014831 | gastro-intestinal system smooth muscle contraction | 2/240 | 0.00978317 | 0.06460029 | 0.04843603 | SULF1/KIT | 2 |
| BP | GO:0032352 | positive regulation of hormone metabolic process | 2/240 | 0.00978317 | 0.06460029 | 0.04843603 | BMP6/EGR1 | 2 |
| BP | GO:0033004 | negative regulation of mast cell activation | 2/240 | 0.00978317 | 0.06460029 | 0.04843603 | IL13RA2/HMOX1 | 2 |
| BP | GO:0034392 | negative regulation of smooth muscle cell apoptotic process | 2/240 | 0.00978317 | 0.06460029 | 0.04843603 | IGF1/EDN1 | 2 |
| BP | GO:0042340 | keratan sulfate catabolic process | 2/240 | 0.00978317 | 0.06460029 | 0.04843603 | LUM/OGN | 2 |
| BP | GO:0044849 | estrous cycle | 2/240 | 0.00978317 | 0.06460029 | 0.04843603 | HAS2/EGR1 | 2 |
| BP | GO:0045989 | positive regulation of striated muscle contraction | 2/240 | 0.00978317 | 0.06460029 | 0.04843603 | ADRA1A/ACE2 | 2 |
| BP | GO:0060601 | lateral sprouting from an epithelium | 2/240 | 0.00978317 | 0.06460029 | 0.04843603 | SULF1/FGFR2 | 2 |
| BP | GO:0061051 | positive regulation of cell growth involved in cardiac muscle cell development | 2/240 | 0.00978317 | 0.06460029 | 0.04843603 | IGF1/EDN1 | 2 |
| BP | GO:0080154 | regulation of fertilization | 2/240 | 0.00978317 | 0.06460029 | 0.04843603 | PLCB1/CFAP69 | 2 |
| BP | GO:1900272 | negative regulation of long-term synaptic potentiation | 2/240 | 0.00978317 | 0.06460029 | 0.04843603 | EPHA4/FAM107A | 2 |
| BP | GO:1904424 | regulation of GTP binding | 2/240 | 0.00978317 | 0.06460029 | 0.04843603 | EPHA4/RIPOR2 | 2 |
| BP | GO:0098869 | cellular oxidant detoxification | 5/240 | 0.009814048 | 0.064674578 | 0.048491729 | HBB/HBA2/S100A9/NQO1/PTGS2 | 5 |
| BP | GO:0048009 | insulin-like growth factor receptor signaling pathway | 3/240 | 0.009863653 | 0.064797938 | 0.048584222 | PLCB1/IGF1/BMP5 | 3 |
| BP | GO:0071214 | cellular response to abiotic stimulus | 10/240 | 0.009923674 | 0.064797938 | 0.048584222 | SFRP2/PIEZO2/BMP6/ITGB6/MME/ITGA2/RIPOR2/EGR1/MFAP4/ANKRD1 | 10 |
| BP | GO:0104004 | cellular response to environmental stimulus | 10/240 | 0.009923674 | 0.064797938 | 0.048584222 | SFRP2/PIEZO2/BMP6/ITGB6/MME/ITGA2/RIPOR2/EGR1/MFAP4/ANKRD1 | 10 |
| BP | GO:0015807 | L-amino acid transport | 4/240 | 0.009950761 | 0.064797938 | 0.048584222 | SLC7A7/SLC36A1/ACE2/SLC7A2 | 4 |
| BP | GO:0045600 | positive regulation of fat cell differentiation | 4/240 | 0.009950761 | 0.064797938 | 0.048584222 | SFRP2/WIF1/MEDAG/PTGS2 | 4 |
| BP | GO:0072678 | T cell migration | 4/240 | 0.009950761 | 0.064797938 | 0.048584222 | CCL21/CXCL12/RIPOR2/CCL5 | 4 |
| BP | GO:0072593 | reactive oxygen species metabolic process | 9/240 | 0.010103142 | 0.065660459 | 0.049230923 | HBB/HBA2/RORA/NQO1/ITGAM/ACE2/SLC18A2/PTGS2/EDN1 | 9 |
| BP | GO:0007200 | phospholipase C-activating G protein-coupled receptor signaling pathway | 5/240 | 0.010210657 | 0.06622857 | 0.049656882 | ADRA1A/C3AR1/CXCR2/FPR1/EDN1 | 5 |
| CC | GO:0062023 | collagen-containing extracellular matrix | 33/251 | 9.33E-17 | 2.48E-14 | 1.93E-14 | LTBP1/COL14A1/POSTN/S100A9/MATN2/HMCN1/FREM1/S100A4/LUM/SULF1/SFRP2/MFGE8/OGN/ASPN/S100A8/CDON/MXRA5/CCDC80/CPA3/FGFR2/FCN3/CXCL12/ADAMTS9/ANGPT2/MFAP4/SERPINE2/FRAS1/COL6A6/COL6A3/COL15A1/SERPINA3/DPT/THBS2 | 33 |
| CC | GO:0009897 | external side of plasma membrane | 26/251 | 1.49E-11 | 1.98E-09 | 1.54E-09 | CSF3R/NT5E/ANTXR1/VCAM1/MFGE8/ITGAM/ABCG2/CA4/CD69/CD14/GFRA1/ITGB6/KIT/CXCR2/CXCR1/ITGA2/CXCL12/SELP/ABCB1/THY1/CCR1/IL13RA2/BTNL9/SERPINE2/CXCL9/CD163 | 26 |
| CC | GO:0030667 | secretory granule membrane | 19/251 | 1.69E-08 | 1.50E-06 | 1.17E-06 | LILRB3/SIGLEC9/ITGAM/CA4/CD14/C3AR1/MGAM/CXCR2/MME/CXCR1/LILRA3/LILRB2/FPR1/SELP/CR1/ANPEP/ITGAX/OLR1/ITGB3 | 19 |
| CC | GO:0045121 | membrane raft | 17/251 | 1.05E-06 | 5.59E-05 | 4.36E-05 | ADRA1A/ATP2B1/SULF1/CNTN1/MALL/ITGAM/ABCG2/CD14/ACE2/MME/THY1/HCK/CR1/HAS2/OLR1/HMOX1/SELE | 17 |
| CC | GO:0098857 | membrane microdomain | 17/251 | 1.05E-06 | 5.59E-05 | 4.36E-05 | ADRA1A/ATP2B1/SULF1/CNTN1/MALL/ITGAM/ABCG2/CD14/ACE2/MME/THY1/HCK/CR1/HAS2/OLR1/HMOX1/SELE | 17 |
| CC | GO:0008305 | integrin complex | 6/251 | 2.39E-06 | 0.000106149 | 8.28E-05 | ITGAM/ITGB6/ITGA2/ITGAX/ITGB3/ITGBL1 | 6 |
| CC | GO:0098636 | protein complex involved in cell adhesion | 6/251 | 6.00E-06 | 0.000228082 | 0.000177809 | ITGAM/ITGB6/ITGA2/ITGAX/ITGB3/ITGBL1 | 6 |
| CC | GO:0070820 | tertiary granule | 11/251 | 9.29E-06 | 0.000308759 | 0.000240703 | HBB/ITGAM/MGAM/LILRA3/LILRB2/CHIT1/FPR1/CR1/ITGAX/OLR1/OLFM4 | 11 |
| CC | GO:0031256 | leading edge membrane | 10/251 | 8.39E-05 | 0.002481 | 0.00193414 | PSD3/FGR/ANTXR1/ATP2B1/THY1/RIPOR2/FAM107A/ITGB3/ROBO2/FAP | 10 |
| CC | GO:0005581 | collagen trimer | 7/251 | 0.000130234 | 0.003163939 | 0.002466545 | COL14A1/LUM/FCN3/COLEC10/COL6A6/COL6A3/COL15A1 | 7 |
| CC | GO:0101003 | ficolin-1-rich granule membrane | 6/251 | 0.00013084 | 0.003163939 | 0.002466545 | MGAM/LILRA3/LILRB2/FPR1/CR1/ITGAX | 6 |
| CC | GO:0031091 | platelet alpha granule | 7/251 | 0.000172719 | 0.003828605 | 0.002984706 | IGF1/HGF/SELP/SERPINE2/ITGB3/SERPINA3/THBS2 | 7 |
| CC | GO:0070821 | tertiary granule membrane | 6/251 | 0.000352838 | 0.007219599 | 0.005628259 | ITGAM/MGAM/LILRA3/LILRB2/ITGAX/OLR1 | 6 |
| CC | GO:0031253 | cell projection membrane | 13/251 | 0.000446325 | 0.008480177 | 0.006610981 | PSD3/FGR/ANTXR1/ATP2B1/ABCG2/CA4/ACE2/THY1/RIPOR2/FAM107A/ITGB3/ROBO2/FAP | 13 |
| CC | GO:0044853 | plasma membrane raft | 7/251 | 0.000583401 | 0.010345638 | 0.008065258 | ADRA1A/ITGAM/HCK/CR1/HAS2/HMOX1/SELE | 7 |
| CC | GO:0045177 | apical part of cell | 14/251 | 0.000991371 | 0.015519441 | 0.012098654 | SLC9A3R2/PDE4D/ATP2B1/VCAM1/ABCG2/CA4/ACE2/MGAM/CNKSR3/ABCB1/THY1/CA2/RIPOR2/FAP | 14 |
| CC | GO:0034774 | secretory granule lumen | 12/251 | 0.000991844 | 0.015519441 | 0.012098654 | FGR/S100A9/RNASE2/LCN2/S100A8/S100A12/IGF1/PLAC8/HGF/CHIT1/SERPINA3/OLFM4 | 12 |
| CC | GO:0060205 | cytoplasmic vesicle lumen | 12/251 | 0.00110256 | 0.016263205 | 0.012678478 | FGR/S100A9/RNASE2/LCN2/S100A8/S100A12/IGF1/PLAC8/HGF/CHIT1/SERPINA3/OLFM4 | 12 |
| CC | GO:0031983 | vesicle lumen | 12/251 | 0.001161657 | 0.016263205 | 0.012678478 | FGR/S100A9/RNASE2/LCN2/S100A8/S100A12/IGF1/PLAC8/HGF/CHIT1/SERPINA3/OLFM4 | 12 |
| CC | GO:0031362 | anchored component of external side of plasma membrane | 3/251 | 0.00174795 | 0.023247732 | 0.01812348 | CA4/CD14/THY1 | 3 |
| CC | GO:0071682 | endocytic vesicle lumen | 3/251 | 0.002037005 | 0.025802069 | 0.020114791 | HBB/HBA2/SAA1 | 3 |
| CC | GO:0031258 | lamellipodium membrane | 3/251 | 0.002700102 | 0.032646692 | 0.025450725 | ANTXR1/ITGB3/FAP | 3 |
| CC | GO:0031233 | intrinsic component of external side of plasma membrane | 3/251 | 0.003075871 | 0.035573113 | 0.027732106 | CA4/CD14/THY1 | 3 |
| CC | GO:0042581 | specific granule | 7/251 | 0.004722407 | 0.052340015 | 0.040803257 | LCN2/ITGAM/C3AR1/LILRA3/CHIT1/OLR1/OLFM4 | 7 |
| CC | GO:0016324 | apical plasma membrane | 11/251 | 0.005945534 | 0.063260482 | 0.04931664 | SLC9A3R2/PDE4D/ATP2B1/ABCG2/CA4/ACE2/MGAM/CNKSR3/ABCB1/THY1/RIPOR2 | 11 |
| CC | GO:0005788 | endoplasmic reticulum lumen | 10/251 | 0.006452084 | 0.063583632 | 0.049568562 | LTBP1/COL14A1/MFGE8/PDGFD/BCHE/ERAP2/COL6A3/COL15A1/PTGS2/EDN1 | 10 |
| CC | GO:0031225 | anchored component of membrane | 7/251 | 0.006536739 | 0.063583632 | 0.049568562 | NT5E/CNTN1/CA4/CD14/GFRA1/THY1/VNN2 | 7 |
| CC | GO:0032589 | neuron projection membrane | 4/251 | 0.006985238 | 0.063583632 | 0.049568562 | ATP2B1/THY1/RIPOR2/ROBO2 | 4 |
| CC | GO:0031252 | cell leading edge | 12/251 | 0.007169113 | 0.063583632 | 0.049568562 | PSD3/FGR/ANTXR1/ATP2B1/DPYSL3/MYH10/THY1/RIPOR2/FAM107A/ITGB3/ROBO2/FAP | 12 |
| CC | GO:0005604 | basement membrane | 5/251 | 0.007331264 | 0.063583632 | 0.049568562 | HMCN1/FREM1/CCDC80/FRAS1/COL15A1 | 5 |
| CC | GO:0046658 | anchored component of plasma membrane | 4/251 | 0.007410123 | 0.063583632 | 0.049568562 | CNTN1/CA4/CD14/THY1 | 4 |
| CC | GO:0032587 | ruffle membrane | 5/251 | 0.00765938 | 0.063668599 | 0.0496348 | PSD3/FGR/FAM107A/ITGB3/FAP | 5 |
| MF | GO:0005201 | extracellular matrix structural constituent | 18/248 | 1.72E-11 | 8.10E-09 | 6.33E-09 | LTBP1/COL14A1/POSTN/MATN2/HMCN1/LUM/MFGE8/OGN/ASPN/MXRA5/TFPI2/MFAP4/FRAS1/COL6A6/COL6A3/COL15A1/DPT/THBS2 | 18 |
| MF | GO:0005178 | integrin binding | 16/248 | 9.22E-11 | 2.17E-08 | 1.70E-08 | ECM2/VCAM1/SFRP2/MFGE8/ITGAM/CCN5/ESM1/IGF1/ITGB6/ITGA2/CXCL12/THY1/ITGAX/ITGB3/FAP/ITGBL1 | 16 |
| MF | GO:0008081 | phosphoric diester hydrolase activity | 11/248 | 3.20E-08 | 5.02E-06 | 3.93E-06 | PDE3A/PDE4D/PDE7B/PDE1A/PLCB4/PLCB1/ENPP2/PDE8B/CCR1/HMOX1/CCL5 | 11 |
| MF | GO:0050786 | RAGE receptor binding | 5/248 | 1.04E-07 | 1.22E-05 | 9.55E-06 | S100A9/S100A4/S100A8/S100A12/FPR1 | 5 |
| MF | GO:0019955 | cytokine binding | 11/248 | 2.44E-06 | 0.00018333 | 0.000143402 | LTBP1/CSF3R/KIT/CXCR2/CXCR1/IL1R2/SOSTDC1/CCR1/IL13RA2/LRRC32/ITGB3 | 11 |
| MF | GO:0140375 | immune receptor activity | 11/248 | 2.44E-06 | 0.00018333 | 0.000143402 | CSF3R/GFRA1/C3AR1/CXCR2/CXCR1/IL1R2/LILRB2/FPR1/CR1/CCR1/IL13RA2 | 11 |
| MF | GO:1901681 | sulfur compound binding | 15/248 | 2.72E-06 | 0.00018333 | 0.000143402 | ECM2/POSTN/CBS/CFH/DPYSL3/CCN5/CCDC80/SAA1/FGFR2/ACADL/ITGA2/SELP/F11/SERPINE2/THBS2 | 15 |
| MF | GO:0008238 | exopeptidase activity | 9/248 | 5.59E-06 | 0.00032937 | 0.000257636 | AGBL1/ACE2/CPA3/MME/F11/ANPEP/ERAP2/FAP/CPB2 | 9 |
| MF | GO:0004114 | 3',5'-cyclic-nucleotide phosphodiesterase activity | 5/248 | 1.20E-05 | 0.000627131 | 0.000490549 | PDE3A/PDE4D/PDE7B/PDE1A/PDE8B | 5 |
| MF | GO:0005539 | glycosaminoglycan binding | 13/248 | 1.44E-05 | 0.000632356 | 0.000494635 | ECM2/POSTN/SULF1/CFH/DPYSL3/CCN5/CCDC80/SAA1/FGFR2/SELP/F11/SERPINE2/THBS2 | 13 |
| MF | GO:0008201 | heparin binding | 11/248 | 1.48E-05 | 0.000632356 | 0.000494635 | ECM2/POSTN/CFH/CCN5/CCDC80/SAA1/FGFR2/SELP/F11/SERPINE2/THBS2 | 11 |
| MF | GO:0030246 | carbohydrate binding | 14/248 | 1.74E-05 | 0.000670544 | 0.000524507 | FREM1/SIGLEC9/CNTN1/SIGLEC10/ENPP2/CD69/MGAM/LGALSL/FCN3/SELP/OLR1/COLEC10/SELE/CHI3L2 | 14 |
| MF | GO:0004112 | cyclic-nucleotide phosphodiesterase activity | 5/248 | 1.85E-05 | 0.000670544 | 0.000524507 | PDE3A/PDE4D/PDE7B/PDE1A/PDE8B | 5 |
| MF | GO:0004115 | 3',5'-cyclic-AMP phosphodiesterase activity | 4/248 | 2.12E-05 | 0.000713683 | 0.000558251 | PDE3A/PDE4D/PDE7B/PDE8B | 4 |
| MF | GO:0008083 | growth factor activity | 10/248 | 7.32E-05 | 0.002297812 | 0.001797372 | MACC1/INHBA/OGN/PDGFD/BMP6/IGF1/HGF/CXCL12/BMP5/AREG | 10 |
| MF | GO:0048018 | receptor ligand activity | 18/248 | 0.000116442 | 0.003427761 | 0.00268123 | MACC1/SFRP2/INHBA/OGN/PDGFD/BMP6/CCL21/IGF1/C5/SEMA3D/HGF/CXCL12/BMP5/AREG/COLEC10/CXCL9/CCL5/EDN1 | 18 |
| MF | GO:0030546 | signaling receptor activator activity | 18/248 | 0.000135668 | 0.003758812 | 0.002940181 | MACC1/SFRP2/INHBA/OGN/PDGFD/BMP6/CCL21/IGF1/C5/SEMA3D/HGF/CXCL12/BMP5/AREG/COLEC10/CXCL9/CCL5/EDN1 | 18 |
| MF | GO:0033691 | sialic acid binding | 4/248 | 0.000197199 | 0.005160044 | 0.004036239 | SIGLEC9/SIGLEC10/SELP/SELE | 4 |
| MF | GO:0031406 | carboxylic acid binding | 10/248 | 0.000209006 | 0.005181148 | 0.004052747 | S100A9/SIGLEC9/S100A8/SIGLEC10/SHMT2/ACOXL/FABP4/SELP/AKR1C2/SELE | 10 |
| MF | GO:0008235 | metalloexopeptidase activity | 6/248 | 0.000224152 | 0.005278784 | 0.004129119 | AGBL1/ACE2/CPA3/ANPEP/ERAP2/CPB2 | 6 |
| MF | GO:0005518 | collagen binding | 6/248 | 0.00031285 | 0.00701677 | 0.00548859 | COL14A1/ECM2/ANTXR1/LUM/ASPN/ITGA2 | 6 |
| MF | GO:0004896 | cytokine receptor activity | 7/248 | 0.000347361 | 0.00743669 | 0.005817055 | CSF3R/GFRA1/CXCR2/CXCR1/IL1R2/CCR1/IL13RA2 | 7 |
| MF | GO:0008559 | ABC-type xenobiotic transporter activity | 3/248 | 0.000372165 | 0.007580478 | 0.005929528 | ABCG2/ABCA8/ABCB1 | 3 |
| MF | GO:0004435 | phosphatidylinositol phospholipase C activity | 4/248 | 0.000386266 | 0.007580478 | 0.005929528 | PLCB4/PLCB1/CCR1/CCL5 | 4 |
| MF | GO:0004875 | complement receptor activity | 3/248 | 0.000491272 | 0.008712083 | 0.006814681 | C3AR1/FPR1/CR1 | 3 |
| MF | GO:0004713 | protein tyrosine kinase activity | 8/248 | 0.000506213 | 0.008712083 | 0.006814681 | FGR/EPHA3/KIT/WEE1/FGFR2/EPHA4/ITK/HCK | 8 |
| MF | GO:0008009 | chemokine activity | 5/248 | 0.00051004 | 0.008712083 | 0.006814681 | CCL21/C5/CXCL12/CXCL9/CCL5 | 5 |
| MF | GO:0004629 | phospholipase C activity | 4/248 | 0.000517916 | 0.008712083 | 0.006814681 | PLCB4/PLCB1/CCR1/CCL5 | 4 |
| MF | GO:0001618 | virus receptor activity | 6/248 | 0.000571218 | 0.009016463 | 0.00705277 | ITGB6/ACE2/ITGA2/CR1/ANPEP/ITGB3 | 6 |
| MF | GO:0004181 | metallocarboxypeptidase activity | 4/248 | 0.000594449 | 0.009016463 | 0.00705277 | AGBL1/ACE2/CPA3/CPB2 | 4 |
| MF | GO:0001664 | G protein-coupled receptor binding | 12/248 | 0.000596526 | 0.009016463 | 0.00705277 | SLC9A3R2/PDE4D/FZD7/PROK2/CCL21/C5/SAA1/FPR1/CXCL12/CXCL9/CCL5/EDN1 | 12 |
| MF | GO:0140272 | exogenous protein binding | 6/248 | 0.000612583 | 0.009016463 | 0.00705277 | ITGB6/ACE2/ITGA2/CR1/ANPEP/ITGB3 | 6 |
| MF | GO:0036041 | long-chain fatty acid binding | 3/248 | 0.000632289 | 0.009024488 | 0.007059048 | S100A9/S100A8/FABP4 | 3 |
| MF | GO:0017147 | Wnt-protein binding | 4/248 | 0.000678676 | 0.009133042 | 0.007143959 | FZD7/SFRP2/WIF1/SFRP4 | 4 |
| MF | GO:0042910 | xenobiotic transmembrane transporter activity | 4/248 | 0.000678676 | 0.009133042 | 0.007143959 | SLC36A1/ABCG2/ABCA8/ABCB1 | 4 |
| MF | GO:0019199 | transmembrane receptor protein kinase activity | 6/248 | 0.000750741 | 0.009822194 | 0.007683021 | LTBP1/EPHA3/KIT/FGFR2/EPHA4/SOSTDC1 | 6 |
| MF | GO:0004089 | carbonate dehydratase activity | 3/248 | 0.000796718 | 0.010142 | 0.007933177 | CA4/CA2/CA12 | 3 |
| MF | GO:0008237 | metallopeptidase activity | 9/248 | 0.000920362 | 0.011407649 | 0.008923181 | AGBL1/ACE2/CPA3/MME/ANPEP/ADAMTS9/TLL1/ERAP2/CPB2 | 9 |
| MF | GO:0019956 | chemokine binding | 4/248 | 0.000981702 | 0.011609986 | 0.009081451 | CXCR2/CXCR1/CCR1/ITGB3 | 4 |
| MF | GO:0001846 | opsonin binding | 3/248 | 0.000985986 | 0.011609986 | 0.009081451 | ITGAM/CR1/VSIG4 | 3 |
| MF | GO:0030414 | peptidase inhibitor activity | 9/248 | 0.001031466 | 0.011849277 | 0.009268626 | PI15/C5/OVOS2/BIRC3/TFPI2/SERPINE2/TIMP4/COL6A3/SERPINA3 | 9 |
| MF | GO:0061134 | peptidase regulator activity | 10/248 | 0.001194699 | 0.013397697 | 0.010479817 | SFRP2/PI15/C5/OVOS2/BIRC3/TFPI2/SERPINE2/TIMP4/COL6A3/SERPINA3 | 10 |
| MF | GO:0061135 | endopeptidase regulator activity | 9/248 | 0.001240627 | 0.013589197 | 0.01062961 | SFRP2/C5/OVOS2/BIRC3/TFPI2/SERPINE2/TIMP4/COL6A3/SERPINA3 | 9 |
| MF | GO:0005125 | cytokine activity | 10/248 | 0.001403317 | 0.015021872 | 0.011750263 | INHBA/BMP6/CCL21/C5/CXCL12/BMP5/AREG/CXCL9/CCL5/EDN1 | 10 |
| MF | GO:0005504 | fatty acid binding | 4/248 | 0.001518896 | 0.015743309 | 0.012314579 | S100A9/S100A8/ACOXL/FABP4 | 4 |
| MF | GO:0042578 | phosphoric ester hydrolase activity | 13/248 | 0.001537563 | 0.015743309 | 0.012314579 | PDE3A/PDE4D/PDE7B/NT5E/PDE1A/PLCB4/PLCB1/ENPP2/PLPPR4/PDE8B/CCR1/HMOX1/CCL5 | 13 |
| MF | GO:0030020 | extracellular matrix structural constituent conferring tensile strength | 4/248 | 0.002232778 | 0.022375284 | 0.017502178 | COL14A1/COL6A6/COL6A3/COL15A1 | 4 |
| MF | GO:0042379 | chemokine receptor binding | 5/248 | 0.002415654 | 0.023472556 | 0.018360475 | CCL21/C5/CXCL12/CXCL9/CCL5 | 5 |
| MF | GO:0004180 | carboxypeptidase activity | 4/248 | 0.002441943 | 0.023472556 | 0.018360475 | AGBL1/ACE2/CPA3/CPB2 | 4 |
| MF | GO:0019838 | growth factor binding | 7/248 | 0.002639503 | 0.024802197 | 0.019400534 | LTBP1/CCN5/ESM1/FGFR2/IL1R2/LRRC32/ITGB3 | 7 |
| MF | GO:0001848 | complement binding | 3/248 | 0.002714621 | 0.024802197 | 0.019400534 | ITGAM/CR1/VSIG4 | 3 |
| MF | GO:0033293 | monocarboxylic acid binding | 5/248 | 0.002738247 | 0.024802197 | 0.019400534 | S100A9/S100A8/ACOXL/FABP4/AKR1C2 | 5 |
| MF | GO:0004620 | phospholipase activity | 6/248 | 0.003041215 | 0.026650966 | 0.02084666 | PLCB4/PLCB1/ENPP2/CCR1/HMOX1/CCL5 | 6 |
| MF | GO:0030021 | extracellular matrix structural constituent conferring compression resistance | 3/248 | 0.003112109 | 0.026650966 | 0.02084666 | LUM/OGN/ASPN | 3 |
| MF | GO:0140359 | ABC-type transporter activity | 3/248 | 0.003112109 | 0.026650966 | 0.02084666 | ABCG2/ABCA8/ABCB1 | 3 |
| MF | GO:0004866 | endopeptidase inhibitor activity | 8/248 | 0.003181098 | 0.026755308 | 0.020928277 | C5/OVOS2/BIRC3/TFPI2/SERPINE2/TIMP4/COL6A3/SERPINA3 | 8 |
| MF | GO:0004715 | non-membrane spanning protein tyrosine kinase activity | 4/248 | 0.003413403 | 0.028205492 | 0.022062626 | FGR/WEE1/ITK/HCK | 4 |
| MF | GO:0016493 | C-C chemokine receptor activity | 3/248 | 0.003543496 | 0.028775632 | 0.022508596 | CXCR2/CXCR1/CCR1 | 3 |
| MF | GO:0042277 | peptide binding | 11/248 | 0.003867089 | 0.030871169 | 0.024147746 | INHBA/ITGAM/CD14/MME/EPHA4/LILRB2/ITGA2/BCHE/ANPEP/VIPR1/ERAP2 | 11 |
| MF | GO:0019957 | C-C chemokine binding | 3/248 | 0.004009647 | 0.030959737 | 0.024217025 | CXCR2/CXCR1/CCR1 | 3 |
| MF | GO:0070696 | transmembrane receptor protein serine/threonine kinase binding | 3/248 | 0.004009647 | 0.030959737 | 0.024217025 | INHBA/BMP6/BMP5 | 3 |
| MF | GO:0046906 | tetrapyrrole binding | 7/248 | 0.004357455 | 0.033102601 | 0.025893196 | HBB/HBA2/CBS/TCN2/HMOX1/PTGS2/IDO1 | 7 |
| MF | GO:0001637 | G protein-coupled chemoattractant receptor activity | 3/248 | 0.00504943 | 0.037160651 | 0.029067444 | CXCR2/CXCR1/CCR1 | 3 |
| MF | GO:0004950 | chemokine receptor activity | 3/248 | 0.00504943 | 0.037160651 | 0.029067444 | CXCR2/CXCR1/CCR1 | 3 |
| MF | GO:0046943 | carboxylic acid transmembrane transporter activity | 7/248 | 0.005576753 | 0.039630866 | 0.030999672 | SLC7A7/SLC36A1/ABCG2/SLC7A2/ABCB1/AQP9/SLCO1A2 | 7 |
| MF | GO:0001540 | amyloid-beta binding | 5/248 | 0.005637512 | 0.039630866 | 0.030999672 | ITGAM/EPHA4/LILRB2/ITGA2/BCHE | 5 |
| MF | GO:0048306 | calcium-dependent protein binding | 5/248 | 0.005637512 | 0.039630866 | 0.030999672 | S100A9/S100A4/S100A8/S100A12/SELP | 5 |
| MF | GO:0005342 | organic acid transmembrane transporter activity | 7/248 | 0.005769866 | 0.039964807 | 0.031260884 | SLC7A7/SLC36A1/ABCG2/SLC7A2/ABCB1/AQP9/SLCO1A2 | 7 |
| MF | GO:0016209 | antioxidant activity | 5/248 | 0.006223246 | 0.041377151 | 0.032365634 | HBB/HBA2/S100A9/NQO1/PTGS2 | 5 |
| MF | GO:0001968 | fibronectin binding | 3/248 | 0.00623732 | 0.041377151 | 0.032365634 | SFRP2/CCDC80/ITGB3 | 3 |
| MF | GO:0033612 | receptor serine/threonine kinase binding | 3/248 | 0.00623732 | 0.041377151 | 0.032365634 | INHBA/BMP6/BMP5 | 3 |
| MF | GO:0005520 | insulin-like growth factor binding | 3/248 | 0.00688842 | 0.045061751 | 0.035247766 | CCN5/ESM1/ITGB3 | 3 |
| MF | GO:0033218 | amide binding | 12/248 | 0.00717449 | 0.0462902 | 0.036208672 | INHBA/ITGAM/CD14/ACADL/MME/EPHA4/LILRB2/ITGA2/BCHE/ANPEP/VIPR1/ERAP2 | 12 |
| MF | GO:0016634 | oxidoreductase activity, acting on the CH-CH group of donors, oxygen as acceptor | 2/248 | 0.007631878 | 0.047928193 | 0.037489926 | ACADL/ACOXL | 2 |
| MF | GO:0031720 | haptoglobin binding | 2/248 | 0.007631878 | 0.047928193 | 0.037489926 | HBB/HBA2 | 2 |
| MF | GO:0042626 | ATPase-coupled transmembrane transporter activity | 4/248 | 0.007825434 | 0.048497097 | 0.037934929 | ABCC9/ABCG2/ABCA8/ABCB1 | 4 |
| MF | GO:0016298 | lipase activity | 6/248 | 0.008539424 | 0.052234656 | 0.040858486 | PLCB4/PLCB1/ENPP2/CCR1/HMOX1/CCL5 | 6 |
| MF | GO:0004714 | transmembrane receptor protein tyrosine kinase activity | 4/248 | 0.009328336 | 0.055615776 | 0.043503233 | EPHA3/KIT/FGFR2/EPHA4 | 4 |
| MF | GO:0015399 | primary active transmembrane transporter activity | 4/248 | 0.009328336 | 0.055615776 | 0.043503233 | ABCC9/ABCG2/ABCA8/ABCB1 | 4 |
| MF | GO:0004867 | serine-type endopeptidase inhibitor activity | 5/248 | 0.01065888 | 0.062754154 | 0.049086946 | OVOS2/TFPI2/SERPINE2/COL6A3/SERPINA3 | 5 |
| MF | GO:0035325 | Toll-like receptor binding | 2/248 | 0.010995923 | 0.063226648 | 0.049456535 | S100A9/S100A8 | 2 |
| MF | GO:0016836 | hydro-lyase activity | 4/248 | 0.011007612 | 0.063226648 | 0.049456535 | CBS/CA4/CA2/CA12 | 4 |
