# Supplementary material for: FAM171B as a Novel Biomarker Mediates Tissue Immune Microenvironment in Pulmonary Arterial Hypertension
Source: Mediators Inflamm. 2022 Sep 22;2022:1878766. doi: 10.1155/2022/1878766 (PMC9553458; doi:10.1155/2022/1878766)
Supplement: Supplementary Materials — Supplementary Table 1: The results of differentially expressed genes (DEGs). Supplementary Table 2: Gene Ontology (GO) enrichment analysis results of differentially expressed genes (DEGs). Supplementary Table 3: Kyoto Encyclopedia of Genes and Genomes (KEGG) enrichment analysis results of differentially expressed genes (DEGs). Supplementary Table 4: Disease Ontology (DO) enrichment analysis results of differentially expressed genes (DEGs). Supplementary Table 5: Metascape function analysis results of differentially expressed genes (DEGs). Supplementary Table 6: results of Gene Set Enrichment Analysis (GSEA) of gene expression matrix. Supplementary Table 7: results of all genes in brown module. Supplementary Table 8: results of key genes in brown module. Supplementary Table 9: results of analyzing the combined data matrix of GSE113439 and GSE117261 using CIBERSORT. Supplementary Table 10: results of the correlation of FAM171B with immune cells. [file 1878766.f1.zip › Supplementary Table3.docx]

| ID | Description | GeneRatio | pvalue | p.adjust | qvalue | geneID | Count |
| --- | --- | --- | --- | --- | --- | --- | --- |
| hsa04512 | ECM-receptor interaction | 9/65 | 2.66E-08 | 3.09E-06 | 2.49E-06 | COL1A1/THBS2/COL6A3/COL1A2/FN1/SPP1/THBS4/COMP/COL2A1 | 9 |
| hsa04971 | Gastric acid secretion | 8/65 | 1.34E-07 | 7.75E-06 | 6.26E-06 | KCNE2/KCNJ16/SST/CCKBR/ATP4A/ATP4B/GAST/CA2 | 8 |
| hsa04974 | Protein digestion and absorption | 7/65 | 1.65E-05 | 0.000636382 | 0.000513956 | COL1A1/COL6A3/COL1A2/COL8A1/CPA2/COL10A1/COL2A1 | 7 |
| hsa04510 | Focal adhesion | 9/65 | 2.82E-05 | 0.000818524 | 0.000661058 | COL1A1/THBS2/COL6A3/COL1A2/FN1/SPP1/THBS4/COMP/COL2A1 | 9 |
| hsa00830 | Retinol metabolism | 5/65 | 0.000199293 | 0.004623588 | 0.003734114 | ADH7/RDH12/UGT2B15/CYP2C18/ADH1C | 5 |
| hsa00982 | Drug metabolism - cytochrome P450 | 5/65 | 0.000260994 | 0.005045882 | 0.004075168 | ADH7/CYP2C19/UGT2B15/ALDH3A1/ADH1C | 5 |
| hsa00980 | Metabolism of xenobiotics by cytochrome P450 | 5/65 | 0.000379536 | 0.006289451 | 0.005079502 | ADH7/UGT2B15/SULT2A1/ALDH3A1/ADH1C | 5 |
| hsa05165 | Human papillomavirus infection | 9/65 | 0.001191366 | 0.016119503 | 0.013018474 | COL1A1/THBS2/COL6A3/COL1A2/FN1/SPP1/THBS4/COMP/COL2A1 | 9 |
| hsa04966 | Collecting duct acid secretion | 3/65 | 0.001250651 | 0.016119503 | 0.013018474 | ATP4A/ATP4B/CA2 | 3 |
| hsa04151 | PI3K-Akt signaling pathway | 9/65 | 0.001899938 | 0.020761609 | 0.016767543 | COL1A1/THBS2/COL6A3/COL1A2/FN1/SPP1/THBS4/COMP/COL2A1 | 9 |
| hsa00010 | Glycolysis / Gluconeogenesis | 4/65 | 0.001968773 | 0.020761609 | 0.016767543 | ADH7/FBP2/ALDH3A1/ADH1C | 4 |
| hsa05204 | Chemical carcinogenesis - DNA adducts | 4/65 | 0.002194186 | 0.021210461 | 0.017130045 | CYP2C19/UGT2B15/SULT2A1/CYP2C18 | 4 |
| hsa00350 | Tyrosine metabolism | 3/65 | 0.002900179 | 0.025878521 | 0.020900076 | ADH7/ALDH3A1/ADH1C | 3 |
| hsa05144 | Malaria | 3/65 | 0.007352643 | 0.059995077 | 0.048453374 | THBS2/THBS4/COMP | 3 |
| hsa00910 | Nitrogen metabolism | 2/65 | 0.007951155 | 0.059995077 | 0.048453374 | CA9/CA2 | 2 |
| hsa04933 | AGE-RAGE signaling pathway in diabetic complications | 4/65 | 0.008275183 | 0.059995077 | 0.048453374 | COL1A1/COL1A2/SERPINE1/FN1 | 4 |
