# Supplementary material for: FAM171B as a Novel Biomarker Mediates Tissue Immune Microenvironment in Pulmonary Arterial Hypertension
Source: Mediators Inflamm. 2022 Sep 22;2022:1878766. doi: 10.1155/2022/1878766 (PMC9553458; doi:10.1155/2022/1878766)
Supplement: Supplementary Materials — Supplementary Table 1: The results of differentially expressed genes (DEGs). Supplementary Table 2: Gene Ontology (GO) enrichment analysis results of differentially expressed genes (DEGs). Supplementary Table 3: Kyoto Encyclopedia of Genes and Genomes (KEGG) enrichment analysis results of differentially expressed genes (DEGs). Supplementary Table 4: Disease Ontology (DO) enrichment analysis results of differentially expressed genes (DEGs). Supplementary Table 5: Metascape function analysis results of differentially expressed genes (DEGs). Supplementary Table 6: results of Gene Set Enrichment Analysis (GSEA) of gene expression matrix. Supplementary Table 7: results of all genes in brown module. Supplementary Table 8: results of key genes in brown module. Supplementary Table 9: results of analyzing the combined data matrix of GSE113439 and GSE117261 using CIBERSORT. Supplementary Table 10: results of the correlation of FAM171B with immune cells. [file 1878766.f1.zip › Supplementary Table4.docx]

| ID | Description | GeneRatio | pvalue | p.adjust | qvalue | geneID | Count |
| --- | --- | --- | --- | --- | --- | --- | --- |
| DOID:1936 | atherosclerosis | 34/182 | 1.88E-13 | 6.48E-11 | 4.10E-11 | PDE4D/S100A9/RORA/RGS5/PDE1A/VCAM1/CFH/PDGFD/S100A8/S100A12/CD14/IGF1/ACE2/SAA1/CXCR2/HGF/ITGA2/CHIT1/FPR1/CXCL12/SELP/CA2/CA12/ANGPT2/HAS2/OLR1/HMOX1/ITGB3/CCL5/PTGS2/CPB2/SELE/EDN1/CD163 | 34 |
| DOID:2348 | arteriosclerotic cardiovascular disease | 34/182 | 2.05E-13 | 6.48E-11 | 4.10E-11 | PDE4D/S100A9/RORA/RGS5/PDE1A/VCAM1/CFH/PDGFD/S100A8/S100A12/CD14/IGF1/ACE2/SAA1/CXCR2/HGF/ITGA2/CHIT1/FPR1/CXCL12/SELP/CA2/CA12/ANGPT2/HAS2/OLR1/HMOX1/ITGB3/CCL5/PTGS2/CPB2/SELE/EDN1/CD163 | 34 |
| DOID:2349 | arteriosclerosis | 34/182 | 5.12E-13 | 1.08E-10 | 6.82E-11 | PDE4D/S100A9/RORA/RGS5/PDE1A/VCAM1/CFH/PDGFD/S100A8/S100A12/CD14/IGF1/ACE2/SAA1/CXCR2/HGF/ITGA2/CHIT1/FPR1/CXCL12/SELP/CA2/CA12/ANGPT2/HAS2/OLR1/HMOX1/ITGB3/CCL5/PTGS2/CPB2/SELE/EDN1/CD163 | 34 |
| DOID:850 | lung disease | 35/182 | 1.40E-09 | 2.21E-07 | 1.40E-07 | HBB/PDE4D/S100A9/RNASE2/VCAM1/ITGAM/MYH10/CCL21/CD14/IGF1/ITGB6/C3AR1/ACE2/C5/TRPC6/CXCR1/ITK/HGF/CXCL12/MS4A2/F11/HCK/CR1/IL13RA2/ANGPT2/AREG/SERPINE2/HMOX1/CXCL9/ITGB3/MUC5B/CCL5/PTGS2/EDN1/IDO1 | 35 |
| DOID:18 | urinary system disease | 33/182 | 6.68E-09 | 8.45E-07 | 5.35E-07 | PDE4D/POSTN/ADRA1A/AHI1/S100A9/STAT4/VCAM1/CFH/PDGFD/LCN2/ITGAM/S100A8/IGF1/C3AR1/ACE2/KIT/CXCR2/TRPC6/MME/HGF/ABCB1/CA2/CR1/ANGPT2/HMOX1/ROBO2/CCL5/PTGS2/SELE/EDN1/CD163/ANKRD1/IDO1 | 33 |
| DOID:557 | kidney disease | 32/182 | 1.03E-08 | 1.09E-06 | 6.88E-07 | PDE4D/POSTN/ADRA1A/AHI1/S100A9/STAT4/VCAM1/CFH/PDGFD/LCN2/ITGAM/S100A8/IGF1/C3AR1/ACE2/KIT/CXCR2/TRPC6/MME/HGF/ABCB1/CA2/CR1/ANGPT2/HMOX1/CCL5/PTGS2/SELE/EDN1/CD163/ANKRD1/IDO1 | 32 |
| DOID:2320 | obstructive lung disease | 25/182 | 2.47E-08 | 2.23E-06 | 1.41E-06 | HBB/PDE4D/RNASE2/VCAM1/ITGAM/CD14/ITGB6/C3AR1/C5/TRPC6/ITK/HGF/MS4A2/F11/HCK/ANGPT2/AREG/SERPINE2/HMOX1/ITGB3/MUC5B/CCL5/PTGS2/EDN1/IDO1 | 25 |
| DOID:5844 | myocardial infarction | 23/182 | 7.11E-08 | 5.62E-06 | 3.56E-06 | POSTN/S100A9/ABCC9/VCAM1/CFH/INHBA/LCN2/CD14/IGF1/HGF/ITGA2/CXCL12/SELP/F11/OLR1/HMOX1/ITGB3/PTGS2/CPB2/SELE/THBS2/EDN1/CD163 | 23 |
| DOID:9408 | acute myocardial infarction | 13/182 | 1.31E-07 | 9.21E-06 | 5.83E-06 | POSTN/INHBA/CD14/IGF1/ITGA2/SELP/F11/OLR1/HMOX1/ITGB3/PTGS2/CPB2/EDN1 | 13 |
| DOID:3393 | coronary artery disease | 25/182 | 2.69E-07 | 1.70E-05 | 1.08E-05 | POSTN/S100A9/ABCC9/VCAM1/CFH/INHBA/LCN2/NQO1/CD14/IGF1/HGF/ITGA2/FABP4/CXCL12/SELP/F11/OLR1/HMOX1/ITGB3/PTGS2/CPB2/SELE/THBS2/EDN1/CD163 | 25 |
| DOID:3083 | chronic obstructive pulmonary disease | 19/182 | 5.20E-07 | 2.83E-05 | 1.79E-05 | HBB/PDE4D/VCAM1/ITGAM/CD14/ITGB6/C3AR1/C5/TRPC6/HGF/F11/HCK/ANGPT2/SERPINE2/HMOX1/MUC5B/CCL5/PTGS2/EDN1 | 19 |
| DOID:2462 | retinal vascular disease | 10/182 | 5.83E-07 | 2.83E-05 | 1.79E-05 | POSTN/VCAM1/IGF1/KIT/MME/ITGA2/CXCL12/CCL5/SELE/EDN1 | 10 |
| DOID:8947 | diabetic retinopathy | 10/182 | 5.83E-07 | 2.83E-05 | 1.79E-05 | POSTN/VCAM1/IGF1/KIT/MME/ITGA2/CXCL12/CCL5/SELE/EDN1 | 10 |
| DOID:5295 | intestinal disease | 15/182 | 2.49E-06 | 0.000110174 | 6.97E-05 | VCAM1/S100A8/S100A12/CCL21/GFRA1/KIT/CA2/BIRC3/AQP9/CXCL9/CCL5/PTGS2/SELE/EDN1/IDO1 | 15 |
| DOID:74 | hematopoietic system disease | 28/182 | 2.61E-06 | 0.000110174 | 6.97E-05 | CSF3R/HBB/HBA2/RNASE2/VCAM1/CFH/LCN2/NQO1/ITGAM/TCN2/ALAS2/IGF1/KIT/CXCR1/HGF/ITGA2/SELP/CA2/F11/CR1/BIRC3/ANGPT2/EGR1/HMOX1/ITGB3/CCL5/CPB2/EDN1 | 28 |
| DOID:12365 | malaria | 12/182 | 3.34E-06 | 0.000131832 | 8.34E-05 | HBB/TCN2/ACE2/SAA1/CHIT1/CR1/ANGPT2/HMOX1/CXCL9/CCL5/PTGS2/EDN1 | 12 |
| DOID:0070004 | myeloma | 22/182 | 3.62E-06 | 0.000134524 | 8.51E-05 | VCAM1/SFRP2/INHBA/LCN2/NQO1/ABCG2/BMP6/IGF1/KIT/CXCR2/CXCR1/HGF/CXCL12/SELP/ABCB1/HCK/AREG/EGR1/SFRP4/ITGB3/PTGS2/CPB2 | 22 |
| DOID:3908 | non-small cell lung carcinoma | 26/182 | 4.25E-06 | 0.000149166 | 9.44E-05 | POSTN/S100A9/MACC1/S100A4/LUM/INHBA/CNTN1/WIF1/NQO1/ITGAM/S100A8/ABCG2/CD14/IGF1/KIT/SAA1/CXCR2/WEE1/HGF/CXCL12/ABCB1/ANPEP/TFPI2/HMOX1/CCL5/PTGS2 | 26 |
| DOID:4960 | bone marrow cancer | 22/182 | 4.65E-06 | 0.000154625 | 9.79E-05 | VCAM1/SFRP2/INHBA/LCN2/NQO1/ABCG2/BMP6/IGF1/KIT/CXCR2/CXCR1/HGF/CXCL12/SELP/ABCB1/HCK/AREG/EGR1/SFRP4/ITGB3/PTGS2/CPB2 | 22 |
| DOID:10952 | nephritis | 14/182 | 5.67E-06 | 0.00017916 | 0.000113392 | VCAM1/CFH/PDGFD/LCN2/C3AR1/KIT/TRPC6/MME/HGF/CR1/CCL5/PTGS2/SELE/ANKRD1 | 14 |
| DOID:13207 | proliferative diabetic retinopathy | 7/182 | 1.10E-05 | 0.000329952 | 0.000208831 | POSTN/VCAM1/KIT/MME/CXCL12/SELE/EDN1 | 7 |
| DOID:1247 | blood coagulation disease | 15/182 | 1.34E-05 | 0.000386147 | 0.000244397 | CFH/LCN2/ITGAM/IGF1/HGF/ITGA2/SELP/F11/CR1/ANGPT2/HMOX1/ITGB3/CCL5/CPB2/EDN1 | 15 |
| DOID:10591 | pre-eclampsia | 19/182 | 1.67E-05 | 0.000457621 | 0.000289633 | VCAM1/CFH/INHBA/LCN2/ENPP2/IGF1/FABP4/SELP/ANGPT2/TFPI2/AQP9/OLR1/HMOX1/ERAP2/PTGS2/CPB2/SELE/EDN1/IDO1 | 19 |
| DOID:865 | vasculitis | 12/182 | 1.95E-05 | 0.000512353 | 0.000324274 | S100A9/STAT4/CCL21/C5/ITGA2/CHIT1/CXCL12/HMOX1/ITGB3/TNFAIP3/CPB2/SELE | 12 |
| DOID:2789 | parasitic protozoa infectious disease | 12/182 | 2.92E-05 | 0.000738086 | 0.000467143 | HBB/TCN2/ACE2/SAA1/CHIT1/CR1/ANGPT2/HMOX1/CXCL9/CCL5/PTGS2/EDN1 | 12 |
| DOID:2237 | hepatitis | 24/182 | 3.87E-05 | 0.000941707 | 0.000596017 | HBB/VCAM1/LCN2/PHGDH/ENPP2/CCL21/CD14/IGF1/SAA1/CXCR1/IL1R2/HGF/ITGA2/CXCL12/CCR1/VSIG4/ANGPT2/VIPR1/HMOX1/CXCL9/CCL5/FAP/PTGS2/IDO1 | 24 |
| DOID:9008 | psoriatic arthritis | 4/182 | 4.87E-05 | 0.001121487 | 0.000709802 | S100A9/S100A8/S100A12/AREG | 4 |
| DOID:3910 | lung adenocarcinoma | 14/182 | 5.01E-05 | 0.001121487 | 0.000709802 | S100A9/MACC1/S100A4/LUM/INHBA/CNTN1/NQO1/S100A8/SAA1/HGF/CXCL12/HMOX1/CCL5/PTGS2 | 14 |
| DOID:1074 | kidney failure | 13/182 | 5.15E-05 | 0.001121487 | 0.000709802 | ADRA1A/S100A9/CFH/LCN2/ITGAM/S100A8/TRPC6/HGF/ABCB1/CCL5/SELE/EDN1/CD163 | 13 |
| DOID:2921 | glomerulonephritis | 9/182 | 6.95E-05 | 0.001402318 | 0.000887543 | VCAM1/CFH/LCN2/KIT/TRPC6/MME/CR1/SELE/ANKRD1 | 9 |
| DOID:3388 | periodontal disease | 12/182 | 7.14E-05 | 0.001402318 | 0.000887543 | S100A8/CD14/C5/CXCR2/IL1R2/HGF/FPR1/ABCB1/CCL5/PTGS2/SELE/EDN1 | 12 |
| DOID:2089 | constipation | 4/182 | 7.52E-05 | 0.001402318 | 0.000887543 | S100A8/KIT/AQP9/PTGS2 | 4 |
| DOID:9779 | bowel dysfunction | 4/182 | 7.52E-05 | 0.001402318 | 0.000887543 | S100A8/KIT/AQP9/PTGS2 | 4 |
| DOID:1883 | hepatitis C | 16/182 | 7.54E-05 | 0.001402318 | 0.000887543 | HBB/LCN2/ENPP2/CCL21/CD14/IGF1/SAA1/IL1R2/ITGA2/CCR1/VIPR1/HMOX1/CCL5/FAP/PTGS2/IDO1 | 16 |
| DOID:9970 | obesity | 19/182 | 8.50E-05 | 0.001534506 | 0.000971206 | S100A9/RORA/VCAM1/LCN2/NQO1/S100A8/ENPP2/IGF1/SAA1/HGF/BCHE/FABP4/SELP/ABCB1/ITGAX/AQP9/OLR1/SELE/EDN1 | 19 |
| DOID:345 | uterine disease | 7/182 | 9.36E-05 | 0.001642356 | 0.001039466 | IGF1/CXCR1/ANGPT2/AQP9/PTGS2/SELE/EDN1 | 7 |
| DOID:9538 | multiple myeloma | 17/182 | 9.96E-05 | 0.001700989 | 0.001076575 | VCAM1/SFRP2/INHBA/NQO1/ABCG2/IGF1/KIT/HGF/CXCL12/SELP/ABCB1/HCK/AREG/EGR1/SFRP4/ITGB3/PTGS2 | 17 |
| DOID:3770 | pulmonary fibrosis | 11/182 | 0.000104436 | 0.001736938 | 0.001099328 | HBB/S100A9/CCL21/IGF1/CXCL12/AREG/HMOX1/MUC5B/CCL5/PTGS2/EDN1 | 11 |
| DOID:1793 | pancreatic cancer | 19/182 | 0.000109362 | 0.001772231 | 0.001121665 | POSTN/S100A4/LUM/PDGFD/LCN2/NQO1/CCN5/IGF1/ACE2/KIT/MME/EPHA4/HGF/ITGA2/CXCL12/CA2/TFPI2/FAP/PTGS2 | 19 |
| DOID:654 | overnutrition | 19/182 | 0.000123729 | 0.001954917 | 0.001237289 | S100A9/RORA/VCAM1/LCN2/NQO1/S100A8/ENPP2/IGF1/SAA1/HGF/BCHE/FABP4/SELP/ABCB1/ITGAX/AQP9/OLR1/SELE/EDN1 | 19 |
| DOID:1398 | parasitic infectious disease | 12/182 | 0.000148647 | 0.002291346 | 0.001450219 | HBB/TCN2/ACE2/SAA1/CHIT1/CR1/ANGPT2/HMOX1/CXCL9/CCL5/PTGS2/EDN1 | 12 |
| DOID:1176 | bronchial disease | 12/182 | 0.000179306 | 0.002698128 | 0.001707676 | S100A9/RNASE2/CD14/CXCR1/ITK/MS4A2/AREG/ITGB3/CCL5/PTGS2/EDN1/IDO1 | 12 |
| DOID:374 | nutrition disease | 19/182 | 0.000232197 | 0.003412763 | 0.002159976 | S100A9/RORA/VCAM1/LCN2/NQO1/S100A8/ENPP2/IGF1/SAA1/HGF/BCHE/FABP4/SELP/ABCB1/ITGAX/AQP9/OLR1/SELE/EDN1 | 19 |
| DOID:10283 | prostate cancer | 22/182 | 0.000239994 | 0.003447182 | 0.002181761 | S100A9/WIF1/PDGFD/S100A8/BMP6/IGF1/MME/HGF/ITGA2/BCHE/CXCL12/BIRC3/ADAMTS9/AREG/AKR1C2/EGR1/SFRP4/ITGB3/CCL5/SERPINA3/PTGS2/MSMB | 22 |
| DOID:8432 | polycythemia | 6/182 | 0.000294667 | 0.00413843 | 0.002619259 | LCN2/IGF1/KIT/HGF/SELP/EDN1 | 6 |
| DOID:1091 | tooth disease | 12/182 | 0.000305714 | 0.004200248 | 0.002658385 | S100A8/CD14/C5/CXCR2/IL1R2/HGF/FPR1/ABCB1/CCL5/PTGS2/SELE/EDN1 | 12 |
| DOID:8398 | osteoarthritis | 13/182 | 0.000320841 | 0.004271405 | 0.002703421 | SULF1/LCN2/ASPN/ITGAM/CCL21/CXCL12/BMP5/HMOX1/TIMP4/CCL5/TNFAIP3/FAP/EDN1 | 13 |
| DOID:3856 | male reproductive organ cancer | 22/182 | 0.000332459 | 0.004271405 | 0.002703421 | S100A9/WIF1/PDGFD/S100A8/BMP6/IGF1/MME/HGF/ITGA2/BCHE/CXCL12/BIRC3/ADAMTS9/AREG/AKR1C2/EGR1/SFRP4/ITGB3/CCL5/SERPINA3/PTGS2/MSMB | 22 |
| DOID:10923 | sickle cell anemia | 6/182 | 0.000337369 | 0.004271405 | 0.002703421 | HBB/HBA2/VCAM1/IGF1/SELP/PTGS2 | 6 |
| DOID:403 | mouth disease | 13/182 | 0.000337928 | 0.004271405 | 0.002703421 | LCN2/S100A8/CD14/C5/CXCR2/IL1R2/HGF/FPR1/ABCB1/CCL5/PTGS2/SELE/EDN1 | 13 |
| DOID:0060100 | musculoskeletal system cancer | 22/182 | 0.000377438 | 0.004676435 | 0.002959769 | EPHA3/S100A4/LUM/INHBA/WIF1/ENPP2/CD14/IGF1/KIT/FGFR2/HGF/ITGA2/CXCL12/ABCB1/TFPI2/HAS2/ITGB3/COL6A3/CCL5/PTGS2/CD163/ANKRD1 | 22 |
| DOID:4961 | bone marrow disease | 6/182 | 0.00038477 | 0.004676435 | 0.002959769 | LCN2/IGF1/KIT/HGF/SELP/EDN1 | 6 |
| DOID:2355 | anemia | 15/182 | 0.000424958 | 0.004928997 | 0.003119618 | CSF3R/HBB/HBA2/VCAM1/CFH/LCN2/NQO1/TCN2/ALAS2/IGF1/CA2/CR1/EGR1/ITGB3/CCL5 | 15 |
| DOID:1037 | lymphoblastic leukemia | 22/182 | 0.000427675 | 0.004928997 | 0.003119618 | PDE4D/PDE7B/NT5E/EPHA3/ARID5B/NQO1/XAF1/ITGAM/ABCG2/CCL21/CD14/MME/CXCR1/HGF/CXCL12/ABCB1/ANPEP/ITGAX/BIRC3/ANGPT2/TNFAIP3/PTGS2 | 22 |
| DOID:104 | bacterial infectious disease | 16/182 | 0.000428947 | 0.004928997 | 0.003119618 | CFH/LCN2/ITGAM/PHGDH/CD14/IGF1/SAA1/CXCR2/BPIFB1/CHIT1/ABCB1/CR1/ITGAX/CCL5/PTGS2/IDO1 | 16 |
| DOID:5082 | liver cirrhosis | 14/182 | 0.000468352 | 0.005129992 | 0.003246831 | MATN2/VCAM1/CD14/IGF1/WEE1/HGF/CXCL12/SELP/CCR1/VIPR1/CXCL9/SERPINA3/CPB2/IDO1 | 14 |
| DOID:3082 | interstitial lung disease | 12/182 | 0.00047507 | 0.005129992 | 0.003246831 | HBB/S100A9/CCL21/IGF1/ACE2/CXCL12/AREG/HMOX1/MUC5B/CCL5/PTGS2/EDN1 | 12 |
| DOID:9074 | systemic lupus erythematosus | 8/182 | 0.00047695 | 0.005129992 | 0.003246831 | LCN2/ITGAM/CXCR2/CXCL12/SELP/ITGB3/CCL5/SELE | 8 |
| DOID:5679 | retinal disease | 19/182 | 0.000478908 | 0.005129992 | 0.003246831 | POSTN/AHI1/RORA/HMCN1/VCAM1/CFH/CA4/IGF1/C5/KIT/FGFR2/MME/ITGA2/CXCL12/SELP/CA12/CCL5/SELE/EDN1 | 19 |
| DOID:1040 | chronic lymphocytic leukemia | 14/182 | 0.000490224 | 0.005163694 | 0.003268161 | PDE4D/PDE7B/NT5E/XAF1/CCL21/CD14/MME/CXCL12/ANPEP/ITGAX/BIRC3/ANGPT2/TNFAIP3/PTGS2 | 14 |
| DOID:5353 | colonic disease | 5/182 | 0.000515724 | 0.005343237 | 0.003381796 | S100A8/GFRA1/KIT/AQP9/PTGS2 | 5 |
| DOID:11335 | sarcoidosis | 8/182 | 0.00061013 | 0.006219387 | 0.003936321 | S100A9/S100A8/CD14/ACE2/CHIT1/CR1/PTGS2/SELE | 8 |
| DOID:2213 | hemorrhagic disease | 11/182 | 0.00066326 | 0.006551912 | 0.00414678 | CFH/LCN2/IGF1/HGF/ITGA2/SELP/HMOX1/ITGB3/CCL5/CPB2/EDN1 | 11 |
| DOID:219 | colon cancer | 16/182 | 0.000663485 | 0.006551912 | 0.00414678 | POSTN/PDCD4/LCN2/IGF1/ITGB6/ITGA2/BCHE/CXCL12/SELP/ABCB1/BIRC3/HAS2/HMOX1/PTGS2/OLFM4/SELE | 16 |
| DOID:13375 | temporal arteritis | 5/182 | 0.000702462 | 0.006665655 | 0.004218769 | STAT4/C5/ITGB3/TNFAIP3/SELE | 5 |
| DOID:525 | central nervous system vasculitis | 5/182 | 0.000702462 | 0.006665655 | 0.004218769 | STAT4/C5/ITGB3/TNFAIP3/SELE | 5 |
| DOID:4138 | bile duct disease | 8/182 | 0.000714529 | 0.006665655 | 0.004218769 | CD14/ITGB6/MME/HGF/HMOX1/CCL5/SELE/EDN1 | 8 |
| DOID:201 | connective tissue cancer | 19/182 | 0.000717191 | 0.006665655 | 0.004218769 | S100A4/LUM/INHBA/WIF1/ENPP2/CD14/IGF1/KIT/FGFR2/HGF/ITGA2/CXCL12/ABCB1/TFPI2/HAS2/ITGB3/COL6A3/CCL5/PTGS2 | 19 |
| DOID:326 | ischemia | 13/182 | 0.00073692 | 0.00674976 | 0.004272 | MFGE8/ADORA3/MYH10/C5/KIT/HGF/CXCL12/SELP/ANGPT2/SERPINA3/PTGS2/SELE/EDN1 | 13 |
| DOID:8857 | lupus erythematosus | 8/182 | 0.000771876 | 0.006870786 | 0.004348599 | LCN2/ITGAM/CXCR2/CXCL12/SELP/ITGB3/CCL5/SELE | 8 |
| DOID:9741 | biliary tract disease | 8/182 | 0.000771876 | 0.006870786 | 0.004348599 | CD14/ITGB6/MME/HGF/HMOX1/CCL5/SELE/EDN1 | 8 |
| DOID:9256 | colorectal cancer | 16/182 | 0.000802033 | 0.00704007 | 0.00445574 | POSTN/PDCD4/LCN2/IGF1/ITGB6/ITGA2/BCHE/CXCL12/SELP/ABCB1/BIRC3/HAS2/HMOX1/PTGS2/OLFM4/SELE | 16 |
| DOID:5672 | large intestine cancer | 16/182 | 0.000832522 | 0.00711317 | 0.004502007 | POSTN/PDCD4/LCN2/IGF1/ITGB6/ITGA2/BCHE/CXCL12/SELP/ABCB1/BIRC3/HAS2/HMOX1/PTGS2/OLFM4/SELE | 16 |
| DOID:2916 | hypersensitivity reaction type IV disease | 8/182 | 0.000832871 | 0.00711317 | 0.004502007 | S100A9/S100A8/CD14/ACE2/CHIT1/CR1/PTGS2/SELE | 8 |
| DOID:299 | adenocarcinoma | 11/182 | 0.000915699 | 0.007631049 | 0.004829778 | S100A4/INHBA/S100A8/IGF1/KIT/ITGA2/SELP/THY1/ITGB3/PTGS2/SELE | 11 |
| DOID:2841 | asthma | 10/182 | 0.000917658 | 0.007631049 | 0.004829778 | RNASE2/CD14/ITK/MS4A2/AREG/ITGB3/CCL5/PTGS2/EDN1/IDO1 | 10 |
| DOID:50 | thyroid gland disease | 11/182 | 0.00096465 | 0.007917645 | 0.005011168 | IGF1/KIT/HGF/ABCB1/PDE8B/CXCL9/CCL5/PTGS2/CPB2/SELE/EDN1 | 11 |
| DOID:0050338 | primary bacterial infectious disease | 14/182 | 0.00101892 | 0.008255864 | 0.005225231 | LCN2/PHGDH/CD14/IGF1/SAA1/CXCR2/BPIFB1/CHIT1/ABCB1/CR1/ITGAX/CCL5/PTGS2/IDO1 | 14 |
| DOID:4074 | pancreas adenocarcinoma | 11/182 | 0.001069064 | 0.00855251 | 0.005412981 | LCN2/NQO1/CCN5/IGF1/ACE2/KIT/EPHA4/HGF/CXCL12/FAP/PTGS2 | 11 |
| DOID:0060084 | cell type benign neoplasm | 22/182 | 0.001088635 | 0.008600214 | 0.005443173 | SLC9A3R2/S100A4/SFRP2/WIF1/OGN/NQO1/MALL/IGF1/FGFR2/MME/HGF/ITGA2/SLC18A2/CXCL12/ABCB1/BIRC3/EGR1/SFRP4/HMOX1/PTGS2/THBS2/EDN1 | 22 |
| DOID:10155 | intestinal cancer | 16/182 | 0.001282714 | 0.010008335 | 0.006334389 | POSTN/PDCD4/LCN2/IGF1/ITGB6/ITGA2/BCHE/CXCL12/SELP/ABCB1/BIRC3/HAS2/HMOX1/PTGS2/OLFM4/SELE | 16 |
| DOID:2377 | multiple sclerosis | 11/182 | 0.001584564 | 0.01211904 | 0.007670279 | CD14/CXCR2/C7/CHIT1/BCHE/CXCL12/CCR1/BIRC3/HMOX1/CCL5/IDO1 | 11 |
| DOID:824 | periodontitis | 9/182 | 0.001591583 | 0.01211904 | 0.007670279 | S100A8/CD14/C5/CXCR2/IL1R2/FPR1/CCL5/PTGS2/SELE | 9 |
| DOID:6432 | pulmonary hypertension | 7/182 | 0.001757315 | 0.013221706 | 0.008368169 | INHBA/TRPC6/CXCL12/SELP/HMOX1/PTGS2/EDN1 | 7 |
| DOID:10286 | prostate carcinoma | 9/182 | 0.00178589 | 0.013278614 | 0.008404186 | S100A9/WIF1/PDGFD/S100A8/IGF1/HGF/EGR1/SERPINA3/PTGS2 | 9 |
| DOID:2452 | thrombophilia | 4/182 | 0.001921995 | 0.013691523 | 0.008665521 | ITGAM/HGF/ANGPT2/CPB2 | 4 |
| DOID:6543 | acne | 4/182 | 0.001921995 | 0.013691523 | 0.008665521 | LCN2/IGF1/MME/ANPEP | 4 |
| DOID:9098 | sebaceous gland disease | 4/182 | 0.001921995 | 0.013691523 | 0.008665521 | LCN2/IGF1/MME/ANPEP | 4 |
| DOID:3451 | skin carcinoma | 6/182 | 0.001928078 | 0.013691523 | 0.008665521 | S100A9/IGF1/CXCL12/AREG/CCL5/PTGS2 | 6 |
| DOID:854 | collagen disease | 12/182 | 0.00205052 | 0.014352443 | 0.009083825 | STAT4/CFH/ENPP2/IGF1/HGF/SELP/IL13RA2/BIRC3/TIMP4/COL15A1/TNFAIP3/EDN1 | 12 |
| DOID:3213 | demyelinating disease | 11/182 | 0.002092161 | 0.014352443 | 0.009083825 | CD14/CXCR2/C7/CHIT1/BCHE/CXCL12/CCR1/BIRC3/HMOX1/CCL5/IDO1 | 11 |
| DOID:3996 | urinary system cancer | 22/182 | 0.002097886 | 0.014352443 | 0.009083825 | POSTN/SFRP2/WIF1/XAF1/BMP6/ESM1/IGF1/KIT/FGFR2/MME/HGF/SOSTDC1/FABP4/CXCL12/ABCB1/FAM107A/BIRC3/ANGPT2/TFPI2/EGR1/PTGS2/EDN1 | 22 |
| DOID:6000 | congestive heart failure | 13/182 | 0.002111989 | 0.014352443 | 0.009083825 | ADRA1A/VCAM1/ZFPM2/LCN2/CD14/IGF1/ACE2/TRPC6/HGF/HMOX1/TIMP4/EDN1/ANKRD1 | 13 |
| DOID:4451 | renal carcinoma | 18/182 | 0.002447463 | 0.016455286 | 0.010414738 | SFRP2/WIF1/XAF1/BMP6/ESM1/IGF1/KIT/FGFR2/MME/HGF/SOSTDC1/CXCL12/ABCB1/FAM107A/ANGPT2/TFPI2/PTGS2/EDN1 | 18 |
| DOID:341 | peripheral vascular disease | 6/182 | 0.00250076 | 0.016636635 | 0.010529516 | VCAM1/IGF1/HGF/ITGA2/SELE/EDN1 | 6 |
| DOID:2214 | inherited blood coagulation disease | 4/182 | 0.002610057 | 0.017182875 | 0.010875237 | ITGA2/F11/CR1/ITGB3 | 4 |
| DOID:13580 | cholestasis | 6/182 | 0.002717467 | 0.01770556 | 0.011206051 | CD14/ITGB6/MME/HMOX1/SELE/EDN1 | 6 |
| DOID:3086 | gingival overgrowth | 3/182 | 0.002792741 | 0.018010327 | 0.011398941 | HGF/ABCB1/EDN1 | 3 |
| DOID:3371 | chondrosarcoma | 6/182 | 0.002947962 | 0.018819315 | 0.011910959 | INHBA/FGFR2/ITGA2/ABCB1/ITGB3/PTGS2 | 6 |
| DOID:2513 | basal cell carcinoma | 4/182 | 0.003010285 | 0.018836634 | 0.01192192 | S100A9/CXCL12/CCL5/PTGS2 | 4 |
| DOID:780 | placenta disease | 4/182 | 0.003010285 | 0.018836634 | 0.01192192 | IGF1/ANGPT2/AQP9/EDN1 | 4 |
| DOID:552 | pneumonia | 8/182 | 0.003106355 | 0.019247222 | 0.012181786 | CCL21/CD14/CXCL12/CR1/IL13RA2/HMOX1/MUC5B/PTGS2 | 8 |
| DOID:11830 | myopia | 5/182 | 0.003358912 | 0.020610023 | 0.013044318 | LUM/CFH/IGF1/HGF/EGR1 | 5 |
| DOID:655 | inherited metabolic disorder | 16/182 | 0.00347727 | 0.020782039 | 0.013153189 | HBB/ABCC9/VCAM1/LUM/CBS/ABCG2/ALAS2/IGF1/KIT/CHIT1/FABP4/CXCL12/IL13RA2/HMOX1/CCL5/SELE | 16 |
| DOID:12241 | beta thalassemia | 3/182 | 0.003495411 | 0.020782039 | 0.013153189 | HBB/LCN2/IGF1 | 3 |
| DOID:184 | bone cancer | 13/182 | 0.003537865 | 0.020782039 | 0.013153189 | S100A4/LUM/WIF1/IGF1/KIT/FGFR2/HGF/CXCL12/ABCB1/HAS2/ITGB3/CCL5/PTGS2 | 13 |
| DOID:3347 | osteosarcoma | 12/182 | 0.00357267 | 0.020782039 | 0.013153189 | S100A4/LUM/WIF1/IGF1/KIT/FGFR2/HGF/CXCL12/HAS2/ITGB3/CCL5/PTGS2 | 12 |
| DOID:9352 | type 2 diabetes mellitus | 12/182 | 0.00357267 | 0.020782039 | 0.013153189 | S100A9/ITGAM/S100A8/BMP6/IGF1/MME/BCHE/CXCL12/SELP/ITGB3/CCL5/SELE | 12 |
| DOID:1575 | rheumatic disease | 11/182 | 0.00365001 | 0.020782039 | 0.013153189 | STAT4/CFH/ENPP2/IGF1/HGF/SELP/IL13RA2/TIMP4/COL15A1/TNFAIP3/EDN1 | 11 |
| DOID:418 | systemic scleroderma | 11/182 | 0.00365001 | 0.020782039 | 0.013153189 | STAT4/CFH/ENPP2/IGF1/HGF/SELP/IL13RA2/TIMP4/COL15A1/TNFAIP3/EDN1 | 11 |
| DOID:419 | scleroderma | 11/182 | 0.00365001 | 0.020782039 | 0.013153189 | STAT4/CFH/ENPP2/IGF1/HGF/SELP/IL13RA2/TIMP4/COL15A1/TNFAIP3/EDN1 | 11 |
| DOID:750 | peptic ulcer disease | 6/182 | 0.003727549 | 0.021034029 | 0.013312677 | CXCR2/CXCR1/ITGB3/PTGS2/SELE/EDN1 | 6 |
| DOID:11963 | esophagitis | 4/182 | 0.003932537 | 0.021994368 | 0.013920486 | POSTN/CXCR2/CXCR1/IL13RA2 | 4 |
| DOID:10652 | Alzheimer's disease | 19/182 | 0.004039845 | 0.022396336 | 0.014174896 | S100A9/CBS/CFH/ITGAM/S100A12/BMP6/IGF1/CXCR2/MME/SLC18A2/BCHE/ABCB1/PDE8B/CR1/OLR1/HMOX1/CXCL9/SERPINA3/PTGS2 | 19 |
| DOID:28 | endocrine system disease | 18/182 | 0.004118464 | 0.022633647 | 0.014325093 | VCAM1/LCN2/PROK2/CD14/IGF1/KIT/HGF/SELP/ABCB1/CA2/PDE8B/AQP9/CXCL9/CCL5/PTGS2/CPB2/SELE/EDN1 | 18 |
| DOID:0050589 | inflammatory bowel disease | 7/182 | 0.004267162 | 0.023248673 | 0.01471435 | S100A12/CCL21/CA2/BIRC3/CXCL9/CCL5/IDO1 | 7 |
| DOID:680 | tauopathy | 19/182 | 0.004465435 | 0.024120984 | 0.015266445 | S100A9/CBS/CFH/ITGAM/S100A12/BMP6/IGF1/CXCR2/MME/SLC18A2/BCHE/ABCB1/PDE8B/CR1/OLR1/HMOX1/CXCL9/SERPINA3/PTGS2 | 19 |
| DOID:263 | kidney cancer | 19/182 | 0.004808489 | 0.025753939 | 0.016299961 | SFRP2/WIF1/XAF1/BMP6/ESM1/IGF1/KIT/FGFR2/MME/HGF/SOSTDC1/CXCL12/ABCB1/FAM107A/ANGPT2/TFPI2/EGR1/PTGS2/EDN1 | 19 |
| DOID:4971 | myelofibrosis | 5/182 | 0.004873768 | 0.025884214 | 0.016382414 | BMP6/KIT/CXCR2/CXCR1/CXCL12 | 5 |
| DOID:3376 | bone osteosarcoma | 5/182 | 0.005317593 | 0.027960707 | 0.01769665 | LUM/IGF1/KIT/CXCL12/ITGB3 | 5 |
| DOID:3326 | purpura | 6/182 | 0.005353237 | 0.027960707 | 0.01769665 | IGF1/HGF/SELP/HMOX1/ITGB3/EDN1 | 6 |
| DOID:5041 | esophageal cancer | 9/182 | 0.005690815 | 0.029216112 | 0.01849121 | S100A9/S100A4/INHBA/S100A8/IGF1/ADAMTS9/EGR1/PTGS2/SELE | 9 |
| DOID:10871 | age related macular degeneration | 6/182 | 0.005732275 | 0.029216112 | 0.01849121 | RORA/HMCN1/CFH/C5/SELP/SELE | 6 |
| DOID:2007 | degeneration of macula and posterior pole | 6/182 | 0.005732275 | 0.029216112 | 0.01849121 | RORA/HMCN1/CFH/C5/SELP/SELE | 6 |
| DOID:2218 | blood platelet disease | 7/182 | 0.006077661 | 0.030484777 | 0.019294163 | CFH/LCN2/ITGA2/SELP/ITGB3/CCL5/CPB2 | 7 |
| DOID:7998 | hyperthyroidism | 7/182 | 0.006077661 | 0.030484777 | 0.019294163 | IGF1/ABCB1/CXCL9/CCL5/CPB2/SELE/EDN1 | 7 |
| DOID:9415 | allergic asthma | 5/182 | 0.006289724 | 0.030934587 | 0.019578853 | RNASE2/CD14/ITK/MS4A2/EDN1 | 5 |
| DOID:2228 | thrombocytosis | 4/182 | 0.00631418 | 0.030934587 | 0.019578853 | LCN2/SELP/ITGB3/CPB2 | 4 |
| DOID:3355 | fibrosarcoma | 4/182 | 0.00631418 | 0.030934587 | 0.019578853 | ENPP2/KIT/TFPI2/PTGS2 | 4 |
| DOID:4448 | macular degeneration | 6/182 | 0.006548657 | 0.031836547 | 0.020149713 | RORA/HMCN1/CFH/C5/SELP/SELE | 6 |
| DOID:0060122 | integumentary system cancer | 7/182 | 0.006792911 | 0.032523635 | 0.020584579 | S100A9/IGF1/FGFR2/CXCL12/AREG/CCL5/PTGS2 | 7 |
| DOID:4159 | skin cancer | 7/182 | 0.006792911 | 0.032523635 | 0.020584579 | S100A9/IGF1/FGFR2/CXCL12/AREG/CCL5/PTGS2 | 7 |
| DOID:13250 | diarrhea | 4/182 | 0.007031319 | 0.033411984 | 0.021146825 | S100A8/ABCG2/ABCB1/PTGS2 | 4 |
| DOID:4607 | biliary tract cancer | 10/182 | 0.007271693 | 0.033929332 | 0.021474261 | POSTN/PDCD4/LCN2/NQO1/ABCG2/MME/CXCL12/ABCB1/CR1/PTGS2 | 10 |
| DOID:200 | giant cell tumor | 3/182 | 0.007329887 | 0.033929332 | 0.021474261 | CD14/COL6A3/PTGS2 | 3 |
| DOID:8719 | in situ carcinoma | 3/182 | 0.007329887 | 0.033929332 | 0.021474261 | S100A9/S100A4/PTGS2 | 3 |
| DOID:120 | female reproductive organ cancer | 19/182 | 0.007354934 | 0.033929332 | 0.021474261 | POSTN/RORA/S100A4/INHBA/LCN2/S100A8/ABCG2/IGF1/KIT/MME/CXCL12/ABCB1/THY1/ANPEP/HAS2/ITGB3/PTGS2/EDN1/TSPAN1 | 19 |
| DOID:4905 | pancreatic carcinoma | 12/182 | 0.007695417 | 0.03524278 | 0.022305557 | LCN2/NQO1/CCN5/IGF1/ACE2/KIT/EPHA4/HGF/CXCL12/TFPI2/FAP/PTGS2 | 12 |
| DOID:974 | upper respiratory tract disease | 8/182 | 0.007855688 | 0.03571795 | 0.022606297 | VCAM1/CD14/CXCR2/ITK/SELP/HMOX1/CCL5/EDN1 | 8 |
| DOID:75 | lymphatic system disease | 7/182 | 0.007980737 | 0.036027328 | 0.022802106 | S100A9/S100A8/S100A12/HGF/CHIT1/ITGAX/CD163 | 7 |
| DOID:9455 | lipid storage disease | 8/182 | 0.009413346 | 0.042193155 | 0.026704529 | HBB/VCAM1/LUM/CHIT1/FABP4/IL13RA2/HMOX1/SELE | 8 |
| DOID:633 | myositis | 6/182 | 0.010075369 | 0.044842486 | 0.02838132 | ADRA1A/ITGAM/S100A8/CCL21/IGF1/HGF | 6 |
| DOID:3713 | ovary adenocarcinoma | 4/182 | 0.010432689 | 0.045787915 | 0.028979693 | INHBA/THY1/ITGB3/PTGS2 | 4 |
| DOID:866 | vein disease | 4/182 | 0.010432689 | 0.045787915 | 0.028979693 | NT5E/IGF1/HMOX1/CCL5 | 4 |
| DOID:5940 | malignant peripheral nerve sheath tumor | 3/182 | 0.011365861 | 0.049539475 | 0.031354098 | CCN5/KIT/BMP5 | 3 |
| DOID:2394 | ovarian cancer | 14/182 | 0.011497717 | 0.049770941 | 0.031500595 | POSTN/S100A4/INHBA/LCN2/IGF1/KIT/ABCB1/THY1/ANPEP/HAS2/ITGB3/PTGS2/EDN1/TSPAN1 | 14 |
| DOID:229 | female reproductive system disease | 10/182 | 0.012466207 | 0.052778192 | 0.033403919 | IGF1/CXCR2/CXCR1/CXCL12/ANGPT2/AQP9/CCL5/PTGS2/SELE/EDN1 | 10 |
| DOID:3111 | cystadenocarcinoma | 4/182 | 0.012472122 | 0.052778192 | 0.033403919 | KIT/THY1/ITGB3/PTGS2 | 4 |
| DOID:3114 | serous cystadenocarcinoma | 4/182 | 0.012472122 | 0.052778192 | 0.033403919 | KIT/THY1/ITGB3/PTGS2 | 4 |
| DOID:0060071 | pre-malignant neoplasm | 3/182 | 0.012944019 | 0.052778192 | 0.033403919 | S100A9/S100A4/PTGS2 | 3 |
| DOID:11123 | Henoch-Schoenlein purpura | 3/182 | 0.012944019 | 0.052778192 | 0.033403919 | IGF1/HGF/HMOX1 | 3 |
| DOID:1557 | hypersensitivity reaction type III disease | 3/182 | 0.012944019 | 0.052778192 | 0.033403919 | IGF1/HGF/HMOX1 | 3 |
| DOID:3369 | peripheral primitive neuroectodermal tumor | 3/182 | 0.012944019 | 0.052778192 | 0.033403919 | IGF1/KIT/PTGS2 | 3 |
| DOID:631 | fibromyalgia | 3/182 | 0.012944019 | 0.052778192 | 0.033403919 | ADRA1A/ITGAM/IGF1 | 3 |
| DOID:9809 | hypersensitivity vasculitis | 3/182 | 0.012944019 | 0.052778192 | 0.033403919 | IGF1/HGF/HMOX1 | 3 |
| DOID:1485 | cystic fibrosis | 8/182 | 0.013205343 | 0.053145581 | 0.033636444 | IGF1/BPIFA1/TRPC6/CXCR1/ABCB1/HMOX1/PTGS2/EDN1 | 8 |
| DOID:11612 | polycystic ovary syndrome | 9/182 | 0.013262185 | 0.053145581 | 0.033636444 | INHBA/LCN2/IGF1/MGAM/FABP4/AQP9/HMOX1/ITGB3/CPB2 | 9 |
| DOID:13241 | Behcet's disease | 6/182 | 0.013306238 | 0.053145581 | 0.033636444 | STAT4/CCL21/ITGA2/CXCL12/HMOX1/CPB2 | 6 |
| DOID:37 | skin disease | 15/182 | 0.013370486 | 0.053145581 | 0.033636444 | RNASE2/LCN2/ADORA3/IGF1/KIT/MME/SELP/MS4A2/ANPEP/HMOX1/CXCL9/ITGB3/CCL5/SELE/IDO1 | 15 |
| DOID:3069 | astrocytoma | 7/182 | 0.013667657 | 0.053967331 | 0.034156539 | MATN2/S100A4/KIT/CXCL12/IL13RA2/AQP9/PTGS2 | 7 |
| DOID:4195 | hyperglycemia | 8/182 | 0.013748007 | 0.053967331 | 0.034156539 | CFH/IGF1/TRPC6/HMOX1/CCL5/PTGS2/SELE/EDN1 | 8 |
| DOID:3748 | esophagus squamous cell carcinoma | 5/182 | 0.013935746 | 0.05406772 | 0.034220076 | S100A9/INHBA/S100A8/PTGS2/SELE | 5 |
| DOID:4766 | embryoma | 15/182 | 0.014024828 | 0.05406772 | 0.034220076 | POSTN/SFRP2/CBS/PDGFD/LCN2/PROK2/IGF1/KIT/CXCL12/BIRC3/AQP9/SFRP4/ITGB3/PTGS2/IDO1 | 15 |
| DOID:0060005 | autoimmune disease of endocrine system | 6/182 | 0.014030231 | 0.05406772 | 0.034220076 | IGF1/CXCL9/CCL5/PTGS2/SELE/EDN1 | 6 |
| DOID:1520 | colon carcinoma | 9/182 | 0.014253731 | 0.054596109 | 0.034554499 | PDCD4/BCHE/SELP/ABCB1/HAS2/HMOX1/PTGS2/OLFM4/SELE | 9 |
| DOID:15 | reproductive system disease | 16/182 | 0.014442744 | 0.054986833 | 0.034801793 | ATP2B1/INHBA/IGF1/C5/CXCR2/CXCR1/IL1R2/CXCL12/ABCB1/ANGPT2/AQP9/ITGB3/CCL5/PTGS2/SELE/EDN1 | 16 |
| DOID:8692 | myeloid leukemia | 8/182 | 0.015473879 | 0.058559831 | 0.037063184 | LCN2/ITGAM/IGF1/KIT/MME/CXCL12/SELE/IDO1 | 8 |
| DOID:26 | pancreas disease | 9/182 | 0.015842644 | 0.059598517 | 0.037720581 | VCAM1/LCN2/CD14/IGF1/SELP/CA2/PTGS2/SELE/EDN1 | 9 |
| DOID:0060085 | organ system benign neoplasm | 12/182 | 0.01616499 | 0.060451325 | 0.038260332 | INHBA/LCN2/IGF1/KIT/SAA1/FGFR2/ITGA2/CXCL12/BMP5/EGR1/PTGS2/EDN1 | 12 |
| DOID:4989 | pancreatitis | 7/182 | 0.017035422 | 0.063331686 | 0.040083345 | LCN2/CD14/SELP/CA2/PTGS2/SELE/EDN1 | 7 |
| DOID:1192 | peripheral nervous system neoplasm | 16/182 | 0.017991875 | 0.066380658 | 0.042013074 | S100A4/INHBA/NQO1/CCN5/IGF1/KIT/HGF/ITGA2/CXCL12/ABCB1/BMP5/FAM107A/TFPI2/EGR1/HMOX1/PTGS2 | 16 |
| DOID:9500 | leukocyte disease | 6/182 | 0.018065622 | 0.066380658 | 0.042013074 | CSF3R/RNASE2/KIT/CXCR1/BIRC3/CCL5 | 6 |
| DOID:14069 | cerebral malaria | 3/182 | 0.018395837 | 0.06720329 | 0.042533728 | CR1/ANGPT2/HMOX1 | 3 |
| DOID:6590 | spondylitis | 5/182 | 0.018884861 | 0.068593289 | 0.043413474 | RGS1/ASPN/CD14/SAA1/ERAP2 | 5 |
| DOID:0050737 | autosomal recessive disease | 14/182 | 0.019753413 | 0.07133804 | 0.045150658 | HBB/HBA2/VCAM1/LCN2/IGF1/BPIFA1/TRPC6/CXCR1/SELP/ABCB1/HMOX1/PTGS2/SELE/EDN1 | 14 |
| DOID:7147 | ankylosing spondylitis | 5/182 | 0.019996904 | 0.071807064 | 0.045447509 | RGS1/ASPN/CD14/SAA1/ERAP2 | 5 |
| DOID:14256 | adult-onset Still's disease | 2/182 | 0.020508655 | 0.072008166 | 0.045574789 | S100A9/HMOX1 | 2 |
| DOID:2438 | dermis tumor | 2/182 | 0.020508655 | 0.072008166 | 0.045574789 | FGFR2/PTGS2 | 2 |
| DOID:2738 | pseudoxanthoma elasticum | 2/182 | 0.020508655 | 0.072008166 | 0.045574789 | SELP/SELE | 2 |
| DOID:4418 | cutaneous fibrous histiocytoma | 2/182 | 0.020508655 | 0.072008166 | 0.045574789 | FGFR2/PTGS2 | 2 |
| DOID:3211 | lysosomal storage disease | 8/182 | 0.020838878 | 0.072763377 | 0.04605277 | HBB/VCAM1/LUM/CHIT1/FABP4/IL13RA2/HMOX1/SELE | 8 |
| DOID:3068 | glioblastoma multiforme | 5/182 | 0.021151058 | 0.073046277 | 0.046231821 | S100A4/KIT/CXCL12/AQP9/PTGS2 | 5 |
| DOID:784 | chronic kidney failure | 5/182 | 0.021151058 | 0.073046277 | 0.046231821 | ADRA1A/HGF/CCL5/SELE/EDN1 | 5 |
| DOID:1107 | esophageal carcinoma | 7/182 | 0.021804889 | 0.074895052 | 0.047401932 | S100A9/S100A4/INHBA/S100A8/EGR1/PTGS2/SELE | 7 |
| DOID:9835 | refractive error | 5/182 | 0.022347863 | 0.076345131 | 0.048319703 | LUM/CFH/IGF1/HGF/EGR1 | 5 |
| DOID:688 | embryonal cancer | 15/182 | 0.02250072 | 0.076454059 | 0.048388645 | POSTN/SFRP2/CBS/PDGFD/LCN2/PROK2/IGF1/KIT/CXCL12/BIRC3/AQP9/SFRP4/ITGB3/PTGS2/IDO1 | 15 |
| DOID:657 | adenoma | 14/182 | 0.022640662 | 0.076518174 | 0.048429224 | SLC9A3R2/SFRP2/WIF1/OGN/NQO1/MALL/MME/HGF/CXCL12/ABCB1/HMOX1/PTGS2/THBS2/EDN1 | 14 |
