# Supplementary material for: FAM171B as a Novel Biomarker Mediates Tissue Immune Microenvironment in Pulmonary Arterial Hypertension
Source: Mediators Inflamm. 2022 Sep 22;2022:1878766. doi: 10.1155/2022/1878766 (PMC9553458; doi:10.1155/2022/1878766)
Supplement: Supplementary Materials — Supplementary Table 1: The results of differentially expressed genes (DEGs). Supplementary Table 2: Gene Ontology (GO) enrichment analysis results of differentially expressed genes (DEGs). Supplementary Table 3: Kyoto Encyclopedia of Genes and Genomes (KEGG) enrichment analysis results of differentially expressed genes (DEGs). Supplementary Table 4: Disease Ontology (DO) enrichment analysis results of differentially expressed genes (DEGs). Supplementary Table 5: Metascape function analysis results of differentially expressed genes (DEGs). Supplementary Table 6: results of Gene Set Enrichment Analysis (GSEA) of gene expression matrix. Supplementary Table 7: results of all genes in brown module. Supplementary Table 8: results of key genes in brown module. Supplementary Table 9: results of analyzing the combined data matrix of GSE113439 and GSE117261 using CIBERSORT. Supplementary Table 10: results of the correlation of FAM171B with immune cells. [file 1878766.f1.zip › Supplementary Table5.docx]

| **Gene Symbol** | **Description** | **Biological Process (GO)** | **Hallmark Gene Sets** |
| --- | --- | --- | --- |
| LTBP1 | latent transforming growth factor beta binding protein 1 | GO:0035583 sequestering of TGFbeta in extracellular matrix;GO:0071694 maintenance of protein location in extracellular region;GO:1901388 regulation of transforming growth factor beta activation | (M5942)HALLMARK UV RESPONSE DN |
| CSF3R | colony stimulating factor 3 receptor | GO:0030593 neutrophil chemotaxis;GO:0071621 granulocyte chemotaxis;GO:1990266 neutrophil migration | (M5897)HALLMARK IL6 JAK STAT3 SIGNALING; (M5932)HALLMARK INFLAMMATORY RESPONSE |
| ANKRD36C | ankyrin repeat domain 36C |  |  |
| HBB | hemoglobin subunit beta | GO:0030185 nitric oxide transport;GO:0015671 oxygen transport;GO:0070293 renal absorption | (M5945)HALLMARK HEME METABOLISM |
| PDE3A | phosphodiesterase 3A | GO:0060282 positive regulation of oocyte development;GO:0060281 regulation of oocyte development;GO:1905879 regulation of oogenesis |  |
| HBA2 | hemoglobin subunit alpha 2 | GO:0030185 nitric oxide transport;GO:0015671 oxygen transport;GO:0015669 gas transport |  |
| NKD1 | NKD inhibitor of WNT signaling pathway 1 | GO:1901229 regulation of non-canonical Wnt signaling pathway via JNK cascade;GO:1901231 positive regulation of non-canonical Wnt signaling pathway via JNK cascade;GO:1901232 regulation of convergent extension involved in axis elongation | (M5895)HALLMARK WNT BETA CATENIN SIGNALING |
| PSD3 | pleckstrin and Sec7 domain containing 3 | GO:0032012 regulation of ARF protein signal transduction;GO:0046578 regulation of Ras protein signal transduction;GO:0051056 regulation of small GTPase mediated signal transduction |  |
| SLC9A3R2 | SLC9A3 regulator 2 | GO:0072659 protein localization to plasma membrane;GO:1990778 protein localization to cell periphery;GO:0072657 protein localization to membrane |  |
| PDE4D | phosphodiesterase 4D | GO:0086024 adenylate cyclase-activating adrenergic receptor signaling pathway involved in positive regulation of heart rate;GO:1901898 negative regulation of relaxation of cardiac muscle;GO:0140200 adenylate cyclase-activating adrenergic receptor signaling pathway involved in regulation of heart rate |  |
| COL14A1 | collagen type XIV alpha 1 chain | GO:0030199 collagen fibril organization;GO:0030198 extracellular matrix organization;GO:0043062 extracellular structure organization |  |
| ECM2 | extracellular matrix protein 2 | GO:0010811 positive regulation of cell-substrate adhesion;GO:0007160 cell-matrix adhesion;GO:0031589 cell-substrate adhesion | (M5930)HALLMARK EPITHELIAL MESENCHYMAL TRANSITION |
| HIVEP1 | HIVEP zinc finger 1 | GO:0030509 BMP signaling pathway;GO:0071772 response to BMP;GO:0071773 cellular response to BMP stimulus |  |
| POSTN | periostin | GO:0071307 cellular response to vitamin K;GO:0032571 response to vitamin K;GO:0071295 cellular response to vitamin | (M5944)HALLMARK ANGIOGENESIS; (M5930)HALLMARK EPITHELIAL MESENCHYMAL TRANSITION |
| PDE7B | phosphodiesterase 7B | GO:0006198 cAMP catabolic process;GO:0009214 cyclic nucleotide catabolic process;GO:0046058 cAMP metabolic process |  |
| ADRA1A | adrenoceptor alpha 1A | GO:0001985 negative regulation of heart rate involved in baroreceptor response to increased systemic arterial blood pressure;GO:0001978 regulation of systemic arterial blood pressure by carotid sinus baroreceptor feedback;GO:0001983 baroreceptor response to increased systemic arterial blood pressure |  |
| NT5E | 5'-nucleotidase ecto | GO:0046086 adenosine biosynthetic process;GO:0046032 ADP catabolic process;GO:0006196 AMP catabolic process | (M5947)HALLMARK IL2 STAT5 SIGNALING; (M5930)HALLMARK EPITHELIAL MESENCHYMAL TRANSITION; (M5937)HALLMARK GLYCOLYSIS |
| FAM171B | family with sequence similarity 171 member B |  |  |
| ACSS2 | acyl-CoA synthetase short chain family member 2 | GO:0006083 acetate metabolic process;GO:0019427 acetyl-CoA biosynthetic process from acetate;GO:0006069 ethanol oxidation | (M5892)HALLMARK CHOLESTEROL HOMEOSTASIS |
| ANKRD36B | ankyrin repeat domain 36B |  |  |
| ANKRD36 | ankyrin repeat domain 36 |  |  |
| BICC1 | BicC family RNA binding protein 1 | GO:0007368 determination of left/right symmetry;GO:0009855 determination of bilateral symmetry;GO:0009799 specification of symmetry |  |
| TSHZ2 | teashirt zinc finger homeobox 2 | GO:0006357 regulation of transcription by RNA polymerase II;GO:0006355 regulation of transcription, DNA-templated;GO:1903506 regulation of nucleic acid-templated transcription |  |
| AHI1 | Abelson helper integration site 1 | GO:0030862 positive regulation of polarized epithelial cell differentiation;GO:0039007 pronephric nephron morphogenesis;GO:0039008 pronephric nephron tubule morphogenesis |  |
| FGR | FGR proto-oncogene, Src family tyrosine kinase | GO:0032815 negative regulation of natural killer cell activation;GO:0033008 positive regulation of mast cell activation involved in immune response;GO:0043306 positive regulation of mast cell degranulation | (M5950)HALLMARK ALLOGRAFT REJECTION |
| S100A9 | S100 calcium binding protein A9 | GO:0035606 peptidyl-cysteine S-trans-nitrosylation;GO:0070488 neutrophil aggregation;GO:0032119 sequestering of zinc ion | (M5907)HALLMARK ESTROGEN RESPONSE LATE; (M5921)HALLMARK COMPLEMENT |
| KRT4 | keratin 4 | GO:0050680 negative regulation of epithelial cell proliferation;GO:0050678 regulation of epithelial cell proliferation;GO:0030855 epithelial cell differentiation | (M5956)HALLMARK KRAS SIGNALING DN |
| LOC441081 | POM121 membrane glycoprotein (rat) pseudogene |  |  |
| RNASE2 | ribonuclease A family member 2 | GO:0002227 innate immune response in mucosa;GO:0002385 mucosal immune response;GO:0002251 organ or tissue specific immune response |  |
| STAT4 | signal transducer and activator of transcription 4 | GO:0007259 receptor signaling pathway via JAK-STAT;GO:0097696 receptor signaling pathway via STAT;GO:0043434 response to peptide hormone | (M5913)HALLMARK INTERFERON GAMMA RESPONSE; (M5950)HALLMARK ALLOGRAFT REJECTION |
| ABCC9 | ATP binding cassette subfamily C member 9 | GO:0033198 response to ATP;GO:1990573 potassium ion import across plasma membrane;GO:0061337 cardiac conduction |  |
| H1-0 | H1.0 linker histone | GO:0016584 nucleosome positioning;GO:2000679 positive regulation of transcription regulatory region DNA binding;GO:0031507 heterochromatin assembly | (M5908)HALLMARK ANDROGEN RESPONSE; (M5902)HALLMARK APOPTOSIS; (M5945)HALLMARK HEME METABOLISM |
| EPHA3 | EPH receptor A3 | GO:0097156 fasciculation of motor neuron axon;GO:0097155 fasciculation of sensory neuron axon;GO:0007413 axonal fasciculation |  |
| ANTXR1 | ANTXR cell adhesion molecule 1 | GO:1901202 negative regulation of extracellular matrix assembly;GO:1905050 positive regulation of metallopeptidase activity;GO:1903054 negative regulation of extracellular matrix organization |  |
| HIVEP2 | HIVEP zinc finger 2 | GO:0006357 regulation of transcription by RNA polymerase II;GO:0006355 regulation of transcription, DNA-templated;GO:1903506 regulation of nucleic acid-templated transcription |  |
| MATN2 | matrilin 2 | GO:0008150 biological_process | (M5930)HALLMARK EPITHELIAL MESENCHYMAL TRANSITION |
| RORA | RAR related orphan receptor A | GO:0021534 cell proliferation in hindbrain;GO:0021924 cell proliferation in external granule layer;GO:0021930 cerebellar granule cell precursor proliferation | (M5947)HALLMARK IL2 STAT5 SIGNALING; (M5891)HALLMARK HYPOXIA |
| GLT8D2 | glycosyltransferase 8 domain containing 2 |  |  |
| ATP2B1 | ATPase plasma membrane Ca2+ transporting 1 | GO:0071386 cellular response to corticosterone stimulus;GO:1990034 calcium ion export across plasma membrane;GO:1901660 calcium ion export | (M5942)HALLMARK UV RESPONSE DN; (M5890)HALLMARK TNFA SIGNALING VIA NFKB; (M5932)HALLMARK INFLAMMATORY RESPONSE |
| RGS5 | regulator of G protein signaling 5 | GO:0008277 regulation of G protein-coupled receptor signaling pathway;GO:0007186 G protein-coupled receptor signaling pathway;GO:0009968 negative regulation of signal transduction |  |
| PDE1A | phosphodiesterase 1A | GO:0007165 signal transduction;GO:0023052 signaling;GO:0007154 cell communication |  |
| GEM | GTP binding protein overexpressed in skeletal muscle | GO:1901842 negative regulation of high voltage-gated calcium channel activity;GO:1901841 regulation of high voltage-gated calcium channel activity;GO:1901386 negative regulation of voltage-gated calcium channel activity | (M5890)HALLMARK TNFA SIGNALING VIA NFKB; (M5930)HALLMARK EPITHELIAL MESENCHYMAL TRANSITION |
| HMCN1 | hemicentin 1 | GO:0090527 actin filament reorganization;GO:0071711 basement membrane organization;GO:0007157 heterophilic cell-cell adhesion via plasma membrane cell adhesion molecules |  |
| SLC7A7 | solute carrier family 7 member 7 | GO:0000821 regulation of arginine metabolic process;GO:0000820 regulation of glutamine family amino acid metabolic process;GO:1990822 basic amino acid transmembrane transport |  |
| LILRB3 | leukocyte immunoglobulin like receptor B3 | GO:0045671 negative regulation of osteoclast differentiation;GO:0002762 negative regulation of myeloid leukocyte differentiation;GO:0045670 regulation of osteoclast differentiation |  |
| PDCD4 | programmed cell death 4 | GO:0060940 epithelial to mesenchymal transition involved in cardiac fibroblast development;GO:1904761 negative regulation of myofibroblast differentiation;GO:0060935 cardiac fibroblast cell differentiation | (M5902)HALLMARK APOPTOSIS; (M5905)HALLMARK ADIPOGENESIS; (M5907)HALLMARK ESTROGEN RESPONSE LATE |
| MYO1F | myosin IF | GO:0030050 vesicle transport along actin filament;GO:0099515 actin filament-based transport;GO:0099518 vesicle cytoskeletal trafficking |  |
| MACC1 | MET transcriptional regulator MACC1 | GO:0051781 positive regulation of cell division;GO:0051302 regulation of cell division;GO:0045944 positive regulation of transcription by RNA polymerase II |  |
| AHCYL2 | adenosylhomocysteinase like 2 | GO:0033353 S-adenosylmethionine cycle;GO:0046500 S-adenosylmethionine metabolic process;GO:0006730 one-carbon metabolic process |  |
| FZD7 | frizzled class receptor 7 | GO:0042665 regulation of ectodermal cell fate specification;GO:0042666 negative regulation of ectodermal cell fate specification;GO:0014834 skeletal muscle satellite cell maintenance involved in skeletal muscle regeneration | (M5903)HALLMARK NOTCH SIGNALING |
| FREM1 | FRAS1 related extracellular matrix 1 | GO:0097094 craniofacial suture morphogenesis;GO:1904888 cranial skeletal system development;GO:0060349 bone morphogenesis |  |
| GGTA1 | glycoprotein alpha-galactosyltransferase 1 (inactive) | GO:0033575 protein glycosylation at cell surface;GO:0033580 protein galactosylation at cell surface;GO:0042125 protein galactosylation |  |
| RASGRP1 | RAS guanyl releasing protein 1 | GO:0032825 positive regulation of natural killer cell differentiation;GO:0033089 positive regulation of T cell differentiation in thymus;GO:0032252 secretory granule localization | (M5941)HALLMARK UV RESPONSE UP; (M5906)HALLMARK ESTROGEN RESPONSE EARLY; (M5921)HALLMARK COMPLEMENT |
| S100A4 | S100 calcium binding protein A4 | GO:0001837 epithelial to mesenchymal transition;GO:0048762 mesenchymal cell differentiation;GO:0043123 positive regulation of I-kappaB kinase/NF-kappaB signaling | (M5944)HALLMARK ANGIOGENESIS; (M5891)HALLMARK HYPOXIA; (M5939)HALLMARK P53 PATHWAY |
| RGS1 | regulator of G protein signaling 1 | GO:0061737 leukotriene signaling pathway;GO:0007193 adenylate cyclase-inhibiting G protein-coupled receptor signaling pathway;GO:0008277 regulation of G protein-coupled receptor signaling pathway | (M5932)HALLMARK INFLAMMATORY RESPONSE |
| GIMAP6 | GTPase, IMAP family member 6 |  |  |
| SLC36A1 | solute carrier family 36 member 1 | GO:0015734 taurine transport;GO:0042918 alkanesulfonate transport;GO:0015824 proline transport |  |
| TBX3 | T-box transcription factor 3 | GO:0046884 follicle-stimulating hormone secretion;GO:0014849 ureter smooth muscle contraction;GO:0072105 ureteric peristalsis |  |
| VCAM1 | vascular cell adhesion molecule 1 | GO:0060945 cardiac neuron differentiation;GO:0140039 cell-cell adhesion in response to extracellular stimulus;GO:0022614 membrane to membrane docking | (M5913)HALLMARK INTERFERON GAMMA RESPONSE; (M5915)HALLMARK APICAL JUNCTION; (M5930)HALLMARK EPITHELIAL MESENCHYMAL TRANSITION |
| LUM | lumican | GO:0032914 positive regulation of transforming growth factor beta1 production;GO:0032908 regulation of transforming growth factor beta1 production;GO:0071636 positive regulation of transforming growth factor beta production | (M5944)HALLMARK ANGIOGENESIS; (M5902)HALLMARK APOPTOSIS; (M5930)HALLMARK EPITHELIAL MESENCHYMAL TRANSITION |
| PLCB4 | phospholipase C beta 4 | GO:0048015 phosphatidylinositol-mediated signaling;GO:0048017 inositol lipid-mediated signaling;GO:0016042 lipid catabolic process | (M5942)HALLMARK UV RESPONSE DN |
| SULF1 | sulfatase 1 | GO:0014846 esophagus smooth muscle contraction;GO:0060686 negative regulation of prostatic bud formation;GO:0060685 regulation of prostatic bud formation |  |
| ARID5B | AT-rich interaction domain 5B | GO:0060613 fat pad development;GO:0010761 fibroblast migration;GO:0030325 adrenal gland development | (M5908)HALLMARK ANDROGEN RESPONSE; (M5913)HALLMARK INTERFERON GAMMA RESPONSE |
| SFRP2 | secreted frizzled related protein 2 | GO:0061185 negative regulation of dermatome development;GO:2000040 regulation of planar cell polarity pathway involved in axis elongation;GO:2000041 negative regulation of planar cell polarity pathway involved in axis elongation |  |
| CBS | cystathionine beta-synthase | GO:0006535 cysteine biosynthetic process from serine;GO:0019343 cysteine biosynthetic process via cystathionine;GO:0043418 homocysteine catabolic process | (M5892)HALLMARK CHOLESTEROL HOMEOSTASIS |
| CFH | complement factor H | GO:0030451 regulation of complement activation, alternative pathway;GO:1903659 regulation of complement-dependent cytotoxicity;GO:0006957 complement activation, alternative pathway | (M5946)HALLMARK COAGULATION; (M5913)HALLMARK INTERFERON GAMMA RESPONSE; (M5921)HALLMARK COMPLEMENT |
| INHBA | inhibin subunit beta A | GO:0042701 progesterone secretion;GO:0046882 negative regulation of follicle-stimulating hormone secretion;GO:0060279 positive regulation of ovulation | (M5890)HALLMARK TNFA SIGNALING VIA NFKB; (M5930)HALLMARK EPITHELIAL MESENCHYMAL TRANSITION; (M5932)HALLMARK INFLAMMATORY RESPONSE |
| SIGLEC9 | sialic acid binding Ig like lectin 9 | GO:0007155 cell adhesion;GO:0022610 biological adhesion;GO:0007166 cell surface receptor signaling pathway |  |
| UACA | uveal autoantigen with coiled-coil domains and ankyrin repeats | GO:1901223 negative regulation of NIK/NF-kappaB signaling;GO:1901222 regulation of NIK/NF-kappaB signaling;GO:0043280 positive regulation of cysteine-type endopeptidase activity involved in apoptotic process |  |
| MFGE8 | milk fat globule EGF and factor V/VIII domain containing | GO:0043277 apoptotic cell clearance;GO:0050766 positive regulation of phagocytosis;GO:0006910 phagocytosis, recognition |  |
| CNTN1 | contactin 1 | GO:0032289 central nervous system myelin formation;GO:0032288 myelin assembly;GO:0022010 central nervous system myelination | (M5915)HALLMARK APICAL JUNCTION |
| ZFPM2 | zinc finger protein, FOG family member 2 | GO:0003221 right ventricular cardiac muscle tissue morphogenesis;GO:2000195 negative regulation of female gonad development;GO:1905940 negative regulation of gonad development | (M5921)HALLMARK COMPLEMENT |
| WIF1 | WNT inhibitory factor 1 | GO:0045600 positive regulation of fat cell differentiation;GO:0045598 regulation of fat cell differentiation;GO:0030178 negative regulation of Wnt signaling pathway |  |
| OGN | osteoglycin | GO:0048662 negative regulation of smooth muscle cell proliferation;GO:0048660 regulation of smooth muscle cell proliferation;GO:0008285 negative regulation of cell population proliferation |  |
| PDGFD | platelet derived growth factor D | GO:0071673 positive regulation of smooth muscle cell chemotaxis;GO:2000439 positive regulation of monocyte extravasation;GO:2000437 regulation of monocyte extravasation |  |
| LCN2 | lipocalin 2 | GO:0015891 siderophore transport;GO:0097577 sequestering of iron ion;GO:0051238 sequestering of metal ion |  |
| NQO1 | NAD(P)H quinone dehydrogenase 1 | GO:1905395 response to flavonoid;GO:1904880 response to hydrogen sulfide;GO:1904772 response to tetrachloromethane | (M5938)HALLMARK REACTIVE OXYGEN SPECIES PATHWAY; (M5909)HALLMARK MYOGENESIS; (M5934)HALLMARK XENOBIOTIC METABOLISM |
| ASPN | asporin | GO:0070171 negative regulation of tooth mineralization;GO:1902617 response to fluoride;GO:0070170 regulation of tooth mineralization |  |
| XAF1 | XIAP associated factor 1 | GO:0035456 response to interferon-beta;GO:0034097 response to cytokine;GO:0006915 apoptotic process | (M5913)HALLMARK INTERFERON GAMMA RESPONSE |
| DPYSL3 | dihydropyrimidinase like 3 | GO:0051764 actin crosslink formation;GO:0051491 positive regulation of filopodium assembly;GO:0051489 regulation of filopodium assembly | (M5930)HALLMARK EPITHELIAL MESENCHYMAL TRANSITION |
| FAT3 | FAT atypical cadherin 3 | GO:0007156 homophilic cell adhesion via plasma membrane adhesion molecules;GO:0098742 cell-cell adhesion via plasma-membrane adhesion molecules;GO:0098609 cell-cell adhesion |  |
| MALL | mal, T cell differentiation protein like | GO:0042632 cholesterol homeostasis;GO:0055092 sterol homeostasis;GO:0042552 myelination | (M5953)HALLMARK KRAS SIGNALING UP |
| ITGAM | integrin subunit alpha M | GO:0045914 negative regulation of catecholamine metabolic process;GO:0045963 negative regulation of dopamine metabolic process;GO:0110090 positive regulation of hippocampal neuron apoptotic process | (M5921)HALLMARK COMPLEMENT |
| AGBL1 | AGBL carboxypeptidase 1 | GO:0035609 C-terminal protein deglutamylation;GO:0035610 protein side chain deglutamylation;GO:0035608 protein deglutamylation |  |
| S100A8 | S100 calcium binding protein A8 | GO:0070488 neutrophil aggregation;GO:0032119 sequestering of zinc ion;GO:0017014 protein nitrosylation |  |
| GLT1D1 | glycosyltransferase 1 domain containing 1 |  |  |
| LRRN4 | leucine rich repeat neuronal 4 | GO:0007616 long-term memory;GO:0008542 visual learning;GO:0007632 visual behavior |  |
| PLCB1 | phospholipase C beta 1 | GO:0060466 activation of meiosis involved in egg activation;GO:1905630 response to glyceraldehyde;GO:1905631 cellular response to glyceraldehyde | (M5923)HALLMARK PI3K AKT MTOR SIGNALING |
| PHGDH | phosphoglycerate dehydrogenase | GO:0009448 gamma-aminobutyric acid metabolic process;GO:0006564 L-serine biosynthetic process;GO:0006566 threonine metabolic process | (M5924)HALLMARK MTORC1 SIGNALING |
| SIGLEC10 | sialic acid binding Ig like lectin 10 | GO:0106015 negative regulation of inflammatory response to wounding;GO:0106014 regulation of inflammatory response to wounding;GO:1903035 negative regulation of response to wounding |  |
| CCN5 | cellular communication network factor 5 | GO:0007155 cell adhesion;GO:0022610 biological adhesion;GO:0007267 cell-cell signaling | (M5891)HALLMARK HYPOXIA; (M5906)HALLMARK ESTROGEN RESPONSE EARLY; (M5907)HALLMARK ESTROGEN RESPONSE LATE |
| CDON | cell adhesion associated, oncogene regulated | GO:0045663 positive regulation of myoblast differentiation;GO:0045661 regulation of myoblast differentiation;GO:0007224 smoothened signaling pathway | (M5942)HALLMARK UV RESPONSE DN |
| PROK2 | prokineticin 2 | GO:0001935 endothelial cell proliferation;GO:0045987 positive regulation of smooth muscle contraction;GO:0045933 positive regulation of muscle contraction | (M5932)HALLMARK INFLAMMATORY RESPONSE |
| PIEZO2 | piezo type mechanosensitive ion channel component 2 | GO:0050974 detection of mechanical stimulus involved in sensory perception;GO:0050982 detection of mechanical stimulus;GO:0071260 cellular response to mechanical stimulus |  |
| ENPP2 | ectonucleotide pyrophosphatase/phosphodiesterase 2 | GO:2000394 positive regulation of lamellipodium morphogenesis;GO:2000392 regulation of lamellipodium morphogenesis;GO:0034638 phosphatidylcholine catabolic process | (M5905)HALLMARK ADIPOGENESIS |
| TCN2 | transcobalamin 2 | GO:0006824 cobalt ion transport;GO:0015889 cobalamin transport;GO:0051180 vitamin transport | (M5939)HALLMARK P53 PATHWAY |
| ABCG2 | ATP binding cassette subfamily G member 2 (Junior blood group) | GO:0097744 urate salt excretion;GO:0015878 biotin transport;GO:1990962 xenobiotic transport across blood-brain barrier | (M5945)HALLMARK HEME METABOLISM |
| S100A12 | S100 calcium binding protein A12 | GO:0045576 mast cell activation;GO:0031640 killing of cells of other organism;GO:0002548 monocyte chemotaxis | (M5921)HALLMARK COMPLEMENT |
| CA4 | carbonic anhydrase 4 | GO:0015701 bicarbonate transport;GO:0006730 one-carbon metabolic process;GO:0015711 organic anion transport | (M5935)HALLMARK FATTY ACID METABOLISM |
| BMP6 | bone morphogenetic protein 6 | GO:0032346 positive regulation of aldosterone metabolic process;GO:0032349 positive regulation of aldosterone biosynthetic process;GO:1903392 negative regulation of adherens junction organization | (M5948)HALLMARK BILE ACID METABOLISM |
| AFF2 | AF4/FMR2 family member 2 | GO:0035063 nuclear speck organization;GO:0030575 nuclear body organization;GO:0006997 nucleus organization |  |
| RASEF | RAS and EF-hand domain containing |  |  |
| SEC14L4 | SEC14 like lipid binding 4 |  |  |
| SHMT2 | serine hydroxymethyltransferase 2 | GO:0019264 glycine biosynthetic process from serine;GO:0006565 L-serine catabolic process;GO:0006545 glycine biosynthetic process | (M5924)HALLMARK MTORC1 SIGNALING; (M5934)HALLMARK XENOBIOTIC METABOLISM |
| MXRA5 | matrix remodeling associated 5 | GO:0071559 response to transforming growth factor beta;GO:0070848 response to growth factor;GO:0009719 response to endogenous stimulus | (M5930)HALLMARK EPITHELIAL MESENCHYMAL TRANSITION |
| ESM1 | endothelial cell specific molecule 1 | GO:1902204 positive regulation of hepatocyte growth factor receptor signaling pathway;GO:1902202 regulation of hepatocyte growth factor receptor signaling pathway;GO:0002040 sprouting angiogenesis |  |
| PI15 | peptidase inhibitor 15 | GO:0010466 negative regulation of peptidase activity;GO:0045861 negative regulation of proteolysis;GO:0051346 negative regulation of hydrolase activity |  |
| PLPPR4 | phospholipid phosphatase related 4 | GO:0140354 lipid import into cell;GO:0046839 phospholipid dephosphorylation;GO:0051966 regulation of synaptic transmission, glutamatergic | (M5902)HALLMARK APOPTOSIS |
| ZNF521 | zinc finger protein 521 | GO:0048663 neuron fate commitment;GO:0045165 cell fate commitment;GO:0030182 neuron differentiation |  |
| MS4A15 | membrane spanning 4-domains A15 | GO:0007166 cell surface receptor signaling pathway;GO:0007165 signal transduction;GO:0023052 signaling |  |
| ADORA3 | adenosine A3 receptor | GO:0001973 G protein-coupled adenosine receptor signaling pathway;GO:0035588 G protein-coupled purinergic receptor signaling pathway;GO:0014061 regulation of norepinephrine secretion |  |
| LILRA1 | leukocyte immunoglobulin like receptor A1 | GO:0019221 cytokine-mediated signaling pathway;GO:0002250 adaptive immune response;GO:0071345 cellular response to cytokine stimulus |  |
| SNORD114-2 | small nucleolar RNA, C/D box 114-2 | GO:0006396 RNA processing;GO:0016070 RNA metabolic process;GO:0010467 gene expression |  |
| CD69 | CD69 molecule | GO:0071466 cellular response to xenobiotic stimulus;GO:0009410 response to xenobiotic stimulus;GO:0070887 cellular response to chemical stimulus | (M5902)HALLMARK APOPTOSIS; (M5890)HALLMARK TNFA SIGNALING VIA NFKB; (M5913)HALLMARK INTERFERON GAMMA RESPONSE |
| MYH10 | myosin heavy chain 10 | GO:0000281 mitotic cytokinesis;GO:0061640 cytoskeleton-dependent cytokinesis;GO:0030048 actin filament-based movement | (M5893)HALLMARK MITOTIC SPINDLE; (M5915)HALLMARK APICAL JUNCTION |
| CCL21 | C-C motif chemokine ligand 21 | GO:0097026 dendritic cell dendrite assembly;GO:2000548 negative regulation of dendritic cell dendrite assembly;GO:0035759 mesangial cell-matrix adhesion |  |
| CD14 | CD14 molecule | GO:0071725 response to triacyl bacterial lipopeptide;GO:0071727 cellular response to triacyl bacterial lipopeptide;GO:0071724 response to diacyl bacterial lipopeptide | (M5897)HALLMARK IL6 JAK STAT3 SIGNALING; (M5902)HALLMARK APOPTOSIS; (M5932)HALLMARK INFLAMMATORY RESPONSE |
| ABCA8 | ATP binding cassette subfamily A member 8 | GO:0006686 sphingomyelin biosynthetic process;GO:0006684 sphingomyelin metabolic process;GO:0033344 cholesterol efflux | (M5948)HALLMARK BILE ACID METABOLISM |
| ALAS2 | 5'-aminolevulinate synthase 2 | GO:0042541 hemoglobin biosynthetic process;GO:0032364 oxygen homeostasis;GO:0006782 protoporphyrinogen IX biosynthetic process | (M5945)HALLMARK HEME METABOLISM |
| SECISBP2L | SECIS binding protein 2 like | GO:0001514 selenocysteine incorporation;GO:0006451 translational readthrough;GO:0006414 translational elongation |  |
| IGF1 | insulin like growth factor 1 | GO:0060283 negative regulation of oocyte development;GO:1904073 regulation of trophectodermal cell proliferation;GO:1904075 positive regulation of trophectodermal cell proliferation | (M5909)HALLMARK MYOGENESIS; (M5934)HALLMARK XENOBIOTIC METABOLISM |
| GFRA1 | GDNF family receptor alpha 1 | GO:0035860 glial cell-derived neurotrophic factor receptor signaling pathway;GO:0038179 neurotrophin signaling pathway;GO:0008584 male gonad development | (M5906)HALLMARK ESTROGEN RESPONSE EARLY |
| ITGB6 | integrin subunit beta 6 | GO:0061520 Langerhans cell differentiation;GO:0060435 bronchiole development;GO:0071604 transforming growth factor beta production |  |
| C3AR1 | complement C3a receptor 1 | GO:0002430 complement receptor mediated signaling pathway;GO:0010759 positive regulation of macrophage chemotaxis;GO:0090023 positive regulation of neutrophil chemotaxis | (M5932)HALLMARK INFLAMMATORY RESPONSE; (M5953)HALLMARK KRAS SIGNALING UP |
| CCDC80 | coiled-coil domain containing 80 | GO:0010811 positive regulation of cell-substrate adhesion;GO:0010810 regulation of cell-substrate adhesion;GO:0030198 extracellular matrix organization |  |
| RNF182 | ring finger protein 182 | GO:0016567 protein ubiquitination;GO:0032446 protein modification by small protein conjugation;GO:0070647 protein modification by small protein conjugation or removal |  |
| SLCO4A1 | solute carrier organic anion transporter family member 4A1 | GO:0070327 thyroid hormone transport;GO:0043252 sodium-independent organic anion transport;GO:0009914 hormone transport |  |
| TMEM45B | transmembrane protein 45B |  |  |
| ACE2 | angiotensin converting enzyme 2 | GO:0070881 regulation of proline transport;GO:1902834 regulation of proline import across plasma membrane;GO:1902836 positive regulation of proline import across plasma membrane |  |
| C5 | complement C5 | GO:0010760 negative regulation of macrophage chemotaxis;GO:1905522 negative regulation of macrophage migration;GO:0006957 complement activation, alternative pathway | (M5956)HALLMARK KRAS SIGNALING DN |
| KIT | KIT proto-oncogene, receptor tyrosine kinase | GO:0070662 mast cell proliferation;GO:0097326 melanocyte adhesion;GO:0120071 regulation of pyloric antrum smooth muscle contraction | (M5942)HALLMARK UV RESPONSE DN |
| MGAM | maltase-glucoamylase | GO:0000025 maltose catabolic process;GO:0005982 starch metabolic process;GO:0005983 starch catabolic process |  |
| BPIFA1 | BPI fold containing family A member 1 | GO:0050828 regulation of liquid surface tension;GO:1900190 regulation of single-species biofilm formation;GO:1900191 negative regulation of single-species biofilm formation |  |
| FAM13C | family with sequence similarity 13 member C |  |  |
| SAA1 | serum amyloid A1 | GO:0048246 macrophage chemotaxis;GO:1905517 macrophage migration;GO:0006953 acute-phase response |  |
| CXCR2 | C-X-C motif chemokine receptor 2 | GO:0038112 interleukin-8-mediated signaling pathway;GO:0098758 response to interleukin-8;GO:0098759 cellular response to interleukin-8 |  |
| PDLIM3 | PDZ and LIM domain 3 | GO:0061061 muscle structure development;GO:0030036 actin cytoskeleton organization;GO:0007507 heart development | (M5941)HALLMARK UV RESPONSE UP; (M5906)HALLMARK ESTROGEN RESPONSE EARLY; (M5907)HALLMARK ESTROGEN RESPONSE LATE |
| LGALSL | galectin like |  |  |
| WEE1 | WEE1 G2 checkpoint kinase | GO:0000086 G2/M transition of mitotic cell cycle;GO:0044839 cell cycle G2/M phase transition;GO:2000134 negative regulation of G1/S transition of mitotic cell cycle | (M5902)HALLMARK APOPTOSIS; (M5925)HALLMARK E2F TARGETS |
| PLAC8 | placenta associated 8 | GO:0040015 negative regulation of multicellular organism growth;GO:0050873 brown fat cell differentiation;GO:0009409 response to cold | (M5891)HALLMARK HYPOXIA |
| CFAP69 | cilia and flagella associated protein 69 | GO:1990834 response to odorant;GO:1905516 positive regulation of fertilization;GO:0042048 olfactory behavior |  |
| TRPC6 | transient receptor potential cation channel subfamily C member 6 | GO:0006828 manganese ion transport;GO:0000041 transition metal ion transport;GO:0032414 positive regulation of ion transmembrane transporter activity |  |
| GBP5 | guanylate binding protein 5 | GO:0032741 positive regulation of interleukin-18 production;GO:1900227 positive regulation of NLRP3 inflammasome complex assembly;GO:0032661 regulation of interleukin-18 production |  |
| RGS13 | regulator of G protein signaling 13 | GO:0045744 negative regulation of G protein-coupled receptor signaling pathway;GO:0008277 regulation of G protein-coupled receptor signaling pathway;GO:0007186 G protein-coupled receptor signaling pathway |  |
| LRRC36 | leucine rich repeat containing 36 |  |  |
| CPA3 | carboxypeptidase A3 | GO:0002002 regulation of angiotensin levels in blood;GO:0002003 angiotensin maturation;GO:0001991 regulation of systemic arterial blood pressure by circulatory renin-angiotensin |  |
| FGFR2 | fibroblast growth factor receptor 2 | GO:0035602 fibroblast growth factor receptor signaling pathway involved in negative regulation of apoptotic process in bone marrow cell;GO:0035603 fibroblast growth factor receptor signaling pathway involved in hemopoiesis;GO:0035604 fibroblast growth factor receptor signaling pathway involved in positive regulation of cell proliferation in bone marrow |  |
| ACADL | acyl-CoA dehydrogenase long chain | GO:0042413 carnitine catabolic process;GO:0019254 carnitine metabolic process, CoA-linked;GO:0006579 amino-acid betaine catabolic process | (M5935)HALLMARK FATTY ACID METABOLISM; (M5905)HALLMARK ADIPOGENESIS |
| MME | membrane metalloendopeptidase | GO:0046449 creatinine metabolic process;GO:0061837 neuropeptide processing;GO:0072338 cellular lactam metabolic process |  |
| SEMA3D | semaphorin 3D | GO:0048843 negative regulation of axon extension involved in axon guidance;GO:0048841 regulation of axon extension involved in axon guidance;GO:0071526 semaphorin-plexin signaling pathway |  |
| CNKSR3 | CNKSR family member 3 | GO:2000651 positive regulation of sodium ion transmembrane transporter activity;GO:1902307 positive regulation of sodium ion transmembrane transport;GO:0033137 negative regulation of peptidyl-serine phosphorylation |  |
| BPIFB1 | BPI fold containing family B member 1 | GO:0034144 negative regulation of toll-like receptor 4 signaling pathway;GO:0034143 regulation of toll-like receptor 4 signaling pathway;GO:0002227 innate immune response in mucosa |  |
| EPHA4 | EPH receptor A4 | GO:0097156 fasciculation of motor neuron axon;GO:1905099 positive regulation of guanyl-nucleotide exchange factor activity;GO:2001108 positive regulation of Rho guanyl-nucleotide exchange factor activity |  |
| SULT1B1 | sulfotransferase family 1B member 1 | GO:0006068 ethanol catabolic process;GO:0034310 primary alcohol catabolic process;GO:0009812 flavonoid metabolic process | (M5948)HALLMARK BILE ACID METABOLISM |
| CXCR1 | C-X-C motif chemokine receptor 1 | GO:0038112 interleukin-8-mediated signaling pathway;GO:0098758 response to interleukin-8;GO:0098759 cellular response to interleukin-8 |  |
| C7 | complement C7 | GO:0006957 complement activation, alternative pathway;GO:0019835 cytolysis;GO:0006958 complement activation, classical pathway |  |
| ITK | IL2 inducible T cell kinase | GO:0001865 NK T cell differentiation;GO:0046629 gamma-delta T cell activation;GO:0038083 peptidyl-tyrosine autophosphorylation | (M5950)HALLMARK ALLOGRAFT REJECTION |
| NR1D2 | nuclear receptor subfamily 1 group D member 2 | GO:2001014 regulation of skeletal muscle cell differentiation;GO:0048512 circadian behavior;GO:0007622 rhythmic behavior | (M5942)HALLMARK UV RESPONSE DN |
| OVOS2 | alpha-2-macroglobulin like 1 pseudogene | GO:0010951 negative regulation of endopeptidase activity;GO:0010466 negative regulation of peptidase activity;GO:0045861 negative regulation of proteolysis |  |
| IL1R2 | interleukin 1 receptor type 2 | GO:0032690 negative regulation of interleukin-1 alpha production;GO:2000660 negative regulation of interleukin-1-mediated signaling pathway;GO:2000659 regulation of interleukin-1-mediated signaling pathway | (M5897)HALLMARK IL6 JAK STAT3 SIGNALING; (M5947)HALLMARK IL2 STAT5 SIGNALING |
| FCN3 | ficolin 3 | GO:0043654 recognition of apoptotic cell;GO:1903028 positive regulation of opsonization;GO:1903027 regulation of opsonization |  |
| LILRA3 | leukocyte immunoglobulin like receptor A3 | GO:0019221 cytokine-mediated signaling pathway;GO:0002250 adaptive immune response;GO:0071345 cellular response to cytokine stimulus |  |
| LILRB2 | leukocyte immunoglobulin like receptor B2 | GO:1905875 negative regulation of postsynaptic density organization;GO:0140105 interleukin-10-mediated signaling pathway;GO:2000524 negative regulation of T cell costimulation |  |
| HGF | hepatocyte growth factor | GO:0060665 regulation of branching involved in salivary gland morphogenesis by mesenchymal-epithelial signaling;GO:0051450 myoblast proliferation;GO:0060638 mesenchymal-epithelial cell signaling | (M5902)HALLMARK APOPTOSIS |
| ITGA2 | integrin subunit alpha 2 | GO:0033343 positive regulation of collagen binding;GO:0033341 regulation of collagen binding;GO:0033591 response to L-ascorbic acid | (M5946)HALLMARK COAGULATION; (M5915)HALLMARK APICAL JUNCTION; (M5930)HALLMARK EPITHELIAL MESENCHYMAL TRANSITION |
| PAMR1 | peptidase domain containing associated with muscle regeneration 1 |  |  |
| CHIT1 | chitinase 1 | GO:0044245 polysaccharide digestion;GO:0006030 chitin metabolic process;GO:0006032 chitin catabolic process |  |
| SOSTDC1 | sclerostin domain containing 1 | GO:2000016 negative regulation of determination of dorsal identity;GO:0060648 mammary gland bud morphogenesis;GO:2000015 regulation of determination of dorsal identity |  |
| SLC18A2 | solute carrier family 18 member A2 | GO:0042137 sequestering of neurotransmitter;GO:0015842 aminergic neurotransmitter loading into synaptic vesicle;GO:0051610 serotonin uptake |  |
| SLC7A2 | solute carrier family 7 member 2 | GO:0097638 L-arginine import across plasma membrane;GO:1903352 L-ornithine transmembrane transport;GO:1902022 L-lysine transport | (M5906)HALLMARK ESTROGEN RESPONSE EARLY; (M5932)HALLMARK INFLAMMATORY RESPONSE |
| ACOXL | acyl-CoA oxidase like | GO:0033540 fatty acid beta-oxidation using acyl-CoA oxidase;GO:0006635 fatty acid beta-oxidation;GO:0019395 fatty acid oxidation |  |
| FPR1 | formyl peptide receptor 1 | GO:0002430 complement receptor mediated signaling pathway;GO:0007263 nitric oxide mediated signal transduction;GO:0007200 phospholipase C-activating G protein-coupled receptor signaling pathway | (M5913)HALLMARK INTERFERON GAMMA RESPONSE; (M5932)HALLMARK INFLAMMATORY RESPONSE |
| BCHE | butyrylcholinesterase | GO:0046448 tropane alkaloid metabolic process;GO:0050783 cocaine metabolic process;GO:0006581 acetylcholine catabolic process |  |
| FABP4 | fatty acid binding protein 4 | GO:0071285 cellular response to lithium ion;GO:0050872 white fat cell differentiation;GO:0010226 response to lithium ion | (M5905)HALLMARK ADIPOGENESIS |
| CXCL12 | C-X-C motif chemokine ligand 12 | GO:1990478 response to ultrasound;GO:1903237 negative regulation of leukocyte tethering or rolling;GO:0033603 positive regulation of dopamine secretion | (M5906)HALLMARK ESTROGEN RESPONSE EARLY; (M5907)HALLMARK ESTROGEN RESPONSE LATE; (M5930)HALLMARK EPITHELIAL MESENCHYMAL TRANSITION |
| SELP | selectin P | GO:0010572 positive regulation of platelet activation;GO:1903238 positive regulation of leukocyte tethering or rolling;GO:1903236 regulation of leukocyte tethering or rolling | (M5947)HALLMARK IL2 STAT5 SIGNALING; (M5913)HALLMARK INTERFERON GAMMA RESPONSE |
| ABCB1 | ATP binding cassette subfamily B member 1 | GO:0099040 ceramide translocation;GO:0099039 sphingolipid translocation;GO:0046864 isoprenoid transport | (M5949)HALLMARK PEROXISOME; (M5941)HALLMARK UV RESPONSE UP; (M5947)HALLMARK IL2 STAT5 SIGNALING |
| THY1 | Thy-1 cell surface antigen | GO:2000298 regulation of Rho-dependent protein serine/threonine kinase activity;GO:0042670 retinal cone cell differentiation;GO:0046549 retinal cone cell development | (M5919)HALLMARK HEDGEHOG SIGNALING; (M5916)HALLMARK APICAL SURFACE; (M5915)HALLMARK APICAL JUNCTION |
| CA2 | carbonic anhydrase 2 | GO:0090088 regulation of oligopeptide transport;GO:0090089 regulation of dipeptide transport;GO:2000878 positive regulation of oligopeptide transport | (M5935)HALLMARK FATTY ACID METABOLISM; (M5941)HALLMARK UV RESPONSE UP; (M5947)HALLMARK IL2 STAT5 SIGNALING |
| MS4A2 | membrane spanning 4-domains A2 | GO:0038095 Fc-epsilon receptor signaling pathway;GO:0038093 Fc receptor signaling pathway;GO:0002768 immune response-regulating cell surface receptor signaling pathway |  |
| RFTN2 | raftlin family member 2 | GO:0033227 dsRNA transport;GO:0043330 response to exogenous dsRNA;GO:0043331 response to dsRNA |  |
| SYNPO2 | synaptopodin 2 | GO:0061684 chaperone-mediated autophagy;GO:0032233 positive regulation of actin filament bundle assembly;GO:0000045 autophagosome assembly |  |
| BMP5 | bone morphogenetic protein 5 | GO:0021502 neural fold elevation formation;GO:1905069 allantois development;GO:0032345 negative regulation of aldosterone metabolic process |  |
| F11 | coagulation factor XI | GO:0051919 positive regulation of fibrinolysis;GO:0031639 plasminogen activation;GO:0051917 regulation of fibrinolysis | (M5946)HALLMARK COAGULATION; (M5934)HALLMARK XENOBIOTIC METABOLISM |
| HIF3A | hypoxia inducible factor 3 subunit alpha | GO:0006366 transcription by RNA polymerase II;GO:0001666 response to hypoxia;GO:0036293 response to decreased oxygen levels |  |
| HCK | HCK proto-oncogene, Src family tyrosine kinase | GO:0002522 leukocyte migration involved in immune response;GO:0045728 respiratory burst after phagocytosis;GO:0002679 respiratory burst involved in defense response |  |
| CA12 | carbonic anhydrase 12 | GO:0055064 chloride ion homeostasis;GO:0055083 monovalent inorganic anion homeostasis;GO:0055081 anion homeostasis | (M5891)HALLMARK HYPOXIA; (M5906)HALLMARK ESTROGEN RESPONSE EARLY; (M5907)HALLMARK ESTROGEN RESPONSE LATE |
| GXYLT2 | glucoside xylosyltransferase 2 | GO:0016266 O-glycan processing;GO:0006493 protein O-linked glycosylation;GO:0006486 protein glycosylation |  |
| PDE8B | phosphodiesterase 8B | GO:0006198 cAMP catabolic process;GO:0009214 cyclic nucleotide catabolic process;GO:0046058 cAMP metabolic process |  |
| CR1 | complement C3b/C4b receptor 1 (Knops blood group) | GO:0002435 immune complex clearance by erythrocytes;GO:1900099 negative regulation of plasma cell differentiation;GO:0001970 positive regulation of activation of membrane attack complex | (M5921)HALLMARK COMPLEMENT |
| ANPEP | alanyl aminopeptidase, membrane | GO:0043171 peptide catabolic process;GO:0046718 viral entry into host cell;GO:0044409 entry into host | (M5930)HALLMARK EPITHELIAL MESENCHYMAL TRANSITION |
| TRAT1 | T cell receptor associated transmembrane adaptor 1 | GO:0001920 negative regulation of receptor recycling;GO:0050862 positive regulation of T cell receptor signaling pathway;GO:0001919 regulation of receptor recycling | (M5950)HALLMARK ALLOGRAFT REJECTION |
| RIPOR2 | RHO family interacting cell polarization regulator 2 | GO:1903904 negative regulation of establishment of T cell polarity;GO:1905872 negative regulation of protein localization to cell leading edge;GO:2001107 negative regulation of Rho guanyl-nucleotide exchange factor activity |  |
| CCR1 | C-C motif chemokine receptor 1 | GO:0002407 dendritic cell chemotaxis;GO:0090026 positive regulation of monocyte chemotaxis;GO:0030502 negative regulation of bone mineralization | (M5897)HALLMARK IL6 JAK STAT3 SIGNALING; (M5950)HALLMARK ALLOGRAFT REJECTION |
| IL13RA2 | interleukin 13 receptor subunit alpha 2 | GO:0043305 negative regulation of mast cell degranulation;GO:0033007 negative regulation of mast cell activation involved in immune response;GO:0002638 negative regulation of immunoglobulin production | (M5951)HALLMARK SPERMATOGENESIS |
| ITGAX | integrin subunit alpha X | GO:1905956 positive regulation of endothelial tube morphogenesis;GO:1901509 regulation of endothelial tube morphogenesis;GO:0031643 positive regulation of myelination |  |
| VSIG4 | V-set and immunoglobulin domain containing 4 | GO:0045957 negative regulation of complement activation, alternative pathway;GO:0030451 regulation of complement activation, alternative pathway;GO:0045916 negative regulation of complement activation |  |
| FAM107A | family with sequence similarity 107 member A | GO:1900272 negative regulation of long-term synaptic potentiation;GO:0051895 negative regulation of focal adhesion assembly;GO:0150118 negative regulation of cell-substrate junction organization |  |
| BIRC3 | baculoviral IAP repeat containing 3 | GO:0070424 regulation of nucleotide-binding oligomerization domain containing signaling pathway;GO:0060546 negative regulation of necroptotic process;GO:0062099 negative regulation of programmed necrotic cell death | (M5902)HALLMARK APOPTOSIS; (M5890)HALLMARK TNFA SIGNALING VIA NFKB; (M5953)HALLMARK KRAS SIGNALING UP |
| TM4SF18 | transmembrane 4 L six family member 18 |  |  |
| GPR146 | G protein-coupled receptor 146 | GO:0007186 G protein-coupled receptor signaling pathway;GO:0007165 signal transduction;GO:0023052 signaling |  |
| VNN2 | vanin 2 | GO:0015939 pantothenate metabolic process;GO:0006767 water-soluble vitamin metabolic process;GO:0006766 vitamin metabolic process |  |
| ADAMTS9 | ADAM metallopeptidase with thrombospondin type 1 motif 9 | GO:0090673 endothelial cell-matrix adhesion;GO:0045636 positive regulation of melanocyte differentiation;GO:0045634 regulation of melanocyte differentiation |  |
| ANGPT2 | angiopoietin 2 | GO:0050928 negative regulation of positive chemotaxis;GO:0048014 Tie signaling pathway;GO:0072012 glomerulus vasculature development |  |
| TFPI2 | tissue factor pathway inhibitor 2 | GO:0071498 cellular response to fluid shear stress;GO:0034405 response to fluid shear stress;GO:0007596 blood coagulation | (M5946)HALLMARK COAGULATION; (M5907)HALLMARK ESTROGEN RESPONSE LATE; (M5921)HALLMARK COMPLEMENT |
| LRRC32 | leucine rich repeat containing 32 | GO:1901398 regulation of transforming growth factor beta3 activation;GO:0032910 regulation of transforming growth factor beta3 production;GO:1901388 regulation of transforming growth factor beta activation |  |
| HAS2 | hyaluronan synthase 2 | GO:0070295 renal water absorption;GO:1900623 regulation of monocyte aggregation;GO:1900625 positive regulation of monocyte aggregation | (M5942)HALLMARK UV RESPONSE DN; (M5932)HALLMARK INFLAMMATORY RESPONSE |
| AREG | amphiregulin | GO:0060598 dichotomous subdivision of terminal units involved in mammary gland duct morphogenesis;GO:0060751 branch elongation involved in mammary gland duct branching;GO:0042695 thelarche | (M5890)HALLMARK TNFA SIGNALING VIA NFKB; (M5906)HALLMARK ESTROGEN RESPONSE EARLY; (M5907)HALLMARK ESTROGEN RESPONSE LATE |
| AKR1C2 | aldo-keto reductase family 1 member C2 | GO:0009753 response to jasmonic acid;GO:0071395 cellular response to jasmonic acid stimulus;GO:0071798 response to prostaglandin D | (M5934)HALLMARK XENOBIOTIC METABOLISM |
| EGR1 | early growth response 1 | GO:0072303 positive regulation of glomerular metanephric mesangial cell proliferation;GO:0071505 response to mycophenolic acid;GO:0071506 cellular response to mycophenolic acid | (M5890)HALLMARK TNFA SIGNALING VIA NFKB |
| MFAP4 | microfibril associated protein 4 | GO:0071493 cellular response to UV-B;GO:0001867 complement activation, lectin pathway;GO:0048251 elastic fiber assembly |  |
| BTNL9 | butyrophilin like 9 | GO:0050852 T cell receptor signaling pathway;GO:0050851 antigen receptor-mediated signaling pathway;GO:0002429 immune response-activating cell surface receptor signaling pathway |  |
| ELF5 | E74 like ETS transcription factor 5 | GO:0006357 regulation of transcription by RNA polymerase II;GO:0006355 regulation of transcription, DNA-templated;GO:1903506 regulation of nucleic acid-templated transcription |  |
| SERPINE2 | serpin family E member 2 | GO:0042628 mating plug formation;GO:0061108 seminal vesicle epithelium development;GO:0061107 seminal vesicle development | (M5930)HALLMARK EPITHELIAL MESENCHYMAL TRANSITION |
| STEAP2 | STEAP2 metalloreductase | GO:0015679 plasma membrane copper ion transport;GO:0098705 copper ion import across plasma membrane;GO:0098711 iron ion import across plasma membrane |  |
| TLL1 | tolloid like 1 | GO:0030199 collagen fibril organization;GO:0030198 extracellular matrix organization;GO:0043062 extracellular structure organization |  |
| AQP9 | aquaporin 9 | GO:0015722 canalicular bile acid transport;GO:1904823 purine nucleobase transmembrane transport;GO:0015855 pyrimidine nucleobase transport | (M5948)HALLMARK BILE ACID METABOLISM; (M5932)HALLMARK INFLAMMATORY RESPONSE; (M5934)HALLMARK XENOBIOTIC METABOLISM |
| OLR1 | oxidized low density lipoprotein receptor 1 | GO:0007159 leukocyte cell-cell adhesion;GO:0042157 lipoprotein metabolic process;GO:0008015 blood circulation | (M5944)HALLMARK ANGIOGENESIS; (M5946)HALLMARK COAGULATION; (M5890)HALLMARK TNFA SIGNALING VIA NFKB |
| SLCO1A2 | solute carrier organic anion transporter family member 1A2 | GO:0043252 sodium-independent organic anion transport;GO:0015721 bile acid and bile salt transport;GO:0015718 monocarboxylic acid transport | (M5948)HALLMARK BILE ACID METABOLISM |
| IFI44L | interferon induced protein 44 like | GO:0051607 defense response to virus;GO:0140546 defense response to symbiont;GO:0009615 response to virus | (M5911)HALLMARK INTERFERON ALPHA RESPONSE; (M5913)HALLMARK INTERFERON GAMMA RESPONSE; (M5956)HALLMARK KRAS SIGNALING DN |
| FRAS1 | Fraser extracellular matrix complex subunit 1 | GO:0003338 metanephros morphogenesis;GO:0001656 metanephros development;GO:0060993 kidney morphogenesis |  |
| VIPR1 | vasoactive intestinal peptide receptor 1 | GO:0007187 G protein-coupled receptor signaling pathway, coupled to cyclic nucleotide second messenger;GO:0007188 adenylate cyclase-modulating G protein-coupled receptor signaling pathway;GO:0008284 positive regulation of cell population proliferation | (M5909)HALLMARK MYOGENESIS |
| COLEC10 | collectin subfamily member 10 | GO:1903028 positive regulation of opsonization;GO:1903027 regulation of opsonization;GO:0001867 complement activation, lectin pathway |  |
| SFRP4 | secreted frizzled related protein 4 | GO:2000119 negative regulation of sodium-dependent phosphate transport;GO:1902174 positive regulation of keratinocyte apoptotic process;GO:2000118 regulation of sodium-dependent phosphate transport | (M5930)HALLMARK EPITHELIAL MESENCHYMAL TRANSITION |
| HMOX1 | heme oxygenase 1 | GO:0032764 negative regulation of mast cell cytokine production;GO:0006788 heme oxidation;GO:0014806 smooth muscle hyperplasia | (M5897)HALLMARK IL6 JAK STAT3 SIGNALING; (M5941)HALLMARK UV RESPONSE UP; (M5902)HALLMARK APOPTOSIS |
| COL6A6 | collagen type VI alpha 6 chain | GO:0030198 extracellular matrix organization;GO:0043062 extracellular structure organization;GO:0045229 external encapsulating structure organization |  |
| TIMP4 | TIMP metallopeptidase inhibitor 4 | GO:0051045 negative regulation of membrane protein ectodomain proteolysis;GO:0051043 regulation of membrane protein ectodomain proteolysis;GO:0042698 ovulation cycle |  |
| PRRX1 | paired related homeobox 1 | GO:0100026 positive regulation of DNA repair by transcription from RNA polymerase II promoter;GO:0048664 neuron fate determination;GO:0042474 middle ear morphogenesis | (M5930)HALLMARK EPITHELIAL MESENCHYMAL TRANSITION; (M5953)HALLMARK KRAS SIGNALING UP |
| CXCL9 | C-X-C motif chemokine ligand 9 | GO:1901741 positive regulation of myoblast fusion;GO:1901739 regulation of myoblast fusion;GO:0045663 positive regulation of myoblast differentiation | (M5897)HALLMARK IL6 JAK STAT3 SIGNALING; (M5913)HALLMARK INTERFERON GAMMA RESPONSE; (M5932)HALLMARK INFLAMMATORY RESPONSE |
| ERAP2 | endoplasmic reticulum aminopeptidase 2 | GO:0019885 antigen processing and presentation of endogenous peptide antigen via MHC class I;GO:0002483 antigen processing and presentation of endogenous peptide antigen;GO:0019883 antigen processing and presentation of endogenous antigen | (M5921)HALLMARK COMPLEMENT |
| ITGB3 | integrin subunit beta 3 | GO:0051611 regulation of serotonin uptake;GO:0050748 negative regulation of lipoprotein metabolic process;GO:0038027 apolipoprotein A-I-mediated signaling pathway | (M5897)HALLMARK IL6 JAK STAT3 SIGNALING; (M5946)HALLMARK COAGULATION; (M5942)HALLMARK UV RESPONSE DN |
| COL6A3 | collagen type VI alpha 3 chain | GO:0010951 negative regulation of endopeptidase activity;GO:0010466 negative regulation of peptidase activity;GO:0007517 muscle organ development | (M5909)HALLMARK MYOGENESIS; (M5930)HALLMARK EPITHELIAL MESENCHYMAL TRANSITION |
| ADGRF1 | adhesion G protein-coupled receptor F1 | GO:0032793 positive regulation of CREB transcription factor activity;GO:0007416 synapse assembly;GO:0007613 memory |  |
| MUC5B | mucin 5B, oligomeric mucus/gel-forming |  |  |
| COL15A1 | collagen type XV alpha 1 chain | GO:0001886 endothelial cell morphogenesis;GO:0003382 epithelial cell morphogenesis;GO:0001885 endothelial cell development | (M5905)HALLMARK ADIPOGENESIS; (M5909)HALLMARK MYOGENESIS |
| ROBO2 | roundabout guidance receptor 2 | GO:0050925 negative regulation of negative chemotaxis;GO:0061364 apoptotic process involved in luteolysis;GO:0035480 regulation of Notch signaling pathway involved in heart induction |  |
| MEDAG | mesenteric estrogen dependent adipogenesis | GO:0045600 positive regulation of fat cell differentiation;GO:0045598 regulation of fat cell differentiation;GO:0045597 positive regulation of cell differentiation |  |
| CCL5 | C-C motif chemokine ligand 5 | GO:2000503 positive regulation of natural killer cell chemotaxis;GO:0031584 activation of phospholipase D activity;GO:0033634 positive regulation of cell-cell adhesion mediated by integrin | (M5890)HALLMARK TNFA SIGNALING VIA NFKB; (M5913)HALLMARK INTERFERON GAMMA RESPONSE; (M5921)HALLMARK COMPLEMENT |
| TNFAIP3 | TNF alpha induced protein 3 | GO:0034147 regulation of toll-like receptor 5 signaling pathway;GO:0034148 negative regulation of toll-like receptor 5 signaling pathway;GO:0070429 negative regulation of nucleotide-binding oligomerization domain containing 1 signaling pathway | (M5890)HALLMARK TNFA SIGNALING VIA NFKB; (M5891)HALLMARK HYPOXIA; (M5913)HALLMARK INTERFERON GAMMA RESPONSE |
| SERPINA3 | serpin family A member 3 | GO:0030277 maintenance of gastrointestinal epithelium;GO:0010669 epithelial structure maintenance;GO:0006953 acute-phase response | (M5907)HALLMARK ESTROGEN RESPONSE LATE; (M5953)HALLMARK KRAS SIGNALING UP |
| GZMK | granzyme K | GO:0006508 proteolysis;GO:0019538 protein metabolic process;GO:1901564 organonitrogen compound metabolic process | (M5921)HALLMARK COMPLEMENT |
| FAP | fibroblast activation protein alpha | GO:1902362 melanocyte apoptotic process;GO:0010710 regulation of collagen catabolic process;GO:0060244 negative regulation of cell proliferation involved in contact inhibition | (M5930)HALLMARK EPITHELIAL MESENCHYMAL TRANSITION |
| PTGS2 | prostaglandin-endoperoxide synthase 2 | GO:0010335 response to non-ionic osmotic stress;GO:0071471 cellular response to non-ionic osmotic stress;GO:0032227 negative regulation of synaptic transmission, dopaminergic | (M5890)HALLMARK TNFA SIGNALING VIA NFKB; (M5913)HALLMARK INTERFERON GAMMA RESPONSE; (M5953)HALLMARK KRAS SIGNALING UP |
| OLFM4 | olfactomedin 4 | GO:1900026 positive regulation of substrate adhesion-dependent cell spreading;GO:1900024 regulation of substrate adhesion-dependent cell spreading;GO:0010770 positive regulation of cell morphogenesis involved in differentiation |  |
| MSMB | microseminoprotein beta | GO:0008150 biological_process | (M5906)HALLMARK ESTROGEN RESPONSE EARLY |
| DPT | dermatopontin | GO:0030199 collagen fibril organization;GO:0030198 extracellular matrix organization;GO:0043062 extracellular structure organization |  |
| CPB2 | carboxypeptidase B2 | GO:2000346 negative regulation of hepatocyte proliferation;GO:0003331 positive regulation of extracellular matrix constituent secretion;GO:0010757 negative regulation of plasminogen activation | (M5946)HALLMARK COAGULATION |
| SELE | selectin E | GO:1903238 positive regulation of leukocyte tethering or rolling;GO:1903236 regulation of leukocyte tethering or rolling;GO:0002523 leukocyte migration involved in inflammatory response | (M5932)HALLMARK INFLAMMATORY RESPONSE |
| THBS2 | thrombospondin 2 | GO:0051965 positive regulation of synapse assembly;GO:0051963 regulation of synapse assembly;GO:1901890 positive regulation of cell junction assembly | (M5930)HALLMARK EPITHELIAL MESENCHYMAL TRANSITION |
| EDN1 | endothelin 1 | GO:0031583 phospholipase D-activating G protein-coupled receptor signaling pathway;GO:0042313 protein kinase C deactivation;GO:0043179 rhythmic excitation | (M5890)HALLMARK TNFA SIGNALING VIA NFKB; (M5932)HALLMARK INFLAMMATORY RESPONSE; (M5956)HALLMARK KRAS SIGNALING DN |
| DEPP1 | DEPP1 autophagy regulator | GO:0006914 autophagy;GO:0061919 process utilizing autophagic mechanism;GO:0010506 regulation of autophagy |  |
| CD163 | CD163 molecule | GO:0006953 acute-phase response;GO:0002526 acute inflammatory response;GO:0006897 endocytosis |  |
| ANKRD1 | ankyrin repeat domain 1 | GO:0070528 protein kinase C signaling;GO:0043517 positive regulation of DNA damage response, signal transduction by p53 class mediator;GO:0035994 response to muscle stretch |  |
| ITGBL1 | integrin subunit beta like 1 | GO:0033627 cell adhesion mediated by integrin;GO:0007229 integrin-mediated signaling pathway;GO:0007160 cell-matrix adhesion | (M5953)HALLMARK KRAS SIGNALING UP |
| IDO1 | indoleamine 2,3-dioxygenase 1 | GO:0036269 swimming behavior;GO:0034275 kynurenic acid metabolic process;GO:0034276 kynurenic acid biosynthetic process | (M5913)HALLMARK INTERFERON GAMMA RESPONSE |
| ROPN1L | rhophilin associated tail protein 1 like | GO:0048240 sperm capacitation;GO:0003351 epithelial cilium movement involved in extracellular fluid movement;GO:0006858 extracellular transport |  |
| TSPAN1 | tetraspanin 1 | GO:0050821 protein stabilization;GO:0031647 regulation of protein stability;GO:0065008 regulation of biological quality | (M5953)HALLMARK KRAS SIGNALING UP |
| CHI3L2 | chitinase 3 like 2 | GO:0006030 chitin metabolic process;GO:0006032 chitin catabolic process;GO:1901072 glucosamine-containing compound catabolic process |  |
