# Supplementary material for: FAM171B as a Novel Biomarker Mediates Tissue Immune Microenvironment in Pulmonary Arterial Hypertension
Source: Mediators Inflamm. 2022 Sep 22;2022:1878766. doi: 10.1155/2022/1878766 (PMC9553458; doi:10.1155/2022/1878766)
Supplement: Supplementary Materials — Supplementary Table 1: The results of differentially expressed genes (DEGs). Supplementary Table 2: Gene Ontology (GO) enrichment analysis results of differentially expressed genes (DEGs). Supplementary Table 3: Kyoto Encyclopedia of Genes and Genomes (KEGG) enrichment analysis results of differentially expressed genes (DEGs). Supplementary Table 4: Disease Ontology (DO) enrichment analysis results of differentially expressed genes (DEGs). Supplementary Table 5: Metascape function analysis results of differentially expressed genes (DEGs). Supplementary Table 6: results of Gene Set Enrichment Analysis (GSEA) of gene expression matrix. Supplementary Table 7: results of all genes in brown module. Supplementary Table 8: results of key genes in brown module. Supplementary Table 9: results of analyzing the combined data matrix of GSE113439 and GSE117261 using CIBERSORT. Supplementary Table 10: results of the correlation of FAM171B with immune cells. [file 1878766.f1.zip › Supplementary Table6.docx]

| ID | enrichmentScore | NES | pvalue | p.adjust | core_enrichment |
| --- | --- | --- | --- | --- | --- |
| GO |  |  |  |  |  |
| GOCC_AZUROPHIL_GRANULE | -0.571793567 | -2.269752515 | 1.00E-10 | 1.14E-07 | TOLLIP/ELANE/GUSB/FTL/CYB5R3/ARSA/NDUFC2/HEXA/PYGB/DEFA4/FUCA1/STX3/GLB1/CAP1/NAPRT/VAMP8/ANXA11/CREG1/RETN/SURF4/GRN/ADA2/ACLY/GALNS/HEBP2/ARHGAP45/GLA/TOM1/NFAM1/UNC13D/ABCA13/CKAP4/PRKCD/DDOST/MAN2B1/NCSTN/FUCA2/DNAJC5/LAMP1/TUBB4B/LAMTOR1/RAB5C/ARL8A/CPPED1/IMPDH1/CTSA/SNAP29/CD68/BRI3/RNASE3/PYCARD/RAB3D/ARG1/GM2A/MGST1/TRAPPC1/ACP3/PADI2/VNN1/VAT1/CEACAM6/STXBP2/FABP5/SERPINB3/PIGR/MNDA/GCA/PLAC8/C3AR1/FPR1/DEFA1B/SERPINA3/RNASE2 |
| GOCC_MITOCHONDRIAL_PROTEIN_CONTAINING_COMPLEX | -0.546332821 | -2.303989993 | 1.00E-10 | 1.14E-07 | COX7C/ATP5F1A/MICOS10/UQCC3/MRPL22/NDUFB1/MRPS12/NDUFA13/TIMM10B/MALSU1/PNPT1/MRPL13/NDUFA1/MRPL48/MRPS17/SDHAF4/NDUFV2/UQCRHL/TIMM10/MRPL46/NDUFS7/TRMT10C/POLRMT/PDHB/UQCRFS1/MRPL20/UQCRC1/TIMM13/MRPL45/NDUFB11/MRPS28/NDUFC2/TIMM50/MRPL30/MRPL17/COX4I1/PDK2/NDUFC2-KCTD14/MRPS21/MRPL12/BAX/TIMM29/ATP5F1EP2/NDUFA7/NDUFA4/ATP5F1E/C15orf48/MRPL14/NDUFA4L2/MRPL16/MRPL40/MRPL24/DNAJC11/MRPL9/MRPS35/MRPL23/SDHC/MRPL35/MRPL49/TIMM17A/NDUFS8/NDUFAB1/NDUFA6/MRPL21/UQCR10/MRPS36/NDUFB6/SDHD/NDUFV3/MRPS2/PHB2/FOXRED1/MRPS33/UQCRQ/MRPL34/ATP5F1D/MTX1/ROMO1/MRPL4/MRPL18/SDHB/NDUFB4/MRPL41/ATP5MPL/TOMM22/BCKDK/CHCHD10/MRPL58/MRPS18C/NDUFB9/ATP5PF/APOO/NDUFA12/MRPL57/NDUFS2/MRPL11/ATP5ME/CYC1/ATP5PO/MPV17L2/SMDT1/NDUFA2/ATP5PD/ATP5MD/NDUFB3/MRPS11/BCS1L/DLAT/NDUFB7/ATP5MC1/NDUFV1/MRPL33/HSD17B10/MRPL53/TOMM40/MRPL28/MRPS15/MRPS16/COX4I2/ATP5MF/MRPL37/MRPS18B/TIMM8B/MRPS34/NDUFAF1/COX5A/PHB/NDUFB10/BCKDHA/DMAC2 |
| GOCC_RESPIRASOME | -0.657588368 | -2.416633557 | 1.00E-10 | 1.14E-07 | UQCC3/NDUFB1/NDUFA13/NDUFA1/SDHAF4/NDUFV2/UQCRHL/NDUFS7/UQCRFS1/UQCRC1/NDUFB11/NDUFC2/COX8A/COX4I1/COA6/NDUFC2-KCTD14/OXA1L/NDUFA7/NDUFA4/C15orf48/NDUFA4L2/SDHC/COX7A2L/SURF1/NDUFS8/NDUFAB1/NDUFA6/UQCR10/NDUFB6/SDHD/NDUFV3/FOXRED1/UQCRQ/COX7A2/UQCR11/COX7A1/SDHB/NDUFB4/HIGD2A/NDUFB9/NDUFA12/NDUFS2/CYC1/NDUFA2/NDUFB3/BCS1L/NDUFB7/NDUFV1/COX4I2/NDUFAF1/RAB5IF/COX5A/COX7B/NDUFB10/COX6B1/DMAC2 |
| GOCC_SECRETORY_GRANULE_MEMBRANE | -0.472601325 | -2.014573407 | 1.00E-10 | 1.14E-07 | TCIRG1/CYB5R1/RAB5B/TOM1/SLC44A2/PTAFR/CD109/NFAM1/HVCN1/FPR2/PTPRJ/TNFRSF1B/ABCA13/CKAP4/STING1/DOK3/DDOST/STK10/NCSTN/DNAJC5/LAMP1/LAMTOR1/CD33/PLAU/RHOF/RAB5C/BST1/IZUMO1/TLR2/ARL8A/PLA1A/CD53/ITGAL/ALDH3B1/SNAP29/CD68/BRI3/CD300A/RAB7A/RAB3D/SIRPB1/SIRPA/MMP25/MGST1/ACP3/CLEC5A/SIGLEC14/VNN1/SIGLEC5/SLC11A1/RHOG/CEACAM1/CEACAM6/C5AR1/TSPAN14/PECAM1/ADGRG3/FABP5/PIGR/KCNAB2/SELL/LAIR1/ITGB2/CD177/FCER1G/LILRB2/ITGAX/SIGLEC9/LILRA3/C3AR1/ITGAM/FPR1/CA4/CD14/CXCR2/CR1/LILRB3/ANPEP/CXCR1/MGAM |
| GOCC_VESICLE_LUMEN | -0.456136167 | -1.969699987 | 1.00E-10 | 1.14E-07 | CANT1/ELANE/GUSB/PPIE/FTL/CYB5R3/ARSA/PYGB/ALAD/TCN1/DEFA4/ACAA1/PGLYRP1/TOR4A/SERPINB1/BIN2/SERPINA1/FUCA1/OSTF1/GHRL/GLB1/MIF/POMC/CAP1/NAPRT/MVP/TGFB1/PSMC3/CREG1/RETN/ITIH3/DERA/GRN/PPIA/VEGFB/ADA2/PSMD11/VEGFC/PSMB1/GHDC/ACLY/CYFIP1/OSCAR/ARHGAP9/COMMD9/GALNS/ALDOA/PSMD3/PNP/COTL1/HEBP2/GYG1/ARHGAP45/PGAM1/GLA/CDA/LRG1/CSTB/QSOX1/UNC13D/HP/SLPI/GSTP1/CTSD/PSMB7/PRKCD/S100P/MAN2B1/FUCA2/TUBB4B/GRP/DBNL/TIMP3/CPPED1/IMPDH1/CTSA/GMFG/PTX3/PDGFB/F5/ORM1/NEU1/CNN2/RNASE3/PYCARD/ARG1/VEGFD/TIMP1/AMPD3/GM2A/IDH1/PKM/ALDOC/FCN1/PTPN6/TRAPPC1/DNASE1L1/PADI2/FERMT3/S100A11/QPCT/VAT1/NME2/PYGL/CFP/FABP5/SERPINB3/MNDA/GCA/LTF/MMP8/HK3/OLFM4/PLAC8/DEFA1B/FGR/SERPINA3/CHIT1/S100A8/LCN2/RNASE2/S100A9/S100A12 |
| GOMF_EXTRACELLULAR_MATRIX_STRUCTURAL_CONSTITUENT | 0.629741725 | 2.328676435 | 1.00E-10 | 1.14E-07 | POSTN/COL14A1/ASPN/OGN/LTBP1/HMCN1/MXRA5/TFPI2/COL6A6/MATN2/MFAP4/PRG4/COL15A1/LUM/COL6A3/FRAS1/THBS2/DPT/THBS1/MFAP5/COL3A1/LTBP2/FGL2/FBN1/CCN1/MGP/LAMA3/COL1A1/COL5A2/IGFBP7/TNXB/MFAP2/DCN/COL12A1/ANOS1/COL4A3/MMRN1/COL1A2/FBLN2/EDIL3/NID2/COL4A1/FGG/ABI3BP/LAMA4/LTBP4/EFEMP2/LAMB1/FN1/EFEMP1/SPARC/FBLN1/COL18A1 |
| GOCC_SPECIFIC_GRANULE | -0.549945124 | -2.195272537 | 1.20E-10 | 1.17E-07 | CD93/CXCL1/CLEC12A/JUP/RAP2B/SLC15A4/TOLLIP/CANT1/ELANE/PTPRB/AGPAT2/MCEMP1/TCN1/P2RX1/DEFA4/ACAA1/PGLYRP1/ADAM8/GPR84/STX3/AP1M1/DGAT1/LAMTOR2/VAMP8/ANXA11/SLC27A2/RETN/CYBA/GHDC/CYFIP1/OSCAR/TRPM2/ANXA3/CYBB/TOM1/SLC44A2/LRG1/QSOX1/HVCN1/FPR2/HP/PTPRJ/TNFRSF1B/SLPI/CKAP4/CTSD/STK10/DNAJC5/LAMTOR1/CD33/PLAU/BST1/CD53/ITGAL/PTX3/ALDH3B1/ORM1/NEU1/CNN2/ARG1/MMP25/PTPN6/DNASE1L1/CLEC5A/QPCT/CFP/CEACAM1/TSPAN14/STXBP2/ADGRG3/KCNAB2/LAIR1/ITGB2/CD177/LTF/MMP8/OLFM4/LILRA3/C3AR1/ITGAM/CHIT1/LCN2 |
| GOBP_OXIDATIVE_PHOSPHORYLATION | -0.57810701 | -2.276613684 | 1.62E-10 | 1.38E-07 | NDUFS3/ATPSCKMT/MLXIPL/ANTKMT/NDUFB8/COX6A1/NIPSNAP2/COX7C/ATP5F1A/BID/UQCC3/NDUFB1/NDUFA13/NDUFA1/TEFM/NDUFV2/UQCRHL/NDUFS7/DMAC2L/UQCRFS1/COX10/UQCRC1/NDUFB11/NDUFC2/COX8A/COX4I1/ABCD1/COA6/NDUFC2-KCTD14/STOML2/COQ9/NDUFA7/NDUFA4/ATP5F1E/TAZ/SDHC/COX7A2L/SURF1/NDUFS8/NDUFAB1/NDUFA6/PINK1/DNAJC30/UQCR10/SDHAF2/NDUFB6/SDHD/NDUFV3/UQCRQ/GHITM/ATP5F1D/COX7A2/UQCR11/COX7A1/NDUFB4/CHCHD10/NDUFB9/ATP5PF/NDUFA12/NDUFS2/ATP5ME/CYC1/ATP5PO/NDUFA2/ATP5PD/NDUFB3/DGUOK/NDUFB7/ATP5MC1/NDUFV1/COX4I2/COX5B/ATP5MF/NDUFAF1/COX5A/COX7B/NDUFB10/COX6B1/UQCC2/NUPR1/SHMT2 |
| GOBP_EXTERNAL_ENCAPSULATING_STRUCTURE_ORGANIZATION | 0.464660771 | 1.855482366 | 3.82E-10 | 2.89E-07 | POSTN/SFRP2/VCAM1/COL14A1/CCDC80/HAS2/ITGA2/ECM2/SULF1/ADAMTS9/ITGB3/CPB2/TLL1/MFAP4/COL15A1/FAP/LUM/ITGB6/COL6A3/ANTXR1/DPT/MMP7/PHLDB2/ITGB8/ITGA9/CTSK/FLRT2/BMP1/THBS1/MFAP5/COL3A1/CSGALNACT1/FBN1/CCN2/ITGA4/CCN1/TPSAB1/DDR2/MMP16/ITGAV/NF1/LAMA3/MYH11/COL1A1/HPSE2/COL5A2/SERPINE1/DPP4/ADAMTSL1/MYO1E/LOX/TNXB/MFAP2/DCN/COL12A1/ADAM12/ADTRP/COL4A3/CLASP2/COL1A2/TNR/FBLN2/ATP7A/ADAMTS12/SMAD3/RIC1/EGFL6/ADAMTS3/LTBP3/HTRA1/NID2/COL4A1/PDGFA/FGG/NDNF/ADAMTS6/LAMA4/ITGA8/KLK7/EFEMP2/ITGA6/CD44/LAMB1/FGF2/CD47/FN1/JAM3/ICAM1/LOXL2/CTSG/TGFB2/ADAMTS4/ITGB1/SPARC/FBLN1/COL18A1 |
| GOCC_OXIDOREDUCTASE_COMPLEX | -0.610982196 | -2.301856342 | 6.15E-10 | 4.19E-07 | UQCC3/NDUFB1/NDUFA13/NOXA1/NDUFA1/SDHAF4/NDUFV2/UQCRHL/CYB5B/NDUFS7/GPD1L/PDHB/UQCRFS1/CYB5R3/UQCRC1/NDUFB11/NDUFC2/PDK2/GMPR2/NDUFC2-KCTD14/NDUFA7/NDUFA4/SDHC/NDUFS8/NDUFAB1/NDUFA6/UQCR10/MRPS36/CYBA/NDUFB6/SDHD/NDUFV3/P4HB/FOXRED1/UQCRQ/GCSH/SDHB/CYBB/NDUFB4/BCKDK/NDUFB9/NDUFA12/NDUFS2/CYC1/NDUFA2/NDUFB3/BCS1L/DLAT/NDUFB7/NDUFV1/NCF1/MTARC1/OGDH/NDUFAF1/DUOX1/NCF2/NDUFB10/BCKDHA/DMAC2/NCF4 |
| GOCC_COLLAGEN_CONTAINING_EXTRACELLULAR_MATRIX | 0.450487215 | 1.810296523 | 7.17E-10 | 4.44E-07 | POSTN/SFRP2/COL14A1/ASPN/CCDC80/OGN/ANGPT2/CPA3/LTBP1/HMCN1/MXRA5/SULF1/COL6A6/ADAMTS9/CXCL12/SERPINE2/FGFR2/MATN2/FREM1/CDON/MFAP4/PRG4/COL15A1/LUM/COL6A3/FRAS1/THBS2/DPT/TGFB3/FREM2/GPC6/THBS1/MFAP5/COL3A1/LTBP2/FGL2/FBN1/CCN2/GPC3/CCN1/TPSAB1/DST/CASK/P3H2/MGP/LAMA3/COL1A1/ANGPTL2/COL5A2/SERPINE1/IGFBP7/TNXB/MFAP2/DCN/COL12A1/SFRP1/RARRES2/COL4A3/CLC/CTSF/MMRN1/CDH2/COL1A2/TNR/FBLN2/EDIL3/EGFL6/ADAMTS3/LTBP3/ATRNL1/HTRA1/NID2/COL4A1/ITIH5/FGG/ABI3BP/LAMA4/LTBP4/ANGPTL1/ANGPT1/EFEMP2/LAMB1/ANXA1/IL7/FN1/ICAM1/LOXL2/CTSG/DLG1/TGFB2/ADAMTS4/EFEMP1/SPARC/FBLN1/COL18A1/FGF10/F3/CMA1/ANXA4/ADAMTS5 |
| GOCC_RESPIRATORY_CHAIN_COMPLEX | -0.665113888 | -2.361540819 | 8.69E-10 | 4.91E-07 | COX7C/UQCC3/NDUFB1/NDUFA13/NDUFA1/SDHAF4/NDUFV2/UQCRHL/NDUFS7/UQCRFS1/UQCRC1/NDUFB11/NDUFC2/COX8A/COX4I1/COA6/NDUFC2-KCTD14/NDUFA7/NDUFA4/C15orf48/NDUFA4L2/SDHC/NDUFS8/NDUFAB1/NDUFA6/UQCR10/NDUFB6/SDHD/NDUFV3/FOXRED1/UQCRQ/SDHB/NDUFB4/NDUFB9/NDUFA12/NDUFS2/CYC1/NDUFA2/NDUFB3/BCS1L/NDUFB7/NDUFV1/COX4I2/NDUFAF1/COX5A/COX7B/NDUFB10/COX6B1/DMAC2 |
| GOMF_ATPASE_ACTIVITY | 0.435936915 | 1.767348912 | 9.37E-10 | 4.91E-07 | ABCG2/ABCC9/ABCB1/ABCA8/ATP2B1/MYH10/ATAD2/MACF1/SMARCA1/ABCA9/SHPRH/ATRX/ABCA6/WRN/ABCA10/ACSL4/NAV3/BTAF1/SMC6/TOP2B/HLTF/CHD6/KIF16B/DDX58/KIF3A/SMCHD1/DDX60/HSPA1B/ATP11A/CHD1/ATP11C/HSPA1A/MYO1E/CHD2/MFSD2A/KIF13A/HSP90AA1/ATP11B/ASCC3/ATP8B1/MYO1B/ATAD2B/MSH2/ERCC6/ATP10D/ATP13A3/DDX5/ATP7A/ABCG1/RAD51B/HELB/DHX36/IFIH1/SMARCA5/ATP2C1/RFC1/CHD7/KIF23/DDX10/DHX15/DYNC2H1/ERCC6L2/CHD1L/YTHDC2/KIF21A/MSH6/KIF13B/SUPV3L1/BPTF/ABCC4/SETX/ATP10A/MCM6/ABCD2/ACSL3/EIF4A2/DDX46/HSPA13/DDX60L/DHX9/VWA8/ABCE1/MSH3/MYO5A/DDX17/SPATA5/HELZ/ANXA1/KIF27/RHOBTB3/DDX47/SMARCA2/ATP9B/LONP2/MTREX/MDN1/DDX3X/KIF15/ABCA5/MCM9/KIF5B/KIF20A/MLH3/CENPE/DDX24/MYO6/NAV2/HSPA6/KIF20B/KIF5C/DHX32/KIF18A/ABCD3/CFTR/HSPD1/DICER1/KIF2A/DDX6/RAD50/TMEM30A/CHD9/PEX1/ATP9A/ABCB7/TTF2/AFG1L/DDX27/HSPA8/MYO5C/IQCA1/RAD54B/MRE11/TOP2A/DDX21/ATP8A1/DHX35/CCT8/DDX42/DDX50/KIF14/DNA2/ATF7IP/DYNC1H1/SLFN11/SNRNP200/DDX18/RECQL/DHX40/HELLS/KATNAL1/SMARCAD1/DSCC1/KATNA1/ATP1A2/ATAD1/ATP13A5/CHD3/YME1L1 |
| GOBP_MITOCHONDRIAL_TRANSLATION | -0.572345184 | -2.240546288 | 1.19E-09 | 5.81E-07 | DAP3/NOA1/AARS2/MRPL36/TSFM/MRPL38/MRPL10/MRPL27/GATC/TUFM/MRPL22/GADD45GIP1/MRPS12/MALSU1/MRPL13/MRPL48/MRPS17/GARS1/MRPL46/TRMT10C/CHCHD1/DARS2/MRPL20/MRPL45/MRPS28/MRPL30/MTRF1L/MRPL17/MTG1/MTIF2/MRPS21/MRPL12/OXA1L/C1QBP/NDUFA7/MRPL14/ERAL1/MRPS23/MRPL16/MRPL40/MRPL24/MRPL9/MRPS35/MRPL23/MRPL35/MRPL49/MRPL21/MRPS36/TARS2/MRPS2/MRPS25/SARS2/MRPS33/RPUSD3/MRPL34/MTG2/MRPL4/MRPL18/MRPL41/MRPL58/MRPS18C/MRPL57/TACO1/MRPL11/MPV17L2/AURKAIP1/MRPS11/MRPL33/MRPL53/MRPL28/MRPS15/MRPS16/MRPL37/MRPS18B/MRPS34/TRUB2/UQCC2/SHMT2 |
| GOCC_TERTIARY_GRANULE | -0.52511381 | -2.092876678 | 2.65E-09 | 1.20E-06 | GOLGA7/SERPINB6/NBEAL2/CYBA/CYFIP1/OSCAR/TRPM2/CYSTM1/ALDOA/CYBB/TCIRG1/CDA/PTAFR/LRG1/CSTB/QSOX1/FPR2/HP/CTSD/DOK3/LAMP1/LAMTOR1/CD33/PLAU/DBNL/ARL8A/CD53/PTX3/ORM1/CNN2/CD300A/SIRPA/IDH1/ALDOC/MMP9/PTPN6/CLEC5A/SIGLEC14/SIGLEC5/SLC11A1/QPCT/CFP/CEACAM1/METTL7A/TSPAN14/STXBP2/KCNAB2/LAIR1/ITGB2/CD177/LTF/MMP8/FCER1G/OLFM4/LILRB2/ITGAX/LILRA3/ITGAM/FPR1/CR1/MGAM/CHIT1 |
| GOCC_MITOCHONDRIAL_MATRIX | -0.386033496 | -1.713710766 | 4.63E-09 | 1.88E-06 | GSTK1/PARS2/IDH3A/CHPF/DAP3/HOGA1/FECH/SOD1/CS/NDUFS3/GOT2/MECR/HSPA1L/MRPL36/HYKK/SHC1/TSFM/IVD/NDUFB8/IBA57/MRPL38/MRPL10/MRPL27/RPS3/PRDX5/NUDT13/AASS/FDXR/ATAD3A/ECHS1/TXNRD2/METTL17/ATP5F1A/TUFM/QARS1/PDP2/SLC25A5/MTHFS/HADH/MRPL22/NUDT2/GADD45GIP1/MRPS12/DHX30/NFS1/ATG4D/MALSU1/PNPT1/MRPL13/MRPL48/MRPS17/DDX28/GARS1/TEFM/SDHAF4/FLAD1/MPG/MRPL46/ME2/GSTZ1/PUS1/ETFB/NDUFS7/PRDX3/DNAJA3/LACTB2/TRMT10C/TBRG4/POLRMT/CHCHD1/PDHB/SDHAF3/DARS2/MRPL20/MYG1/LIPT2/MRPL45/STYXL1/MRPS28/ACSF3/MLYCD/MRPL30/ARL2BP/PYCR1/ACADS/MTRF1L/MRPL17/MTG1/ADPRS/PDK2/BDH1/LRRC59/ACOT2/ISCA2/IDH3B/DHFR2/MRPS21/ACAD10/MRPL12/OXA1L/GCDH/MCEE/C1QBP/NDUFA7/MTRES1/SARDH/ATP5F1E/TP53/ACO2/ECI1/MRPL14/ACAD8/ACP6/ERAL1/CCAR2/PYCR2/MRPL16/MRPL40/MIPEP/MRPL24/MRPL9/MRPS35/MRPL23/ACSM5/MRPL35/MPST/MRPL49/NDUFS8/SUOX/ALAS1/NDUFAB1/ADHFE1/FPGS/HAGH/MRPL21/PCCB/SIRT5/SDHAF2/MRPS36/TARS2/MRPS2/SARS2/MRPS33/RPUSD3/PPM1K/MRM2/MRPL34/ALDH4A1/ATP5F1D/CLPP/ARL2/MTG2/TIMM44/ABAT/MRPL4/LONP1/MRPL18/GCSH/MRPL41/DTYMK/IDH3G/NAXE/BCKDK/MRPL58/MRPS18C/TXN2/MRPL57/ALDH6A1/NDUFS2/POLDIP2/MRPL11/FAHD1/ELAC2/MPV17L2/SMDT1/MRPS11/DLAT/DGUOK/HMGCL/COASY/MCAT/MRPL33/HSD17B10/MRPL53/MRPL28/MRPS15/MRPS16/MRPL37/OGDH/MRPS18B/RIDA/IDH2/PCK2/DHTKD1/CYP27A1/MMAB/MRPS34/NDUFAF1/MDH2/TRUB2/GSR/BCL2L1/BLOC1S1/PDSS1/PRODH/UQCC2/BCKDHA/BCAT2/TST/ACSS2/SHMT2 |
| GOCC_INNER_MITOCHONDRIAL_MEMBRANE_PROTEIN_COMPLEX | -0.555250565 | -2.172207223 | 4.68E-09 | 1.88E-06 | COX7C/ATP5F1A/MICOS10/UQCC3/NDUFB1/NDUFA13/TIMM10B/NDUFA1/SDHAF4/NDUFV2/UQCRHL/TIMM10/NDUFS7/UQCRFS1/UQCRC1/NDUFB11/NDUFC2/TIMM50/COX4I1/NDUFC2-KCTD14/TIMM29/ATP5F1EP2/NDUFA7/NDUFA4/ATP5F1E/C15orf48/NDUFA4L2/DNAJC11/SDHC/TIMM17A/NDUFS8/NDUFAB1/NDUFA6/UQCR10/NDUFB6/SDHD/NDUFV3/PHB2/FOXRED1/UQCRQ/ATP5F1D/MTX1/ROMO1/SDHB/NDUFB4/ATP5MPL/CHCHD10/NDUFB9/ATP5PF/APOO/NDUFA12/NDUFS2/ATP5ME/CYC1/ATP5PO/SMDT1/NDUFA2/ATP5PD/ATP5MD/NDUFB3/BCS1L/NDUFB7/ATP5MC1/NDUFV1/COX4I2/ATP5MF/NDUFAF1/COX5A/PHB/NDUFB10/DMAC2 |
| GOCC_ORGANELLAR_RIBOSOME | -0.625412317 | -2.281794366 | 6.89E-09 | 2.61E-06 | MRPL22/MRPS12/MALSU1/MRPL13/MRPL48/MRPS17/MRPL46/CHCHD1/MRPL20/MRPL45/MRPS28/MRPL30/MRPL17/MTG1/MRPS21/MRPL12/NDUFA7/MRPL14/MRPL16/MRPL40/MRPL24/MRPL9/MRPS35/MRPL23/MRPL35/MRPL49/NDUFAB1/MRPL21/MRPS36/MRPS2/MRPS33/MRPL34/MTG2/MRPL4/MRPL18/MRPL41/MRPL58/MRPS18C/MRPL57/MRPL11/MPV17L2/MRPS11/MRPL33/MRPL53/MRPL28/MRPS15/MRPS16/MRPL37/MRPS18B/MRPS34 |
| GOBP_MITOCHONDRIAL_GENE_EXPRESSION | -0.513380259 | -2.053959719 | 8.62E-09 | 3.00E-06 | GATC/TUFM/MRPL22/GADD45GIP1/MRPS12/MALSU1/PNPT1/MRPL13/MRPL48/MRPS17/GARS1/TEFM/MRPL46/PUS1/TRMT10C/TBRG4/POLRMT/PPARGC1B/CHCHD1/DARS2/MRPL20/MRPL45/MRPS28/MRPL30/MTRF1L/MRPL17/MTG1/MTIF2/MRPS21/MRPL12/OXA1L/C1QBP/NDUFA7/MTRES1/MRPL14/ERAL1/MRPS23/MRPL16/MRPL40/MRPL24/MRPL9/MRPS35/MRPL23/MRPL35/MRPL49/MRPL21/MRPS36/TARS2/MRPS2/MRPS25/SARS2/MRPS33/RPUSD3/MRPL34/MTG2/MRPL4/MRPL18/MRPL41/CHCHD10/MRPL58/MRPS18C/MRPL57/TACO1/MRPL11/ELAC2/MPV17L2/AURKAIP1/MRPS11/MRPL33/HSD17B10/MRPL53/MRPL28/MRPS15/MRPS16/MRPL37/MRPS18B/MRPS34/TRUB2/UQCC2/SHMT2 |
| GOBP_MITOCHONDRIAL_TRANSLATIONAL_TERMINATION | -0.616871134 | -2.256966854 | 8.81E-09 | 3.00E-06 | MRPL22/GADD45GIP1/MRPS12/MRPL13/MRPL48/MRPS17/MRPL46/CHCHD1/MRPL20/MRPL45/MRPS28/MRPL30/MTRF1L/MRPL17/MRPS21/MRPL12/OXA1L/MRPL14/ERAL1/MRPS23/MRPL16/MRPL40/MRPL24/MRPL9/MRPS35/MRPL23/MRPL35/MRPL49/MRPL21/MRPS36/MRPS2/MRPS25/MRPS33/MRPL34/MRPL4/MRPL18/MRPL41/MRPL58/MRPS18C/MRPL57/MRPL11/AURKAIP1/MRPS11/MRPL33/MRPL53/MRPL28/MRPS15/MRPS16/MRPL37/MRPS18B/MRPS34 |
| GOBP_DEFENSE_RESPONSE_TO_BACTERIUM | -0.449361928 | -1.900490111 | 1.00E-08 | 3.25E-06 | TNF/H2BC7/H2BC12/H2BC21/FCN2/TNFRSF1A/KRT6A/ANG/GRN/NOD2/HAMP/HLA-E/CYBA/H2BC4/CCL20/ANXA3/ROMO1/IRF8/SIRT2/WFDC12/TLR5/FPR2/SYK/HP/PRB3/GNLY/SLPI/PRKCD/CEBPB/TLR2/MAVS/BCL3/SIGLEC16/RNASE3/PYCARD/MYD88/NAIP/TLR4/S100A14/SLC11A1/WFDC2/TLR6/CFP/STAB1/C5AR1/RNASE6/TREM2/MPEG1/PPP1R11/NLRC4/LTF/AQP1/PI3/SLAMF8/FCER1G/PLAC8/DEFA1B/FGR/S100A8/LCN2/BPIFA1/S100A9/S100A12 |
| GOMF_ELECTRON_TRANSFER_ACTIVITY | -0.547537032 | -2.131155795 | 1.86E-08 | 5.76E-06 | NDUFB1/COX11/ASPH/NDUFA13/NDUFA1/AOX1/NDUFV2/UQCRHL/AKR1B1/CYB5B/ME2/ETFB/P4HA2/NDUFS7/UQCRFS1/COX10/UQCRC1/NDUFC2/ME1/COX8A/COX4I1/NDUFC2-KCTD14/IDH3B/NDUFA7/NDUFA4/C15orf48/SRD5A1/NDUFA4L2/GLRX/SDHC/COX7A2L/SURF1/NDUFS8/NDUFAB1/NDUFA6/UQCR10/CYBA/NDUFB6/SDHD/NDUFV3/UQCRQ/ALDH4A1/CYB5A/COX7A2/UQCR11/COX7A1/SDHB/CYBB/NDUFB4/GFUS/NDUFB9/NDUFA12/NDUFS2/NDUFA2/NDUFB3/NDUFB7/NDUFV1/NCF1/AIFM2/COX4I2/COX5B/AKR1A1/GSR/COX5A/NCF2/COX7B/NDUFB10/COX6B1/GPX2/DHRS3/NQO2/PHGDH |
| GOMF_PROTON_TRANSMEMBRANE_TRANSPORTER_ACTIVITY | -0.563737531 | -2.178867641 | 2.02E-08 | 5.99E-06 | DMAC2L/ATP6V0C/SLC15A4/ATP6V0E2/SLC33A1/COX10/COX8A/COX4I1/SLC2A9/ATP6V1C2/ATP6V1A/ATP5F1EP2/NDUFA4/ATP5F1E/C15orf48/NDUFA4L2/COX7A2L/ATP6V1B2/SURF1/TMCO3/SLC17A5/ATP2A3/MFSD3/SLC46A1/CYB5A/ATP5F1D/COX7A2/COX7A1/SLC4A11/TCIRG1/ATP12A/ATP5PF/ATP6V0D2/HVCN1/ATP5ME/SLC15A2/ATP5PO/ATP5PD/ATP6V0B/SLC9A8/COX4I2/SLC39A8/COX5B/ATP5MF/ATP6V0D1/SLC2A10/COX5A/ATP6V1F/COX7B/SLC11A1/COX6B1/SLC36A1 |
| GOBP_ATP_SYNTHESIS_COUPLED_ELECTRON_TRANSPORT | -0.608250267 | -2.232036584 | 4.06E-08 | 1.15E-05 | COX7C/BID/UQCC3/NDUFB1/NDUFA13/NDUFA1/NDUFV2/UQCRHL/NDUFS7/UQCRFS1/COX10/UQCRC1/NDUFB11/NDUFC2/COX8A/COX4I1/COA6/NDUFC2-KCTD14/COQ9/NDUFA7/NDUFA4/TAZ/SDHC/COX7A2L/NDUFS8/NDUFAB1/NDUFA6/PINK1/UQCR10/SDHAF2/NDUFB6/SDHD/NDUFV3/UQCRQ/GHITM/UQCR11/NDUFB4/NDUFB9/NDUFA12/NDUFS2/CYC1/NDUFA2/NDUFB3/DGUOK/NDUFB7/NDUFV1/COX4I2/COX5B/NDUFAF1/COX5A/COX7B/NDUFB10/COX6B1 |
| GOBP_CELLULAR_RESPIRATION | -0.489028604 | -1.963308315 | 5.14E-08 | 1.40E-05 | COX7C/MTFR1/BID/UQCC3/NDUFB1/NDUFA13/ETFRF1/PNPT1/NDUFA1/SDHAF4/NDUFV2/UQCRHL/ME2/ETFB/NDUFS7/PDHB/UQCRFS1/COX10/UQCRC1/NDUFB11/NDUFC2/COX8A/MDH1/COX4I1/MDH1B/COA6/CISD1/NDUFC2-KCTD14/IDH3B/COQ9/OXA1L/NDUFA7/NDUFA4/ACO2/MYBBP1A/BNIP3/TAZ/SDHC/COQ10A/COX7A2L/SURF1/NDUFS8/NDUFAB1/NDUFA6/PINK1/UQCR10/SDHAF2/MTFR1L/NDUFB6/SDHD/NDUFV3/UQCRQ/GHITM/SLC25A22/ATP5F1D/UQCR11/SDHB/NDUFB4/IDH3G/NDUFB9/CBFA2T3/NDUFA12/NDUFS2/CYC1/FAHD1/NDUFA2/NDUFB3/DLAT/DGUOK/NDUFB7/NDUFV1/AIFM2/COX4I2/COX5B/OGDH/IDH2/DHTKD1/PRELID1/NDUFAF1/MDH2/COX5A/BLOC1S1/IDH1/COX7B/NDUFB10/COX6B1/SHMT2 |
| GOMF_OXIDOREDUCTASE_ACTIVITY_ACTING_ON_CH_OH_GROUP_OF_DONORS | -0.536028251 | -2.085684563 | 6.99E-08 | 1.83E-05 | HSD11B2/UEVLD/HSD17B4/CRYL1/LDHD/ADH1C/HSD17B12/AKR1C1/HADH/LDHA/ADH1A/IMPDH2/AKR1B1/ME2/AKR1E2/HSD17B13/PTGR2/ME1/MDH1/CHDH/MDH1B/BDH1/IDH3B/GRHPR/CBR3/HSD17B2/SORD/DHRS9/ADHFE1/SRD5A2/CTBP1/PTGR1/HMGCR/SPR/GFUS/IDH3G/PRXL2B/ADH7/HSD17B10/HSD17B14/NSDHL/AKR1A1/IMPDH1/H6PD/IDH2/DHRS13/MDH2/IDH1/CBR1/ALDH3A1/DCXR/AKR1C3/KCNAB2/PGD/DHRS3/HPGD/G6PD/FASN/AKR1C2/PHGDH |
| GOCC_VACUOLAR_MEMBRANE | -0.383152497 | -1.674777984 | 8.20E-08 | 2.07E-05 | ATP6V1C2/RHEB/DAGLB/HPS6/AP1M1/VAC14/ATP6V1A/ZFYVE26/WDR81/CD1D/BLOC1S2/MFSD12/GABARAP/LAMTOR2/VAMP8/RNF13/MTOR/SLC26A11/ABCC10/ATP6V1B2/SLC2A6/RPTOR/SURF4/GRN/TMEM150B/SLC17A5/PIP4P1/GPR137B/PLD3/SPHK2/TMEM9B/STARD3/WIPI1/AP1S1/TRPM2/AP5B1/VPS4A/TECPR1/SNAPIN/AP3D1/TM9SF1/GNA11/PIP4P2/TCIRG1/RILP/TMEM199/TOM1/SLC44A2/IFITM3/UBA1/NFAM1/ATP6V0D2/VPS11/VPS33B/GPR137/AP5S1/ECE1/ATG16L2/SLC15A3/ABCA13/CKAP4/STING1/CTSD/PCSK9/DDOST/MAN2B1/ATRAID/CLTA/NCSTN/LAPTM5/CLCN6/CYB561A3/VPS18/DNAJC5/AP2M1/LAMP1/LAMTOR1/MARCHF1/ATP6V0B/RAB5C/ELAPOR1/SLC39A8/KXD1/THBD/ARRB1/RRAGD/ARL8A/AP1M2/ATP13A2/CTSA/ATP6V0D1/SNAP29/NAPA/AP2A1/P2RX4/CD68/ABHD6/NEU1/SLC35F6/BRI3/RAB7A/PGAP6/MARCHF2/RAB3D/FLOT1/SLC48A1/BLOC1S1/AP2S1/SCARB1/PSEN2/MCOLN1/ATP6V1F/MGST1/TMEM138/ACP3/ABCB6/CLN3/CLCN7/VNN1/AP1B1/GBA/CEACAM6/ACP2/PIGR/CTNS/GLMP/SLC3A2/SLC66A1L/TLR8/C3AR1/SLC36A1/FPR1/TSPAN1/ANPEP |
| GOMF_OXIDOREDUCTASE_ACTIVITY_ACTING_ON_NAD_P_H | -0.582008917 | -2.139447665 | 1.13E-07 | 2.75E-05 | GMPR/NDUFS3/PGK1/NOX5/NOX1/NDUFB8/TXNRD2/AKR1C1/NDUFB1/NDUFA13/AIFM1/TXNDC17/NDUFA1/NDUFV2/NDUFS7/PRDX3/CYB5R3/NDUFC2/GMPR2/NDUFC2-KCTD14/NDUFA7/NDUFA4/CYB5R4/NDUFS8/NDUFAB1/NDUFA6/CYBA/NDUFB6/NDUFV3/MICAL1/CYBB/TXN/NDUFB4/CYB5R1/NDUFB9/NDUFA12/NDUFS2/NDUFA2/NDUFB3/TP53I3/NDUFB7/NDUFV1/NCF1/POR/AIFM2/GSR/DUOX1/NCF2/NDUFB10/CBR1/DCXR/AKR1C3/NQO2/TXNRD1/NCF4/AKR1C2/NQO1 |
| GOBP_ELECTRON_TRANSPORT_CHAIN | -0.487337566 | -1.947430793 | 1.26E-07 | 2.96E-05 | UQCC3/NDUFB1/COX11/ASPH/NDUFA13/ETFRF1/NDUFA1/AOX1/NDUFV2/UQCRHL/AKR1B1/CYB5B/ME2/ETFB/P4HA2/NDUFS7/UQCRFS1/COX10/UQCRC1/NDUFB11/NDUFC2/ME1/COX8A/COX4I1/COA6/NDUFC2-KCTD14/IDH3B/COQ9/NDUFA7/NDUFA4/C15orf48/MYBBP1A/TAZ/SRD5A1/NDUFA4L2/GLRX/SDHC/COX7A2L/SURF1/NDUFS8/NDUFAB1/NDUFA6/PINK1/UQCR10/SDHAF2/CYBA/NDUFB6/SDHD/NDUFV3/UQCRQ/GHITM/SLC25A22/ALDH4A1/CYB5A/COX7A2/UQCR11/COX7A1/SDHB/CYBB/NDUFB4/GFUS/NDUFB9/NDUFA12/NDUFS2/CYC1/NDUFA2/NDUFB3/DGUOK/NDUFB7/NDUFV1/NCF1/AIFM2/COX4I2/COX5B/AKR1A1/NDUFAF1/GSR/COX5A/NCF2/COX7B/NDUFB10/COX6B1/GPX2/DHRS3/NQO2/PHGDH |
| GOBP_PROTON_TRANSMEMBRANE_TRANSPORT | -0.513116542 | -2.030763941 | 1.33E-07 | 3.01E-05 | NDUFS7/DMAC2L/ATP6V0C/SLC15A4/ATP6V0E2/SLC33A1/COX10/COX8A/COX4I1/SLC2A9/STOML2/ATP6V1C2/ATP6V1A/ATP5F1EP2/NDUFA4/ATP5F1E/C15orf48/NDUFA4L2/COX7A2L/ATP6V1B2/SURF1/COX17/TMCO3/SLC17A5/DNAJC30/ATP2A3/MFSD3/PHB2/SPHK2/SLC46A1/CYB5A/ATP5F1D/COX7A2/COX7A1/SLC4A11/TCIRG1/ATP12A/ATP5PF/ATP6V0D2/HVCN1/TACO1/ATP5ME/SLC15A2/ATP5PO/ATP5PD/ATP5MC1/ATP6V0B/SLC9A3R1/SLC9A8/COX4I2/ATP5MF/ATP6V0D1/ATP6AP1/SLC2A10/COX5A/ATP6V1F/COX7B/COX6B1/UCP2/SLC36A1 |
| GOCC_AZUROPHIL_GRANULE_LUMEN | -0.575273051 | -2.114125229 | 2.04E-07 | 4.48E-05 | TOLLIP/ELANE/GUSB/FTL/CYB5R3/ARSA/PYGB/FUCA1/GLB1/CAP1/NAPRT/CREG1/RETN/GRN/ADA2/ACLY/GALNS/HEBP2/ARHGAP45/GLA/UNC13D/PRKCD/MAN2B1/FUCA2/TUBB4B/CPPED1/IMPDH1/CTSA/RNASE3/PYCARD/ARG1/GM2A/TRAPPC1/PADI2/VAT1/FABP5/SERPINB3/MNDA/GCA/PLAC8/DEFA1B/SERPINA3/RNASE2 |
| GOBP_RESPONSE_TO_FUNGUS | -0.687297069 | -2.290632922 | 2.15E-07 | 4.57E-05 | COTL1/BAK1/SYK/GNLY/PTX3/GAPDH/MYD88/TLR4/ARG1/CLEC7A/SCIMP/LTF/DEFA1B/S100A8/S100A9/S100A12 |
| GOBP_RESPONSE_TO_TRANSFORMING_GROWTH_FACTOR_BETA | 0.468738423 | 1.790716467 | 3.17E-07 | 6.55E-05 | POSTN/ASPN/PDE3A/LTBP1/MXRA5/PDGFD/ANKRD1/EDN1/FGFR2/ITGB6/NREP/TGFB3/ITGB8/MIR21/THBS1/COL3A1/LTBP2/FBN1/FOS/SOX5/ZFYVE9/PTPRK/FUT8/SNX25/BMPR1A/PDE2A/COL1A1/HSPA1A/LOX/TWSG1/PMEPA1/SFRP1/SMAD9/SMURF2/COL1A2/ZNF451/SMAD3/SKIL/SPRY1/MEF2C/MAP3K7/MYOCD/LTBP3/HTRA1/APAF1/PPARGC1A/MIR27A/NR3C1/LTBP4/UBB/SIRT1/ROCK1/SMURF1/LIMS1/JUN/FERMT2/LEMD3/TGFB2/ING2/TRIM33/USP9X/PBLD/SMAD2/HSP90AB1/LDLRAD4/MPP5/APPL1/ZFP36L2/ROCK2/COL4A2/ACVR1/SOX6/FYN/PARD3/SMAD5/APPL2/GLG1/PTK2/BAMBI/ADAM17/SMAD4/CFLAR/CLDN1/MIR29B1/WNT5A/SNW1/LATS1/RNF111/PPM1A/MIRLET7F1/MIR26A1/IL17RD/CREB1/CLEC3B/SMAD1 |
| GOBP_SKELETAL_SYSTEM_DEVELOPMENT | 0.402514772 | 1.634244278 | 3.94E-07 | 7.89E-05 | SFRP2/HAS2/IGF1/KIT/SULF1/BMP6/SFRP4/EDN1/TLL1/FGFR2/FREM1/LUM/BMP5/ITGB6/ARID5B/PRRX1/LRRC17/TGFB3/ITGB8/MIR21/BMP1/HIF1A/BMP3/PHEX/COL3A1/CSGALNACT1/TIPARP/FBN1/RAB23/FRZB/CCN2/ZFAND5/CCN1/MTHFD1L/SOX5/WDR48/CTNNB1/MMP16/MGP/GJA1/FBXW7/CLDN18/BMPR1A/NIPBL/COL1A1/COL5A2/FST/FAT4/SLC39A14/MBTD1/LOX/RANBP3L/SFRP1/AKAP13/PLEKHA1/ACVR2A/NFIB/COL1A2/BBX/NAB1/ATP7A/LRP6/ADAMTS12/SMAD3/SNAI2/RPS6KA3/EIF2AK3/HHIP/MEF2C/BBS2/RBP4/CHD7/LTBP3/MEX3C/CDH11/SETD2/IFT80/TRIP11/PCSK5/CD44/FGF2/FOXP1/RYK/RAB33B/NSD2/ARSL/LOXL2/DLG1/TGFB2/ASH1L/PBX1/ADAMTS4/IGF2/FGFR1/EFEMP1/SLC9B2/WDR19/MBTPS2/SMAD2/SBDS |
| GOBP_RESPONSE_TO_MECHANICAL_STIMULUS | 0.494230551 | 1.843669285 | 5.36E-07 | 0.000104403 | POSTN/ANGPT2/ITGA2/PIEZO2/KIT/ANKRD1/BMP6/EDN1/CXCL12/SERPINE2/FOSB/ADGRV1/MMP7/THBS1/COL3A1/FOS/RYR2/CXCR4/CTNNB1/GJA1/PDE2A/CXCL10/COL1A1/TTN/DCN/MAPK8/STRBP/PKD2/BTG2/CDH2/NFKB1/PPARG/DMD/RPS6KB1/SLITRK6/PIK3CA/BDKRB1/TNFRSF10B/MAP3K2/GADD45A/JUN/MAP1B/SLC38A2/USP53/XPA/CASP1/SCEL/FAS/HTR2A/MAP3K1/NFKBIA |
| GOMF_HYDROLASE_ACTIVITY_ACTING_ON_GLYCOSYL_BONDS | -0.515395566 | -2.005402836 | 5.80E-07 | 0.000107886 | HEXD/CTBS/GUSB/MYORG/MOGS/HEXA/GBA2/MAN1C1/ADPRS/FUCA1/GLB1/IL18R1/KL/SMUG1/APEX1/GANAB/GLB1L/NAGLU/MAN2A2/GLA/NAGPA/MAN2B1/DNPH1/FUCA2/AMY2A/BST1/PCNA/TLR2/NEU1/CHIA/TLR4/GLB1L2/GM2A/CEMIP/HYAL2/SMPDL3B/NAGA/SMPD1/TLR6/GBA/TLR1/IL18RAP/HYAL1/IL1RL1/MGAM/CHIT1 |
| GOBP_TRANSLATIONAL_TERMINATION | -0.548086899 | -2.064900276 | 5.86E-07 | 0.000107886 | MRPL38/MRPL10/MRPL27/UPF1/MRPL22/GADD45GIP1/MRPS12/MRPL13/MRPL48/MRPS17/MRPL46/CHCHD1/MRPL20/MRPL45/MRPS28/MRPL30/MTRF1L/MRPL17/MRPS21/MRPL12/OXA1L/MRPL14/ERAL1/MRPS23/MRPL16/JMJD4/MRPL40/MRPL24/MRPL9/MRPS35/MRPL23/MRPL35/MRPL49/MRPL21/MRPS36/MRPS2/MRPS25/GLE1/MRPS33/MRPL34/MRPL4/MRPL18/MRPL41/MRPL58/MRPS18C/MRPL57/MRPL11/AURKAIP1/MRPS11/MRPL33/MRPL53/APEH/MRPL28/MRPS15/MRPS16/MRPL37/MRPS18B/MRPS34/EIF5AL1 |
| GOCC_FICOLIN_1_RICH_GRANULE_MEMBRANE | -0.634464617 | -2.161417292 | 8.13E-07 | 0.000145809 | RAC1/NCKAP1L/SERPINB6/NBEAL2/TRPM2/TCIRG1/FPR2/DOK3/LAMP1/LAMTOR1/ARL8A/CD300A/SIRPA/SIGLEC14/SIGLEC5/SLC11A1/ITGB2/FCER1G/LILRB2/ITGAX/LILRA3/FPR1/CR1/MGAM |
| GOMF_OXIDOREDUCTASE_ACTIVITY_ACTING_ON_NAD_P_H_QUINONE_OR_SIMILAR_COMPOUND_AS_ACCEPTOR | -0.663213069 | -2.218296204 | 8.80E-07 | 0.000153707 | AKR1C1/NDUFB1/NDUFA13/NDUFA1/NDUFV2/NDUFS7/NDUFC2/NDUFC2-KCTD14/NDUFA7/NDUFA4/NDUFS8/NDUFAB1/NDUFA6/NDUFB6/NDUFV3/NDUFB4/NDUFB9/NDUFA12/NDUFS2/NDUFA2/NDUFB3/TP53I3/NDUFB7/NDUFV1/AIFM2/NDUFB10/CBR1/DCXR/AKR1C3/NQO2/AKR1C2/NQO1 |
| GOBP_DETECTION_OF_MECHANICAL_STIMULUS | 0.701840045 | 2.151619193 | 9.08E-07 | 0.000154667 | ITGA2/PIEZO2/KIT/CXCL12/SERPINE2/ADGRV1/CXCR4/CTNNB1/TTN/PKD2/CDH2 |
| GOCC_FICOLIN_1_RICH_GRANULE | -0.452743455 | -1.828068489 | 9.90E-07 | 0.000164627 | ADAM8/BIN2/SERPINA1/OSTF1/COMMD3/GLB1/MIF/RAC1/NCKAP1L/MVP/PSMC3/SERPINB6/DERA/PPIA/NBEAL2/PSMD11/PSMB1/ACLY/COMMD9/TRPM2/ALDOA/PSMD3/PNP/COTL1/TCIRG1/GYG1/PGAM1/CDA/LRG1/CSTB/FPR2/GSTP1/CTSD/PSMB7/DOK3/LAMP1/LAMTOR1/APEH/DBNL/ARL8A/IMPDH1/GMFG/CTSB/CD300A/AMPD3/SIRPA/IDH1/PKM/ALDOC/FCN1/MMP9/SIGLEC14/CAPN1/SIGLEC5/SLC11A1/QPCT/NME2/PYGL/PLEKHO2/MNDA/ITGB2/HK3/FCER1G/LILRB2/ITGAX/LILRA3/FPR1/CR1/MGAM |
| GOBP_TRANSMEMBRANE_RECEPTOR_PROTEIN_SERINE_THREONINE_KINASE_SIGNALING_PATHWAY | 0.419491784 | 1.663496442 | 1.16E-06 | 0.000181784 | SFRP2/ASPN/LTBP1/INHBA/SULF1/BMP6/EGR1/SFRP4/HIVEP1/BMP5/ITGB6/PDCD4/NREP/TGFB3/ITGB8/MIR21/BMP3/THBS1/COL3A1/LTBP2/FBN1/GPC3/FOS/CCN1/ZFYVE9/PTPRK/FUT8/SNX25/BMPR1A/FST/HSPA1A/LOX/TWSG1/PMEPA1/SFRP1/VIM/SMAD9/SMURF2/ACVR2A/COL1A2/ELAPOR2/DDX5/ZNF451/SMAD3/FSTL1/SKIL/SPRY1/NEO1/LEF1/MAP3K7/MYOCD/CTDSPL2/LTBP3/SFRP5/HTRA1/ZFYVE16/PPARG/LTBP4/UBB/SIRT1/SMURF1/LRP2/JUN/FERMT2/LEMD3/TGFB2/CRIM1/ING2/TRIM33/USP9X/PBLD/SMAD2/FGF10/HSP90AB1/LDLRAD4/MPP5/APPL1/ACVR1/NBL1/RUNX2/DKK1/PARD3/GREM1/SMAD5/BMPER/SPART/APPL2/GLG1/PTK2/RBPJ/BAMBI/ADAM17/SMAD4/ABL1/CCN3/MIR29B1/WNT5A/SNW1/LATS1/RNF111/PPM1A/MIRLET7F1/MIR26A1/IL17RD/CREB1/TGIF2 |
| GOBP_MUSCLE_CELL_MIGRATION | 0.577782687 | 1.993010454 | 1.17E-06 | 0.000181784 | POSTN/HAS2/ITGA2/IGF1/PDGFD/ITGB3/CCL5/NR4A3/MIR21/NET1/BMPR1A/SERPINE1/PRKG1/SEMA6D/IGFBP3/ATP7A/DOCK7/MEF2C/MYOCD/DOCK4/PDGFA/RPS6KB1/BCL2/PPARGC1A/NFE2L2/ROCK1/LPAR1/DOCK5/ANXA1/TRIB1 |
| GOMF_CYCLIC_NUCLEOTIDE_PHOSPHODIESTERASE_ACTIVITY | 0.807278821 | 2.147340498 | 1.17E-06 | 0.000181784 | PDE3A/PDE1A/PDE7B/PDE4D/PDE8B/PDE8A/PDE5A/PDE10A/PDE2A/PDE3B/PDE4B |
| GOBP_RESPIRATORY_ELECTRON_TRANSPORT_CHAIN | -0.530962333 | -2.010424274 | 1.51E-06 | 0.000228943 | COX7C/BID/UQCC3/NDUFB1/NDUFA13/ETFRF1/NDUFA1/NDUFV2/UQCRHL/ETFB/NDUFS7/UQCRFS1/COX10/UQCRC1/NDUFB11/NDUFC2/COX8A/COX4I1/COA6/NDUFC2-KCTD14/COQ9/NDUFA7/NDUFA4/MYBBP1A/TAZ/SDHC/COX7A2L/NDUFS8/NDUFAB1/NDUFA6/PINK1/UQCR10/SDHAF2/NDUFB6/SDHD/NDUFV3/UQCRQ/GHITM/SLC25A22/UQCR11/SDHB/NDUFB4/NDUFB9/NDUFA12/NDUFS2/CYC1/NDUFA2/NDUFB3/DGUOK/NDUFB7/NDUFV1/AIFM2/COX4I2/COX5B/NDUFAF1/COX5A/COX7B/NDUFB10/COX6B1 |
| GOBP_POSITIVE_REGULATION_OF_GTPASE_ACTIVITY | 0.413538507 | 1.646694017 | 1.68E-06 | 0.000248375 | RGS1/RGS5/THY1/CCL21/RASGRP1/PLCB1/CCL5/PREX2/ERRFI1/CHN1/DOCK10/F2R/ARHGAP21/RASAL2/ARHGAP42/RAPGEF6/IQGAP2/NET1/LRRK2/NF1/TBC1D4/SRGAP1/CCL18/ARHGAP15/RGS6/RASGRP3/RABGAP1L/DENND1B/MAP4K4/SIPA1L1/DLC1/MYO9A/RAPGEF2/RGS4/RGS2/STXBP5/ARHGEF7/RANBP3L/SFRP1/SOS1/RASA2/DOCK1/RGPD1/RGS16/ARHGAP24/RANBP2/TBC1D15/RASA1/DNM1L/USP6NL/RALGAPA1/RIC1/DOCK7/SNX9/ARHGEF12/TBC1D30/RAB3GAP2/RAB3GAP1/DOCK4/ARFGEF1/ARHGAP5/ASAP2/RALGAPA2/CXCL13/ARHGEF5/ARHGAP18/RAP1GAP/TBCK/DOCK11/EZH2/ZC3H15/TBC1D8B/LIMS1/ARFGAP3/ITGA6/JUN/DOCK5/ALS2/RACGAP1/SIPA1L2/CHML/HACD3/SGSM2/LARS1/RASGRF1/FERMT2/TBC1D22B/ICAM1/PKP4/DOCK9/ITGB1/ARHGEF6/TBC1D19/SNX13/CCL19/FAM13B/RABEP1/NRP1/RABGAP1/RGL3/ARHGAP20/TAGAP/CHM/EVI5/PICALM |
| GOMF_STRUCTURAL_CONSTITUENT_OF_RIBOSOME | -0.479221748 | -1.911002066 | 1.91E-06 | 0.000277467 | RPL19/RPL36AL/RPL7A/MRPL2/RPS9/RPS18/RPL26L1/RPL39/DAP3/RPL10/MRPL36/RPS15/MRPL10/MRPL27/RPS3/RPLP2/MRPL22/MRPS12/RPL37A/RPS27/MRPL13/RPL29/MRPS17/MRPL46/RPL12/RPL10A/MRPL20/MRPL30/MRPL17/RPL28/RPS19/MRPS21/MRPL12/RPS7/NDUFA7/RPL36/MRPL14/RPLP0/MRPS23/RPL18A/MRPL16/RPLP1/MRPL24/MRPL9/RPS21/MRPS35/MRPL23/RPS10/MRPL35/MRPL49/MRPL21/RPL27/MRPS36/MRPS2/MRPS25/MRPS33/RPL13/RPS17/MRPL34/RPL35/MRPL4/MRPL18/MRPL41/RPSA/RPS4Y1/RPL23/MRPS18C/MRPL57/MRPL11/MRPS11/MRPL33/MRPL28/MRPS15/MRPS16/MRPL37/MRPS18B/MRPS34/RPS5/RPL22L1/RPS26 |
| GOBP_AEROBIC_RESPIRATION | -0.56976582 | -2.050313728 | 2.02E-06 | 0.000286267 | UQCRHL/ME2/NDUFS7/PDHB/COX10/UQCRC1/COX8A/MDH1/COX4I1/MDH1B/IDH3B/OXA1L/NDUFA4/ACO2/BNIP3/SDHC/COX7A2L/SURF1/NDUFS8/UQCR10/SDHAF2/MTFR1L/SDHD/ATP5F1D/SDHB/IDH3G/CBFA2T3/FAHD1/DLAT/COX4I2/COX5B/OGDH/IDH2/DHTKD1/MDH2/COX5A/BLOC1S1/IDH1/COX7B/COX6B1/SHMT2 |
| GOBP_DETECTION_OF_MECHANICAL_STIMULUS_INVOLVED_IN_SENSORY_PERCEPTION | 0.758378077 | 2.127003359 | 2.07E-06 | 0.000288584 | ITGA2/PIEZO2/KIT/CXCL12/SERPINE2/ADGRV1/CXCR4 |
| GOMF_HELICASE_ACTIVITY | 0.506347119 | 1.848616209 | 2.16E-06 | 0.000293973 | SMARCA1/SHPRH/ATRX/WRN/BTAF1/SMC6/HLTF/CHD6/DDX58/DDX60/CHD1/CHD2/ASCC3/DDX5/HELB/DHX36/IFIH1/SMARCA5/CHD7/DDX10/DHX15/ERCC6L2/CHD1L/YTHDC2/SUPV3L1/SETX/MCM6/EIF4A2/DDX46/DDX60L/DHX9/DDX17/HELZ/ANXA1/DDX47/SMARCA2/MTREX/DDX3X/MCM9/DDX24/NAV2/DHX32/DICER1/DDX6/RAD50/CHD9/TTF2/DDX27/RAD54B/MRE11/DDX21/DHX35/DDX42/DDX50/DNA2/SLFN11/SNRNP200/DDX18/RECQL/DHX40/HELLS/SMARCAD1/DSCC1/CHD3/BRIP1/DHX29/EIF4A3/HFM1/DDX1/CHD8/HELQ/CHD4/INO80 |
| GOMF_RAGE_RECEPTOR_BINDING | -0.933151931 | -2.079215435 | 2.37E-06 | 0.000310646 | S100A4/FPR1/S100A8/S100A9/S100A12 |
| GOBP_REGULATION_OF_LEUKOCYTE_DEGRANULATION | -0.691121534 | -2.246795218 | 2.37E-06 | 0.000310646 | VAMP8/FES/LGALS9/SPHK2/PTAFR/SYK/UNC13D/LYN/PRAM1/LAMP1/RAC2/IL4R/CD300A/CEACAM1/STXBP2/CCR2/ITGB2/CD177/ADGRE2/FOXF1/GATA2/ITGAM/FGR/HMOX1 |
| GOBP_PHAGOCYTOSIS | -0.397539571 | -1.687899825 | 2.44E-06 | 0.000313911 | ELANE/LMAN2/MST1R/XKR6/PIK3R2/RAPGEF1/BAIAP2/CDC42SE1/TMEM175/FCGR1A/TUSC2/IL2RB/WIPF2/ARPC1B/BIN2/TM9SF4/XKR8/TNF/RAC1/MARCO/NCKAP1L/FCN2/ANXA11/NR1H3/RAB39A/CSK/ARHGAP25/NOD2/FCGR2A/RHOBTB2/RAB20/SH3BP1/CALR/CYBA/RARA/SNX3/CYFIP1/CEACAM4/ANXA3/VAV1/C1orf43/ADORA2A/PIP4P2/IRF8/WAS/ARPC3/C4BPB/FPR2/WASF2/AIF1/SYK/UNC13D/PTPRJ/PRKCD/MYO1C/MERTK/LYN/SRPX/RAC2/CCL2/LIMK1/TLR2/ITGAL/PTX3/ICAM3/PRKCE/TYRO3/ARPC4/CD300A/RAB7A/PYCARD/MYD88/TLR4/MYO1G/GRB2/SIRPB1/SIRPA/SCARB1/CD300LF/FCN1/NCF2/DNM2/IL15RA/CLN3/SLC11A1/RHOG/DYSF/PECAM1/TREM2/CLEC7A/ITGB2/CORO1A/FCER1G/GATA2/NCF4/MFGE8/HCK/ITGAM/CD14/FGR/FCN3 |
| GOCC_TERTIARY_GRANULE_MEMBRANE | -0.585945355 | -2.057476028 | 3.36E-06 | 0.000424054 | LAMTOR2/VAMP8/SERPINB6/NBEAL2/CYBA/TRPM2/CYSTM1/CYBB/PTAFR/FPR2/CD33/PLAU/CD53/CD300A/SIRPA/CLEC5A/SIGLEC14/SIGLEC5/SLC11A1/CEACAM1/TSPAN14/KCNAB2/LAIR1/ITGB2/CD177/FCER1G/LILRB2/ITGAX/LILRA3/ITGAM/MGAM |
| GOBP_REGULATION_OF_GTPASE_ACTIVITY | 0.397449554 | 1.607675026 | 3.59E-06 | 0.000444264 | RGS1/RGS5/THY1/CCL21/RASGRP1/EPHA3/PLCB1/EPHA4/CCL5/PREX2/ERRFI1/CHN1/MIR21/DOCK10/F2R/ARHGAP21/RASAL2/ARHGAP42/RAPGEF6/IQGAP2/NET1/LRRK2/NF1/TBC1D4/SRGAP1/CCL18/ARHGAP15/RHOH/WNK1/FGD4/RGS6/LRCH1/RASGRP3/RABGAP1L/DENND1B/PRKG1/MAP4K4/SIPA1L1/KLRC4-KLRK1/DLC1/MYO9A/RAPGEF2/RGS4/RGS2/STXBP5/ARHGEF7/RANBP3L/SFRP1/SOS1/RASA2/DOCK1/RGPD1/CBLB/RGS16/ARHGAP24/RANBP2/TBC1D15/MET/RASA1/DNM1L/USP6NL/RALGAPA1/RIC1/DOCK7/FGD6/SPRY1/SNX9/ARHGEF12/TBC1D30/RAB3GAP2/RAB3GAP1/DOCK4/ARFGEF1/ARHGAP5/ASAP2/RALGAPA2/RICTOR/CXCL13/ARHGEF5/ARHGAP18/RAP1GAP/TBCK/DOCK11/EZH2/ZC3H15/TBC1D8B/LIMS1/ARFGAP3/ITGA6/JUN/DOCK5/ALS2/RACGAP1/SIPA1L2/CHML/VAV2/HACD3/SGSM2/LARS1/RASGRF1/FERMT2/TBC1D22B/ICAM1/PKP4/DOCK9/ITGB1/ARHGEF6/TBC1D19/SNX13/CCL19/FAM13B/RABEP1/NRP1/RABGAP1/RGL3/ARHGAP20/PLXNA4/TAGAP/CHM/EVI5/PICALM/TRAPPC6B/EIF5/ARHGAP12/ARAP2 |
| GOMF_IMMUNE_RECEPTOR_ACTIVITY | -0.492688265 | -1.928715214 | 4.01E-06 | 0.000488489 | C5AR2/FPR2/CRLF1/FLT3/IL10RB/CX3CR1/IL4R/CSF2RB/IL10RA/ACKR4/FPR3/IL15RA/C5AR1/CMKLR1/PIGR/IL18RAP/CCRL2/CCR2/IL17RA/LILRB1/IL1RL1/FCER1G/LILRB2/C3AR1/FPR1/CCR1/CXCR2/CR1/CXCR1/CSF3R/IL1R2 |
| GOCC_NUCLEAR_SPECK | 0.405911028 | 1.626281677 | 4.38E-06 | 0.000523264 | PLCB1/LUC7L3/NR4A2/PNISR/HIF1A/SMC4/SCAPER/MEOX2/WRN/PYHIN1/HNRNPU/SMC6/SMC5/NRIP1/HSPA1B/SNRPA1/SF3B1/DENND1B/HSPA1A/PNN/ZNF638/CCNL1/RBM25/HBP1/SRSF5/RBM39/DOCK1/GPATCH2/ASCC3/SMURF2/PPIG/NFKBIZ/THOC1/MECOM/SRSF11/KMT2E/PSME4/RSRC1/IFI16/DHX36/API5/JADE1/TCF12/TRIM22/MEF2C/NOC3L/FNDC1/BNIP3L/TCIM/FNBP4/CMYA5/DHX15/PPP1R16B/PRKAA2/RBBP6/SFPQ/BCLAF1/CDC5L/DYRK3/SREK1/ARHGAP18/NR3C1/YTHDC1/CSNK1A1/USP36/WBP4/TOPORS/PRPF4B/TENM1/TRIP11/TARDBP/GADD45A/DDX46/PRPF18/DDX17/WTAP/SYF2/CPSF6/SRSF6/MTREX/ADAMTS4/S100PBP/DUSP11/BMP2K/WAC/LMNA/SRSF1/MAML2/PRPF40A/METTL3/SRSF10/MORF4L1/HP1BP3/PPP4R3A/SFMBT2/HAUS6/TCERG1/SON/SRSF7/ERBIN/ZC3H13/NSRP1/PIAS2/YLPM1/SF3A3/CWC22/EPOR/SART3/FYTTD1/CRY2/ZNF217/RADX/THOC2/DYRK1A/NXF1/CDK13/CIR1/DDX42/DUSP10/COPS4/CWC15/TAF5L/PPP4R3B/ZNF830/RBM15/TRIP12/SNW1/SMNDC1/FTO/RBM27/CRNKL1/WT1/GLI3/SDE2/SRP54/POLI/NAMPT/FAM76B/SLU7/SMU1/ZC3H14/GLI2/PRKN/AFDN/CDK12/ITPKC/CARMIL1/HABP4/SRPK2/EIF4A3/NCBP3/BCAS2 |
| GOBP_TOLL_LIKE_RECEPTOR_SIGNALING_PATHWAY | -0.469146013 | -1.862289827 | 4.58E-06 | 0.000537781 | CTSL/MAPKAPK3/TLR5/LY96/LYN/TICAM1/NMI/CNPY3/TLR2/UNC93B1/PRKCE/TYRO3/CTSB/UBE2D1/CD300A/MYD88/TLR4/FLOT1/CD300LF/S100A14/PTPRS/SMPDL3B/TLR6/PIK3AP1/TLR1/SCIMP/ITGB2/LTF/LILRA2/ARRB2/TLR8/ITGAM/CD14/S100A8/S100A9/BPIFB1 |
| GOCC_MITOCHONDRIAL_LARGE_RIBOSOMAL_SUBUNIT | -0.623695594 | -2.098316575 | 5.26E-06 | 0.000607706 | MRPL36/MRPL38/MRPL10/MRPL27/MRPL22/MALSU1/MRPL13/MRPL48/MRPL46/MRPL20/MRPL45/MRPL30/MRPL17/MRPL12/MRPL14/MRPL16/MRPL40/MRPL24/MRPL9/MRPL23/MRPL35/MRPL49/NDUFAB1/MRPL21/MRPL34/MRPL4/MRPL18/MRPL41/MRPL58/MRPL57/MRPL11/MPV17L2/MRPL33/MRPL53/MRPL28/MRPL37 |
| GOMF_NUCLEOSIDE_TRIPHOSPHATASE_REGULATOR_ACTIVITY | 0.387944457 | 1.579558504 | 5.55E-06 | 0.000630594 | RGS1/RGS5/THY1/RASGRP1/PLCB1/PREX2/ERRFI1/CHN1/RASGRF2/HSPH1/DOCK10/ARHGAP21/RASAL2/ARHGAP42/RAPGEF6/IQGAP2/LRRK2/ITSN1/NF1/TBC1D4/SRGAP1/DNAJB1/ARHGAP15/DNAJA1/RHOH/FGD4/RGS6/DNAJB4/RASGRP3/RABGAP1L/DENND1B/SIPA1L1/DLC1/PLCE1/MYO9A/RAPGEF2/RGS4/DENND11/TRIO/MYCBP2/RGS2/STXBP5/ARHGEF7/RANBP3L/SOS1/RASGEF1B/RASA2/RALGPS2/DOCK1/AKAP13/RGPD1/RGS16/HERC1/ARHGAP24/DNMBP/RANBP2/DENND2C/RAB3IP/TBC1D15/RASA1/DNM1L/USP6NL/HERC2/RALGAPA1/RIC1/DOCK7/FGD6/DENND4A/ARHGEF12/TBC1D30/RAB3GAP2/RAB3GAP1/DOCK4/ARFGEF1/FNIP2/ARHGAP5/DENND4C/ASAP2/RALGAPA2/ITSN2/RABGEF1/ARHGEF5/ARHGAP18/RAP1GAP/TBCK/DOCK11/AHSA2P/ARFGEF3/ATP1B3/DENND5A/SOS2/TBC1D8B/ARFGAP3/JUN/DOCK5/ALS2/RACGAP1/SIPA1L2/CHML/VAV2/HACD3/SGSM2/LARS1/ARHGEF28/RASGRF1/FNIP1/DENND5B/TBC1D22B/DOCK9/ARHGEF6/TBC1D19/FAM13B/RABEP1/NRP1/RABGAP1/RGL3/ARHGAP20/ELMO1/TAGAP/CHM/DENND2A/EVI5 |
| GOBP_RESPONSE_TO_ACTIVITY | 0.612994738 | 1.992817931 | 6.31E-06 | 0.000704572 | POSTN/ANGPT2/ITGA2/BMP6/EDN1/HIF1A/RYR2/CXCR4/RARRES2/CRY1/PRKAA2/PPARGC1A/CAPN3/CAT/OPA1/SOD2/COL4A2/ADIPOQ |
| GOBP_ENERGY_DERIVATION_BY_OXIDATION_OF_ORGANIC_COMPOUNDS | -0.392653355 | -1.660607666 | 8.10E-06 | 0.000890828 | GAA/PPP1R3F/COX7C/MTFR1/BID/KHK/UQCC3/NDUFB1/NDUFA13/TIGAR/ETFRF1/PNPT1/NDUFA1/SDHAF4/NDUFV2/UQCRHL/ME2/ETFB/NDUFS7/PHLDA2/PDHB/UQCRFS1/PGM1/COX10/UQCRC1/NDUFB11/NDUFC2/PYGB/SORBS1/COX8A/MDH1/COX4I1/MDH1B/COA6/CISD1/PHKG2/NDUFC2-KCTD14/IDH3B/PPP1R3E/COQ9/OXA1L/NDUFA7/NDUFA4/TP53/IRS2/ACO2/POMC/MYBBP1A/BNIP3/KL/TAZ/MTOR/SDHC/COQ10A/COX7A2L/SURF1/NDUFS8/NDUFAB1/NDUFA6/PPP1R3D/PINK1/PCDH12/UQCR10/SDHAF2/MTFR1L/NDUFB6/SDHD/NDUFV3/UQCRQ/GHITM/SLC25A22/GYS1/ATP5F1D/UQCR11/SDHB/NDUFB4/GYG1/IDH3G/GSK3A/NDUFB9/CBFA2T3/NDUFA12/NDUFS2/CYC1/FAHD1/NDUFA2/NDUFB3/DLAT/DGUOK/NDUFB7/NDUFV1/AIFM2/COX4I2/INPP5K/COX5B/OGDH/STK40/IDH2/DHTKD1/PRELID1/PPP1R3C/PPP1CA/NDUFAF1/MDH2/COX5A/BLOC1S1/IDH1/COX7B/NDUFB10/COX6B1/PYGL/SHMT2 |
| GOCC_PHAGOCYTIC_VESICLE | -0.473826101 | -1.865950361 | 8.53E-06 | 0.00091543 | TRIM14/VAMP8/RAB32/MTOR/ANXA11/RAB11B/RAB39A/NOD2/HLA-E/RAB20/PIP4P1/CALR/CYBA/SNX3/ABCA1/ANXA3/CYBB/RAB8A/PIP4P2/TCIRG1/WAS/RILP/ATP6V0D2/HVCN1/ZDHHC5/SYK/GNLY/MYO1C/LAMP1/RAC2/ATP6V0B/NCF1/TLR2/UNC93B1/ATP6V0D1/FMNL1/RAB7A/CDC42EP2/NCF2/MCOLN1/DNM2/SLC11A1/TLR6/TLR1/MPEG1/SCIMP/LTF/CORO1A/NCF4 |
| GOMF_EXTRACELLULAR_MATRIX_STRUCTURAL_CONSTITUENT_CONFERRING_TENSILE_STRENGTH | 0.701596665 | 2.08960634 | 8.79E-06 | 0.00091543 | COL14A1/COL6A6/COL15A1/COL6A3/COL3A1/COL1A1/COL5A2/COL12A1/COL4A3/COL1A2/COL4A1 |
| GOMF_GROWTH_FACTOR_ACTIVITY | 0.49724445 | 1.8233518 | 8.81E-06 | 0.00091543 | OGN/MACC1/INHBA/IGF1/PDGFD/AREG/BMP6/CXCL12/HGF/BMP5/TGFB3/BMP1/BMP3/FGF7/PPBP/CCN2/HBEGF/FGF14/HDGFL3/PDGFA/FGF1/EPGN/VEGFA/FGF2/IL7/TGFB2/IGF2/EFEMP1/RABEP1/FGF10 |
| GOBP_ORGAN_OR_TISSUE_SPECIFIC_IMMUNE_RESPONSE | -0.699759478 | -2.192713205 | 8.87E-06 | 0.00091543 | H2BC7/H2BC12/XCL1/H2BC21/RAB17/NOD2/H2BC4/FFAR2/RNASE3/PIGR/LTF/DEFA1B/RNASE2/BPIFB1 |
| GOCC_VACUOLAR_LUMEN | -0.441288107 | -1.775135767 | 9.57E-06 | 0.000973409 | BGN/TOLLIP/ELANE/GUSB/FTL/CYB5R3/ARSA/HEXA/PYGB/PDGFRB/GPC1/LIPA/FUCA1/GLB1/SGSH/CSPG5/CAP1/NAPRT/CSPG4/CREG1/RETN/GRN/ADA2/PLD3/ACLY/GALNS/NAGLU/HEBP2/GYG1/ARHGAP45/GLA/CTSL/UNC13D/CTSD/PRELP/EPDR1/PRKCD/IFI30/MAN2B1/FUCA2/TUBB4B/NAAA/CPPED1/IMPDH1/ATP13A2/CTSA/CTSB/NEU1/RNASE3/PYCARD/TPP1/ARG1/GM2A/TRAPPC1/PADI2/VAT1/PLBD2/SMPD1/GBA/ACP2/FABP5/SERPINB3/MNDA/GCA/HYAL1/TCN2/PLAC8/DEFA1B/SERPINA3/RNASE2 |
| GOBP_SMOOTH_MUSCLE_CELL_MIGRATION | 0.58320556 | 1.947853389 | 1.03E-05 | 0.001028882 | POSTN/HAS2/ITGA2/IGF1/PDGFD/ITGB3/CCL5/NR4A3/MIR21/BMPR1A/SERPINE1/PRKG1/SEMA6D/IGFBP3/ATP7A/DOCK7/MEF2C/MYOCD/DOCK4/PDGFA/RPS6KB1/BCL2/PPARGC1A/NFE2L2/LPAR1/DOCK5/TRIB1 |
| GOCC_LARGE_RIBOSOMAL_SUBUNIT | -0.486079921 | -1.867444603 | 1.08E-05 | 0.00106378 | RPL19/RPL36AL/RPL7A/MRPL2/RPL26L1/NSUN4/RPL39/RPL10/MRPL36/MRPL38/MRPL10/MRPL27/RPLP2/MRPL22/RPL37A/MALSU1/MRPL13/MRPL48/RPL29/MRPL46/RPL12/RPL10A/MRPL20/MRPL45/MRPL30/MRPL17/RPL28/MRPL12/RPL36/MRPL14/RPLP0/RPL18A/MRPL16/MRPL40/RPLP1/MRPL24/MRPL9/MRPL23/MRPL35/MRPL49/NDUFAB1/MRPL21/RPL27/RPL13/MRPL34/RPL35/MRPL4/MRPL18/MRPL41/RPL23/MRPL58/MRPL57/MRPL11/MPV17L2/MRPL33/MRPL53/MRPL28/MRPL37 |
| GOMF_PEPTIDE_RECEPTOR_ACTIVITY | -0.464519495 | -1.843413693 | 1.14E-05 | 0.001105278 | CX3CR1/INPP5K/LGR5/AGTR2/PTH1R/RXFP1/ACKR4/RAMP2/FPR3/F2RL3/RAMP3/CMKLR1/AGTRAP/CCRL2/CCR2/NPR3/EDNRB/AGTR1/FPR1/CCR1/CXCR2/VIPR1/CXCR1 |
| GOMF_CALMODULIN_BINDING | 0.461628606 | 1.719554087 | 1.23E-05 | 0.001176221 | RGS1/GEM/PDE1A/PLCB1/ATP2B1/MYH10/RASGRF2/EEA1/CAMK4/IQGAP2/RYR2/CASK/CAMSAP2/CALD1/MYH11/TTN/AKAP12/MYO1E/AKAP5/RGS4/RGS2/RGS16/CAMK2D/MYO1B/PHKA1/CNN1/ADD3/INVS/CEP97/IQCB1/FBXL2/KCNN3/ATP2B4/SPTAN1/PPP3CA/CNN3/MYO5A/SNTB2/TJP1/UNC13B/CAMK1D/MYO6/DAPK2/FAS/RYR3/STRN/STRN3/ASPM/SPTBN1/CAMSAP1/ADD2/MYO5C |
| GOMF_HEPARIN_BINDING | 0.485727361 | 1.783794908 | 1.26E-05 | 0.001188965 | POSTN/CCDC80/ECM2/CFH/SERPINE2/SELP/FGFR2/THBS2/F11/MMP7/PCOLCE2/RSPO3/THBS1/FGF7/LTBP2/FBN1/LPL/CCN2/CCN1/CXCL11/CXCL10/TNXB/LXN/ANOS1/SFRP1/HBEGF/SLIT3/FSTL1/ADAMTS3/ZNF146/FGF1/CXCL13/NDNF/TENM1/EFEMP2/VEGFA/FGF2/FN1/CTSG/FGFR1/NRP1/FGF10/PAFAH1B1/ADAMTS5 |
| GOBP_MITOCHONDRIAL_RESPIRATORY_CHAIN_COMPLEX_ASSEMBLY | -0.530444557 | -1.954125268 | 1.31E-05 | 0.001200729 | NDUFS3/NDUFAF3/TMEM126B/COA4/PET117/NDUFB8/ECSIT/UQCC3/NDUFB1/NDUFA13/AIFM1/NDUFA1/SDHAF4/NDUFV2/NDUFS7/CHCHD4/SDHAF3/UQCRFS1/NDUFB11/NDUFC2/OXA1L/NDUFA7/NDUFAF4/TAZ/COX18/SURF1/NDUFS8/COX17/NDUFAB1/NDUFA6/SDHAF2/NDUFB6/NDUFV3/FOXRED1/NDUFB4/NDUFB9/NDUFA12/NDUFS2/TACO1/NDUFA2/NDUFB3/BCS1L/NDUFB7/NDUFV1/NDUFAF1/NDUFB10/UQCC2/DMAC2 |
| GOMF_GLYCOSAMINOGLYCAN_BINDING | 0.447831654 | 1.687581027 | 1.33E-05 | 0.001200729 | POSTN/CCDC80/ECM2/SULF1/CFH/SERPINE2/SELP/FGFR2/THBS2/DPYSL3/F11/MMP7/PCOLCE2/TNFAIP6/RSPO3/THBS1/FGF7/IGHM/LTBP2/FBN1/LPL/CCN2/JCHAIN/CCN1/CXCL11/CXCL10/TNXB/DCN/LXN/NLRP3/ANOS1/SFRP1/HBEGF/SLIT3/FSTL1/ADAMTS3/ZNF146/SEMA5A/FGF1/CXCL13/NDNF/LTBP4/TENM1/EFEMP2/VEGFA/CD44/FGF2/EXTL2/FN1/CTSG/FGFR1/NOD1/NRP1/FGF10/PAFAH1B1 |
| GOBP_CYCLIC_NUCLEOTIDE_CATABOLIC_PROCESS | 0.867637384 | 2.048053747 | 1.34E-05 | 0.001200729 | PDE1A/PDE7B/PDE4D/PDE8B/PDE8A/PDE5A/PDE10A/PDE2A/PDE4B |
| GOMF_3_5_CYCLIC_AMP_PHOSPHODIESTERASE_ACTIVITY | 0.892559008 | 2.064297124 | 1.35E-05 | 0.001200729 | PDE3A/PDE7B/PDE4D/PDE8B/PDE8A/PDE2A/PDE3B/PDE4B |
| GOMF_NAD_P_H_DEHYDROGENASE_QUINONE_ACTIVITY | -0.670357942 | -2.151735209 | 1.36E-05 | 0.001200729 | NDUFB1/NDUFA13/NDUFA1/NDUFV2/NDUFS7/NDUFC2/NDUFC2-KCTD14/NDUFA7/NDUFA4/NDUFS8/NDUFAB1/NDUFA6/NDUFB6/NDUFV3/NDUFB4/NDUFB9/NDUFA12/NDUFS2/NDUFA2/NDUFB3/NDUFB7/NDUFV1/NDUFB10/NQO2/NQO1 |
| GOCC_NADH_DEHYDROGENASE_COMPLEX | -0.656026453 | -2.143282382 | 1.41E-05 | 0.001233139 | NDUFB1/NDUFA13/NDUFA1/NDUFV2/NDUFS7/NDUFB11/NDUFC2/NDUFC2-KCTD14/NDUFA7/NDUFA4/NDUFS8/NDUFAB1/NDUFA6/NDUFB6/NDUFV3/FOXRED1/NDUFB4/NDUFB9/NDUFA12/NDUFS2/NDUFA2/NDUFB3/NDUFB7/NDUFV1/NDUFAF1/NDUFB10/DMAC2 |
| GOBP_RESPONSE_TO_TUMOR_NECROSIS_FACTOR | 0.416215396 | 1.620593269 | 1.45E-05 | 0.00125362 | POSTN/VCAM1/HAS2/FABP4/SELE/CCL21/ANKRD1/EDN1/RORA/BIRC3/TNFAIP3/CCL5/GBP1/TNFSF8/GPD1/THBS1/CYLD/GBP3/ZFP36/CCL18/CLDN18/TRAF5/COL1A1/AKAP12/HSPA1B/HSPA1A/AIM2/NR1D1/PLVAP/SFRP1/STAT1/NUB1/BIRC2/PSME4/ADAMTS12/TNFRSF21/NFKB1/UBD/TNFSF13B/RPS6KB1/PPARGC1A/GBP2/YTHDC2/NFE2L2/TANK/SIRT1/CRHBP/TNFRSF9/LIMS1/CD40LG/CCDC3/DHX9/MAP3K5/WDR35/ICAM1/TRAF3/PTPN2/CCL19/SGMS1/CASP1/TAX1BP1/FAS/NFKBIA/ZFP36L2/MAP4K3/MIR24-1/OTULIN/ADIPOQ |
| GOBP_REGULATION_OF_REGULATED_SECRETORY_PATHWAY | -0.474628778 | -1.875299477 | 1.47E-05 | 0.001255843 | LGALS9/MICAL1/SPHK2/GIPC1/SNAPIN/ADORA2A/PTAFR/SYK/UNC13D/LYN/PRAM1/VPS18/LAMP1/RAC2/CDK5/CALM3/P2RY1/IL4R/GIT1/RAB15/CACNA1H/CD300A/NOTCH1/RAB3D/CEACAM1/STXBP2/CCR2/ITGB2/CD177/ADGRE2/FOXF1/GATA2/ITGAM/FGR/ADRA1A/HMOX1 |
| GOBP_PATTERN_RECOGNITION_RECEPTOR_SIGNALING_PATHWAY | -0.421750322 | -1.730612955 | 1.78E-05 | 0.001499018 | CTSL/MAPKAPK3/RELA/TLR5/ZDHHC5/SLC15A3/LY96/SLC15A2/LYN/TICAM1/NMI/CNPY3/TLR2/UNC93B1/FFAR2/PRKCE/TYRO3/CLPB/RNF135/CTSB/UBE2D1/CD300A/MYD88/TLR4/FLOT1/PHB/CD300LF/S100A14/FCN1/PTPRS/SMPDL3B/TLR6/PIK3AP1/TLR1/SCIMP/ITGB2/LTF/LILRA2/CLEC4E/ARRB2/TLR8/ITGAM/CD14/S100A8/S100A9/BPIFB1 |
| GOMF_ANION_TRANSMEMBRANE_TRANSPORTER_ACTIVITY | -0.330455148 | -1.45804099 | 1.91E-05 | 0.001585164 | SLC26A8/SLC19A1/BLOC1S3/CD320/SLC4A2/SLC26A11/SLC1A4/SLC25A4/ABCC10/SLC27A2/TIMM17A/ANO8/SLC2A6/SLC17A5/CLIC1/SLC16A3/MFSD4A/SLC38A6/SLC38A10/SLC51B/SLC46A1/SLC25A22/ABCA1/SLC25A19/SLCO2B1/SLC12A8/SLC26A6/GABRP/SLC4A11/SLC29A2/TOMM22/SLC44A2/SLC5A1/SLC5A6/CLDN4/SLC15A3/FLVCR2/SLC15A2/SLC2A1/SLC22A4/CLCN6/SLC37A4/TTYH3/MPC1/TOMM40/SLC9A3R1/SLC25A20/FXYD3/SLC9A8/SLC12A9/SLC43A1/SLC38A7/SLC39A8/SLC25A44/SLC16A12/GET3/SLC1A5/SFXN2/SLC7A11/SLC35B1/SLC44A4/ADAMTS8/SLC2A10/AZGP1/SLC25A1/MFSD5/SLC38A5/SLC48A1/SLC35C1/ABCB6/CLCN7/SLC35A4/SLC16A13/DISP1/SLC25A11/CEACAM1/SLC37A2/CTNS/SLC6A4/SLC50A1/SLC3A2/SLC66A1L/AQP1/SLC6A9/SLC16A6/SLCO2A1/C1orf194/SLCO1A2/SLC7A7/SLC36A1/AQP9/SLCO4A1 |
| GOBP_UROGENITAL_SYSTEM_DEVELOPMENT | 0.412479934 | 1.614010391 | 1.93E-05 | 0.001585164 | HAS2/ANGPT2/SULF1/PDGFD/BMP6/MME/BICC1/EGR1/ROBO2/FGFR2/CA2/EPHA4/ARID5B/FRAS1/AHI1/FREM2/TIPARP/FBN1/FRS2/GPC3/ENPEP/LRRK2/CTNNB1/NF1/NIPBL/FAT4/MYO1E/GCNT4/DCN/CEP290/PLCE1/SFRP1/STAT1/NPHP3/PKD2/COL4A3/BCL2L11/SMAD3/PYGO1/SPRY1/MEF2C/SMARCC1/MYOCD/RBP4/COL4A1/PDGFA/APAF1/FGF1/BCL2/DYNC2H1/CNTRL/ADAMTS6/ACTA2/LRP4/NFIA/ITGA8/ANGPT1/LRP2/PCSK5/VEGFA/YAP1/ZNF354A/FGF2/NUP107/TBC1D32/KCNJ8/ANXA1/CENPF/CAT/SDC4/DLG1/TGFB2/PBX1/KLHL3/SMAD2/FGF10/LGR4/RPGRIP1L/CRIP1/PPAT/WDPCP/UBE3A/ADIPOQ/HNF1B/NUP160/FEM1B |
| GOBP_REGULATION_OF_TRANSMEMBRANE_RECEPTOR_PROTEIN_SERINE_THREONINE_KINASE_SIGNALING_PATHWAY | 0.433431292 | 1.647972114 | 2.05E-05 | 0.001658452 | SFRP2/ASPN/LTBP1/INHBA/SULF1/BMP6/SFRP4/BMP5/NREP/TGFB3/MIR21/BMP3/THBS1/FBN1/GPC3/CCN1/SNX25/BMPR1A/FST/HSPA1A/LOX/TWSG1/PMEPA1/SFRP1/SMURF2/ACVR2A/ELAPOR2/ZNF451/SMAD3/FSTL1/SKIL/SPRY1/NEO1/MYOCD/CTDSPL2/SFRP5/HTRA1/PPARG/LTBP4/UBB/SIRT1/SMURF1/LRP2/LEMD3/TGFB2/CRIM1/ING2/TRIM33/PBLD/SMAD2/FGF10/HSP90AB1/LDLRAD4/MPP5/ACVR1/NBL1/DKK1/GREM1/BMPER/SPART/GLG1/RBPJ/BAMBI/ADAM17/SMAD4/ABL1/CCN3/MIR29B1/WNT5A/SNW1/LATS1/RNF111/PPM1A/MIRLET7F1/MIR26A1/IL17RD |
| GOMF_INTEGRIN_BINDING | 0.503068907 | 1.818017528 | 2.07E-05 | 0.001658452 | SFRP2/VCAM1/ESM1/THY1/ITGA2/ECM2/IGF1/ITGB3/CXCL12/FAP/ITGB6/ITGBL1/ITGB8/THBS1/COL3A1/FBN1/CCN2/UTRN/ITGA4/CCN1/DST/ITGAV/LAMA3/TNXB/COL4A3/CXADR/CD226/EDIL3/EGFL6/FGF1/LTBP4/CD9/CD40LG/LAMB1/FGF2/PLPP3/FN1/JAM3/FERMT2/ICAM1/ADAM23/PTPN2/IGF2/ITGB1 |
| GOBP_CARTILAGE_DEVELOPMENT | 0.464522236 | 1.728736459 | 2.12E-05 | 0.001663754 | SFRP2/SULF1/BMP6/EDN1/LUM/BMP5/PRRX1/ITGB8/MIR21/BMP1/HIF1A/BMP3/CSGALNACT1/FRZB/CCN2/CCN1/SOX5/MGP/BMPR1A/COL1A1/SLC39A14/NFIB/ATP7A/LRP6/ADAMTS12/SMAD3/SNAI2/EIF2AK3/MEF2C/BBS2/LTBP3/MEX3C/IFT80/TRIP11/CD44/FGF2/LOXL2/EFEMP1/EVC/TRPS1/SOX6/RUNX2/MBOAT2/GREM1/SMAD5/SCIN/LNPK/MDK/GLG1/CHSY1/TAPT1/CCN3/WNT5A/SERPINH1/RARB/GLI3/SMAD1/PTPN11/OSR1 |
| GOBP_DEFENSE_RESPONSE_TO_FUNGUS | -0.686454885 | -2.142501431 | 2.12E-05 | 0.001663754 | JAGN1/HAMP/COTL1/GNLY/GAPDH/ARG1/LTF/DEFA1B/S100A8/S100A9/S100A12 |
| GOBP_ATP_METABOLIC_PROCESS | -0.377958005 | -1.610656954 | 2.19E-05 | 0.001690049 | COX7C/ATP5F1A/BID/FOXK2/HDAC4/UQCC3/GPI/NDUFB1/NUDT2/NDUFA13/LDHA/TIGAR/TSPO/PGAM2/NDUFA1/EIF6/TEFM/NDUFV2/UQCRHL/NUP85/NDUFS7/JMJD8/DMAC2L/ATP6V0C/ENTPD5/UQCRFS1/NDC1/PGM1/COX10/UQCRC1/NDUFB11/NDUFC2/FIS1/COX8A/NUP37/COX4I1/ABCD1/COA6/NDUFC2-KCTD14/STOML2/COQ9/ATP6V1A/ATP5F1EP2/NDUFA7/NDUFA4/ATP5F1E/TP53/TPI1/TAZ/PRXL2C/TGFB1/SDHC/COX7A2L/ATP6V1B2/SURF1/NDUFS8/SLC2A6/NDUFAB1/NDUFA6/PINK1/POM121C/DNAJC30/PGAM4/UQCR10/SDHAF2/NDUFB6/SDHD/NDUFV3/PRKAG1/SPHK2/UQCRQ/GHITM/GALK1/ALDOA/ATP5F1D/NUDT5/COX7A2/UQCR11/FAM20C/COX7A1/NDUFB4/ENO2/PGAM1/CHCHD10/NDUFB9/ATP5PF/CBFA2T3/NDUFA12/NDUFS2/ATP5ME/CYC1/ATP5PO/NDUFA2/ATP5PD/NDUFB3/DGUOK/NDUFB7/ATP5MC1/ZBTB7A/NDUFV1/COX4I2/COX5B/ATP5MF/PFKFB4/OGDH/AAAS/ENO1/GIT1/GAPDH/DHTKD1/NDUFAF1/COX5A/PKM/ALDOC/COX7B/NADK/NDUFB10/COX6B1/UQCC2/TREM2/CTNS/NUPR1/HK3/SHMT2 |
| GOBP_STEROL_METABOLIC_PROCESS | -0.442137782 | -1.768928933 | 2.22E-05 | 0.001690049 | LBR/CYB5R3/GBA2/APOBR/APOL1/GPAM/NFYC/LIPA/SCD/APOE/SC5D/LDLRAP1/SCAP/CEBPA/FDFT1/PIP4P1/ELOVL6/NFE2L1/SREBF2/ACLY/SULT2B1/STARD3/HMGCR/ABCA1/LPCAT3/SNX17/CYB5R1/MSMO1/PCSK9/HMGCS1/INSIG1/FAXDC2/DGAT2/SOAT1/RXRA/SREBF1/NSDHL/MVK/CYP27A1/MVD/CLN6/EBPL/SCARB1/TM7SF2/ERG28/SMPD1/GBA/FDPS/ACAT2/G6PD/FASN/DHCR7/LSS/EBP/CYP1B1 |
| GOCC_SPECIFIC_GRANULE_MEMBRANE | -0.524738394 | -1.933104113 | 2.23E-05 | 0.001690049 | CD93/CLEC12A/RAP2B/SLC15A4/PTPRB/AGPAT2/MCEMP1/P2RX1/ADAM8/GPR84/AP1M1/DGAT1/LAMTOR2/VAMP8/SLC27A2/CYBA/TRPM2/CYBB/TOM1/SLC44A2/HVCN1/FPR2/PTPRJ/TNFRSF1B/CKAP4/STK10/DNAJC5/LAMTOR1/CD33/PLAU/BST1/CD53/ITGAL/ALDH3B1/MMP25/CLEC5A/CEACAM1/TSPAN14/ADGRG3/KCNAB2/LAIR1/ITGB2/CD177/LILRA3/C3AR1/ITGAM |
| GOCC_HEMOGLOBIN_COMPLEX | 0.915311187 | 1.973557582 | 2.32E-05 | 0.001735931 | HBB/HBA2/HBD/HBG2 |
| GOBP_CONNECTIVE_TISSUE_DEVELOPMENT | 0.435207746 | 1.648456346 | 2.48E-05 | 0.0018376 | SFRP2/SULF1/PDGFD/BMP6/EGR1/EDN1/LUM/BMP5/ARID5B/PRRX1/ITGB8/MIR21/BMP1/HIF1A/BMP3/CSGALNACT1/FRZB/CCN2/CCN1/SOX5/ARRDC3/MGP/BMPR1A/COL1A1/SLC39A14/LOX/NFIB/ATP7A/LRP6/ADAMTS12/SMAD3/SNAI2/EIF2AK3/MEF2C/BBS2/LTBP3/PIK3CA/PPARGC1A/MEX3C/ACTA2/UBB/SIRT1/IFT80/TRIP11/CD44/FGF2/LOXL2/ATF2/EFEMP1/EVC/EBF2/CRIP1/TRPS1/SOX6/RUNX2/MBOAT2/GREM1/SMAD5/SCIN/SPART/LNPK/MDK/GLG1/CHSY1/TAPT1/CFLAR/CCN3/WNT5A/SERPINH1/WT1/RARB/GLI3/SMAD1/PTPN11/OSR1 |
| GOBP_OXYGEN_TRANSPORT | 0.884063601 | 2.044649074 | 2.63E-05 | 0.001928916 | HBB/HBA2/HBD/HBG2/IPCEF1 |
| GOBP_POSITIVE_REGULATION_OF_SMOOTH_MUSCLE_CELL_MIGRATION | 0.685028148 | 2.040259356 | 2.67E-05 | 0.001934331 | POSTN/HAS2/ITGA2/IGF1/PDGFD/CCL5/NR4A3/MIR21/SEMA6D/ATP7A/DOCK7/DOCK4/RPS6KB1/BCL2/LPAR1/DOCK5 |
| GOMF_OXYGEN_CARRIER_ACTIVITY | 0.885659062 | 2.008828439 | 3.12E-05 | 0.002238533 | HBB/HBA2/HBD/HBG2/IPCEF1 |
| GOBP_POSITIVE_REGULATION_OF_LEUKOCYTE_DEGRANULATION | -0.760952811 | -2.134853946 | 3.24E-05 | 0.002299793 | GAB2/VAMP8/SPHK2/PTAFR/SYK/LAMP1/IL4R/ITGB2/CD177/GATA2/ITGAM/FGR |
| GOBP_REGULATION_OF_WOUND_HEALING | 0.500064514 | 1.795424646 | 3.54E-05 | 0.002450402 | CPB2/EDN1/SERPINE2/FAP/TNFAIP3/F11/PHLDB2/F2R/THBS1/CASK/CXCR4/GJA1/SERPINE1/PRKG1/SERPINB2/ADTRP/HBEGF/SLC12A2/CLASP2/SMAD3/KANK1/PROS1/UBASH3B/ARFGEF1/PDGFA/FGG/NFE2L2/C1QTNF1/CD9/FGF2/PLPP3/ANXA1/FERMT2/SRSF6/MIR29A/MYOZ1/ITGB1 |
| GOBP_CELL_REDOX_HOMEOSTASIS | -0.645407124 | -2.098180376 | 3.54E-05 | 0.002450402 | APEX1/CYBA/CYBB/TXN/TXN2/GCLC/HVCN1/RAC2/PRDX1/NCF1/GIT1/SLC2A10/GSR/NCF2/SLC11A1/TXNRD1/NCF4/NQO1 |
| GOCC_BASEMENT_MEMBRANE | 0.548592625 | 1.900919552 | 3.56E-05 | 0.002450402 | CCDC80/HMCN1/FREM1/COL15A1/FRAS1/FREM2/FBN1/DST/CASK/P3H2/LAMA3/COL4A3/EGFL6/ATRNL1/NID2/COL4A1/LAMA4/EFEMP2/LAMB1/FN1/LOXL2/DLG1/SPARC/COL18A1/COL4A2/ERBIN/THBS4/HSPG2/COL4A4/NTN4/SERPINF1/VWA2/LAMA1/LAMA2 |
| GOBP_ANTIGEN_RECEPTOR_MEDIATED_SIGNALING_PATHWAY | 0.436337109 | 1.654812671 | 3.64E-05 | 0.002480609 | THY1/ITK/PDE4D/TRAT1/GBP1/IGKC/IGHG1/SH2D1A/PRKCQ/IGHM/THEMIS/HLA-DQA1/CTLA4/CD38/HLA-DPB1/IGHA1/WNK1/NFATC2/RIPK2/DENND1B/PDE4B/BMX/BTN3A1/CBLB/PLEKHA1/NFKBIZ/CUL1/CD226/MALT1/PSME4/TEC/TNFRSF21/NFKB1/MEF2C/LAX1/MAP3K7/PTPN22/FBXW11/SLC39A10/HLA-DRA/ELF2/HLA-DPA1/BCL2/BTRC/PIK3CA/SKP1/BTN3A3/HLA-DQA2/ELF1/CD28/RC3H1/CD3G/HLA-DRB1/RFTN1/FOXP1/PRKCH/RC3H2/STAP1/HLA-DRB3/HLA-DQB2/CBFB/PTPN2/GRAP2/GCSAML/PAWR/HLA-DQB1/TXK/MS4A1/EZR/SLA2/TESPA1/FYN/FCGR2B/KLHL6/NCK1/PLCL2/KHDRBS1/TRAF6/PTPRC |
| GOCC_AZUROPHIL_GRANULE_MEMBRANE | -0.5964986 | -2.013223308 | 3.76E-05 | 0.00253466 | TOM1/NFAM1/ABCA13/CKAP4/DDOST/NCSTN/DNAJC5/LAMP1/LAMTOR1/RAB5C/ARL8A/SNAP29/CD68/BRI3/RAB3D/MGST1/ACP3/VNN1/CEACAM6/PIGR/C3AR1/FPR1 |
| GOBP_ANTIMICROBIAL_HUMORAL_RESPONSE | -0.455841792 | -1.795127274 | 4.14E-05 | 0.002748948 | BCL3/GAPDH/RNASE3/SLC11A1/WFDC2/RNASE6/LTF/PI3/DEFA1B/S100A8/LCN2/BPIFA1/S100A9/S100A12/BPIFB1 |
| GOBP_SMALL_MOLECULE_CATABOLIC_PROCESS | -0.336036087 | -1.468922742 | 4.15E-05 | 0.002748948 | TYMP/CYP4F12/GOT1/GLYCTK/PGM1/PNKD/ACSF3/MLYCD/AMDHD2/ACADS/TKFC/SULT1A2/ACAA1/ABCD1/BDH1/IDNK/ACAD10/APOBEC3F/GCDH/APOBEC2/MCEE/GLUL/GLB1/SARDH/PLA2G15/TP53/IRS2/ECI1/CTH/CRAT/TPI1/CBR3/ACAD8/APOE/UPB1/MTOR/SORD/HAL/BCAT1/APOBEC3A/SLC27A2/MPST/NUDT16/CRYM/DERA/SLC27A4/ADHFE1/HAGH/ECH1/PCCB/ADA2/PPM1K/UPP1/GALK1/ALDH4A1/ALDOA/NUDT5/SNX17/PNP/ABAT/ALDH1A1/GCSH/SULT1A1/ENO2/PGAM1/CDA/BCKDK/ADH7/GSK3A/TXN2/ALDH6A1/ALDH1L1/HMGCL/GALE/ACOT8/MCAT/HSD17B10/ASRGL1/NUDT7/CPT1A/SHMT1/INPP5K/AKR1A1/OGDH/SLC25A44/ENO1/ALDH3B1/RIDA/SRD5A3/PCK2/GAPDH/IMPA2/CYP27A1/CPT2/INPP5A/NAGK/ARG1/MGAT1/PCBD1/AHCY/SCARB1/PKM/ALDOC/PRODH/RENBP/FUT1/FAH/DCXR/AFMID/GNPDA1/BCKDHA/AKR1C3/PGD/ACAT2/BCAT2/TST/HK3/NPL/SHMT2/CBS/SULT1B1 |
| GOBP_FOREBRAIN_DEVELOPMENT | 0.39473168 | 1.565266174 | 4.25E-05 | 0.002784593 | INHBA/CXCL12/ROBO2/FGFR2/PLCB1/CDON/DCLK1/NR4A2/HIF1A/COL3A1/ROBO1/ATRX/FRS2/LRRK2/CCDC141/CXCR4/TOP2B/NF1/DNAJB1/PCM1/BMPR1A/FAT4/PRKG1/FOXP2/UBA6/ERBB4/DLC1/TWSG1/MFSD2A/RAPGEF2/CDK6/ZSWIM6/BTG2/NFIB/CDH2/TNR/SCYL2/ATP7A/LRP6/DOCK7/LEF1/BBS2/CHD7/SEMA5A/RAB3GAP1/APAF1/DYNC2H1/DIXDC1/PPARGC1A/NDNF/CEP120/SRGAP2C/ATP2B4/ALDH1A3/EZH2/UBB/SETD2/EFHC1/LRP2/LPAR1/PGAP1/LAMB1/RYK/RELN/SLC38A2/GNG12/HOOK3/METTL14/KIF5B/TOX/FGF10/TRA2B/PAFAH1B1/PLXNA4/MPP5/RPGRIP1L/MCPH1/KDM1A/METTL3/ETS1/SEMA3A/NIN/FYN/TACC1/ADCYAP1/PEX13/SIN3A/NOTCH2NLA/SRGAP2/ATP1B2/RBPJ/AVPR1A/KIF14/CASP3/RRM1/NCOA1/GNAO1/OPHN1/SLC8A1/WNT5A/SYNE2/RARB/CREB1/GLI3 |
| GOBP_NEGATIVE_REGULATION_OF_WOUND_HEALING | 0.578210647 | 1.906257603 | 4.35E-05 | 0.002826493 | CPB2/EDN1/SERPINE2/FAP/F11/PHLDB2/THBS1/CASK/GJA1/SERPINE1/PRKG1/SERPINB2/ADTRP/SLC12A2/CLASP2/SMAD3/PROS1/UBASH3B/PDGFA/FGG/C1QTNF1/CD9/FGF2/MYOZ1 |
| GOBP_REGULATION_OF_CHROMOSOME_ORGANIZATION | 0.419687676 | 1.612224112 | 4.46E-05 | 0.002866354 | RPS6KA5/PRKCQ/ATRX/MAP3K4/LRRK2/HNRNPU/CTNNB1/SMC5/MTF2/OGT/NIPBL/XRN1/PRKD1/NBN/TNKS/RIF1/GEN1/KMT2E/SENP6/TNKS2/HNRNPA2B1/DHX36/NSD3/CDC27/FMR1/PAXBP1/TPR/TENT4B/MNAT1/SMG1/HNRNPA1/PCID2/SFPQ/PPARGC1A/ATM/SIRT1/MPHOSPH8/TASOR/SETMAR/VEGFA/TCP1/SLF1/CENPF/USP7/NAF1/KMT2A/DUSP1/ING2/KNTC1/CENPE/TERF2IP/MCPH1/TET1/KDM1A/RIOK2/RAD50/NDC80/TERF1/ZNF207/WAPL/ATR/KAT7/BRD7/RAD21/LIF/YLPM1/PPP1R10/RTF1/HDAC8/AUTS2/PTTG2/HNRNPD/SART3/MRE11/JARID2/TOP2A/SIN3A/CUL3/NEK7/ANAPC1/APC/CCT8/MYB/SLF2/DLGAP5/CCT2/POT1/SMAD4/GNL3/DKC1/SNW1/ATF7IP/MIER1/WDR61 |
| GOBP_PHOSPHOLIPASE_C_ACTIVATING_G_PROTEIN_COUPLED_RECEPTOR_SIGNALING_PATHWAY | -0.503646655 | -1.890982183 | 4.50E-05 | 0.002869214 | GNA11/NMUR1/GNA14/C5AR2/FPR2/GRP/CX3CR1/SLC9A3R1/P2RY1/PTH1R/CHRM1/FPR3/F2RL3/C5AR1/ADRA1B/CMKLR1/NPR3/GPR4/S1PR1/EDNRB/AGTR1/C3AR1/FPR1/CXCR2/ADRA1A |
| GOMF_COLLAGEN_BINDING | 0.599695683 | 1.94666034 | 4.67E-05 | 0.002949592 | COL14A1/ASPN/ITGA2/ECM2/LUM/ANTXR1/ITGA9/CTSK/PCOLCE2/THBS1/DDR2/LOX/DCN/ADGRG6/SMAD3/MUSK/NID2/PDGFA/C1QTNF1/CD44/FN1/ITGB1/SPARC |
| GOBP_NEGATIVE_REGULATION_OF_TOLL_LIKE_RECEPTOR_SIGNALING_PATHWAY | -0.66389061 | -2.107418403 | 4.72E-05 | 0.002952478 | LY96/LYN/TICAM1/TYRO3/CD300A/TLR4/CD300LF/PTPRS/SMPDL3B/TLR6/LILRA2/ARRB2/CD14/BPIFB1 |
| GOBP_REGULATION_OF_MRNA_PROCESSING | 0.49001768 | 1.754687074 | 4.91E-05 | 0.0030417 | HNRNPU/RBM25/RBM39/MBNL2/RBFOX2/IWS1/TIA1/HNRNPA2B1/DDX5/DHX36/FMR1/SLTM/MBNL1/HNRNPA1/MBNL3/FAM172A/FXR1/YTHDC1/RBM5/DHX9/DDX17/WTAP/CPSF6/SRSF6/NCL/TRA2B/HNRNPK/AHCYL1/CCNT1/SAFB/SRSF10/RBM7/SON/SRSF7/NSRP1/NCBP2/HSPA8/CWC22/CELF2/KHDRBS1/NUDT21/CDC73/DYRK1A/REST/TRA2A/NUP98/PTCD2/RBM15/SNW1/RBMX/SMU1/NOVA1/ZC3H14/KHDRBS2/JMJD6/SRPK2/PAPOLA/SRSF4/LARP7/BARD1 |
| GOMF_SULFUR_COMPOUND_BINDING | 0.422605494 | 1.614475321 | 5.32E-05 | 0.003267588 | POSTN/CCDC80/ITGA2/ECM2/CFH/ACADL/SERPINE2/SELP/FGFR2/THBS2/DPYSL3/F11/MMP7/PCOLCE2/RSPO3/THBS1/FGF7/LTBP2/FBN1/LPL/CCN2/CCN1/RYR2/CXCL11/CXCL10/HPSE2/FST/TNXB/LXN/ANOS1/SFRP1/HBEGF/SLIT3/FSTL1/ADAMTS3/KMT5B/SCP2/ZNF146/SEMA5A/N6AMT1/FGF1/CXCL13/NDNF/BMT2/PCCA/GSTM3/TENM1/EFEMP2/VEGFA/FGF2/FN1/LANCL1/CTSG/FGFR1/NRP1/FGF10/PAFAH1B1/ACACB/GSTM2/ADAMTS5 |
| GOCC_PROTON_TRANSPORTING_TWO_SECTOR_ATPASE_COMPLEX | -0.620447602 | -2.052768705 | 5.53E-05 | 0.00336551 | ATP6V1E1/ATP6V1G1/ATP5F1A/ATP6V0E1/DMAC2L/ATP6V0C/ATP6V0E2/ATP6V1C2/ATP6V1A/ATP5F1EP2/ATP5F1E/ATP6V1B2/ATP5F1D/ATP5MPL/TCIRG1/TMEM199/ATP5PF/ATP6V0D2/ATP5ME/ATP5PO/ATP5PD/ATP5MD/ATP5MC1/ATP6V0B/ATP5MF/ATP6V0D1/ATP6AP1/ATP6V1F |
| GOMF_HYDROLASE_ACTIVITY_HYDROLYZING_O_GLYCOSYL_COMPOUNDS | -0.521738069 | -1.914571225 | 5.76E-05 | 0.003473453 | HEXD/CTBS/GUSB/MYORG/MOGS/HEXA/GBA2/MAN1C1/ADPRS/FUCA1/GLB1/KL/GANAB/GLB1L/NAGLU/MAN2A2/GLA/NAGPA/MAN2B1/FUCA2/AMY2A/NEU1/CHIA/GLB1L2/GM2A/CEMIP/HYAL2/NAGA/GBA/HYAL1/MGAM/CHIT1 |
| GOBP_DIGESTION | -0.461714651 | -1.805097118 | 5.91E-05 | 0.003534926 | NR1H3/SNX10/NOD2/HAMP/TFF3/SLC46A1/LPCAT3/SLC26A6/NR1H2/SLC5A1/CAPN9/AMY2A/CHIA/TLR4/CHRM1/HRH2/SCARB1/PIR/AQP5/NPR3/AQP1/MUC4/AKR1C2/SERPINA3/MGAM/CHIT1 |
| GOBP_MITOCHONDRIAL_ELECTRON_TRANSPORT_NADH_TO_UBIQUINONE | -0.615849271 | -2.045402051 | 6.15E-05 | 0.003645105 | NDUFB1/NDUFA13/NDUFA1/NDUFV2/NDUFS7/NDUFB11/NDUFC2/NDUFC2-KCTD14/COQ9/NDUFA7/NDUFA4/NDUFS8/NDUFAB1/NDUFA6/PINK1/NDUFB6/NDUFV3/NDUFB4/NDUFB9/NDUFA12/NDUFS2/NDUFA2/NDUFB3/NDUFB7/NDUFV1/NDUFAF1/NDUFB10 |
| GOCC_MHC_CLASS_II_PROTEIN_COMPLEX | 0.844997175 | 2.023451322 | 6.35E-05 | 0.003732589 | HLA-DQA1/HLA-DPB1/HLA-DOA/HLA-DRA/HLA-DPA1/HLA-DQA2/CD74/HLA-DOB/HLA-DRB1/HLA-DRB3/HLA-DQB2/HLA-DQB1/HLA-DMA |
| GOMF_COMPLEMENT_RECEPTOR_ACTIVITY | -0.89517616 | -1.994599193 | 6.43E-05 | 0.003743078 | C5AR2/FPR2/FPR3/C5AR1/CMKLR1/C3AR1/FPR1/CR1 |
| GOBP_NEGATIVE_REGULATION_OF_EXTRINSIC_APOPTOTIC_SIGNALING_PATHWAY | 0.529928041 | 1.852404634 | 6.55E-05 | 0.003766678 | SFRP2/IGF1/HGF/BMP5/TNFAIP3/EYA4/THBS1/PHIP/ITGAV/SERPINE1/HSPA1B/HSPA1A/RB1CC1/ZMYND11/SNAI2/SH3RF1/MCL1/BIRC6/RPS6KB1/FGG/BCL2/TNFRSF10B/ITGA6/YAP1/IL7/ICAM1/DDX3X/FGF10/FAS/ACVR1/PF4/FYN |
| GOBP_COMPLEMENT_RECEPTOR_MEDIATED_SIGNALING_PATHWAY | -0.895133949 | -2.062114057 | 6.58E-05 | 0.003766678 | C5AR2/FPR2/FPR3/C5AR1/CMKLR1/C3AR1/FPR1/CR1 |
| GOMF_SODIUM_INDEPENDENT_ORGANIC_ANION_TRANSMEMBRANE_TRANSPORTER_ACTIVITY | -0.830069136 | -2.124860185 | 6.82E-05 | 0.003870573 | SLCO2B1/SLCO2A1/SLCO1A2/SLCO4A1 |
| GOBP_ALCOHOL_METABOLIC_PROCESS | -0.34667306 | -1.502941247 | 6.91E-05 | 0.003889296 | SC5D/LDLRAP1/SCAP/CEBPA/FDFT1/PIP4P1/ELOVL6/NFE2L1/SREBF2/SPHK2/ACLY/SULT2B1/STARD3/HMGCR/ABCA1/LPCAT3/GALK1/SPR/SNX17/CYP2R1/DHFR/ALDH1A1/CD244/SULT1A1/PLCB2/MSMO1/IDH3G/ADH7/PTAFR/INPPL1/PCSK9/HMGCS1/INSIG1/DGAT2/PLPP2/DPAGT1/SOAT1/RXRA/GPER1/ITPK1/PLCD3/INPP5K/SREBF1/NSDHL/NAAA/P2RY1/AKR1A1/H6PD/ALDH3B1/IDH2/SRD5A3/PCK2/PTH1R/IMPA2/MVK/CYP27A1/CACNA1H/INPP5A/DHRS13/PGP/MVD/DHDDS/PCBD1/CLN6/IDH1/SCARB1/TM7SF2/SMPD1/GBA/PLCD1/AKR1C3/DHRS3/FDPS/ACAT2/G6PD/FASN/DHCR7/LSS/EBP/CYP1B1/ACSS2/AKR1C2/SULT1B1 |
| GOBP_NEGATIVE_REGULATION_OF_LEUKOCYTE_MEDIATED_IMMUNITY | -0.574331857 | -1.934670234 | 7.06E-05 | 0.003941771 | NOD2/HLA-E/LGALS9/SERPINB4/C4BPB/PARP3/BCL6/JAK3/CD300A/ARG1/FOXJ1/PTPN6/CEACAM1/CCR2/LILRB4/LILRB1/FOXF1/ARRB2/CR1/HMOX1 |
| GOBP_BONE_DEVELOPMENT | 0.451273471 | 1.676694154 | 7.57E-05 | 0.004196356 | SFRP2/HAS2/IGF1/KIT/SULF1/BMP6/SFRP4/FGFR2/FREM1/ITGB6/LRRC17/TGFB3/PHEX/CSGALNACT1/FBN1/RAB23/MMP16/GJA1/FBXW7/CLDN18/COL1A1/FAT4/LOX/RANBP3L/AKAP13/BBX/NAB1/LRP6/MEF2C/LTBP3 |
| GOBP_STEROL_BIOSYNTHETIC_PROCESS | -0.532426496 | -1.890423469 | 8.42E-05 | 0.004627767 | LBR/CYB5R3/GPAM/NFYC/SCD/APOE/SC5D/SCAP/FDFT1/ELOVL6/SREBF2/ACLY/HMGCR/LPCAT3/CYB5R1/MSMO1/HMGCS1/INSIG1/FAXDC2/SREBF1/NSDHL/MVK/MVD/TM7SF2/ERG28/FDPS/ACAT2/G6PD/FASN/DHCR7/LSS/EBP |
| GOCC_SPECIFIC_GRANULE_LUMEN | -0.57180974 | -1.954012996 | 8.57E-05 | 0.004674406 | CXCL1/JUP/TOLLIP/CANT1/ELANE/TCN1/DEFA4/ACAA1/PGLYRP1/RETN/GHDC/CYFIP1/OSCAR/LRG1/QSOX1/HP/SLPI/CTSD/PTX3/ORM1/NEU1/CNN2/ARG1/PTPN6/DNASE1L1/QPCT/CFP/LTF/MMP8/OLFM4/CHIT1/LCN2 |
| GOBP_GRANULOCYTE_MIGRATION | -0.43429246 | -1.726856965 | 8.89E-05 | 0.004788997 | CSF1R/RAC2/CCL2/MPP1/BST1/PIK3CD/CD300A/MYD88/S100A14/CCL23/RHOG/C5AR1/CMKLR1/PECAM1/ITGB2/CD177/ADGRE2/PREX1/SLAMF8/FCER1G/RIPOR2/C3AR1/CXCR2/CXCR1/CSF3R/S100A8/SAA1/S100A9/S100A12 |
| GOMF_TOLL_LIKE_RECEPTOR_BINDING | -0.868334674 | -2.067509985 | 8.92E-05 | 0.004788997 | SYK/LY96/TLR2/UNC93B1/MYD88/TLR6/TLR1/S100A8/S100A9 |
| GOBP_SULFUR_COMPOUND_METABOLIC_PROCESS | -0.338198393 | -1.472167004 | 9.12E-05 | 0.004856793 | B4GAT1/NDST2/SLC35D2/B3GALT6/PDHB/CHST1/BGN/GGT5/HEXA/ACSF3/CHST5/SLC25A10/MLYCD/SLC35B2/MTHFR/CNDP2/B4GALT2/GPAM/SULT1A2/PDK2/ABCD1/SCD/ACOT2/ISCA2/CHPF2/GCDH/MCEE/AMD1/ADI1/GLB1/GSTO1/CSPG5/CTH/DGAT1/CSPG4/OPLAH/ACSM5/MMACHC/NDST1/MPST/SUOX/B4GALT3/GGT3P/CLIC1/HAGH/CHST2/PCCB/SQOR/ELOVL6/GAL3ST4/MICAL1/ACLY/SULT2B1/ELOVL1/GALNS/PEMT/SLC25A19/XYLT1/ACSL1/STAT5A/FITM2/SULT1A1/PAPSS2/ACOT1/GSS/TXN2/GCLC/NUBP1/SLC5A6/GGTLC2/GSTP1/PRELP/HMGCS1/CHST6/DGAT2/DLAT/HMGCL/ACOT8/DSE/GSTA1/NUDT7/CHST15/HS3ST3B1/GSTM4/AKR1A1/OGDH/GSTA2/GCLM/MGST3/MGST2/MVK/TECR/SLC7A11/CIAO2A/SLC25A1/GSR/MVD/B3GNT7/AHCY/IDH1/GSTT2/TPST2/MGST1/ACP3/GSTT1/GGTLC1/ACOT13/DSEL/CTNS/GSTA4/G6PD/HYAL1/FASN/TST/ACSS2/CBS/SULT1B1/PHGDH |
| GOBP_COLLAGEN_FIBRIL_ORGANIZATION | 0.6073502 | 1.889664248 | 9.57E-05 | 0.005056895 | SFRP2/COL14A1/LUM/DPT/COL3A1/DDR2/NF1/COL1A1/COL5A2/LOX/TNXB/COL12A1/COL1A2/ATP7A/ADAMTS3 |
| GOMF_GTPASE_ACTIVATOR_ACTIVITY | 0.42379653 | 1.616929887 | 9.88E-05 | 0.005178597 | RGS1/RGS5/THY1/PLCB1/PREX2/ERRFI1/CHN1/ARHGAP21/RASAL2/ARHGAP42/IQGAP2/LRRK2/NF1/TBC1D4/SRGAP1/ARHGAP15/RGS6/RASGRP3/RABGAP1L/SIPA1L1/DLC1/MYO9A/RAPGEF2/RGS4/RGS2/STXBP5/RANBP3L/SOS1/RASA2/DOCK1/RGPD1/RGS16/ARHGAP24/RANBP2/TBC1D15/RASA1/DNM1L/USP6NL/RALGAPA1/ARHGEF12/TBC1D30/RAB3GAP2/RAB3GAP1/DOCK4/ARHGAP5/ASAP2/RALGAPA2/ARHGAP18/RAP1GAP/TBCK/TBC1D8B/ARFGAP3/JUN/DOCK5/ALS2/RACGAP1/SIPA1L2/CHML/HACD3/SGSM2/LARS1/TBC1D22B/ARHGEF6/TBC1D19/FAM13B/RABEP1/NRP1/RABGAP1/ARHGAP20/TAGAP/CHM/EVI5 |
| GOBP_HEART_MORPHOGENESIS | 0.421915405 | 1.595571382 | 0.000100792 | 0.005243488 | SFRP2/HAS2/ANKRD1/ROBO2/FGFR2/BMP5/PDCD4/ZFPM2/AHI1/SEMA3C/MIR21/FLRT2/HIF1A/PKP2/ROBO1/CPE/CCN1/RYR2/ARID2/CTNNB1/MIB1/BMPR1A/NIPBL/TTN/FAT4/MICAL2/DLC1/ADGRG6/SOS1/NPHP3/PKD2/SLIT3/SMAD3/SNAI2/SPRY1/MEF2C/RBP4/CHD7/NPY1R/BBS7/INSR/FHL2/LRP2/VEGFA/YAP1/NSD2/TGFB2/SEC24B/ATF2/MDM4/NRP1 |
| GOBP_NEGATIVE_REGULATION_OF_RESPONSE_TO_OXIDATIVE_STRESS | 0.761625322 | 2.000176091 | 0.000112097 | 0.00578745 | HGF/NCOA7/NR4A3/MIR21/PDE8A/LRRK2/H19/OXR1/MCTP1/MET/NFE2L2 |
| GOBP_NADH_DEHYDROGENASE_COMPLEX_ASSEMBLY | -0.574143678 | -1.946356863 | 0.000115228 | 0.005904348 | NDUFS3/NDUFAF3/TMEM126B/NDUFB8/ECSIT/NDUFB1/NDUFA13/NDUFA1/NDUFV2/NDUFS7/NDUFB11/NDUFC2/OXA1L/NDUFA7/NDUFAF4/TAZ/NDUFS8/NDUFAB1/NDUFA6/NDUFB6/NDUFV3/FOXRED1/NDUFB4/NDUFB9/NDUFA12/NDUFS2/NDUFA2/NDUFB3/BCS1L/NDUFB7/NDUFV1/NDUFAF1/NDUFB10/DMAC2 |
| GOBP_MYELOID_LEUKOCYTE_MIGRATION | -0.38611125 | -1.592061335 | 0.00011757 | 0.005979405 | C5AR2/FPR2/AIF1/SYK/LYN/EMILIN1/CSF1R/RAC2/CX3CR1/CCL2/MPP1/BST1/PDGFB/PIK3CD/P2RX4/CD300A/MYD88/VEGFD/SIRPA/S100A14/CCL23/MMP14/DDT/RHOG/C5AR1/CMKLR1/PECAM1/TREM2/CCR2/ITGB2/CD177/EDNRB/ADGRE2/PREX1/SLAMF8/FCER1G/PLA2G7/RIPOR2/C3AR1/CCR1/CXCR2/CXCR1/CSF3R/S100A8/SAA1/S100A9/S100A12 |
| GOBP_MUSCLE_TISSUE_DEVELOPMENT | 0.388012722 | 1.542629912 | 0.000119059 | 0.006010295 | IGF1/ANKRD1/ADAMTS9/EGR1/NR1D2/EDN1/FGFR2/CDON/BMP5/FZD7/ZFPM2/SEMA3C/PKP2/TIPARP/FRS2/MEOX2/HLF/FOS/ZFAND5/RYR2/ARID2/HNRNPU/SVIL/NF1/GJA1/MYH11/BMPR1A/AKAP6/TTN/DSG2/PGM5/SGCD/LOX/DCN/ERBB4/RGS4/RGS2/AKAP13/PKD2/BTG2/CXADR/PI16/SGCB/CCNT2/DDX5/SMAD3/ATF3/LEF1/MEF2C/SORBS2/MYOCD/RBP4/CHD7/PDLIM5/RPS6KB1/BCL2/COPS2/PPARGC1A/FHL2/MYOM1/LRP2/EFEMP2/VEGFA/YAP1/FGF2/PPP3CA/DDX17/CENPF/GJC1/MYOZ1/DLG1/TGFB2/IGF2 |
| GOBP_NEGATIVE_REGULATION_OF_COAGULATION | 0.607795019 | 1.885127277 | 0.000122991 | 0.006147488 | CPB2/EDN1/SERPINE2/FAP/F11/THBS1/SERPINE1/PRKG1/SERPINB2/ADTRP/PROS1/UBASH3B/PDGFA/FGG/PROCR/C1QTNF1/CD9 |
| GOBP_GASTRULATION | 0.445515931 | 1.653124369 | 0.000123581 | 0.006147488 | SFRP2/ITGA2/INHBA/ITGB3/FGFR2/FZD7/PHLDB2/NR4A3/FRS2/GPC3/ITGA4/ITGAV/GJA1/LAMA3/APLN/KIF16B/APLNR/BMPR1A/WNK1/COL5A2/COL12A1/TWSG1/SFRP1/NPHP3/ACVR2A/CLASP2/LRP6/SMAD3/LEF1/EXOC4/ITGA8/SETD2/TASOR/LAMB1/RNF2/FN1/SYF2/DUSP1/ITGB1/SMAD2/SUPT20H/ITGA7/COL4A2/ADIPOQ/ACVR1/DUSP4/POGLUT1/PRKAR1A/EXT2/RTF1/DKK1/CDC73/CUL3 |
| GOBP_HUMORAL_IMMUNE_RESPONSE | -0.357465193 | -1.512776615 | 0.00012943 | 0.006320146 | C5AR2/WFDC12/GNLY/SLPI/C1QC/TFE3/CCL2/BST1/BCL3/C1RL/GAPDH/CFB/SPNS2/RNASE3/NOTCH1/FOXJ1/PHB/FCN1/PTPN6/SLC11A1/WFDC2/C1R/CFP/C5AR1/RNASE6/TREM2/LTF/PI3/VSIG4/C3AR1/DEFA1B/CR1/FCN3/S100A8/LCN2/BPIFA1/S100A9/S100A12/BPIFB1 |
| GOBP_RESPONSE_TO_XENOBIOTIC_STIMULUS | -0.465272991 | -1.797608918 | 0.000129589 | 0.006320146 | GSTO2/CYP2B6/AKR1C1/CYP2A6/TIGAR/PON3/GSTA3/CYP2D7/CYB5B/RHBDD3/E2F1/CYP2A13/CYB5R3/SULT1A2/ACAA1/AIP/GSTO1/CBR3/CYP2G1P/APOBEC3A/CES2/GUK1/CYP2S1/CYP2R1/ACSL1/SULT1A1/GSS/GSTP1/ACY1/GSTA1/POR/MTARC1/GSTM4/PCNA/GSTA2/MGST3/MGST2/MGST1/ALDH3A1/CYP2F1/SCGB1A1/AOC3/GSTA4/NQO2/CYP1B1/SULT1B1/NQO1/S100A12 |
| GOBP_SODIUM_INDEPENDENT_ORGANIC_ANION_TRANSPORT | -0.820401716 | -2.10011295 | 0.000129834 | 0.006320146 | SLCO2B1/SLCO2A1/SLCO1A2/SLCO4A1 |
| GOBP_ALCOHOL_BIOSYNTHETIC_PROCESS | -0.426765554 | -1.694059264 | 0.000137822 | 0.006659795 | APOE/IP6K3/SC5D/SCAP/FDFT1/ELOVL6/SREBF2/SPHK2/ACLY/HMGCR/LPCAT3/SPR/CYP2R1/DHFR/CD244/MSMO1/PTAFR/HMGCS1/INSIG1/GPER1/SREBF1/NSDHL/P2RY1/H6PD/SRD5A3/PCK2/PTH1R/IMPA2/MVK/CYP27A1/CACNA1H/PGP/MVD/DHDDS/PCBD1/TM7SF2/GBA/FDPS/ACAT2/G6PD/FASN/DHCR7/LSS/EBP |
| GOCC_MICROFIBRIL | 0.859613586 | 1.949752779 | 0.000138766 | 0.006659795 | LTBP1/MFAP4/MFAP5/FBN1/MFAP2/LTBP4/EFEMP2 |
| GOBP_RESPONSE_TO_BMP | 0.458192581 | 1.689735221 | 0.000140136 | 0.006678526 | SFRP2/SULF1/BMP6/EGR1/SFRP4/HIVEP1/BMP5/PDCD4/TGFB3/MIR21/FBN1/GPC3/CCN1/BMPR1A/FST/TWSG1/SFRP1/SMAD9/SMURF2/ACVR2A/ELAPOR2/DDX5/ADAMTS12/SMAD3/FSTL1/SKIL/NEO1/LEF1/CTDSPL2/SFRP5/HTRA1/ZFYVE16/PPARG/SMURF1/LRP2/LEMD3/TGFB2/CRIM1/TRIM33/USP9X/SMAD2 |
| GOBP_PEPTIDYL_SERINE_MODIFICATION | 0.394075166 | 1.542511325 | 0.000141149 | 0.006680064 | SFRP2/PDE4D/HGF/CNKSR3/DCLK1/PLK2/RPS6KA5/STK32A/MARK1/PRKCQ/STK38L/CAMK4/MAP3K13/TOP1/LRRK2/GALNT1/CAPRIN2/CLK1/WNK1/RIPK2/PKN2/DCN/MAPK8/PRKD1/HSP90AA1/PRKCI/TNKS/CAMK2D/TFRC/AKAP9/RPS6KA6/DOCK7/RPS6KA3/EIF2AK3/SMG1/FNIP2/DMD/RPS6KB1/BCL2/TLK1/PRKAA2/PIK3CA/RICTOR/NDNF/BRAF/DYRK3/HIPK3/ATM/POGLUT2/ATP2B4/CSNK1A1/PWP1/ROCK1/TAF1/PLCL1/CDK1/ANGPT1/TENM1/GADD45A/VEGFA/CD44/ERN1/PRKCH/VRK2/FNIP1/TBK1/MAST4/CSNK1G3/CAMK1D/TERF2IP/HSP90AB1/GALNT3/MAPK9/SMYD3/ROCK2/MARK3/ATR/CSF3/INPP5F/GGNBP2/MORC3/POGLUT1/MASTL/NCK1/LIF/DKK1/DYRK2/POGLUT3/PLCL2 |
| GOBP_SEQUESTERING_OF_METAL_ION | -0.825153622 | -2.065141451 | 0.000148706 | 0.006989175 | AP3D1/S100A8/LCN2/S100A9 |
| GOCC_COLLAGEN_TRIMER | 0.539856052 | 1.843501934 | 0.000150619 | 0.007030606 | COL14A1/COL6A6/COL15A1/LUM/COLEC10/COL6A3/COL3A1/C1QTNF3/COL1A1/COL5A2/LOX/DCN/COL12A1/COL4A3/COL1A2/C1QTNF7/COLEC12/WDR33/MSR1/COL4A1/SFTPD/C1QTNF1 |
| GOMF_FIBRONECTIN_BINDING | 0.707762169 | 1.948752854 | 0.0001524 | 0.007065356 | SFRP2/CCDC80/ITGB3/CTSK/THBS1/CCN2/ITGA4/ITGAV/IGFBP3/VEGFA/SDC4/ITGB1/FBLN1 |
| GOBP_CELL_MATRIX_ADHESION | 0.426069489 | 1.606274074 | 0.000161429 | 0.007384203 | POSTN/VCAM1/THY1/ITGA2/CCL21/ECM2/EPHA3/ADAMTS9/ITGB3/FREM1/ITGB6/ITGBL1/CD96/PHLDB2/MACF1/THBS1/COL3A1/CCN2/UTRN/ITGA4/CASK/ITGAV/LIMCH1/NF1/PTPRK/SERPINE1/MAP4K4/DLC1/ARHGEF7/SFRP1/CDK6/CLASP2/RASA1/BCL2L11/ADAMTS12/SMAD3/MKLN1/SNED1/NID2/FGG/BCL2/SLK/ITGA8/ROCK1/SGCE/LIMS1/EFEMP2/VEGFA/CD44/FN1/JAM3/FERMT2/VCL/SDC4/ITGB1 |
| GOBP_CELL_ADHESION_MEDIATED_BY_INTEGRIN | 0.557808289 | 1.841364748 | 0.000161445 | 0.007384203 | SFRP2/ITGA2/CCL21/ITGB3/ITGB6/ITGBL1/CCL5/ITGB8/FBN1/ITGA4/ITGAV/WNK1/SERPINE1/DPP4/PDE3B/SNAI2/PIK3CG/IFT74/CXCL13/PLPP3/JAM3/ICAM1/TGFB2/ITGB1 |
| GOBP_TRANSLATIONAL_ELONGATION | -0.444836541 | -1.739111281 | 0.000163726 | 0.007438609 | DAP3/TRNAU1AP/MRPL36/TSFM/MRPL38/MRPL10/MRPL27/RPLP2/TUFM/EEF1B2/MRPL22/GADD45GIP1/MRPS12/MRPL13/MRPL48/MRPS17/MRPL46/CHCHD1/MRPL20/MRPL45/MRPS28/MRPL30/EEF2K/MRPL17/MRPS21/MRPL12/OXA1L/MRPL14/ERAL1/MRPS23/MRPL16/MRPL40/RPLP1/MRPL24/MRPL9/MRPS35/MRPL23/MRPL35/MRPL49/MRPL21/MRPS36/MRPS2/MRPS25/MRPS33/MRPL34/MRPL4/MRPL18/MRPL41/ABTB1/MRPL58/MRPS18C/MRPL57/MRPL11/AURKAIP1/MRPS11/MRPL33/MRPL53/MRPL28/MRPS15/MRPS16/MRPL37/MRPS18B/MRPS34/EIF5AL1 |
| GOBP_REGULATION_OF_MYELOID_LEUKOCYTE_MEDIATED_IMMUNITY | -0.581025915 | -1.943398949 | 0.000169244 | 0.0076036 | VAMP8/FES/HLA-E/LGALS9/SPHK2/PTAFR/SYK/UNC13D/LYN/PRAM1/RAC2/TICAM1/IL4R/MAVS/CD300A/ARG1/STXBP2/CCR2/ITGB2/CD177/ADGRE2/FOXF1/GATA2/ITGAM/FGR/HMOX1 |
| GOBP_POSITIVE_REGULATION_OF_CELL_PROJECTION_ORGANIZATION | 0.388571324 | 1.532738392 | 0.000170678 | 0.0076036 | ENPP2/ITGA2/CCL21/KIT/ANKRD1/EPHA3/CXCL12/ROBO2/HGF/CNTN1/BMP5/EPHA4/DPYSL3/RUFY3/TGFB3/MACF1/MIR21/PTPRD/ROBO1/MAP3K13/VLDLR/CAPRIN2/ENC1/PPP1R9A/EPS8/PLCE1/RAPGEF2/PRKD1/FNBP1L/RGS2/ARHGEF7/PRKCI/DZIP1/CCP110/MNS1/DNM1L/ATP7A/DHX36/SKIL/KIDINS220/FMR1/ABL2/SEMA5A/CNR1/PIK3CA/ZDHHC15/GOLGA4/NDNF/CEP120/NFE2L2/DOCK11/EZH2/DNM3/SETX/TENM1/ITGA6/VEGFA/RALA/RELN/MAP1B/FN1/FBXO31/SERPINI1/CAMK1D/NLGN1/CCL19/TOX/TENM3/NRP1/OPA1/SCARB2/PLXNA4/KDM1A/TMEM30A/RAB8B/BHLHB9/NIN/FYN/SEPTIN7/CX3CL1/NCK1/ADCYAP1/AUTS2/COBL/ABI2/FBXW8/MDK/APC/MIR222/RP1/ATP1B2/PAK3/TAPT1/SERPINF1/CFLAR/IST1/NCKAP1/WNT5A/DBN1/EIF4G2/TIAM1 |
| GOCC_CHROMOSOME_CENTROMERIC_REGION | 0.430990091 | 1.605426444 | 0.000170704 | 0.0076036 | SMC4/ATRX/PELI1/HNRNPU/SMC6/SMC5/CENPC/AHCTF1/TP53BP1/SEPTIN6/UHRF2/CLASP2/STAG2/PPP1R12A/PDS5A/FMR1/KMT5B/TPR/STAG1/FBXW11/MIS18BP1/BOD1L1/UVRAG/CSNK1A1/NUP107/CENPF/SEPTIN2/KNTC1/CENPE/PAFAH1B1/KIF18A/CBX1/NDC80/ZNF207/WAPL/NUP160/KNL1/KAT7/SEPTIN7/RAD21/FBXO28/PDS5B/DYNLT3/TOP2A/SIN3A/PHF6/BOD1/SGO1/APC/ORC2/SUV39H2/PKHD1/NUP98/SMC3/NUP133/BAZ1B/CENPW/CENPN/CFDP1/SPAG5/PPP2CA/ITGB3BP/NCAPD3/ZNF330/HELLS/DSCC1/ESCO2/XPO1/MEAF6/BUB3/MAD2L1/MTBP/CKAP5 |
| GOCC_CYTOCHROME_COMPLEX | -0.665165332 | -1.994453802 | 0.000175736 | 0.007771374 | COX6A1/COX7C/UQCC3/UQCRHL/UQCRFS1/COX10/UQCRC1/COX8A/COX4I1/COA6/NDUFA4/C15orf48/NDUFA4L2/UQCR10/UQCRQ/CYC1/BCS1L/COX4I2/COX5A/COX7B/COX6B1 |
| GOMF_EXTRACELLULAR_MATRIX_STRUCTURAL_CONSTITUENT_CONFERRING_COMPRESSION_RESISTANCE | 0.761461992 | 1.983169941 | 0.000176752 | 0.007771374 | ASPN/OGN/PRG4/LUM/DCN |
| GOMF_COMPLEMENT_BINDING | -0.768282368 | -2.079286082 | 0.000178094 | 0.007780214 | PTX3/CFB/PHB/C5AR1/ITGB2/VSIG4/ITGAM/CR1 |
| GOBP_CYCLIC_NUCLEOTIDE_METABOLIC_PROCESS | 0.649618915 | 1.926630688 | 0.000182224 | 0.007909908 | PDE1A/PDE7B/PDE4D/PDE8B/RORA/PDE8A/PDE5A/PDE10A/PDE2A/PDE4B |
| GOBP_INNATE_IMMUNE_RESPONSE_IN_MUCOSA | -0.739090414 | -2.10561746 | 0.000183679 | 0.007922603 | H2BC8/H2BC6/DEFA4/H2BC7/H2BC12/H2BC21/NOD2/H2BC4/RNASE3/LTF/DEFA1B/RNASE2/BPIFB1 |
| GOMF_LYASE_ACTIVITY | -0.389393189 | -1.582675407 | 0.000186337 | 0.007986714 | GOT1/ADCY5/NPR1/MLYCD/ME1/ALAD/TKFC/ADCY1/MOCOS/AMD1/HCCS/ACO2/ECI1/CTH/TPI1/GGCX/POLL/HAL/DERA/DDTL/PAICS/CYP2S1/ALDOA/ENO2/ADCY9/FAHD1/HMGCL/UROD/ADCY4/SHMT1/BST1/GSTM4/ENO1/CA3/PCK2/MGST3/MGST2/ECHDC3/MVD/PCBD1/ALOX5AP/ALDOC/DDT/TBXAS1/BCKDHA/FASN/NPL/CYP1B1/SHMT2/CBS/CA4/RNASE2 |
| GOBP_IRON_ION_TRANSPORT | -0.499162635 | -1.782764252 | 0.000200524 | 0.008541088 | ARHGAP1/RAB11B/ATP6V1B2/HAMP/SLC46A1/TCIRG1/ATP6V0D2/FLVCR2/ATP6V0B/SLC39A8/ATP6V0D1/ATP6AP1/SLC25A37/SLC48A1/MCOLN1/ATP6V1F/DNM2/ABCB6/SLC11A1/STEAP3/LTF/SLC6A9/C1orf194/LCN2 |
| GOMF_CYTOKINE_RECEPTOR_ACTIVITY | -0.485135948 | -1.799374834 | 0.000202878 | 0.008587656 | CRLF1/FLT3/IL10RB/CX3CR1/IL4R/CSF2RB/IL10RA/ACKR4/IL15RA/CMKLR1/IL18RAP/CCRL2/CCR2/IL17RA/IL1RL1/CCR1/CXCR2/CXCR1/CSF3R/IL1R2 |
| GOMF_RECEPTOR_REGULATOR_ACTIVITY | 0.356439549 | 1.455224396 | 0.000204607 | 0.008607385 | SFRP2/OGN/CCL21/MACC1/INHBA/IGF1/SEMA3D/PDGFD/AREG/BMP6/CXCL9/EDN1/CXCL12/C5/HGF/BMP5/COLEC10/CCL5/TGFB3/TNFSF8/SEMA3C/FLRT2/BMP1/BMP3/FGF7/PPBP/FBN1/CCN2/IL33/CXCL11/CCL18/APLN/CXCL10/DPP4/FST/SEMA6D/NTS/CXCL14/HBEGF/FGF14 |
| GOMF_ABC_TYPE_XENOBIOTIC_TRANSPORTER_ACTIVITY | 0.868765658 | 1.932270286 | 0.000218627 | 0.009140753 | ABCG2/ABCB1/ABCA8 |
| GOBP_REGULATION_OF_EXTRINSIC_APOPTOTIC_SIGNALING_PATHWAY | 0.464103421 | 1.691790643 | 0.000220973 | 0.00918251 | SFRP2/INHBA/IGF1/HGF/BMP5/TNFAIP3/EYA4/THBS1/PHIP/CYLD/ITGAV/SERPINE1/HSPA1B/HSPA1A/SFRP1/RB1CC1/ZMYND11/SNAI2/SH3RF1/SKIL/ATF3/MCL1/BIRC6/RPS6KB1/FGG/BCL2/PPP2R1B/TNFRSF10B/ITM2C/ITGA6/YAP1/IL7/ICAM1/DDX3X/FGFR1/FGF10/FAS/STK3/FAF1/ACVR1/PF4/FYN/FEM1B |
| GOBP_REGULATION_OF_COAGULATION | 0.556559009 | 1.828954415 | 0.00023407 | 0.009667815 | CPB2/EDN1/SERPINE2/FAP/F11/F2R/THBS1/SERPINE1/PRKG1/SERPINB2/ADTRP/PROS1/UBASH3B/PDGFA/FGG/PROCR/NFE2L2/C1QTNF1/CD9 |
| GOBP_MITOCHONDRIAL_TRANSPORT | -0.361715483 | -1.53503727 | 0.000241428 | 0.009911619 | ATP5F1A/SLC25A5/BID/SFXN3/TMEM14C/NDUFA13/SLC25A15/TIMM10B/TSPO/AIFM1/BCAP31/PNPT1/ZNF205/SLC25A2/NAIF1/BBC3/TIMM10/DMAC2L/UBL5/CHCHD4/E2F1/TIMM13/TIMM50/SLC25A10/FIS1/STOML2/SLC25A16/BAX/AIP/UBE2L3/TIMM29/OXA1L/SLC25A24/SAE1/SLC25A38/MCUR1/ATP5F1E/TP53/BLOC1S2/BNIP3/NOL3/TP63/MIPEP/SLC25A4/ATG13/COX18/TIMM17A/TMEM102/MUL1/CNP/PINK1/FZD5/DNAJC30/YWHAE/MPV17L/SREBF2/STARD3/SLC25A22/BAP1/ATP5F1D/ROMO1/TIMM44/SFXN4/MRPL18/HEBP2/MFN2/TOMM22/CHCHD10/GSK3A/ATP5PF/VPS11/BAK1/NMT1/ATP5ME/ATP5PO/SMDT1/ATP5PD/BCS1L/ATP5MC1/SLC8B1/RAC2/STPG1/MPC1/TOMM40/SLC25A20/CPT1A/SLC39A8/SREBF1/MIR29C/ATP5MF/HAX1/TIMM8B/SFXN2/SLC25A37/CPT2/SLC35F6/SLC25A1/BCL2L1/PSEN2/MID1IP1/TST/SFN/UCP2 |
| GOBP_REGULATION_OF_RNA_SPLICING | 0.462190905 | 1.662878618 | 0.000250467 | 0.010207479 | HNRNPU/CLK1/HSPA1A/RBM25/PTBP2/SRSF5/RBM39/HNRNPLL/ZNF326/MBNL2/RBFOX2/TIA1/HNRNPA2B1/HNRNPH1/DDX5/HNRNPH3/METTL4/FMR1/MBNL1/HNRNPA1/MBNL3/FAM172A/FXR1/YTHDC1/CLK4/RBM5/SETX/ERN1/DDX17/WTAP/SLC38A2/SRSF6/NCL/TRA2B/HNRNPK/SRSF10/RBM7/SON/SRSF7/NSRP1/HSPA8/CWC22/CELF2/KHDRBS1/RPS13/DYRK1A/REST/TRA2A/NUP98/RBM15/SNW1/RBMX/RBM12B/SMU1/NOVA1/CDK12/KHDRBS2/RRP1B/HABP4/JMJD6/SRPK2/SRSF4/PTBP3/LARP7 |
| GOBP_CENTRAL_NERVOUS_SYSTEM_NEURON_DIFFERENTIATION | 0.436407785 | 1.619327827 | 0.00025163 | 0.010207479 | SFRP2/INHBA/ROBO2/FGFR2/RORA/EPHA4/DCLK1/NR4A2/ROBO1/CTNNB1/ERBB4/RAPGEF2/MYCBP2/HSP90AA1/SFRP1/HERC1/ZSWIM6/BTG2/NFIB/SCYL2/ATP7A/LRP6/DYNC2H1/NDNF/DCC/CDH11/UBB/RYK/TOX/PAFAH1B1/HSP90AB1/PLXNA4/MPP5/SEMA3A/NIN/MDGA1/FAIM2 |
| GOBP_SISTER_CHROMATID_SEGREGATION | 0.427242217 | 1.59296413 | 0.000262737 | 0.010549582 | EML4/SMC4/ATRX/HNRNPU/CTNNB1/TOP2B/SMC5/FBXW7/CENPC/NIPBL/TTN/TNKS/GEN1/STAG2/PDS5A/CDC27/TPR/STAG1/KIF23/PCID2/SFPQ/ATM/RACGAP1/SLF1/PRC1/ESCO1/CENPF/PIBF1/DUSP1/KNTC1/CENPE/KIF18A/TENT4A/RIOK2/NDC80/ZNF207/WAPL/CHMP4C/RAD21/PDS5B/HDAC8/PTTG2/MRE11/TOP2A/BOD1/CUL3/NUSAP1/ANAPC1/SGO1/APC/SLF2/DLGAP5/KIF14/SMC3/LATS1/CHMP2B/SPAG5/POGZ/NCAPD3/DSCC1/ESCO2/DIS3L2/PHF13/CDK5RAP2/BUB3/NAA50/CDC16/ANAPC4/MAD2L1/NUMA1 |
| GOBP_RESPONSE_TO_FIBROBLAST_GROWTH_FACTOR | 0.466624069 | 1.693809582 | 0.000263159 | 0.010549582 | POSTN/SULF1/FGFR2/CCL5/FLRT2/THBS1/FGF7/KDM5B/FRS2/CCN2/CEP57/ZFP36/APLN/KIF16B/COL1A1/FAT4/ANOS1/SFRP1/RBFOX2/TIA1/NR4A1/HNRNPH1/SPRY1/HHIP/HNRNPA1/FGF1/CDC5L/CXCL13/NDNF/SETX/FLRT3/CD44/FGF2 |
| GOBP_SUPEROXIDE_METABOLIC_PROCESS | -0.532260976 | -1.838685107 | 0.00027531 | 0.010972145 | FBLN5/TGFB1/CYB5R4/CYBA/DHFR/CYBB/MIR27B/HVCN1/FPR2/SYK/GSTP1/PRKCD/SOD3/PRDX1/NCF1/BST1/NRROS/DUOX1/NCF2/CLEC7A/ITGB2/CD177/PREX1/NCF4/ITGAM/NQO1 |
| GOBP_STEROID_METABOLIC_PROCESS | -0.333501118 | -1.436160644 | 0.000277311 | 0.010987644 | HSD17B2/APOE/SRD5A1/DHRS9/SLC27A2/SC5D/LDLRAP1/SCAP/SRD5A2/CEBPA/FDFT1/PIP4P1/PIAS4/ELOVL6/NFE2L1/SREBF2/ACLY/SULT2B1/STARD3/HMGCR/ABCA1/LPCAT3/SNX17/CYP2R1/APOC1/SULT1A1/CYB5R1/MSMO1/MIR27B/PCSK9/HMGCS1/INSIG1/FAXDC2/DGAT2/ACOT8/SOAT1/LAMTOR1/HSD17B10/RXRA/HSD17B14/SREBF1/NSDHL/H6PD/SRD5A3/MVK/CYP27A1/VDR/CACNA1H/MVD/CLN6/EBPL/SCARB1/TM7SF2/ERG28/HINT2/SMPD1/GBA/AKR1C3/FDPS/ACAT2/FGFR4/G6PD/ADM/FASN/DHCR7/LSS/EBP/AGTR1/CYP1B1/AKR1C2/SULT1B1/SPP1 |
| GOBP_BODY_MORPHOGENESIS | 0.617967313 | 1.868551179 | 0.000288228 | 0.011354168 | CDON/ARID5B/TGFB3/PHLDB2/TIPARP/GPC3/NIPBL/COL1A1/PLEKHA1/CLASP2/LRP6/LEF1/RAB3GAP1/CRISPLD1 |
| GOBP_SERINE_FAMILY_AMINO_ACID_METABOLIC_PROCESS | -0.628500785 | -1.995078861 | 0.00029077 | 0.0113885 | DHFR2/SEPHS1/CTH/MPST/DHFR/GCSH/SEPHS2/GCLC/SHMT1/SARS1/GCLM/RIDA/SLC7A11/TST/PSAT1/SHMT2/CBS/PHGDH |
| GOBP_REGULATION_OF_EXOCYTOSIS | -0.393515857 | -1.619256288 | 0.000298033 | 0.011606244 | LGALS9/MICAL1/SPHK2/GIPC1/STXBP6/VPS4A/SNAPIN/RAB8A/SNF8/ADORA2A/PTAFR/SYK/UNC13D/LYN/PRAM1/VPS18/LAMP1/RAC2/CDK5/CALM3/P2RY1/IL4R/ATP13A2/GIT1/RAB15/ATP6AP1/CACNA1H/CD300A/RAB7A/NOTCH1/RAB3D/CEACAM1/STXBP2/CCR2/ITGB2/CD177/ADGRE2/FOXF1/GATA2/ITGAM/FGR/ADRA1A/HMOX1 |
| GOBP_ORGANELLE_FISSION | 0.356280903 | 1.443934166 | 0.000304299 | 0.011782939 | PDE3A/IGF1/EDN1/PLCB1/MYBL1/EML4/SMC4/PHIP/ATRX/LRRK2/HNRNPU/TOP2B/SMC5/CENPC/NIPBL/TTN/SPIRE1/HSPA1B/HSPA1A/DCN/EPS8/MCU/TNKS/CEP192/DDHD1/GEN1/CLASP2/STAG2/DNM1L/BCL2L11/USP16/RAD51B/PDS5A/CDC27/TPR/STAG1/CEP97/KIF23/SPICE1/EPGN/PCID2/PPARGC1A/INSR/PPP2R2A/ATM/TMEM135/CD28/UBB/CDK1/DNM3/TASOR/UBR2/RACGAP1/SLF1/MSH3/PRC1/CENPF/PIBF1/TPX2/MARCHF5/DUSP1/IGF2/ING2/MLH3/KNTC1/PPP2R2D/CENPE/OPA1/KIF20B/KIF18A/NEK9/TENT4A/LMNA/REEP3/KIF2A/RIOK2/DNM1/RAD50/NDC80/ZNF207/TOGARAM1/CHMP4C/CCNE2/SYCP2/FZR1/RAD21/PDS5B/PRKAR1A/MND1/LIF/RAD54B/PTTG2/MRE11/TOP2A/BOD1/GDAP1/CUL3/NUSAP1/CDK13/ANAPC1/SGO1/APC/MKI67/SLF2/DLGAP5/KIF14/VPS35/SMC3/WNT5A/CHMP2B/SPAG5/PPP2CA/POGZ/NCAPD3/IHO1/LSM14A/CDC14B/DSCC1/DIS3L2/TOM1L1/PHF13/CORO1C/BRIP1/PRKN/TMCC1/CDK5RAP2/BUB3/KIF11/CKS2/NAA50/CDC16/ANAPC4/MAD2L1/MTBP/NUMA1/FANCD2/BTC/AP3B1/HFM1/ANLN/TEX12/SLC25A46/INO80/KIF22/MAP9/SEC16B/CHEK2/CHEK1/ANKRD31/NCAPG/RAD51 |
| GOMF_PHOSPHORIC_DIESTER_HYDROLASE_ACTIVITY | 0.516549519 | 1.771369846 | 0.000307826 | 0.011852178 | ENPP2/PDE3A/PDE1A/PDE7B/PDE4D/PDE8B/PLCB1/PLCB4/CCL5/PDE8A/EDNRA/PDE5A/PDE10A/PDE2A/PLCE1/PDE3B/PDE4B/PLD1/PLCH1/ENPP3/F2RL2/PLCL1/PLCXD1 |
| GOBP_POSITIVE_REGULATION_OF_ANION_TRANSMEMBRANE_TRANSPORT | 0.567370746 | 1.817634408 | 0.000314272 | 0.011966883 | IGF1/ABCB1/CA2/ACE2/NR4A3/C3/GPC3/PRKCI/OSBPL8/BRAF/INSR/NFE2L2/ITGB1/CFTR/APPL1/ADIPOQ/AZIN1/ARPP19 |
| GOBP_JNK_CASCADE | 0.431246952 | 1.606108263 | 0.000314317 | 0.011966883 | SFRP2/CCL21/RASGRP1/PLCB1/FZD7/PDCD4/SPAG9/CCN2/CYLD/MAP3K13/DNAJA1/TRAF5/MAPK10/RIPK2/MAP4K4/MAPK8/SFRP1/RB1CC1/MAP4K5/TAOK1/TPD52L1/MECOM/ZMYND11/ERCC6/MAP3K20/SH3RF1/PJA2/MAP3K7/MTURN/PTPN22/ANKRD6/PER1/HIPK3/UBB/MAP3K2/CD40LG/GADD45A/ERN1/MAP3K5/HACD3/TRIB1/TRAF3/ARHGEF6/CCL19/NOD1/TAB3/MAPK9/STK3/ITCH/MAP4K3/SEMA3A/TNIK/DACT1/FCGR2B/HMGB1/MAP2K4/PTGER4/ZNF675/FKTN/TRAF6/TRAF1/DUSP10/TNFRSF19/SLAMF1/NCOR1/WNT5A |
| GOBP_GAS_TRANSPORT | 0.77367925 | 1.951883412 | 0.000316824 | 0.011995316 | HBB/HBA2/CA2/HBD/HBG2/IPCEF1 |
| GOBP_BONE_MORPHOGENESIS | 0.515160707 | 1.766607284 | 0.000322398 | 0.012138896 | SFRP2/HAS2/BMP6/SFRP4/FGFR2/FREM1/TGFB3/CSGALNACT1/RAB23/MMP16/COL1A1/NAB1/LRP6/MEF2C/LTBP3/IFT80/TRIP11 |
| GOBP_POSITIVE_REGULATION_OF_CHROMOSOME_ORGANIZATION | 0.447820996 | 1.644586814 | 0.000326327 | 0.012219337 | RPS6KA5/PRKCQ/ATRX/MAP3K4/LRRK2/CTNNB1/SMC5/MTF2/OGT/NIPBL/PRKD1/NBN/TNKS/RIF1/KMT2E/TNKS2/HNRNPA2B1/DHX36/NSD3/FMR1/PAXBP1/TPR/HNRNPA1/SFPQ/PPARGC1A/ATM/SIRT1/MPHOSPH8/TASOR/VEGFA/TCP1/SLF1/NAF1/KMT2A/ING2/TET1/KDM1A/RAD50/ATR/KAT7/BRD7/RAD21/LIF/PPP1R10/RTF1/AUTS2/HNRNPD/SART3/MRE11/JARID2/SIN3A/NEK7/CCT8/MYB/SLF2/CCT2/POT1/SMAD4/GNL3/DKC1/SNW1/ATF7IP/MIER1/WDR61 |
| GOMF_HISTONE_BINDING | 0.409894601 | 1.550440254 | 0.000332886 | 0.012396836 | ATAD2/PHIP/KDM5B/ATRX/MLLT3/SCML1/MTF2/TSPYL2/CHD1/MBTD1/TP53BP1/CHD2/SPIN1/SFMBT1/TNKS/STAT1/UHRF2/L3MBTL3/ATAD2B/KMT2E/ZMYND11/TBL1XR1/PHF14/RSF1/PSME4/USP16/PYGO1/LEF1/SMARCA5/SMARCC1/FMR1/MORC4/MSH6/PWP1/BPTF/SIRT1/TAF1/MPHOSPH8/MLLT10/KDM7A/KDM5A/SMARCA2/TRIM24/NAP1L4/TAF7/KMT2A/NAP1L5/L3MBTL4/DNAJC2/ING2/KAT6B/MYSM1/HAT1/NAP1L3/ANP32E/SFMBT2/SBNO1/NASP/KAT7/MORC3/BRD7/DEK/SART3/PHF6/PHC3/PHF10 |
| GOMF_MHC_CLASS_II_RECEPTOR_ACTIVITY | 0.877981559 | 1.893068924 | 0.000344819 | 0.012715845 | HLA-DQA1/HLA-DOA/HLA-DRA/HLA-DPA1/HLA-DQA2/HLA-DOB/HLA-DRB1/HLA-DRB3/HLA-DQB2/HLA-DQB1 |
| GOBP_INNERVATION | 0.73944812 | 1.94193445 | 0.000345184 | 0.012715845 | VCAM1/SULF1/SERPINE2/ITGA4/CHD7/SLITRK6/LRIG2/NRP1/SEMA3A |
| GOBP_REGULATION_OF_CELL_DEVELOPMENT | 0.353767731 | 1.43351458 | 0.000349736 | 0.012753057 | POSTN/HAS2/PDE3A/THY1/IGF1/KIT/SEMA3D/MME/CXCL12/SERPINE2/ROBO2/PLCB1/TRPC6/EPHA4/ETV5/RUFY3/GBP1/MACF1/SEMA3C/PTPRD/HIF1A/FBN1/ROBO1/FRZB/MAP3K13/LRRK2/CXCR4/CAPRIN2/CTNNB1/NF1/PCM1/FBXW7/CLDN18/BMPR1A/THRB/ASPA/SEMA6D/NR1D1/RAPGEF2/ARHGEF7/PRKCI/DOCK1/PER2/TNR/DNM1L/MAN2A1/DOCK7/TUG1/DHX36/SKIL/KANK1/CHD7/SEMA5A/PPARG/TMEM98/FGG/BCL2/GOLGA4/DCC/LRP4/EZH2/ROCK1/RFX3/LRP2/LIMS1/VEGFA/YAP1/DOCK5/RYK/PPP3CA/RELN/MAP1B/SPEN/PRKCH/FN1/FBXO31/TRIB1/FERMT2/PRTG/VCL/HOOK3/SLC9B2/NRP1/FBLN1/OPA1/PLXNA4/DICER1/KDM1A/UFL1/ROCK2/FZD3/BHLHB9/ADIPOQ/SEMA3A/BHLHE40/NIN/ADGRA2/CX3CL1/LIF/ADCYAP1/SPART/FBXW8/REST/APPL2/MDK/MIR222/MYB/PAK3/SMAD4/KIF14/RECK/DUSP10/SERPINF1/ABL1/IST1/MIR29B1/WNT5A/SNW1/DBN1/EIF4G2/IFNG/TIAM1/BCL11A |
| GOBP_REGULATION_OF_CAMP_MEDIATED_SIGNALING | 0.65029185 | 1.914139858 | 0.000349937 | 0.012753057 | PDE3A/PDE4D/APLNR/PDE2A/RAPGEF2/RGS2/PDE3B/SCTR/GNAI1/PRKAR2B/LPAR1/CRTC3/PRKAR1A/ADCYAP1 |
| GOBP_STRESS_ACTIVATED_PROTEIN_KINASE_SIGNALING_CASCADE | 0.396386515 | 1.522713043 | 0.000361974 | 0.013053647 | SFRP2/CCL21/RASGRP1/HGF/PLCB1/FZD7/PDCD4/SPAG9/CCN2/CYLD/MAP3K13/MAP3K4/ZFP36/DNAJA1/TRAF5/MAPK10/RIPK2/MAP4K4/MAPK8/SFRP1/MBIP/RB1CC1/MAP4K5/TAOK1/TPD52L1/MECOM/CUL1/ZMYND11/ERCC6/MAP3K20/SH3RF1/EIF2AK2/NFKB1/PJA2/MAP3K7/MTURN/PTPN22/ANKRD6/FBXW11/BTRC/SKP1/PER1/HIPK3/UBB/MAP3K2/CD40LG/GADD45A/VEGFA/ERN1/MAP3K5/HACD3/TRIB1/TRAF3/DLG1/TGFB2/DUSP1/ARHGEF6/CCL19/NOD1/FAS/TAB3/MAPK9/EZR/STK3/ITCH/MAP4K3/SEMA3A/TNIK/KAT7/DACT1/FCGR2B/HMGB1/MAP2K4/PTGER4/ZNF675/CCDC88C/MID1/FKTN/NBR1/TRAF6/PAK3/RELL1/TRAF1/DUSP10/TNFRSF19/SLAMF1 |
| GOCC_RIBOSOMAL_SUBUNIT | -0.387662354 | -1.565019245 | 0.000362016 | 0.013053647 | RPL19/RPL36AL/RPL7A/MRPL2/RPS9/EIF2A/RPS18/RPL26L1/NSUN4/RPL39/DAP3/RPL10/MRPL36/RPS15/MRPL38/MRPL10/MRPL27/RPS3/RPLP2/MRPL22/MRPS12/RPL37A/RPS27/MALSU1/MRPL13/MRPL48/RPL29/MRPS17/MRPL46/RPL12/RPL10A/MRPL20/MRPL45/MRPS28/MRPL30/MRPL17/RPL28/RPS19/MRPS21/MRPL12/RPS7/RPL36/MRPL14/RPLP0/RPL18A/MRPL16/MRPL40/RPLP1/MRPL24/MRPL9/RPS21/MRPS35/MRPL23/RPS10/MRPL35/MRPL49/NDUFAB1/MRPL21/RPL27/MRPS36/MRPS2/MRPS33/RPL13/RPS17/MRPL34/RPL35/MRPL4/MRPL18/MRPL41/RPSA/RPS4Y1/RPL23/MRPL58/MRPS18C/MRPL57/MRPL11/MPV17L2/MRPS11/MRPL33/MRPL53/MRPL28/MRPS15/MRPS16/MRPL37/MRPS18B/MRPS34/RPS5/RPS26 |
| GOBP_NEGATIVE_REGULATION_OF_CAMP_MEDIATED_SIGNALING | 0.813899663 | 1.921206122 | 0.000365045 | 0.013093572 | PDE3A/PDE4D/APLNR/PDE2A/RGS2/PDE3B/LPAR1/CRTC3 |
| GOBP_REGULATION_OF_INNATE_IMMUNE_RESPONSE | -0.336269388 | -1.445844025 | 0.000369233 | 0.013106479 | IFNGR1/PSMD4/NCR3/FADD/TIRAP/TKFC/PSMB2/HMGB2/ADAM8/SH2D1B/PSMD9/RPS19/PQBP1/PSME3/PSMA3/PSMB4/IRAK3/IFNGR2/CD1D/PSMB5/POLR3B/ZCCHC3/APOE/NR1H3/PSMC3/USP18/MUL1/GRN/NOD2/MUCL1/IKBKE/HLA-E/PSMC4/PSMD11/LGALS9/PSMB1/PSMD8/SERPINB4/PSMD3/VAV1/HRAS/PSMA7/NR1H2/RELA/FPR2/PRKACA/SYK/STING1/PSMB7/PRKCD/TRIM21/LYN/SOCS3/LAMP1/NMI/TTLL12/MUC16/MUC20/MAVS/PSMB3/ICAM3/FFAR2/TYRO3/RNF135/PYCARD/ICAM2/TLR4/ARG1/FCN1/PTPN6/CEACAM1/MUC21/TREM2/MNDA/IL18RAP/NLRC4/LILRB1/MUC4/SLAMF8/FCER1G/LILRA2/CLEC4E/MUC15/ARRB2/TLR8/MUC5B/HCK/VSIG4/FGR/CR1 |
| GOBP_LEUKOCYTE_MIGRATION | -0.317073247 | -1.396535803 | 0.000369251 | 0.013106479 | STK10/MERTK/LYN/GYPC/EMILIN1/CSF1R/SLC8B1/RAC2/ITGA5/CX3CR1/CCL2/MPP1/BST1/THBD/ITGAL/FFAR2/PDGFB/PIK3CD/P2RX4/SPNS2/SLC7A11/ESAM/GPR15/CD300A/PYCARD/MYD88/VEGFD/MYO1G/GRB2/SIRPA/FOXJ1/S100A14/MMP9/PTPN6/PADI2/CCL23/SELPLG/MMP14/DDT/RHOG/CEACAM1/GBA/CEACAM6/C5AR1/CMKLR1/PECAM1/TREM2/DOK2/SELL/CCR2/APOD/SLC3A2/ITGB2/S1PR1/CD177/EDNRB/ADGRE2/PREX1/SLAMF8/CORO1A/FCER1G/PLA2G7/SLC7A7/ITGAX/HCK/RIPOR2/C3AR1/ITGAM/DEFA1B/CCR1/CXCR2/HMOX1/CXCR1/CSF3R/S100A8/SAA1/S100A9/S100A12 |
| GOBP_POSITIVE_REGULATION_OF_REGULATED_SECRETORY_PATHWAY | -0.563473278 | -1.877951325 | 0.000373838 | 0.013200547 | SPHK2/PTAFR/SYK/UNC13D/LAMP1/CDK5/IL4R/RAB15/CACNA1H/RAB3D/ITGB2/CD177/GATA2/ITGAM/FGR |
| GOCC_RIBOSOME | -0.369727004 | -1.522263291 | 0.00037826 | 0.013287839 | MRPL22/GADD45GIP1/MRPS12/RPL37A/RPS27/MALSU1/PNPT1/MRPL13/MRPL48/RPL29/MRPS17/MRPL46/CHCHD1/RPL12/RPL10A/MRPL20/LARP1/MRPL45/MRPS28/MRPL30/MRPL17/MTG1/RPL28/RPS19/MRPS21/MRPL12/RPS7/NDUFA7/RPL36/MRPL14/RPLP0/MRPS23/RPL18A/MRPL16/MRPL40/RPLP1/MRPL24/MRPL9/RPS21/MRPS35/MRPL23/RPS10/MRPL35/MRPL49/NDUFAB1/APEX1/MRPL21/RPL27/MRPS36/MRPS2/MRPS25/MRPS33/RPL13/RPS17/MRPL34/RPL35/MTG2/MRPL4/MRPL18/MRPL41/RPSA/RPS4Y1/RPL23/MRPL58/MRPS18C/MRPL57/MRPL11/MPV17L2/AURKAIP1/MRPS11/MRPL33/MRPL53/MRPL28/MRPS15/MRPS16/MRPL37/MRPS18B/MRPS34/RPS5/RPL22L1/RPS26/APOD |
| GOBP_DNA_GEOMETRIC_CHANGE | 0.499737355 | 1.766371907 | 0.000390686 | 0.013653965 | SMARCA1/ATRX/WRN/CHD6/CHD1/CHD2/NBN/ZRANB3/ASCC3/ERCC6/HNRNPA2B1/HELB/DHX36/MNAT1/CHD7/CHD1L/SUPV3L1/UBB/SETX/MCM6/DHX9/ANXA1/DDX3X/MCM9/XPA/NAV2/RAD50/CHD9/HMGB1/RAD54B/MRE11/TOP2A/POT1/DNA2/RECQL/SMARCAD1/CUL4B/DSCC1/CUL4A/CHD3/BRIP1/XPC/HFM1/DDX1/CHD8/HELQ/CHD4/INO80 |
| GOBP_POST_EMBRYONIC_DEVELOPMENT | 0.537241986 | 1.782257279 | 0.000403374 | 0.013932233 | SLC18A2/FGFR2/ARID5B/NR4A2/SEMA3C/TIPARP/FBN1/KDM5B/ATRX/ITPR1/MYO1E/MFAP2/PLEKHA1/BCL2L11/ZFX/BCL2/SCN9A/ATM/VEGFA/AGO2/RC3H2/ASH1L/EFEMP1/SMAD2 |
| GOBP_MESENCHYME_DEVELOPMENT | 0.39109325 | 1.505466753 | 0.000406249 | 0.013932233 | SFRP2/HAS2/SEMA3D/EPHA3/EDN1/ROBO2/FGFR2/HGF/BMP5/PDCD4/ZFPM2/TGFB3/PHLDB2/BNC2/SEMA3C/MIR21/HIF1A/ROBO1/EDNRA/FRZB/CTNNB1/BMPR1A/COL1A1/SEMA6D/ERBB4/RANBP3L/SFRP1/STAT1/PKD2/CLASP2/CDH2/DDX5/LRP6/SMAD3/SNAI2/SPRY1/LEF1/MEF2C/SEMA5A/FAM172A/BCL2/EXOC4/ACTA2/EZH2/TASOR/YAP1/DDX17/FN1/FERMT2/LOXL2/TGFB2/FGFR1/PBLD/MDM4/NRP1/SMAD2/FGF10/LDLRAD4/FAM83D/EFNA1/ACVR1/SEMA3A/POGLUT1/DLG5/GREM1/ACTG2/MDK/MIR222/MEOX1/RBPJ/BAMBI/TAPT1/SMAD4/RDH10/NOLC1/NUP133/MIR29B1/WNT5A/PPP2CA/TIAM1/WT1/IL17RD |
| GOMF_GLUTATHIONE_PEROXIDASE_ACTIVITY | -0.71998945 | -1.995073851 | 0.000407287 | 0.013932233 | GSTP1/GSTA1/MGST3/MGST2/ALOX5AP/MGST1/GSTT1/GPX2/GPX3 |
| GOBP_DENDRITE_MORPHOGENESIS | 0.455677734 | 1.639445416 | 0.000407522 | 0.013932233 | TRPC6/EPHA4/PREX2/DCLK1/PTPRD/DOCK10/VLDLR/LRRK2/CAPRIN2/SIPA1L1/PPP1R9A/RAPGEF2/RBFOX2/DNM1L/DHX36/KIDINS220/PDLIM5/ZDHHC15/TANC2/LRP4/DNM3/SHANK2/PPP3CA/RELN/FBXO31/NLGN1/NRP1/OPA1/PICALM/EFNA1/BHLHB9/UBE3A/SEMA3A/FYN/TNIK/ABI2/FBXW8/PAK3/TMEM106B/SDC2/DBN1/TIAM1/DTNBP1/NEDD4/MAP2/DCDC2 |
| GOBP_ALCOHOL_CATABOLIC_PROCESS | -0.538104173 | -1.812635176 | 0.000409929 | 0.013932233 | TKFC/SULT1A2/TPI1/APOE/SORD/SNX17/SULT1A1/ADH7/INPP5K/ALDH3B1/SRD5A3/IMPA2/CYP27A1/INPP5A/SCARB1/AKR1C3/SULT1B1 |
| GOBP_ENDOCRINE_PROCESS | 0.528652327 | 1.760145709 | 0.000410914 | 0.013932233 | CPA3/INHBA/BMP6/MME/EDN1/ACE2/F2R/ENPEP/GJA1/APLN/C1QTNF3/HCAR2/CRY1/CORIN/C1QTNF1/CRHBP/PCSK5/TMF1/CTSG/CMA1/RAB8B |
| GOBP_LEUKOCYTE_CHEMOTAXIS | -0.378424544 | -1.559782182 | 0.00041633 | 0.013995549 | BSG/MIF/RAC1/XCL1/NCKAP1L/IL16/GPSM3/CKLF/CSF1/TMEM102/NOD2/PPIA/CREB3/VEGFB/CALR/RIN3/LGALS9/VEGFC/MMP28/TRPM2/CCL20/VAV1/C5AR2/FPR2/AIF1/SYK/LYN/CSF1R/SLC8B1/RAC2/CCL2/MPP1/BST1/FFAR2/PDGFB/PIK3CD/VEGFD/S100A14/PADI2/CCL23/DDT/C5AR1/CMKLR1/CCR2/ITGB2/S1PR1/EDNRB/ADGRE2/PREX1/SLAMF8/CORO1A/FCER1G/PLA2G7/RIPOR2/C3AR1/DEFA1B/CCR1/CXCR2/CXCR1/CSF3R/S100A8/SAA1/S100A9/S100A12 |
| GOCC_MHC_PROTEIN_COMPLEX | 0.735019571 | 1.930304219 | 0.000419595 | 0.013995549 | HLA-DQA1/HLA-DPB1/HLA-DOA/HLA-DRA/HLA-DPA1/HLA-DQA2/CD74/HLA-DOB/HLA-DRB1/HLA-DRB3/HLA-DQB2/HLA-DQB1/HLA-DMA |
| GOBP_REGULATION_OF_RESPONSE_TO_DNA_DAMAGE_STIMULUS | 0.423775544 | 1.598320101 | 0.000420911 | 0.013995549 | SFRP2/ANKRD1/CXCL12/EYA4/MIR21/PYHIN1/ARID2/WDR48/PLA2R1/UBR5/SMCHD1/SPIRE1/TP53BP1/USP47/RIF1/THOC1/CRY1/ERCC6/DDX5/MAP3K20/SNAI2/HELB/SKIL/FMR1/KMT5B/MCL1/MARCHF7/SMG1/BCL2/BCLAF1/FXR1/DYRK3/KLHL15/ATM/CD74/SIRT1/PPP4R2/SETD2/SETMAR/CD44/DHX9/SLF1/NSD2/RNF169/ING2/FGF10/TERF2IP/HNRNPK/KDM1A/ATR/FEM1B/KAT7/HMGB1/DEK/TPT1/PPP1R10/BRCC3/RADX/DYRK1A/SLF2/POT1/ABL1/CLU/TRIP12/RNF8 |
| GOBP_REGULATION_OF_LEUKOCYTE_TETHERING_OR_ROLLING | 0.840281972 | 1.943391577 | 0.000420996 | 0.013995549 | SELE/CCL21/CXCL12/SELP/ITGA4 |
| GOMF_PRIMARY_ACTIVE_TRANSMEMBRANE_TRANSPORTER_ACTIVITY | 0.560961276 | 1.797100968 | 0.000427406 | 0.014139666 | ABCG2/ABCC9/ABCB1/ABCA8/ABCA9/ABCA6/ABCA10/ATP7A/ABCG1/ABCC4/ABCD2/ABCA5/ABCD3/CFTR/ABCB7 |
| GOBP_CAMP_MEDIATED_SIGNALING | 0.562524867 | 1.798155287 | 0.000437875 | 0.014416031 | PDE3A/PDE7B/PDE4D/APLNR/AKAP6/PDE2A/ADGRG6/RAPGEF2/RGS2/PDE3B/SCTR/GNAI1/PRKAR2B/AHR/LPAR1/CRTC3/PDE7A/PRKAR1A/ADCYAP1/CAP2 |
| GOMF_DNA_DEPENDENT_ATPASE_ACTIVITY | 0.581883238 | 1.804759714 | 0.000442068 | 0.014484109 | SMARCA1/ATRX/BTAF1/TOP2B/HLTF/CHD6/MSH2/ERCC6/RAD51B/DHX36/SMARCA5/RFC1/MSH6/BPTF/MSH3/SMARCA2/TTF2/RAD54B/TOP2A |
| GOBP_REGULATION_OF_NEURON_PROJECTION_DEVELOPMENT | 0.362305364 | 1.457614131 | 0.000444805 | 0.014504055 | SFRP2/THY1/SEMA3D/ANKRD1/EPHA3/CXCL12/ROBO2/HGF/TRPC6/CNTN1/BMP5/EPHA4/PRRX1/DPYSL3/RUFY3/CHN1/MACF1/SEMA3C/PTPRD/MARK1/ROBO1/CD38/MAP3K13/VLDLR/LRRK2/SNAP25/CAPRIN2/CAMSAP2/ENC1/SEMA6D/SIPA1L1/PPP1R9A/MFSD2A/RAPGEF2/PRKD1/RGS2/SFRP1/PRKCI/VIM/CDH2/TNR/DNM1L/DHX36/SKIL/KIDINS220/ABL2/KANK1/SEMA5A/PDLIM5/CNR1/ZDHHC15/GOLGA4/NDNF/DCC/TANC2/NFE2L2/LRP4/LRIG2/KIF13B/EZH2/DENND5A/DNM3/SETX/LPAR1/ITM2C/ITGA6/VEGFA/RYK/PPP3CA/RELN/MAP1B/UST/FN1/FBXO31/SERPINI1/CAMK1D/NLGN1/TOX/TENM3/NRP1/OPA1/SCARB2/PLXNA4/KDM1A/EFNA1/TMEM30A/BHLHB9/ADCY6/UBE3A/SEMA3A/NIN/FYN/TNIK/INPP5F/CX3CL1/NCK1/ADCYAP1/COBL/SPART/ABI2/FBXW8/MDK/MIR222/ATP1B2/PAK3/MBOAT1/SERPINF1/CFLAR/LRRC4C/IST1/WNT5A/SDC2/DBN1/EIF4G2/EFHC2/TIAM1/BCL11A |
| GOMF_CALCIUM_DEPENDENT_PROTEIN_BINDING | -0.48547992 | -1.733896302 | 0.000448762 | 0.014563408 | VPS37C/ANXA3/NSMF/S100P/TNNC1/S100A6/CALM3/S100A16/SNTN/S100A14/S100A11/CD177/S100A3/S100A4/S100A8/S100A9/S100A12 |
| GOBP_NUCLEAR_CHROMOSOME_SEGREGATION | 0.395685643 | 1.515364146 | 0.000451665 | 0.014588138 | GEM/EML4/SMC4/ATRX/HNRNPU/CTNNB1/TOP2B/SMC5/FBXW7/CENPC/NIPBL/TTN/TNKS/GEN1/STAG2/PDS5A/CDC27/TPR/STAG1/KIF23/SPICE1/PCID2/SFPQ/ATM/RACGAP1/SLF1/PRC1/ESCO1/CENPF/PIBF1/DUSP1/MLH3/KNTC1/CENPE/KIF18A/TENT4A/FAM83D/RIOK2/NDC80/ZNF207/WAPL/CHMP4C/KNL1/CCNE2/SYCP2/RAD21/PDS5B/HDAC8/PTTG2/MRE11/TOP2A/BOD1/CUL3/NUSAP1/ANAPC1/SGO1/APC/SLF2/DLGAP5/KIF14/SMC3/LATS1/CHMP2B/SPAG5/POGZ/DYNC1H1/NCAPD3/IHO1/DSCC1/ESCO2/DIS3L2/PHF13/BRIP1/CDK5RAP2/BUB3/NAA50/CDC16/ANAPC4/MAD2L1/NUMA1/FANCD2/AXIN2/HFM1/TEX12/INO80/KIF22/ANKRD31/CENPQ/NCAPG |
| GOBP_CELL_SUBSTRATE_ADHESION | 0.3773707 | 1.494941968 | 0.000462893 | 0.014880273 | POSTN/VCAM1/CCDC80/HAS2/ANGPT2/THY1/ITGA2/CCL21/ECM2/EPHA3/ADAMTS9/ITGB3/FREM1/FZD7/ITGB6/ANTXR1/ITGBL1/CD96/PHLDB2/GBP1/MACF1/THBS1/COL3A1/CCN2/FER/UTRN/ITGA4/CCN1/CASK/ITGAV/LIMCH1/NF1/PTPRK/COL1A1/SERPINE1/MAP4K4/DLC1/ARHGEF7/SFRP1/DOCK1/CDK6/CLASP2/RASA1/FBLN2/BCL2L11/EDIL3/ADAMTS12/SMAD3/EGFL6/MKLN1/SNED1/KANK1/ATRNL1/NID2/FGG/BCL2/NDNF/SLK/ITGA8/ROCK1/ANGPT1/SGCE/LIMS1/EFEMP2/ITGA6/VEGFA/CD44/DOCK5/LAMB1/FN1/JAM3/FERMT2/VCL/SDC4 |
| GOBP_RESPONSE_TO_MUSCLE_ACTIVITY | 0.721606327 | 1.908284624 | 0.000465376 | 0.014889836 | POSTN/ITGA2/HIF1A/RYR2/PRKAA2/PPARGC1A/CAPN3/OPA1 |
| GOCC_PHAGOCYTIC_VESICLE_MEMBRANE | -0.496295372 | -1.757813547 | 0.000470444 | 0.014981662 | VAMP8/RAB32/RAB11B/RAB39A/HLA-E/RAB20/PIP4P1/CALR/CYBA/ANXA3/CYBB/RAB8A/PIP4P2/TCIRG1/RILP/ATP6V0D2/HVCN1/LAMP1/RAC2/ATP6V0B/TLR2/ATP6V0D1/RAB7A/MCOLN1/DNM2/SLC11A1/TLR6/TLR1/CORO1A |
| GOBP_OSSIFICATION | 0.364605036 | 1.454956906 | 0.00047968 | 0.01520475 | SFRP2/ASPN/IGF1/BMP6/FGFR2/HGF/BMP5/ATP2B1/ADGRV1/TGFB3/TXLNG/MIR21/BMP1/HIF1A/BMP3/PHEX/CSGALNACT1/CCN2/GPC3/CCN1/HNRNPU/DDR2/MMP16/NF1/MGP/BMPR1A/NIPBL/COL1A1/COL5A2/FAT4/LOX/TWSG1/IGFBP3/PRKD1/RANBP3L/SFRP1/CDK6/ACVR2A/COL1A2/SUCO/DDX5/NAB1/LRP6/SMAD3/SNAI2/DHX36/EIF2AK3/LEF1/MEF2C/LTBP3/PPARG/BCL2/MIR27A/CDH11/FHL2/LRP4/LIMD1/IFT80/YAP1/SLC24A3/DHX9/JUNB/FERMT2/CAT/TGFB2/CRIM1/CBFB/PBX1/IGF2/SPARC/SBDS/BMP2K/LGR4/TMEM64/MRC2/SGMS2/UFL1/ACVR1/SYNCRIP/CTHRC1/RUNX2/PTGER4/EXT2/DKK1/GREM1/SMAD5/NBR1/TRAF6/RDH14/DDX21/INTU/REST/MDK/PTK2/CHSY1/RBPJ/BAMBI/TAPT1/MMP2/ANKH/SLC8A1/MIR29B1/WNT5A/ANO6/RRAS2/SNRNP200/GLI3/CLEC3B/RBMX/SMAD1/PTPN11/OSR1/ZBTB40/TOB1/SP3/GLI2/EGFR/THBS3 |
| GOBP_INNATE_IMMUNE_RESPONSE_ACTIVATING_SIGNAL_TRANSDUCTION | -0.437633354 | -1.691470056 | 0.000482676 | 0.01522889 | PSMB2/PSMD9/PSME3/PSMA3/PSMB4/PSMB5/PSMC3/MUCL1/PSMC4/PSMD11/PSMB1/PSMD8/PSMD3/HRAS/PSMA7/RELA/PRKACA/SYK/PSMB7/PRKCD/LYN/MUC16/MUC20/PSMB3/ICAM3/FFAR2/ICAM2/FCN1/MUC21/MUC4/FCER1G/LILRA2/CLEC4E/MUC15/MUC5B/HCK |
| GOBP_REGULATION_OF_CELLULAR_RESPONSE_TO_GROWTH_FACTOR_STIMULUS | 0.392475139 | 1.513562034 | 0.000488048 | 0.015309056 | SFRP2/ASPN/LTBP1/SULF1/ITGB3/SFRP4/NREP/TGFB3/MIR21/HIF1A/THBS1/FBN1/ROBO1/GPC3/CCN1/CASK/SNX25/APLN/FST/HSPA1A/LOX/DCN/TWSG1/PMEPA1/SFRP1/GRB10/SMURF2/ELAPOR2/ZNF451/ADAMTS12/SMAD3/FSTL1/ADAMTS3/SKIL/SPRY1/NEO1/HHIP/MYOCD/CTDSPL2/SFRP5/HTRA1/PPARG/FGF1/CXCL13/ATP2B4/LTBP4/UBB/SIRT1/SMURF1/ZDHHC17/LRP2/FGF2/LEMD3/CRIM1/FGFR1/ING2/TRIM33/PBLD/SMAD2/FGF10/HSP90AB1/LDLRAD4/MPP5 |
| GOBP_REGULATION_OF_G_PROTEIN_COUPLED_RECEPTOR_SIGNALING_PATHWAY | 0.453222493 | 1.630611906 | 0.00048971 | 0.015309056 | RGS1/RGS5/EDN1/PLCB1/RGS13/CCL5/C3/BICD1/ARRDC3/APLN/APLNR/RGS6/SLC39A14/TMOD2/PLCE1/RGS4/GPRASP1/RGS2/NMT2/PDE4B/RGS16/ZDHHC3/MET/CRY1 |
| GOBP_AMINO_SUGAR_CATABOLIC_PROCESS | -0.834082401 | -2.02634329 | 0.000498766 | 0.015474633 | CHIA/NAGK/MGAT1/RENBP/GNPDA1/NPL/CHI3L2/CHIT1 |
| GOMF_ATPASE_COUPLED_INTRAMEMBRANE_LIPID_TRANSPORTER_ACTIVITY | 0.707370108 | 1.88158594 | 0.000501682 | 0.015474633 | ABCB1/ATP11A/ATP11C/MFSD2A/ATP11B/ATP8B1/ATP10D/ABCG1/ATP10A/ATP9B/TMEM30A/ATP9A/ATP8A1 |
| GOBP_PHAGOSOME_ACIDIFICATION | -0.666729635 | -1.938252691 | 0.000501819 | 0.015474633 | ATP6V1E1/ATP6V1G1/ATP6V0E1/ATP6V0C/ATP6V0E2/ATP6V1C2/ATP6V1A/ATP6V1B2/RAB39A/RAB20/TCIRG1/ATP6V0D2/ATP6V0B/ATP6V0D1/RAB7A/ATP6V1F/SLAMF8 |
| GOBP_FAT_CELL_DIFFERENTIATION | 0.411265246 | 1.549175779 | 0.000527586 | 0.016195935 | SFRP2/WIF1/FABP4/PTGS2/MEDAG/PLCB1/RORA/ARID5B/ZFPM2/STEAP4/NR4A3/NR4A2/MIR21/LPL/FRZB/HNRNPU/ZFP36/C1QTNF3/NIPBL/NR1D1/TRIO/RGS2/SFRP1/RARRES2/PER2/FABP3/OSBPL8/NR4A1/SMAD3/BBS2/NOC3L/ANKRD26/PPARG/BBS7/PPARGC1A/MEX3C/MIR27A/SIRT1/YAP1/CCDC3/FERMT2/SYAP1/ATF2/OSBPL11/FGF10/HTR2A/CCND1/STK3/EBF2/TMEM64/NAPEPLD/ZFP36L2/ADIPOQ/ERAP1/BBS9 |
| GOBP_SKELETAL_SYSTEM_MORPHOGENESIS | 0.41183551 | 1.546493476 | 0.000547777 | 0.016740351 | SFRP2/HAS2/BMP6/SFRP4/FGFR2/FREM1/ARID5B/PRRX1/TGFB3/BMP1/CSGALNACT1/TIPARP/RAB23/CCN2/ZFAND5/MTHFD1L/SOX5/WDR48/CTNNB1/MMP16/MGP/NIPBL/COL1A1/SFRP1/PLEKHA1/NAB1/LRP6/SMAD3/HHIP/MEF2C/LTBP3/SETD2/IFT80/TRIP11/RYK/RAB33B/DLG1/FGFR1/WDR19/SMAD2 |
| GOBP_ORGANIC_HYDROXY_COMPOUND_BIOSYNTHETIC_PROCESS | -0.357071554 | -1.49671802 | 0.000550306 | 0.016742504 | MFSD12/APOE/IP6K3/SLC27A2/SC5D/SCAP/SRD5A2/FDFT1/ELOVL6/SREBF2/SPHK2/ACLY/HMGCR/GIPC1/LPCAT3/PNPO/SPR/CYP2R1/DHFR/CD244/CYB5R1/MSMO1/PTAFR/HMGCS1/INSIG1/FAXDC2/ACOT8/HSD17B10/GPER1/SREBF1/NSDHL/P2RY1/H6PD/AGTR2/SRD5A3/PCK2/PTH1R/IMPA2/MVK/CYP27A1/SLC7A11/CACNA1H/PNMT/PGP/MVD/DHDDS/PCBD1/TM7SF2/ERG28/DDT/GBA/AKR1C3/CTNS/FDPS/ACAT2/FGFR4/G6PD/FASN/DHCR7/LSS/EBP/PSAT1 |
| GOMF_HEME_COPPER_TERMINAL_OXIDASE_ACTIVITY | -0.718288859 | -2.046355268 | 0.000552761 | 0.016742504 | COX10/COX8A/COX4I1/NDUFA4/C15orf48/NDUFA4L2/COX7A2L/SURF1/CYB5A/COX7A2/COX7A1/COX4I2/COX5B/COX5A/COX7B/COX6B1 |
| GOBP_ANTIGEN_PROCESSING_AND_PRESENTATION_OF_EXOGENOUS_PEPTIDE_ANTIGEN_VIA_MHC_CLASS_I | -0.481300965 | -1.718971123 | 0.000557379 | 0.016807681 | PSMD4/FCGR1A/PSMB2/PSMD9/PSME3/PSMA3/PSMB4/PSMB5/VAMP8/PSMC3/HLA-E/PSMC4/CALR/PSMD11/CYBA/PSMB1/PSMD8/PSMD3/PSMA7/CYBB/PSMB7/MFSD6/IFI30/NCF1/PSMB3/NCF2/FCER1G/NCF4 |
| GOMF_G_PROTEIN_COUPLED_CHEMOATTRACTANT_RECEPTOR_ACTIVITY | -0.681065935 | -1.972716752 | 0.000565188 | 0.016968091 | CX3CR1/ACKR4/CMKLR1/CCRL2/CCR2/CCR1/CXCR2/CXCR1 |
| GOBP_CELLULAR_AMINO_ACID_METABOLIC_PROCESS | -0.331799283 | -1.432382298 | 0.000575549 | 0.017203363 | PSMD4/ASPG/ASS1/PYCR1/MTHFR/PFAS/PSMB2/EEF1E1/PSMD9/DHFR2/EPRS1/SEPHS1/PSME3/GCDH/GLUL/CAD/ADI1/PSMA3/SARDH/PSMB4/THAP4/CTH/PSMB5/VARS2/ACAD8/PYCR2/UPB1/HAL/BCAT1/PSMC3/MPST/CRYM/ADHFE1/FPGS/PSMC4/PSMD11/TARS2/PSMB1/PSMD8/SARS2/PEMT/ALDH4A1/PSMD3/DHFR/ABAT/GCSH/PSMA7/SEPHS2/BCKDK/FARSA/GSS/GCLC/ALDH6A1/VARS1/PSMB7/ACY1/AIMP2/HMGCL/MARS1/HSD17B10/ASRGL1/YARS1/SHMT1/SLC39A8/AARS1/SARS1/OGDH/RIMKLA/GCLM/PSMB3/RIDA/SLC7A11/PEPD/PLOD3/ARG1/PCBD1/AHCY/PRODH/CLN3/FAH/AFMID/CTNS/BCAT2/TST/PSAT1/SLC7A7/SHMT2/CBS/PHGDH/NQO1 |
| GOBP_INTERLEUKIN_6_PRODUCTION | -0.411262634 | -1.634384465 | 0.000601178 | 0.017890946 | AGER/POU2F2/GHRL/IRAK3/TNF/NCKAP1L/IL16/CSK/NOD2/MBP/LGALS9/CYBA/SPHK2/KLF2/PTAFR/C5AR2/AIF1/SYK/TICAM1/CEBPB/ARRB1/TLR2/MAVS/MAPK13/SIGLEC16/ORM1/PYCARD/MYD88/TLR4/SIRPA/FOXJ1/PTPN6/HYAL2/NLRP12/TMEM106A/TLR6/GBA/TREM2/TLR1/CLEC7A/SCIMP/LILRB4/IL17RA/LILRA5/MMP8/LILRA2/ARRB2/TLR8/LILRB2 |
| GOMF_WNT_PROTEIN_BINDING | 0.670662725 | 1.877240055 | 0.000614523 | 0.018208582 | SFRP2/WIF1/SFRP4/FZD7/FRZB/SFRP1/LRP6/SFRP5/RYK/FZD6/WLS/FZD3/CTHRC1 |
| GOBP_TRICARBOXYLIC_ACID_CYCLE | -0.626606728 | -1.925009872 | 0.000632962 | 0.018673744 | ME2/PDHB/MDH1/MDH1B/IDH3B/ACO2/SDHC/SDHAF2/SDHD/SDHB/IDH3G/FAHD1/DLAT/OGDH/IDH2/DHTKD1/MDH2/IDH1 |
| GOBP_REGULATION_OF_MRNA_SPLICING_VIA_SPLICEOSOME | 0.500627198 | 1.744603941 | 0.000663295 | 0.019476127 | HNRNPU/RBM25/RBM39/MBNL2/RBFOX2/TIA1/HNRNPA2B1/DDX5/FMR1/MBNL1/HNRNPA1/MBNL3/FAM172A/FXR1/YTHDC1/RBM5/DDX17/WTAP/SRSF6/NCL/TRA2B/HNRNPK/SRSF10/RBM7/SON/SRSF7/NSRP1/HSPA8/CWC22/CELF2/KHDRBS1/DYRK1A/REST/TRA2A/NUP98/RBM15/SNW1/RBMX/SMU1/NOVA1/KHDRBS2/JMJD6/SRPK2/SRSF4/LARP7 |
| GOBP_TOLL_LIKE_RECEPTOR_4_SIGNALING_PATHWAY | -0.593032198 | -1.895273692 | 0.000665875 | 0.019476127 | LY96/LYN/NMI/PRKCE/TLR4/S100A14/PIK3AP1/SCIMP/ITGB2/LTF/LILRA2/ITGAM/CD14/BPIFB1 |
| GOBP_CELL_CHEMOTAXIS | -0.336932132 | -1.435713025 | 0.000691249 | 0.02005523 | C5AR2/FPR2/AIF1/SYK/CORO1B/GSTP1/PRKCD/LYN/CSF1R/SLC8B1/RAC2/CX3CR1/CCL2/MPP1/BST1/FFAR2/PDGFB/PIK3CD/P2RX4/NOTCH1/MIR15A/VEGFD/ACKR4/S100A14/PADI2/CCL23/DDT/RHOG/C5AR1/CMKLR1/CCRL2/CCR2/ITGB2/S1PR1/EDNRB/ADGRE2/PREX1/SLAMF8/CORO1A/FCER1G/AGTR1/PLA2G7/ARRB2/RIPOR2/C3AR1/DEFA1B/CCR1/CXCR2/CXCR1/CSF3R/S100A8/SAA1/S100A9/S100A12 |
| GOBP_POSITIVE_REGULATION_OF_DEVELOPMENTAL_GROWTH | 0.428364843 | 1.580634559 | 0.00069156 | 0.02005523 | IGF1/EDN1/CXCL12/FGFR2/PLCB1/ZFPM2/RUFY3/MACF1/MAP3K13/CXCR4/BMPR1A/AKAP6/NIPBL/ERBB4/GPR21/MEF2C/BBS2/CHD7/SEMA5A/RPS6KB1/BCL2/GOLGA4/ITSN2/INSR/SMURF1/VEGFA/CAPN3/YAP1/FGF2/MAP1B/IL7/FN1/IGF2/NRP1/ACACB/EZR |
| GOBP_NEPHRIC_DUCT_DEVELOPMENT | 0.826307229 | 1.911071001 | 0.000700115 | 0.02021731 | EPHA4/AHI1/GPC3/PKD2 |
| GOCC_BLOOD_MICROPARTICLE | 0.475257604 | 1.674245844 | 0.000703139 | 0.020218943 | HBB/HBA2/CFH/BCHE/HBD/IGKC/C4BPA/IGHG1/IGHM/CD5L/HBG2/C3/JCHAIN/IGHA1/CFHR1/HSPA1B/HSPA1A |
| GOBP_CELLULAR_MODIFIED_AMINO_ACID_METABOLIC_PROCESS | -0.377124642 | -1.539500949 | 0.000722124 | 0.020651752 | PLAAT3/GGT5/FOXE1/ASS1/MTHFR/CNDP2/CHDH/ABHD12/DHFR2/SARDH/GSTO1/PLA2G15/SERINC2/CRAT/OPLAH/SLC19A1/MMACHC/PLA2G10/CRYM/GGT3P/FPGS/CLIC1/HAGH/CKMT1A/CKMT1B/SLC46A1/PEMT/LPCAT3/ALDH4A1/DIO3/DHFR/DUOXA1/GSS/GCLC/SLC5A6/ALDH1L1/GGTLC2/GSTP1/SLC22A4/GSTA1/CPT1A/SHMT1/GSTM4/PLA1A/GSTA2/GCLM/MGST2/SLC7A11/CPT2/PLOD3/GSR/AHCY/IDH1/GSTT2/DUOX1/PRODH/VNN1/GSTT1/GGTLC1/CTNS/GSTA4/G6PD/SHMT2/SULT1B1/VNN2 |
| GOBP_PHAGOSOME_MATURATION | -0.564355285 | -1.850008161 | 0.000724971 | 0.020651752 | ATP6V1E1/ATP6V1G1/ATP6V0E1/SYT7/ATP6V0C/ATP6V0E2/TMEM175/ATP6V1C2/ATP6V1A/RAB32/ATP6V1B2/RAB39A/RAB20/TCIRG1/ATP6V0D2/SRPX/ATP6V0B/ATP6V0D1/RAB7A/MCOLN1/ATP6V1F/CLN3/SLAMF8/CORO1A |
| GOBP_NUCLEOSIDE_TRIPHOSPHATE_BIOSYNTHETIC_PROCESS | -0.481332908 | -1.723246148 | 0.000727281 | 0.020651752 | ATP5F1A/NME6/UQCC3/NUDT2/IMPDH2/DMAC2L/ATP6V0C/AK8/AK7/STOML2/NME5/ATP6V1A/ATP5F1EP2/CAD/ATP5F1E/TGFB1/PINK1/DNAJC30/SPHK2/UCK1/ALDOA/ATP5F1D/DTYMK/ATP5PF/ATP5ME/ATP5PO/ATP5PD/CMPK2/DGUOK/ATP5MC1/ATP5MF/IMPDH1/NME1/ENO1/PKM/NME2/TREM2 |
| GOBP_RHYTHMIC_PROCESS | 0.380841493 | 1.470607835 | 0.000749068 | 0.021182147 | HAS2/INHBA/EGR1/NR1D2/ROBO2/RORA/MMP7/TGFB3/KDM5B/HLF/TOP1/HNRNPU/NRIP1/PER3/FBXW7/OGT/MAPK10/SERPINE1/PTGDS/NR1D1/MAPK8/MYCBP2/ID4/PLEKHA1/PER2/SLIT3/CRY1/DNM1L/CREM/DDX5/HNRNPR/FBXW11/PPARG/PRKAA2/BTRC/SFPQ/PPARGC1A/PER1/ARNTL2/EZH2/AHR/SIRT1/CDK1/BTBD9/SETX/TARDBP/JUN/DHX9/KDM5A/PAM/USP7/NFIL3/KMT2A/TGFB2/NLGN1/CIART/USP9X/MAPK9/LGR4/KLF9/METTL3/ROCK2/UBE3A/ETS1/ADIPOQ/BHLHE40/HNF1B/NFYA/EGR3/FBXL3/HNRNPD/CRY2/TOP2A/SIN3A/DYRK1A/MDK/SUV39H2/NCOA2/CASP3/MMP19/GPR176/SERPINF1/NCOA1/ZNF830/NCOR1 |
| GOCC_INTRINSIC_COMPONENT_OF_ORGANELLE_MEMBRANE | -0.312553423 | -1.367833728 | 0.000764327 | 0.021462273 | SLC15A4/MMGT1/DNAJB2/GRAMD2A/LBR/TBL2/UBIAD1/TRAM2/EMC7/WFS1/PCSK7/CHST5/FIS1/FKBP8/SLC35B2/TMEM33/SLC66A1/PEX16/A4GALT/MAN1C1/VAMP5/P2RX1/EMC1/CCDC51/ABCD1/SCD/GRAMD1A/RAB35/DPM2/SEC61A1/OXA1L/TMEM43/MCUR1/RTN2/PEX12/BNIP3/HM13/FURIN/DNAJC11/SLC25A4/RAB11B/DHRS9/BET1L/SLC27A2/COX18/TIMM17A/RTN1/MUL1/GOLGA7/ADCK1/HLA-E/DOLK/PINK1/FICD/CHST2/BSCL2/CALR/DERL2/DPM3/ELOVL6/EMC8/SREBF2/GPAA1/ELOVL1/GHITM/SYNE4/SLC25A19/DCSTAMP/MTX1/LFNG/SFXN4/TMEM70/MFN2/PREB/ATF6B/FITM2/RAB5B/CHCHD10/APOO/QSOX1/BAK1/ECE1/YIF1A/DOLPP1/STING1/SYVN1/YIF1B/SMDT1/EMC4/INSIG1/DGAT2/DNAJC5/DMPK/DPAGT1/LAMP1/PIGS/SLC8B1/TEX261/SLC37A4/MPC1/TOMM40/CPT1A/EMC6/ATP13A2/ERGIC1/P2RX4/SFXN2/TECR/SLC35B1/SLC39A13/TM7SF2/PTPRS/STX10/ABCB6/CLN3/SLC35A4/SLC37A2/DHCR7/MFNG/SUN2/SLC6A9 |
| GOBP_REGULATION_OF_TELOMERE_MAINTENANCE | 0.51497564 | 1.714609237 | 0.000765273 | 0.021462273 | PRKCQ/ATRX/MAP3K4/HNRNPU/XRN1/NBN/TNKS/TNKS2/HNRNPA2B1/DHX36/TENT4B/SMG1/HNRNPA1/ATM/TCP1/USP7/NAF1/TERF2IP/RAD50/TERF1/ATR/YLPM1/PPP1R10/HDAC8/HNRNPD/MRE11/NEK7/CCT8/CCT2/POT1/GNL3/DKC1 |
| GOBP_ALTERNATIVE_MRNA_SPLICING_VIA_SPLICEOSOME | 0.548067313 | 1.79131256 | 0.000770366 | 0.021516571 | HNRNPU/RBM25/MBNL2/RBFOX2/RSRC1/DDX5/FMR1/MBNL1/HNRNPA1/MBNL3/FAM172A/SFPQ/FXR1/YTHDC1/RBM5/DHX9/DDX17/WTAP/SRSF6/TRA2B/SRSF1/KDM1A/RBM7/NSRP1/CELF2/KHDRBS1/DYRK1A/CDK13/REST/RBM15/RBMX/SLU7/SMU1/NOVA1/KHDRBS2 |
| GOCC_EXTERNAL_SIDE_OF_PLASMA_MEMBRANE | 0.371025831 | 1.448219617 | 0.000785287 | 0.021843803 | VCAM1/THY1/ITGA2/CD69/KIT/ABCG2/ABCB1/CXCL9/NT5E/CXCL12/SERPINE2/SELP/IL13RA2/ITGB6/ANTXR1/GFRA1/CD163L1/IGKC/IGHG1/THBS1/IGHM/PDCD1LG2/CUBN/CTLA4/ENPEP/ADGRA3/CXCR4/ITGAV/SCARA5/IGHA1/BMPR1A/CXCL10/CCR4/KLRC4-KLRK1/CD1C/CLEC2D/BTN3A1/IL12RB2/NRCAM/MCAM/TFRC/CD226/ABCG1/MSR1/CD248/ENPP3/CD2/FGG/CD200R1/CD83/BTN3A3/INSR/CD80/CD74/IL2RA/CD28/ABCC4/CD3G/HLA-DRB1/TNFRSF9/LRP2/CD40LG/LIFR/MAP3K5/ANXA1 |
| GOCC_SPLICEOSOMAL_TRI_SNRNP_COMPLEX | -0.604887545 | -1.920122271 | 0.000822309 | 0.022780636 | USP39/SNRPF/ZMAT2/SNRPE/PRPF31/EFTUD2/SNRPD1/RNU5D-1/PPIH/LSM7/SNRPB/SART1/SNU13/RNU5B-1/LSM2/TXNL4A/RNU5A-1/LSM6/LSM5/SNRPD2/LSM3/LSM4/RNU5E-1/RNU4-1/RNU4-2 |
| GOMF_TUBULIN_BINDING | 0.363417883 | 1.444120901 | 0.000860649 | 0.023670591 | AGBL1/CEP70/HSPH1/MACF1/EML4/SYBU/SGIP1/NAV3/LRRK2/DST/CAMSAP2/CEP57/GJA1/MTUS1/OFD1/KIF16B/KIF3A/MAP4K4/CCSER2/CEP295/RGS2/KIF13A/PDE4B/STRBP/TAOK1/MAP7D3/CLASP2/DNM1L/BCL2L11/SYT11/FMR1/HOOK1/TPR/HDGFL3/FBXW11/KIF23/IFT74/DIXDC1/PPARGC1A/MACO1/ALMS1/KIF21A/KIF13B/DNM3/POLB/SETD2/EFHC1/JAKMIP2/RACGAP1/CNN3/MAP1B/CEP44/PRC1/KIF27/CENPF/TPX2/HOOK3/DDX3X/TPPP3/KIF15/CCDC66/VPS41/KIF5B/KIF20A/IRAG2/IFT81/CENPE/RABGAP1/SBDS/PAFAH1B1/OPA1/KIF20B/KIF5C/KIF18A/CENPJ/EZR/UNC5C/REEP3/FAM83D/KIF2A/APPL1/DNM1/MID2/CEP350/BEX4/TERF1/ZNF207/TOGARAM1/NIN/FYN/HAUS6/CAMSAP1/CCDC88C/VASH2/MID1/PHF6/NUSAP1/GABARAPL1/APC/RP1/MAP1LC3B/DLGAP5/KIF14/TTLL7/KRIT1/SMC3/TBCEL/TTBK2/EFHC2/SPAG5/TIAM1/KATNAL1/NDN/BBS4/KATNA1/MAP2/PRNP/GABARAPL2/TRIM36/PRKN/CDK5RAP2/BCAS3/KIF11/MAP1LC3B2/CEP57L1/NUMA1/FNTA/VAPA/CKAP5 |
| GOMF_DNA_HELICASE_ACTIVITY | 0.526200761 | 1.737026055 | 0.00086138 | 0.023670591 | ATRX/WRN/SMC6/CHD6/CHD1/CHD2/ASCC3/HELB/DHX36/CHD7/CHD1L/SUPV3L1/SETX/MCM6/DHX9/ANXA1/DDX3X/MCM9/NAV2/RAD50/CHD9/RAD54B/MRE11/DNA2/RECQL/SMARCAD1/DSCC1/CHD3/BRIP1/HFM1/CHD8/HELQ/CHD4/INO80/RAD51/ERCC3 |
| GOBP_TRANSFERRIN_TRANSPORT | -0.61413246 | -1.916775165 | 0.000872049 | 0.023798923 | ATP6V1E1/ATP6V1G1/ATP6V0E1/ATP6V0C/ATP6V0E2/ATP6V1C2/ATP6V1A/ARHGAP1/RAB11B/ATP6V1B2/TCIRG1/ATP6V0D2/ATP6V0B/ATP6V0D1/ATP6AP1/MCOLN1/ATP6V1F/DNM2/STEAP3 |
| GOBP_GLIAL_CELL_DEVELOPMENT | -0.427759057 | -1.643384775 | 0.000873345 | 0.023798923 | ADORA2A/SIRT2/PICK1/DAG1/FPR2/EIF2B2/GSTP1/NCSTN/LYN/CDK5/TLR2/TLR4/NRROS/LAMC3/LGI4/PRX/C5AR1/TREM2/PHGDH/S100A8/S100A9 |
| GOCC_MYOSIN_II_COMPLEX | 0.74067573 | 1.870019232 | 0.000881549 | 0.023798923 | MYH10/LIMCH1/MYH11 |
| GOBP_NEUTROPHIL_MIGRATION | -0.437762095 | -1.691318992 | 0.000882239 | 0.023798923 | RAC2/CCL2/MPP1/BST1/PIK3CD/MYD88/CCL23/RHOG/C5AR1/PECAM1/ITGB2/CD177/PREX1/SLAMF8/FCER1G/RIPOR2/C3AR1/CXCR2/CXCR1/CSF3R/S100A8/SAA1/S100A9/S100A12 |
| GOBP_RIBOSE_PHOSPHATE_BIOSYNTHETIC_PROCESS | -0.377510435 | -1.540207338 | 0.00088654 | 0.023798923 | ADCY2/ATP5F1A/HSD17B12/PDP2/NME6/ACSBG1/UQCC3/NUDT2/PPT1/PRPSAP1/GUCY1B1/PPCS/APRT/IMPDH2/GSTZ1/DMAC2L/PANK4/ATP6V0C/PDHB/ADCY5/NPR1/ACSF3/MLYCD/SLC35B2/PFAS/PDK2/ADCY1/SCD/STOML2/NME5/GCDH/ATP6V1A/ATP5F1EP2/CAD/ATP5F1E/DHODH/TGFB1/PINK1/PAICS/DNAJC30/ELOVL6/SPHK2/ACLY/ELOVL1/UCK1/UPP1/ALDOA/ATP5F1D/ACSL1/PAPSS2/ATP5PF/ADCY9/ATP5ME/ATP5PO/ATP5PD/DLAT/DGUOK/COASY/ATP5MC1/ADCY4/ATP5MF/IMPDH1/NME1/ENO1/TECR/SLC25A1/AMPD3/TKT/PKM/NME2/PYGL/TREM2/G6PD/FASN/ACSS2 |
| GOBP_REGULATION_OF_ANION_TRANSMEMBRANE_TRANSPORT | 0.477165713 | 1.683310969 | 0.000887003 | 0.023798923 | IGF1/ABCB1/EDN1/CA2/ACE2/NR4A3/SLC25A27/THBS1/C3/EDNRA/GPC3/GOPC/RGS4/RGS2/PRKCI/GRB10/PER2/OSBPL8/RPS6KB1/BRAF/INSR/NFE2L2/SELENOS/PLA2G1B/ITGB1/CFTR/APPL1/ADIPOQ/AZIN1/ARPP19 |
| GOMF_INTRAMEMBRANE_LIPID_TRANSPORTER_ACTIVITY | 0.652610279 | 1.845613086 | 0.000903546 | 0.024147722 | ABCB1/ATP11A/ATP11C/PLSCR4/MFSD2A/ATP11B/ATP8B1/ATP10D/ABCG1/ATP10A/ATP9B/TMEM30A/ATP9A/ATP8A1 |
| GOBP_REGULATION_OF_LEUKOCYTE_MEDIATED_IMMUNITY | -0.369237461 | -1.520142262 | 0.000922805 | 0.024566083 | IL18R1/TNF/CD1D/XCL1/VAMP8/TGFB1/TNFSF13/FES/NOD2/HLA-E/FZD5/LGALS9/SPHK2/SERPINB4/MAD2L2/VAV1/WAS/C4BPB/PTAFR/PARP3/SYK/UNC13D/BCL6/TNFRSF1B/LYN/JAK3/PRAM1/LAMP1/RAC2/TICAM1/SASH3/IL4R/MAVS/CD300A/AZGP1/TLR4/ARG1/FOXJ1/PTPN6/CEACAM1/STXBP2/IL18RAP/CLEC7A/CCR2/SCIMP/ITGB2/LILRB4/CD177/LILRB1/ADGRE2/FOXF1/ARRB2/GATA2/ITGAM/FGR/CR1/HMOX1 |
| GOBP_REGULATION_OF_SUPEROXIDE_ANION_GENERATION | -0.732156539 | -1.981514824 | 0.000933726 | 0.024760087 | TGFB1/HVCN1/FPR2/SYK/GSTP1/PRKCD/CLEC7A/ITGB2/CD177/ITGAM |
| GOBP_GLUTATHIONE_METABOLIC_PROCESS | -0.508681635 | -1.733966118 | 0.00096002 | 0.025358675 | GSTA3/GGT6/GSTZ1/GGT5/CNDP2/GSTO1/OPLAH/MMACHC/GGT3P/CLIC1/HAGH/GSS/GCLC/GGTLC2/GSTP1/GSTA1/GSTM4/GSTA2/GCLM/MGST2/SLC7A11/GSR/IDH1/GSTT2/GSTT1/GGTLC1/CTNS/GSTA4/G6PD |
| GOBP_MITOCHONDRIAL_TRANSMEMBRANE_TRANSPORT | -0.442714288 | -1.676283787 | 0.000974732 | 0.02564787 | ATP5F1A/SLC25A5/SFXN3/SLC25A15/AIFM1/PNPT1/SLC25A2/DMAC2L/CHCHD4/TIMM50/STOML2/SLC25A38/MCUR1/ATP5F1E/SLC25A4/TIMM17A/DNAJC30/SLC25A22/ATP5F1D/ROMO1/TIMM44/SFXN4/MRPL18/ATP5PF/ATP5ME/ATP5PO/SMDT1/ATP5PD/ATP5MC1/SLC8B1/MPC1/TOMM40/SLC25A20/CPT1A/SLC39A8/ATP5MF/SFXN2/SLC25A37/CPT2/SLC25A1/PSEN2/MID1IP1/TST/UCP2 |
| GOBP_REGULATION_OF_SYSTEMIC_ARTERIAL_BLOOD_PRESSURE_BY_CIRCULATORY_RENIN_ANGIOTENSIN | 0.746942072 | 1.88442929 | 0.000995439 | 0.026091981 | CPA3/MME/ACE2/F2R/ENPEP/PCSK5/CTSG/CMA1 |
| GOBP_POSITIVE_REGULATION_OF_CELL_CYCLE_PROCESS | 0.375079422 | 1.450587945 | 0.001002354 | 0.026172579 | IGF1/EDN1/PLCB1/FAP/PLK2/PHIP/ATRX/CCN2/CNOT6L/HNRNPU/SVIL/SMC5/NIPBL/PKN2/CEP295/UHRF2/CSPP1/PKD2/BTG2/GEN1/KMT2E/TFDP2/MAP3K20/RAD51B/CDC27/TPR/KIF23/EPGN/PCID2/SFPQ/INSR/DYRK3/CEP120/ANKRD17/ATM/CD28/EZH2/CDK1/GADD45A/CCND2/RACGAP1/SLF1/ANXA1/PKP4/MIR29A/DDX3X/PBX1/IGF2/MDM4/FGF10/KIF20B/CENPJ/CCND1/TMOD3/CIT/CNOT2/FAM83D/NDC80/CNOT4/ROCK2/CNOT6/RAD21/TAS2R13/RBL2/RDX/SIN3A/CDC73/CUL3/NUSAP1/MIR222/SLF2/DLGAP5/ADAM17/KIF14/UBE2E2/CNOT1/MTA3/WNT5A/SPAG5/DYNC1H1/CDC14B/PLAGL1/CUL4A/EGFR/ID2/DBF4/CDKN1A/DTL/CDC16/ANAPC4/MAD2L1/MTBP/TFDP1/NUMA1 |
| GOMF_GLYCEROPHOSPHOLIPID_FLIPPASE_ACTIVITY | 0.816493039 | 1.888372887 | 0.001027308 | 0.026721766 | ABCB1/ATP11A/ATP11C/MFSD2A/ATP8B1/ATP10A |
| GOBP_ACTIVATION_OF_INNATE_IMMUNE_RESPONSE | -0.399992436 | -1.58498053 | 0.001036071 | 0.026783012 | PSMD9/PQBP1/PSME3/PSMA3/PSMB4/PSMB5/ZCCHC3/PSMC3/MUCL1/PSMC4/PSMD11/PSMB1/PSMD8/PSMD3/HRAS/PSMA7/RELA/PRKACA/SYK/STING1/PSMB7/PRKCD/LYN/MUC16/MUC20/MAVS/PSMB3/ICAM3/FFAR2/PYCARD/ICAM2/TLR4/FCN1/MUC21/MNDA/NLRC4/MUC4/FCER1G/LILRA2/CLEC4E/MUC15/MUC5B/HCK |
| GOBP_DETECTION_OF_ABIOTIC_STIMULUS | 0.442571232 | 1.595455037 | 0.001037761 | 0.026783012 | ITGA2/PIEZO2/KIT/CXCL12/SERPINE2/ADGRV1/CXCR4/CTNNB1/ANO1/TTN/LXN/NMT2/PKD2/CDH2 |
| GOBP_RESPONSE_TO_MUSCLE_STRETCH | 0.726474174 | 1.892046825 | 0.001041452 | 0.026783012 | ANKRD1/EDN1/FOS/RYR2/CTNNB1/TTN/CDH2/NFKB1/DMD/PIK3CA |
| GOBP_FACE_DEVELOPMENT | 0.595719138 | 1.789199481 | 0.001048006 | 0.026850238 | ARID5B/TGFB3/TIPARP/ZFAND5/NIPBL/COL1A1/PLEKHA1/LRP6/LEF1/CHD7/RAB3GAP1/CRISPLD1/ALDH1A3 |
| GOBP_NEGATIVE_REGULATION_OF_CYTOKINE_PRODUCTION | -0.320351467 | -1.388187908 | 0.00106884 | 0.02723718 | IRAK3/TNF/POMC/RAC1/XCL1/NCKAP1L/FURIN/TGFB1/CSK/MUL1/NOD2/IKBKE/HDAC7/FKBP1A/LGALS9/RARA/TRIB2/PIN1/KAT5/RGCC/KLF2/MIR27B/GPNMB/C5AR2/BCL6/GSTP1/ILRUN/MERTK/LAPTM5/JAK3/CD274/CD33/NMI/CX3CR1/ARRB1/SARS1/MAVS/BCL3/GIT1/TRIM25/RNF135/ORM1/PYCARD/MEFV/SLC2A10/MIR15A/TLR4/ARG1/SIRPA/FOXJ1/VSIR/PTPN6/NLRP12/PTPRS/LGALS9C/SLC11A1/NDRG2/SCGB1A1/TLR6/CEACAM1/GBA/CMKLR1/TREM2/MIR145/KLF4/PPP1R11/APOD/LILRB4/LTF/LILRA5/LILRB1/MMP8/IL1RL1/ARRB2/TLR8/VSIG4/LRRC32/CR1/HMOX1/IL1R2 |
| GOMF_LYS63_SPECIFIC_DEUBIQUITINASE_ACTIVITY | 0.817625754 | 1.85451709 | 0.001075054 | 0.02723718 | TNFAIP3/CYLD/DESI2/STAMBPL1/YOD1/OTUD4/USP27X/ATXN3/BRCC3 |
| GOBP_REGULATION_OF_RESPONSE_TO_WOUNDING | 0.425216981 | 1.559233385 | 0.001075099 | 0.02723718 | CPB2/EDN1/SERPINE2/FAP/EPHA4/TNFAIP3/F11/PHLDB2/MIR21/F2R/THBS1/CASK/CXCR4/GJA1/SERPINE1/PRKG1/SERPINB2/ADTRP/HBEGF/SLC12A2/CLASP2/TNR/ATP7A/SMAD3/KANK1/PROS1/UBASH3B/ARFGEF1/PDGFA/FGG/NFE2L2/LRIG2/C1QTNF1/CD9/FGF2/PLPP3/ANXA1/FERMT2/SRSF6/MIR29A/MYOZ1/ITGB1 |
| GOBP_MESENCHYMAL_CELL_DIFFERENTIATION | 0.403242549 | 1.521883155 | 0.001085168 | 0.027390436 | SFRP2/HAS2/SEMA3D/EPHA3/EDN1/FGFR2/HGF/BMP5/PDCD4/TGFB3/PHLDB2/SEMA3C/MIR21/HIF1A/EDNRA/FRZB/CTNNB1/BMPR1A/COL1A1/SEMA6D/ERBB4/RANBP3L/SFRP1/STAT1/CLASP2/CDH2/DDX5/LRP6/SMAD3/SNAI2/SPRY1/LEF1/MEF2C/SEMA5A/FAM172A/BCL2/EZH2/TASOR/DDX17/FN1/FERMT2/LOXL2/TGFB2/FGFR1/PBLD/NRP1/SMAD2/FGF10/LDLRAD4/FAM83D/EFNA1/ACVR1/SEMA3A/DLG5/GREM1/MDK/MIR222/RBPJ/BAMBI/TAPT1/SMAD4/RDH10/NOLC1/MIR29B1/WNT5A/PPP2CA/TIAM1 |
| GOBP_NEGATIVE_REGULATION_OF_LOCOMOTION | 0.360540429 | 1.429286446 | 0.001099916 | 0.027660247 | SFRP2/ANGPT2/THY1/CCL21/SEMA3D/SULF1/ADAMTS9/CXCL12/C5/ROBO2/PLCB1/BMP5/DPYSL3/PHLDB2/SEMA3C/MIR21/THBS1/COL3A1/ROBO1/MEOX2/IL33/NAV3/ARID2/ARRDC3/LIMCH1/NF1/PTPRK/SRGAP1/GJA1/CD200/BMPR1A/LRCH1/SERPINE1/DPP4/PRKG1/SEMA6D/KLRC4-KLRK1/DCN/ERBB4/DLC1/IGFBP3/TP53INP1/ADTRP/SFRP1/MCTP1/CLASP2/OSBPL8/MEF2C/MYOCD/KANK1/SEMA5A/PPARG/BCL2/CD200R1/PPARGC1A/CXCL13/NFE2L2/SRGAP2C/ATP2B4/CD74/MITF/GADD45A/FGF2/RYK/STAP1/TRIB1/VCL/MIR29A/PTPN2/DUSP1/PBLD/NRP1/FBLN1 |
| GOBP_REGULATION_OF_ANGIOTENSIN_LEVELS_IN_BLOOD | 0.817177325 | 1.853499974 | 0.001109455 | 0.02779757 | CPA3/MME/ACE2/ENPEP/CTSG/CMA1 |
| GOBP_NADP_METABOLIC_PROCESS | -0.608463817 | -1.880718869 | 0.001140779 | 0.028477688 | PGAM1/PGLS/TP53I3/H6PD/IDH2/TKT/IDH1/NADK/DCXR/KCNAB2/PGD/G6PD/TALDO1/NQO1 |
| GOMF_METALLOCARBOXYPEPTIDASE_ACTIVITY | 0.665711964 | 1.83297179 | 0.00115431 | 0.028580036 | AGBL1/CPA3/CPB2/ACE2/CPE/CPXM2/CPXM1/NAALAD2/VASH1/CPM/FOLH1/VASH2 |
| GOMF_METALLOPEPTIDASE_ACTIVITY | 0.419592179 | 1.558158452 | 0.001157446 | 0.028580036 | AGBL1/CPA3/ERAP2/MME/ADAMTS9/CPB2/TLL1/ACE2/MMP7/BMP1/PHEX/CPE/ENPEP/PAPPA/MMP16/ADAMTSL1/CPXM2/CPXM1/ADAM12/STAMBPL1/TRHDE/ADAMTS12/ADAMTS3/ADAMTS6/ADAM28/ADAM23/ADAMTS4/LNPEP/MBTPS2/MYSM1/NAALAD2/ADAMTS5/EIF3H/VASH1/CPM/ERAP1/XPNPEP2/FOLH1/ADAM19/VASH2/BRCC3/CPQ/ADAM17/MMP19/MMP2/ADAM20/LVRN/METAP1/ADAM22/OMA1/LAP3/CPA6/LTA4H/YME1L1/LMLN |
| GOBP_HYDROGEN_PEROXIDE_CATABOLIC_PROCESS | 0.667186211 | 1.84886303 | 0.001158508 | 0.028580036 | HBB/HBA2/HBD/HBG2 |
| GOBP_MICROTUBULE_ORGANIZING_CENTER_ORGANIZATION | 0.445329313 | 1.596648042 | 0.001161654 | 0.028580036 | CHORDC1/PLK2/GCC2/CTNNB1/PCM1/KIF3A/CNTLN/SLC16A1/CEP295/CEP192/PKD2/GEN1/CCP110/CLASP2/KIAA0753/PPP1R12A/FBXW11/SPICE1/CEP120/UVRAG/ALMS1/CEP68/SSX2IP/SIRT1/CDK1/GADD45A/CEP44/PAFAH1B1/CENPJ/MCPH1/NDC80/ROCK2/CHMP4C/USP33/NIN/HAUS6/HAUS3/SDCCAG8/SGO1/PKHD1/CHMP2B/TUBE1/BBS4/CHD3/CDK5RAP2/BCAS3/KIF11/RTTN/ODF2/CKAP5 |
| GOBP_REGULATION_OF_TOLL_LIKE_RECEPTOR_SIGNALING_PATHWAY | -0.491459124 | -1.714576323 | 0.001178479 | 0.02873442 | TLR5/LY96/LYN/TICAM1/TLR2/TYRO3/CD300A/TLR4/FLOT1/CD300LF/PTPRS/SMPDL3B/TLR6/TLR1/LTF/LILRA2/ARRB2/CD14/BPIFB1 |
| GOBP_REGULATION_OF_PHAGOCYTOSIS | -0.456073918 | -1.680148387 | 0.001179693 | 0.02873442 | TNF/NCKAP1L/CSK/NOD2/CALR/CYBA/SNX3/PIP4P2/FPR2/SYK/PTPRJ/MERTK/CCL2/TLR2/PTX3/CD300A/PYCARD/SIRPB1/SIRPA/SCARB1/CD300LF/DNM2/IL15RA/SLC11A1/DYSF/TREM2/CLEC7A/FCER1G/GATA2/MFGE8/HCK/FGR |
| GOBP_NEGATIVE_REGULATION_OF_LYMPHOCYTE_MEDIATED_IMMUNITY | -0.551141744 | -1.806692969 | 0.001180578 | 0.02873442 | XCL1/NOD2/HLA-E/LGALS9/SERPINB4/C4BPB/PARP3/BCL6/ARG1/FOXJ1/PTPN6/CEACAM1/LILRB4/LILRB1/ARRB2/CR1 |
| GOBP_EXTRINSIC_APOPTOTIC_SIGNALING_PATHWAY | 0.398761482 | 1.500624283 | 0.001185748 | 0.028757542 | SFRP2/INHBA/IGF1/HGF/BMP5/TNFAIP3/EYA4/THBS1/PHIP/IL33/CYLD/ITGAV/SERPINE1/HSPA1B/HSPA1A/SFRP1/RB1CC1/ZDHHC3/ZMYND11/BCL2L11/SMAD3/SNAI2/SH3RF1/SKIL/ATF3/MCL1/BIRC6/RPS6KB1/FGG/BCL2/PPP2R1B/DCC/TNFRSF10B/ITM2C/ITGA6/YAP1/IL7/DDX47/ICAM1/DDX3X/TGFB2/FGFR1/FGF10/FAS/STK3/APPL1/FAF1/ACVR1/PF4/FYN/SGPP1/FEM1B |
| GOBP_NEPHRIC_DUCT_MORPHOGENESIS | 0.852902371 | 1.838994176 | 0.001190326 | 0.028766214 | EPHA4/AHI1/GPC3 |
| GOMF_PROTEIN_SERINE_THREONINE_KINASE_ACTIVITY | 0.350583608 | 1.409110266 | 0.001202852 | 0.028910433 | LTBP1/DCLK1/PLK2/RPS6KA5/STK32A/MARK1/PRKCQ/STK38L/CAMK4/MAP3K13/MAP3K4/TOP1/LRRK2/CASK/CLK1/PRKACB/BMPR1A/MAPK6/WNK1/TTN/MAPK10/RIPK2/PKN2/PRKG1/SIK2/MAP4K4/MAPK8/GRK3/PRKD1/TRIO/PRKCI/AKAP13/CDK6/MAP4K5/PIM2/TAOK1/CAMK2D/ACVR2A/RPS6KA6/CDKL2/STK17B/PHKA1/MAP3K20/CDK17/RPS6KA3/EIF2AK2/EIF2AK3/CDK15/MAP3K7/PRKD3/CDC42BPA/SMG1/PIK3CG/RPS6KB1/NEK1/TLK1/PRKAA2/PIK3CA/BRAF/DYRK3/SLK/HIPK3/ATM/CSNK1A1/CLK4/LTBP4/ROCK1/TAF1/SIK3/CDK1/MAP3K2/PRPF4B/CDK14/ERN1/MAP3K5/PRKCH/VRK2/RPS6KC1/TBK1/MAST4/CSNK1G3/DCLK2/CIITA/CAMK1D/CPNE3/DAPK2/BMP2K/MAPK9/NEK9/CIT/STK3/MAP3K1/RIOK2/TRPM7/PIK3R4/MAP4K3/ROCK2/MARK3/ATR/ACVR1 |
| GOBP_SERINE_FAMILY_AMINO_ACID_BIOSYNTHETIC_PROCESS | -0.738883093 | -1.92743622 | 0.001204778 | 0.028910433 | DHFR2/SEPHS1/CTH/DHFR/SEPHS2/SHMT1/PSAT1/SHMT2/CBS/PHGDH |
| GOMF_FLIPPASE_ACTIVITY | 0.773456296 | 1.883448353 | 0.001224142 | 0.029272031 | ABCB1/ATP11A/ATP11C/MFSD2A/ATP8B1/ATP10D/ATP10A/TMEM30A/ATP8A1 |
| GOBP_CHROMOSOME_SEGREGATION | 0.370329211 | 1.450372274 | 0.001232939 | 0.029379295 | GEM/EML4/SMC4/ATRX/TOP1/HNRNPU/CTNNB1/SMC6/TOP2B/SMC5/FBXW7/CENPC/NIPBL/TTN/TNKS/GEN1/STAG2/PUM2/PDS5A/CDC27/TPR/STAG1/KIF23/SPICE1/TLK1/PCID2/SFPQ/UVRAG/NR3C1/ATM/RACGAP1/SLF1/PRC1/ESCO1/CENPF/PIBF1/RAD18/DDX3X/DUSP1/MLH3/KNTC1/USP9X/CENPE/KIF18A/TENT4A/FAM83D/RIOK2/NDC80/BEX4/ZNF207/WAPL/CHMP4C/KNL1/CCNE2/SYCP2/RAD21/PDS5B/NEK11/HDAC8/PTTG2/MRE11/TOP2A/BOD1/CUL3/NUSAP1/ANAPC1/SGO1/APC/MKI67/SLF2/DLGAP5/KIF14/PUM1/SMC3/CENPW/CENPN/LATS1/CHMP2B/SPAG5/POGZ/DYNC1H1/NCAPD3/IHO1/SMARCAD1/DSCC1/ESCO2/DIS3L2/PHF13/BRIP1/RIOK3/CDK5RAP2/BUB3/NAA50/CDC16/ANAPC4/MAD2L1/NUMA1/FANCD2/CSNK2A2/AXIN2/HFM1/TEX12/CDCA2/INO80 |
| GOBP_NEGATIVE_REGULATION_OF_VIRAL_LIFE_CYCLE | -0.696949932 | -1.985562141 | 0.0012511 | 0.029708188 | LY6E/PPIA/TRIM26/SNX3/IFITM3/TRIM8/PTX3/TRIM25/FCN1/IFITM2/FCN3 |
| GOBP_REGULATION_OF_CHOLESTEROL_BIOSYNTHETIC_PROCESS | -0.554725552 | -1.842910395 | 0.001296175 | 0.030671643 | GPAM/NFYC/SCD/APOE/SC5D/SCAP/FDFT1/ELOVL6/SREBF2/HMGCR/LPCAT3/HMGCS1/SREBF1/MVK/MVD/TM7SF2/FDPS/FASN/DHCR7/LSS |
| GOBP_GRANULOCYTE_CHEMOTAXIS | -0.422581018 | -1.631553442 | 0.001304216 | 0.030741526 | CSF1R/RAC2/CCL2/MPP1/BST1/PIK3CD/S100A14/CCL23/C5AR1/CMKLR1/ITGB2/ADGRE2/PREX1/FCER1G/RIPOR2/C3AR1/CXCR2/CXCR1/CSF3R/S100A8/SAA1/S100A9/S100A12 |
| GOBP_REGULATION_OF_NAD_P_H_OXIDASE_ACTIVITY | -0.840042417 | -1.871752177 | 0.001308296 | 0.030741526 | GNAI2/SLAMF8/AGTR1 |
| GOCC_CENTRIOLE | 0.438582545 | 1.581075952 | 0.001312661 | 0.030741526 | AHI1/PLK2/PCM1/OFD1/CCDC146/KIF3A/HSPA1B/HSPA1A/CNTLN/CEP290/CEP295/ODF2L/CEP192/LRRCC1/DZIP1/CCP110/HERC2/KIAA0753/SPICE1/CEP128/IQCB1/CEP120/ALMS1/CEP83/ROCK1/TOPORS/CEP78/CCDC68/CEP44/CEP170/HSPA6/CENPJ/KIF2A/CEP350/NIN/SCLT1/KIAA0586/CEP55/SDCCAG8/TOP2A/CEP43 |
| GOBP_SUPEROXIDE_ANION_GENERATION | -0.604105737 | -1.867248351 | 0.00132916 | 0.03102131 | TGFB1/CYBA/CYBB/HVCN1/FPR2/SYK/GSTP1/PRKCD/NCF1/DUOX1/NCF2/CLEC7A/ITGB2/CD177/ITGAM |
| GOBP_LEUKOCYTE_TETHERING_OR_ROLLING | 0.647784549 | 1.813202162 | 0.001351164 | 0.031427256 | VCAM1/SELE/CCL21/CXCL12/SELP/ITGA4 |
| GOBP_CARDIAC_CHAMBER_DEVELOPMENT | 0.433102091 | 1.577974946 | 0.001364446 | 0.031628237 | SFRP2/ROBO2/FGFR2/BMP5/ZFPM2/ANK2/SEMA3C/HIF1A/PKP2/ROBO1/FRS2/CPE/CCN1/RYR2/BMPR1A/PDE2A/ADGRG6/SOS1/NPHP3/SLIT3/MEF2C/MATR3/MYOCD/RBP4/CHD7/CNTRL/ADAMTS6/FHL2/LRP2/TRIP11/NSD2/TGFB2/MDM4/NRP1 |
| GOBP_REGULATION_OF_WNT_SIGNALING_PATHWAY | 0.364125379 | 1.445861248 | 0.00139 | 0.032098748 | SFRP2/WIF1/SULF1/BICC1/EGR1/SFRP4/FGFR2/FZD7/TNFAIP3/MACF1/RSPO3/FRZB/GPC3/CYLD/LRRK2/MLLT3/CAPRIN2/CTNNB1/ZRANB1/UBR5/CMAHP/WNK1/COL1A1/SPIN1/USP47/SFRP1/TNKS/NPHP3/GRB10/SMURF2/CDH2/TBL1XR1/PSME4/TNKS2/SCYL2/LRP6/TMEM131L/SMAD3/SNAI2/JADE1/NFKB1/LEF1/INVS/IGFBP4/ANKRD6/KANK1/SEMA5A/SFRP5/USP34/PPM1B/BTRC/DIXDC1/LRP4/CSNK1A1/LIMD1/IFT80/USP8/YAP1/CDK14/PLPP3/RNF146/RBMS3/CSNK1G3/DDX3X/CBFB/NXN/KPNA1/FZD6/WLS/FGF10/SCEL/LGR4/STK3/TMEM64 |
| GOCC_DNA_PACKAGING_COMPLEX | -0.435049831 | -1.647263254 | 0.001395702 | 0.032098748 | H2BC8/H2BC9/H2AC12/H2BC19P/H4C13/H2BC6/H2AC4/H4C6/GLYR1/H3C1/H2AJ/H2BC7/H2BC12/H2BC21/H1-3/H4C2/H3-3A/SPHK2/H2BC4/H1-10/H1-4/H2AC6/H2AC8/H4C15/H2AZ1/H2BC5/MACROH2A1/H2AC19/H4C14/H2AC20/H4C3/H3C11/H2AC21/H1-2/H1-0 |
| GOMF_SINGLE_STRANDED_DNA_BINDING | 0.459986601 | 1.620448886 | 0.001398874 | 0.032098748 | SMC4/IGHM/JCHAIN/TOP1/HNRNPU/WDR48/SMC6/FUBP1/FAM111A/MSH2/HNRNPA2B1/RAD51B/DHX36/POLA1/RPA4/HNRNPA1/RBMS1/MCM6/SETMAR/DHX9/MSH3/ANXA1/RNF138/MYEF2/RAD18/MCM9/PURB/TERF2IP/HSPD1/ERCC5/RAD50/TERF1/TDP1/HMGB1/POLR3C/RTF1/CRY2/RADX |
| GOMF_PROTEIN_SERINE_KINASE_ACTIVITY | 0.380693366 | 1.443220949 | 0.001422555 | 0.032489926 | DCLK1/RPS6KA5/STK32A/MARK1/STK38L/LRRK2/CASK/WNK1/TTN/RIPK2/SIK2/MAP4K4/TRIO/MAP4K5/PIM2/TAOK1/RPS6KA6/STK17B/RPS6KA3/EIF2AK2/EIF2AK3/CDC42BPA/SMG1/PIK3CG/RPS6KB1/NEK1/TLK1/PRKAA2/PIK3CA/BRAF/SLK/HIPK3/ATM/CSNK1A1/ROCK1/TAF1/SIK3/PRPF4B/ERN1/VRK2/RPS6KC1/TBK1/MAST4/CSNK1G3/DCLK2/CIITA/DAPK2/BMP2K/NEK9/CIT/STK3/RIOK2/TRPM7/PIK3R4/MAP4K3/ROCK2/MARK3/ATR/TNIK/MASTL/NEK11/NEK7/HUNK/PAK3/WNK3/AKT3/LRRK1/PIKFYVE/TTBK2/STK39/TRPM6/LATS1/KSR1/PRKX/STK33/OXSR1/SIK1/RIOK3/CSNK1G1/CILK1/TAOK3/SRPK2/PRKY/CSNK2A2/ULK2/CSNK1E/CHEK2/CHEK1/ALPK3/RIOK1/STK17A/NEK3/SGK1/SGK2/DAPK1/PRKDC/MELK/AURKA/TLK2/AAK1 |
| GOBP_NEGATIVE_REGULATION_OF_VIRAL_ENTRY_INTO_HOST_CELL | -0.719850839 | -1.948210569 | 0.001425457 | 0.032489926 | LY6E/TRIM26/SNX3/IFITM3/TRIM8/PTX3/TRIM25/FCN1/IFITM2/FCN3 |
| GOBP_KILLING_OF_CELLS_OF_OTHER_ORGANISM | -0.502919553 | -1.72231571 | 0.00143251 | 0.032541854 | AZU1/CXCL6/LCE3B/ELANE/APOL1/TUSC2/DEFA4/PGLYRP1/RPS19/H2BC12/KRT6A/HAMP/ROMO1/SYK/GNLY/GAPDH/ARG1/BCL2L1/CLEC7A/LTF/DEFA1B/S100A12 |
| GOBP_REGULATION_OF_CELL_DIVISION | 0.418407686 | 1.545412322 | 0.001444974 | 0.032715948 | SFRP2/MACC1/PDGFD/FGFR2/TGFB3/PLK2/FGF7/PPBP/GAREM1/MLLT3/SVIL/AHCTF1/PKN2/KIF13A/CSPP1/CCP110/PIK3C3/BIRC6/KIF23/PDGFA/FGF1/KLHL13/UVRAG/SETD2/EFHC1/VEGFA/FGF2/RACGAP1/PRC1/CAT/PKP4/TGFB2/IGF2/KIF20A/KIF20B/CIT/PRPF40A/PIK3R4/CHMP4C/THBS4/TAS2R13/RBL2/PDGFC/CUL3/INTU/MDK |
| GOBP_L_SERINE_METABOLIC_PROCESS | -0.836678858 | -1.864257617 | 0.001468084 | 0.033129123 | SHMT1/PSAT1/SHMT2/CBS/PHGDH |
| GOCC_HIGH_DENSITY_LIPOPROTEIN_PARTICLE | -0.651992128 | -1.888504075 | 0.001477743 | 0.033154994 | APOL1/APOE/APOC1/APOO/HPR/SAA2/PLA2G7/SAA1 |
| GOBP_REGULATION_OF_ALTERNATIVE_MRNA_SPLICING_VIA_SPLICEOSOME | 0.548354408 | 1.720827102 | 0.001478961 | 0.033154994 | HNRNPU/RBM25/MBNL2/RBFOX2/DDX5/FMR1/MBNL1/HNRNPA1/MBNL3/FAM172A/FXR1/YTHDC1/RBM5/DDX17/WTAP/SRSF6/TRA2B/RBM7/NSRP1/CELF2/KHDRBS1/DYRK1A/REST/RBM15/RBMX/SMU1/NOVA1/KHDRBS2 |
| GOBP_DENDRITE_DEVELOPMENT | 0.392349529 | 1.483557384 | 0.001500158 | 0.033519923 | TRPC6/BMP5/EPHA4/PREX2/DCLK1/PLK2/PTPRD/MARK1/DOCK10/ACSL4/VLDLR/LRRK2/CAPRIN2/CAMSAP2/ITSN1/PRKG1/SIPA1L1/UBA6/PPP1R9A/MFSD2A/RAPGEF2/RBFOX2/DNM1L/DHX36/KIDINS220/MEF2C/FMR1/PDLIM5/ZDHHC15/DCC/TANC2/SRGAP2C/LRP4/EZH2/DNM3/SHANK2/LPAR1/PPP3CA/RELN/MAP1B/FBXO31/CAMK1D/NLGN1/NRP1/OPA1/PICALM/EFNA1/STRN/BHLHB9/UBE3A/SEMA3A/FYN/TNIK/DLG5/COBL/ABI2/FBXW8/SRGAP2/PAK3/ARF4/TMEM106B/SDC2/DBN1/EIF4G2/TIAM1/BCL11A/DTNBP1/BBS4/NEDD4/MAP2 |
| GOMF_CHEMOKINE_ACTIVITY | 0.587908941 | 1.777663513 | 0.00150629 | 0.03354694 | CCL21/CXCL9/CXCL12/C5/CCL5/PPBP/CXCL11/CCL18/CXCL10/CXCL14/CXCL2 |
| GOBP_RIBONUCLEOTIDE_CATABOLIC_PROCESS | 0.615856504 | 1.823241612 | 0.00151166 | 0.033556874 | PDE1A/PDE7B/NT5E/PDE4D/PDE8B/PDE8A/PDE5A/PDE10A/PDE2A/PDE4B/NUDT4 |
| GOCC_FIBRILLAR_CENTER | 0.440914775 | 1.580814059 | 0.001523911 | 0.033718999 | KIT/ANKRD1/PTPN13/SAMD4A/RAI14/CAMK4/TOP1/NRIP1/SESN1/FAM111A/AFF4/SP140/RNMT/NFIB/RPS6KA6/WDR43/RERG/MALT1/WDR33/SMARCA5/NOP58/UBD/HERC4/TAF4B/USO1/SIRT1/CD2AP/ZNF415/DDX46/POLR1E/RSAD2/COIL/URB1/DUSP11/EZR |
| GOCC_PROTON_TRANSPORTING_TWO_SECTOR_ATPASE_COMPLEX_PROTON_TRANSPORTING_DOMAIN | -0.687351483 | -1.90463481 | 0.001541803 | 0.034004486 | ATP6V0E1/DMAC2L/ATP6V0C/ATP6V0E2/TCIRG1/ATP5PF/ATP6V0D2/ATP5ME/ATP5PO/ATP5PD/ATP5MC1/ATP6V0B/ATP5MF/ATP6V0D1 |
| GOBP_MITOTIC_SISTER_CHROMATID_SEGREGATION | 0.415123917 | 1.531097133 | 0.00159032 | 0.0349614 | EML4/SMC4/ATRX/HNRNPU/SMC5/CENPC/NIPBL/TTN/TNKS/GEN1/PDS5A/CDC27/TPR/KIF23/PCID2/ATM/RACGAP1/SLF1/PRC1/CENPF/PIBF1/DUSP1/KNTC1/CENPE/KIF18A/TENT4A/RIOK2/NDC80/ZNF207/CHMP4C/RAD21/PDS5B/PTTG2/BOD1/CUL3/NUSAP1/ANAPC1/SGO1/APC/SLF2/DLGAP5/KIF14/CHMP2B/SPAG5/POGZ/NCAPD3/DSCC1/DIS3L2/PHF13/CDK5RAP2/BUB3/NAA50/CDC16/ANAPC4/MAD2L1/NUMA1 |
| GOBP_REGULATION_OF_CELL_MORPHOGENESIS | 0.366795492 | 1.428598008 | 0.001667053 | 0.036530429 | POSTN/ENPP2/HAS2/KIT/EPHA4/MYH10/GBP1/MACF1/RHOJ/MIR21/PTPRD/PHIP/SPAG9/BRWD1/MAP3K13/CXCR4/CAPRIN2/ZRANB1/ARHGAP15/RHOH/FGD4/RND3/DLC1/MFSD2A/EPS8/MYO9A/ARHGEF7/DOCK1/RND1/DNMBP/RASA1/DNM1L/DHX36/FGD6/MKLN1/PALMD/KANK1/ZMYM4/EPB41L3/FGG/ITSN2/ARHGAP18/SMURF1/LIMD1/ATP10A/LIMS1/LPAR1/VEGFA/CD44/DOCK5/FAM171A1/RELN/ANXA1/RHOBTB3/FN1/RHOBTB1/RHOU/FBXO31/FERMT2/ICAM1/FMNL2/DLG1/BRWD3/SPARC/NRP1/FBLN1/OPA1/PLXNA4/EZR/PRPF40A/LARP4/PDZD8/ITGA7/BHLHB9/DIAPH1/WDPCP/FYN/SEPTIN7/CCL24/CDC42SE2/CAMSAP1/RDX/RHOB/PALM2AKAP2/FBXW8/MDK/PTK2/PAK3/BAMBI |
| GOCC_RESPIRATORY_CHAIN_COMPLEX_IV | -0.709007307 | -1.904986835 | 0.001678793 | 0.036669798 | COX6A1/COX7C/UQCRFS1/COX8A/COX4I1/COA6/NDUFA4/C15orf48/NDUFA4L2/COX4I2/COX5A/COX7B/COX6B1 |
| GOBP_VITAMIN_METABOLIC_PROCESS | -0.407602372 | -1.585979792 | 0.001685922 | 0.036707852 | SLC23A1/MTHFS/MMADHC/AOX1/AMN/FLAD1/PDXP/CYP4F12/UBIAD1/CYB5R3/MTHFR/TCN1/DHFR2/SNAI1/GSTO1/TNF/SLC19A1/CD320/MMACHC/FPGS/PCCB/TTPA/PIAS4/SLC46A1/SLC25A19/PNPO/CYB5A/CYP2R1/DHFR/SLC5A6/ALDH1L1/SLC2A1/SHMT1/RBP2/AKR1A1/ACP5/CYP27A1/VDR/MMAB/ACP3/VNN1/CBR1/AKR1C3/RGN/PSAT1/SHMT2/TCN2/VNN2 |
| GOBP_OLIGOSACCHARIDE_LIPID_INTERMEDIATE_BIOSYNTHETIC_PROCESS | -0.714301997 | -1.933193136 | 0.001692085 | 0.036724705 | ALG12/RFT1/ALG1/DOLK/ALG8/DOLPP1/DPAGT1/SRD5A3/MVD/DHDDS/ALG3/MPDU1 |
| GOBP_NEGATIVE_REGULATION_OF_RESPONSE_TO_REACTIVE_OXYGEN_SPECIES | 0.730505459 | 1.842962038 | 0.001698088 | 0.036738005 | HGF/NR4A3/MIR21/PDE8A/LRRK2/H19/MET/NFE2L2 |
| GOBP_BRANCHING_MORPHOGENESIS_OF_A_NERVE | 0.845638956 | 1.823333088 | 0.001713724 | 0.036958962 | FGFR2/MAP3K13/LRRK2 |
| GOBP_IMMUNE_RESPONSE_INHIBITING_SIGNAL_TRANSDUCTION | -0.830396172 | -1.850258763 | 0.001747716 | 0.037573128 | LYN/CD33/LILRB4/LILRB1/LILRB2 |
| GOMF_PROTEIN_HETERODIMERIZATION_ACTIVITY | -0.317506999 | -1.367018638 | 0.001773962 | 0.038017454 | CHRAC1/H2BC8/H2BC9/ADRA2C/H2AC12/H2BC19P/H4C13/ADRA2A/H2BC6/H2AC4/PIK3R2/HEXA/H4C6/TAF8/NFYC/RRAGA/PEF1/H3C1/KATNB1/BAX/MAFG/HIP1/SAE1/IRAK3/H2AJ/TP53/ERBB2/H2BC7/H2BC12/H2BC21/FZD4/PAFAH1B3/H4C2/BCL2A1/SDCBP2/YWHAE/ADD1/H3-3A/CYBA/TPM2/SLC51B/P4HB/PHB2/IRAK1/H2BC4/BCL2L2/LSM6/USF1/CYBB/ITGA3/LSM5/H2AC6/BAK1/RCC1/H2AC8/PPP2R1A/EPAS1/TAF12/SUPT4H1/PRMT5/H4C15/TAF6/TAF6L/CEBPB/H2AZ1/H2BC5/MACROH2A1/P2RY1/RRAGD/PDGFB/ADRB1/USF2/TLR4/BCL2L1/PHB/H2AC19/PDSS1/H4C14/MLX/H2AC20/H4C3/TLR6/CEACAM6/AOC3/ADRA1B/H3C11/H2AC21/GCA/AGTR1/ADRA1A |
| GOBP_NADPH_REGENERATION | -0.709927461 | -1.869530566 | 0.001787157 | 0.038180176 | TP53/DERA/PGAM1/PGLS/H6PD/TKT/IDH1/PGD/G6PD/TALDO1 |
| GOCC_CELL_CORTEX | 0.37113376 | 1.433121722 | 0.001814354 | 0.038640076 | SELE/HMCN1/FGFR2/MYH10/PHLDB2/PLEKHH2/CCN2/FER/RAI14/UTRN/DST/CTNNB1/CALD1/MPP7/RHOH/AKAP12/SPIRE1/RND3/DLC1/PPP1R9A/EPS8/FNBP1L/SEPTIN6/ARHGEF7/CALB2/AKAP13/EXOC6B/CTTNBP2/RND1/PKD2/FLNB/CLASP2/MYRIP/CDH2/EXOC6/SEPTIN11/ADD3/GNAI1/CYTIP/MKLN1/SNX9/FGF1/EXOC4/TRPC4/EPB41L2/SPTAN1/SLC4A1/RHOBTB3/RHOBTB1/FERMT2/EXOC2/FRYL/SEPTIN2/UNC13B/FRY/NCL/MYO6/PAFAH1B1/EZR/AQP7/WDPCP/SEPTIN7/ERC1/SPTBN1/MICAL3/RDX/PARD3/PARD3B/COBL/SCIN/RHOB/LANCL2/TRAF6/PTK2/ACTN2/RIC8B/SEPTIN8/SEPTIN10 |
| GOBP_RIBONUCLEOSIDE_TRIPHOSPHATE_BIOSYNTHETIC_PROCESS | -0.477142398 | -1.663813203 | 0.00184949 | 0.039265653 | UCK2/VPS9D1/ATPSCKMT/NME9/ANTKMT/ATP5F1A/NME6/UQCC3/NUDT2/IMPDH2/DMAC2L/ATP6V0C/STOML2/NME5/ATP6V1A/ATP5F1EP2/CAD/ATP5F1E/TGFB1/PINK1/DNAJC30/SPHK2/UCK1/ALDOA/ATP5F1D/ATP5PF/ATP5ME/ATP5PO/ATP5PD/DGUOK/ATP5MC1/ATP5MF/IMPDH1/NME1/ENO1/PKM/NME2/TREM2 |
| GOBP_BICARBONATE_TRANSPORT | 0.597851451 | 1.791994162 | 0.001900153 | 0.040215975 | HBB/HBA2/CA2/CA12/SLC4A7/CA1/SLC4A4 |
| GOBP_CENTRAL_NERVOUS_SYSTEM_NEURON_DEVELOPMENT | 0.495512205 | 1.664756389 | 0.001915047 | 0.040405705 | ROBO2/FGFR2/EPHA4/DCLK1/NR4A2/ROBO1/RAPGEF2/MYCBP2/HSP90AA1/BTG2/NFIB/SCYL2/ATP7A/NDNF/DCC/CDH11/UBB/PAFAH1B1/HSP90AB1/PLXNA4/MPP5/SEMA3A/NIN |
| GOBP_EPITHELIAL_CELL_PROLIFERATION | 0.348226918 | 1.398737433 | 0.001931434 | 0.040625681 | SFRP2/HAS2/IGF1/KIT/SULF1/AREG/BMP6/ITGB3/CPB2/CXCL12/FGFR2/HGF/FAP/BMP5/FZD7/TNFAIP3/ERRFI1/NR4A3/MIR21/HIF1A/THBS1/FGF7/ROBO1/KDM5B/FRS2/GPC3/ITGA4/CASK/WDR48/NF1/PTPRK/GJA1/ZFP36/APLN/FBXW7/APLNR/BMPR1A/FST/NR1D1/IGFBP3/PRKD1/SFRP1/STAT1/CDK6/COL4A3/ACVR2A/NFIB/NR4A1/ATP7A/SMAD3/SNAI2/MEF2C/IGFBP4/SEMA5A/HTRA1/PPARG/FGF1/PPP1R16B/EPGN/IFT74/BTRC/RICTOR/MIR27A/SIRT1/IFT80/VEGFA/YAP1/LAMB1/FGF2/ERN1/SRSF6/LOXL2/MIR29A/DLG1/TGFB2/IGF2/ATF2/FGFR1/PBLD/SPARC/NRP1/FGF10/F3/CCND1/LGR4 |
| GOBP_MESODERMAL_CELL_DIFFERENTIATION | 0.632287033 | 1.788137972 | 0.001949299 | 0.040875292 | SFRP2/ITGA2/INHBA/ITGB3/FGFR2/GJA1/BMPR1A |
| GOBP_CHONDROCYTE_DIFFERENTIATION | 0.4618581 | 1.612011064 | 0.002017201 | 0.042169408 | SFRP2/SULF1/BMP6/CCN2/SOX5/BMPR1A/SLC39A14/NFIB/ADAMTS12/SMAD3/SNAI2/EIF2AK3/MEF2C/LTBP3/MEX3C/IFT80/TRIP11/LOXL2/EFEMP1/TRPS1/SOX6/RUNX2/MBOAT2/GREM1/SCIN/LNPK/MDK/GLG1/CHSY1/CCN3/SERPINH1/RARB/GLI3/PTPN11/OSR1/GLI2/SULF2/PTHLH |
| GOBP_MIDBRAIN_DOPAMINERGIC_NEURON_DIFFERENTIATION | 0.76142185 | 1.854143198 | 0.002041595 | 0.042548836 | SFRP2/NR4A2/CTNNB1/SFRP1/LRP6/RYK |
| GOBP_TUMOR_NECROSIS_FACTOR_SUPERFAMILY_CYTOKINE_PRODUCTION | -0.380460648 | -1.518297678 | 0.002075502 | 0.043063897 | NOD2/HLA-E/FZD5/LGALS9/CYBA/RARA/SPHK2/MIR27B/PTAFR/C5AR2/SYK/PTPRJ/GSTP1/ILRUN/LY96/CD274/CD33/TICAM1/SASH3/CX3CR1/TLR2/MAVS/BCL3/ORM1/PYCARD/MYD88/TLR4/SIRPA/VSIR/PTPN6/DDT/TMEM106A/TREM2/TLR1/CLEC7A/CCR2/LTF/LILRA5/LILRB1/MMP8/LILRA2/ARRB2/CD14 |
| GOBP_LYSOSOME_LOCALIZATION | -0.466496049 | -1.652268229 | 0.002084686 | 0.043063897 | BLOC1S2/HDAC6/VAMP8/FES/PIP4P1/LGALS9/SPHK2/SNAPIN/VPS33B/SYK/UNC13D/LYN/PLEKHM2/LAMTOR1/RAC2/MILR1/KXD1/IL4R/LAT2/PIK3CD/CD300A/BLOC1S1/STXBP2/ADGRE2/FOXF1/GATA2/FGR/HMOX1 |
| GOBP_NEURON_MIGRATION | 0.432810042 | 1.570401457 | 0.002091448 | 0.043063897 | CXCL12/DCLK1/NR4A2/FLRT2/MARK1/COL3A1/CXCR4/TOP2B/PCM1/NIPBL/PRKG1/CCR4/ERBB4/RAPGEF2/NRCAM/SH3RF1/MEF2C/NDNF/DCC/SRGAP2C/LRIG2/VEGFA/RELN/MAP1B/FBXO31/FGFR1/USP9X/NRP1/PAFAH1B1/KIF20B/LRP12/FZD3/SEMA3A/FYN/MDGA1/CX3CL1/AUTS2/SDCCAG8/PEX13/MDK/SRGAP2 |
| GOCC_FIBRILLAR_COLLAGEN_TRIMER | 0.799393669 | 1.813163557 | 0.002091585 | 0.043063897 | LUM/COL3A1/COL1A1/COL5A2/COL1A2 |
| GOBP_CYCLIC_NUCLEOTIDE_MEDIATED_SIGNALING | 0.494812494 | 1.68968707 | 0.002104639 | 0.04319311 | PDE3A/PDE7B/PDE4D/THBS1/PDE10A/APLNR/AKAP6/PDE2A/PRKG1/ADGRG6/RAPGEF2/RGS2/PDE3B/SCTR/GNAI1/NUDT4/PRKAR2B/AHR/LPAR1/CRTC3/PDE7A/PRKAR1A/IRAG1/PDE9A/ADCYAP1/CAP2 |
| GOCC_CELL_CELL_JUNCTION | 0.336642592 | 1.364121206 | 0.00211314 | 0.04319311 | HMCN1/KIT/ITK/PDLIM3/ITGB3/TRPC6/EPHA4/AHI1/ANK2/CDH6/MPDZ/FRMD4B/FLRT2/FAT1/PKP2/FRS2/PATJ/THEMIS/CGNL1/CASK/CTNNB1/PTPRK/GJA1/CLDN18/MPP7/ANK3/DDX58/AKAP6/DPP4/DSG2/PKN2/PGM5/MYO1E/PNN/MAGI1/RAPGEF2/STEAP1/PRKCI/CALB2/RND1/PKD2/ARHGAP24/DNMBP/CXADR/CDH2/ADD3/CLMP/PDZD2/PLXDC1/CDC42BPA/PDLIM5/CD2/CLDN11/EPB41L3/PIK3CA/NEXN/CADM1/TRPC4/CDH11/SSX2IP/CD2AP/LIMD1/LIMS1/ITGA6/RAP1B/FLRT3/VEGFA/PPP3CA/CNN3/PLPP3/ANXA1/JAM3/FERMT2/GJC1/USP53/VCL/PKP4/DLG1/TJP1/ASH1L/POF1B/TMEM47/CCND1/MPP5/TMOD3/RPGRIP1L/DES/AMOTL1/AQP7/DDX6/NFASC/VEZT/DSC3/STRN/NECTIN3/SPECC1L/ACTN1/B4GALT1/KLHL24/DLG5/NCK1/NHS/CLDN7/RDX/PARD3/SDCCAG8/PARD3B/PDLIM1/ABI2/TJP2/APC/LIN7C/ADAM17/WNK3/PKP1/KRIT1/PIKFYVE/SAV1/SLC8A1/CLDN1/CCN3/LAMA1/DBN1/EIF4G2 |
| GOBP_GLYCOSYLATION | -0.332515202 | -1.406237376 | 0.002116874 | 0.04319311 | GAL3ST1/MGAT2/MAN1B1/FKRP/MGAT3/ST6GALNAC6/B4GAT1/ENTPD5/B3GALT6/SDF2L1/PIGB/MOGS/GBA2/MPI/ASGR2/ABO/B4GALT2/MAN1C1/DAD1/DPM2/ALG12/POMT2/POFUT1/RFT1/FUT11/GORASP1/FUT3/UBE2J1/B4GALT3/MUCL1/ALG1/DOLK/VEGFB/DPM3/SLC51B/FUOM/ST6GALNAC2/MAN2A2/B3GAT3/ALG8/NAGPA/RPN1/B3GALT4/NUDT14/GALNT6/DOLPP1/PIGM/ST6GALNAC3/DDOST/DPAGT1/MGAT4B/SLC39A8/MUC16/ST8SIA6/MUC20/GALNT15/PIGV/SRD5A3/NANS/PLOD3/MVD/MGAT1/DHDDS/B3GNT7/GBGT1/ALG3/SLC35C1/GALNT18/FUT1/GBA/ST6GALNAC1/MPDU1/TMTC1/MUC21/GALNT13/GALNT17/B3GALNT1/MUC4/MUC15/MUC5B |
| GOBP_RNA_SPLICING | 0.339339672 | 1.373165971 | 0.002136241 | 0.043458166 | LUC7L3/HNRNPU/ZRANB2/CLK1/RAVER2/SNRPA1/SF3B1/HSPA1A/PNN/ZNF638/HNRNPA3/RBM25/PTBP2/SRSF5/RBM39/HNRNPLL/ZNF326/MBNL2/PRPF39/PPIG/THOC1/RBM41/RBFOX2/SRSF11/IWS1/TIA1/RSRC1/HNRNPA2B1/HNRNPH1/IVNS1ABP/DDX5/HNRNPH3/CSTF3/WDR33/HNRNPR/METTL4/FMR1/CWF19L2/RNVU1-19/MBNL1/HNRNPA1/DHX15/MBNL3/FAM172A/SFPQ/CDC5L/PPARGC1A/FXR1/SREK1/FIP1L1/PPWD1/SCAF11/YTHDC1/UPF3B/CLK4/LUC7L/PSIP1/WBP4/RBM5/PPP4R2/SETX/PRPF4B/TARDBP/GEMIN8/PRPF38B/DDX46/PRPF18/DHX9/ERN1/PCF11/SPEN/DDX17/WTAP/SLC38A2/SYF2/CCAR1/DDX47/PRPF38A/HTATSF1/RNPC3/SRSF6/ZCCHC8/MTREX/METTL14/GCFC2/FAM98B/U2SURP/NCL/SREK1IP1/COIL/TRA2B/HNRNPK/KHDC4/SRSF1/KDM1A/PRPF40A/HNRNPA1L2/METTL3/SRSF10/RBM6/SYNCRIP/RBM7/TTF2/CLNS1A/TCERG1/SON/SRSF7/ZC3H13/NSRP1/NCBP2/CWC27/SF3A3/HSPA8/CWC22/TGS1/ECD/HNRNPD/SART3/CELF2/RNVU1-15/KHDRBS1/RPS13/NUDT21/THOC2/DYRK1A/CDK13/REST/CIR1/DHX35/TRA2A/SNRNP48/DDX42/NUP98/CWC15/ZNF830/RBM15/SNW1/SMNDC1/PPP2CA/RBMX2/CRNKL1/WT1/SNRNP200/RBMX/ZRSR2/RBM12B |
| GOCC_COMPLEX_OF_COLLAGEN_TRIMERS | 0.7000023 | 1.81502886 | 0.002147591 | 0.043559023 | LUM/COL3A1/COL1A1/COL5A2/COL4A3/COL1A2/COL4A1 |
| GOCC_SPINDLE | 0.351521649 | 1.3962444 | 0.002172946 | 0.04392312 | GEM/TRAT1/PLK2/EML4/CYLD/HNRNPU/CTNNB1/SMC6/MTUS1/KIF16B/KIF3A/CEP295/SEPTIN6/TNKS/RIF1/CSPP1/PKD2/MAP7D3/CLASP2/STAG2/TBL1XR1/TFDP2/CEP95/CDC27/INVS/TPR/STAG1/BIRC6/KIF23/SPICE1/CEP128/IQCB1/CNTRL/BOD1L1/NR3C1/ALMS1/PTP4A1/ATM/TBCK/CDK1/POLB/TOPORS/TAF1D/EFHC1/PHLPP2/RACGAP1/SPATA5/CEP44/PRC1/CENPF/CEP170/PARP4/YPEL5/PKP4/TPX2/SEPTIN2/CKAP2/KIF15/FRY/KIF20A/IRAG2/KNTC1/CENPE/SBDS/PAFAH1B1/KIF20B/KIF18A/CBX1/EVI5/KIFAP3/FAM83D/KIF2A/CEP350/BEX4/TERF1/ZNF207/TOGARAM1/WAPL/DIAPH1/SPECC1L/NIN/HAUS6/SEPTIN7/ASPM/RAD21/TPT1/MICAL3/HAUS3/MID1/BRCC3/HSPB1/BOD1/CUL3/NEK7/NUSAP1/SGO1/DLGAP5/KIF14/TTC28/PKHD1/CKAP2L/SMC3/NCOR1/EPB41/LATS1/EFHC2/SPAG5/PPP2CA/LSM14A/CDC14B/KATNAL1/KATNA1/NEDD1/DCDC2/SHCBP1/XIAP/CDK5RAP2/KIF11/CDC16/MAD2L1/ODF2/NUMA1/TBL1X/CKAP5/NSUN2 |
| GOBP_GLUCOSE_6_PHOSPHATE_METABOLIC_PROCESS | -0.648297702 | -1.86250503 | 0.002178432 | 0.04392312 | GPI/TIGAR/G6PC2/TP53/DERA/PGAM1/PGLS/H6PD/TKT/G6PC3/PGD/G6PD/HK3/TALDO1 |
| GOBP_POSITIVE_REGULATION_OF_DEFENSE_RESPONSE | -0.311256352 | -1.347288508 | 0.002192365 | 0.044073646 | ADAM8/SH2D1B/PSMD9/RPS19/PQBP1/PSME3/PSMA3/PSMB4/TNF/CD1D/PSMB5/POLR3B/ZCCHC3/IL16/TNFRSF1A/VAMP8/GPSM3/PSMC3/GRN/NOD2/MUCL1/IKBKE/HLA-E/MIR126/CEBPA/PSMC4/PSMD11/CYBA/PSMB1/PSMD8/PSMD3/VAV1/HRAS/PSMA7/RELA/FPR2/PRKACA/SYK/STING1/PSMB7/PRKCD/LYN/LAMP1/NMI/CEBPB/MUC16/TLR2/MUC20/MAVS/PSMB3/ICAM3/FFAR2/MAPK13/SIGLEC16/MGST2/PYCARD/ICAM2/TLR4/ARG1/FCN1/HYAL2/NLRP12/DDT/MUC21/TREM2/MNDA/IL18RAP/CCR2/NUPR1/NLRC4/GPR4/LILRA5/MUC4/MMP8/IL1RL1/FCER1G/LILRA2/AGTR1/PLA2G7/CLEC4E/MUC15/TLR8/MUC5B/HCK/S100A8/S100A9/S100A12 |
| GOBP_T_CELL_RECEPTOR_SIGNALING_PATHWAY | 0.405737024 | 1.511407105 | 0.002207489 | 0.044247179 | THY1/ITK/PDE4D/TRAT1/GBP1/SH2D1A/PRKCQ/THEMIS/HLA-DQA1/CTLA4/HLA-DPB1/WNK1/RIPK2/DENND1B/PDE4B/BTN3A1/CBLB/NFKBIZ/CUL1/CD226/MALT1/PSME4/TEC/TNFRSF21/NFKB1/MAP3K7/PTPN22/FBXW11/HLA-DRA/HLA-DPA1/BTRC/PIK3CA/SKP1/BTN3A3/HLA-DQA2/CD28/RC3H1/CD3G/HLA-DRB1/RFTN1/RC3H2/HLA-DRB3/HLA-DQB2/PTPN2/GRAP2/PAWR/HLA-DQB1/TXK/EZR |
| GOBP_CELLULAR_RESPONSE_TO_EXTERNAL_STIMULUS | 0.364538652 | 1.413478114 | 0.002236235 | 0.044691917 | POSTN/SFRP2/VCAM1/ITGA2/PIEZO2/ANKRD1/BMP6/ATP2B1/MMP7/NR4A2/PHEX/LPL/WRN/FOS/ITGA4/LRRK2/GJA1/PDE2A/SESN1/COL1A1/SIK2/MFSD2A/MAPK8/PRKD1/SESN3/SFRP1/TNKS/DNM1L/PIK3C3/SNAI2/IFI16/EIF2AK2/EIF2AK3/NFKB1/ATF3/MYOCD/PDK4/PPARG/HNRNPA1/BCL2/PRKAA2/RRAGB/BMT2/NFE2L2/WDR59/SIRT1/TNFRSF10B/MAP3K2/RNF152/GADD45A/JUN/MAP3K5/LARS1/SLC38A2/FNIP1/TRIM24/ICAM1 |
| GOBP_REPRODUCTIVE_SYSTEM_DEVELOPMENT | 0.347276852 | 1.390537305 | 0.002252136 | 0.04483573 | SFRP2/INHBA/KIT/SULF1/BMP6/MME/SERPINE2/ROBO2/FGFR2/BMP5/ARID5B/ZFPM2/ITGB8/PLEKHA5/RSPO3/HIF1A/TIPARP/KDM5B/ATRX/C3/FRS2/CCN1/WDR48/NRIP1/GJA1/ARID4A/NIPBL/FST/DCN/SFRP1/PLEKHA1/PKD2/ACVR2A/MSH2/BIRC2/SLIT3/BCL2L11/LRP6/LEF1/SMARCC1/MYOCD/BIRC6/ZFX/RBP4/CHD7/HTRA1/PPARG/BCL2/ATM/BPTF/UBB/SIRT1/SETD2/LRP2/VEGFA/NUP107/KDM5A/ANXA1/TMF1/JUNB/RHOBTB3/ZNF568/ICAM1/DLG1/TGFB2/TPPP3/ASH1L/IGF2/ING2/WDR19/FGF10/HSP90AB1/KIF18A/CCND1/LGR4/STK3/CRIP1/VASH1/UBE3A/SEMA3A/FEM1B/GGNBP2/NASP/SYCP2/LIF/ADAM19/VASH2/ADCYAP1/EPOR/FBXW8/PTK2/RBPJ/IMMP2L/SMAD4/CASP3/MMP19/SERPINF1/ADAM20/RRM1/RDH10/NCOA1/TMED2/ZNF830 |
| GOBP_NEGATIVE_REGULATION_OF_APOPTOTIC_SIGNALING_PATHWAY | 0.391812642 | 1.474624157 | 0.002256589 | 0.04483573 | SFRP2/IGF1/CXCL12/HGF/BMP5/TNFAIP3/EYA4/NR4A2/MIR21/HIF1A/THBS1/PHIP/LRRK2/ITGAV/DNAJA1/SERPINE1/HSPA1B/HSPA1A/USP47/RB1CC1/ZMYND11/IVNS1ABP/SNAI2/SH3RF1/MCL1/BIRC6/MARCHF7/RPS6KB1/FGG/BCL2/NFE2L2/CD74/SIRT1/TNFRSF10B/ITGA6/YAP1/CD44/URI1/IL7/ICAM1/SELENOS/HERPUD1/DDX3X/ING2/FGF10/FAS/OPA1/KDM1A/SOD2/ACVR1/PF4/FYN/CX3CL1/FAIM2/TPT1 |
| GOBP_TELENCEPHALON_DEVELOPMENT | 0.386477393 | 1.462149695 | 0.002332979 | 0.046025407 | INHBA/CXCL12/ROBO2/PLCB1/CDON/HIF1A/COL3A1/ROBO1/LRRK2/CCDC141/CXCR4/NF1/FAT4/FOXP2/UBA6/ERBB4/MFSD2A/CDK6/ZSWIM6/BTG2/NFIB/CDH2/TNR/LRP6/LEF1/BBS2/CHD7/DIXDC1/CEP120/SRGAP2C/ATP2B4/ALDH1A3/EZH2/EFHC1/LPAR1/LAMB1/RYK/RELN/SLC38A2/GNG12/KIF5B/TRA2B/PAFAH1B1/PLXNA4/MPP5/RPGRIP1L/MCPH1/KDM1A/SEMA3A/NIN/TACC1/PEX13/NOTCH2NLA/SRGAP2/ATP1B2/AVPR1A/KIF14/CASP3/NCOA1/SLC8A1/WNT5A/SYNE2/RARB/GLI3/BBS4/ATP1A2 |
| GOBP_NEUTROPHIL_CHEMOTAXIS | -0.427126959 | -1.586480362 | 0.002336694 | 0.046025407 | RAC2/CCL2/MPP1/BST1/PIK3CD/CCL23/C5AR1/ITGB2/PREX1/FCER1G/RIPOR2/C3AR1/CXCR2/CXCR1/CSF3R/S100A8/SAA1/S100A9/S100A12 |
| GOBP_INNER_MITOCHONDRIAL_MEMBRANE_ORGANIZATION | -0.524269425 | -1.76064426 | 0.002336726 | 0.046025407 | ATP5F1A/MICOS10/UQCC3/NDUFA13/TIMM10B/TIMM10/DMAC2L/TIMM13/TIMM29/ATP5F1E/TAZ/DNAJC11/ADCK1/PINK1/GHITM/ATP5F1D/MTX1/ROMO1/CHCHD10/ATP5PF/APOO/ATP5ME/ATP5PO/ATP5PD/ATP5MC1/ATP5MF/TIMM8B |
| GOBP_MITOCHONDRIAL_MEMBRANE_ORGANIZATION | -0.38768856 | -1.536376218 | 0.002353951 | 0.046231051 | ATP5F1A/MICOS10/SLC25A5/BID/UQCC3/NDUFA13/TIMM10B/ZNF205/NAIF1/BBC3/TIMM10/DMAC2L/E2F1/TIMM13/TIMM50/BAX/TIMM29/OXA1L/ATP5F1E/TP53/BLOC1S2/BNIP3/NOL3/TAZ/TP63/DNAJC11/COX18/TMEM102/MUL1/ADCK1/CNP/PINK1/YWHAE/MPV17L/GHITM/ATP5F1D/MTX1/ROMO1/HEBP2/MFN2/TOMM22/CHCHD10/GSK3A/ATP5PF/APOO/BAK1/NMT1/ATP5ME/ATP5PO/ATP5PD/BCS1L/ATP5MC1/STPG1/CALM3/MIR29C/ATP5MF/TIMM8B/SLC35F6/BCL2L1/SFN |
| GOBP_INTERLEUKIN_8_PRODUCTION | -0.439440531 | -1.629890003 | 0.002362231 | 0.046260363 | CD244/C5AR2/RELA/TLR5/SYK/CD33/ARRB1/TLR2/MAVS/BCL3/FFAR2/PYCARD/MYD88/TLR4/FCN1/HYAL2/TLR6/TLR1/KLF4/CLEC7A/LILRA2/TLR8/CD14 |
| GOBP_INTERLEUKIN_1_PRODUCTION | -0.409547485 | -1.583339335 | 0.002375465 | 0.046386231 | CD33/CX3CR1/TRIM16/GIT1/ORM1/PYCARD/MEFV/TLR4/SIRPA/NLRP12/TMEM106A/TLR6/CEACAM1/TREM2/MNDA/CLEC7A/NLRC4/LILRB4/LILRA5/LILRA2/ARRB2/TLR8/SAA1/IL1R2 |
| GOBP_CELLULAR_RESPONSE_TO_VITAMIN | 0.632705107 | 1.770993565 | 0.002382466 | 0.046390026 | POSTN/ATP2B1/PHEX/COL1A1/SFRP1/SNAI2/PPARG |
| GOBP_BONE_MINERALIZATION | 0.458889237 | 1.618836527 | 0.002410289 | 0.046756736 | ASPN/IGF1/BMP6/FGFR2/ATP2B1/ADGRV1/TGFB3/TXLNG/HIF1A/PHEX/GPC3/CCN1/DDR2/MGP/BMPR1A/LOX/ACVR2A/COL1A2/SMAD3/EIF2AK3/MEF2C/LTBP3 |
| GOMF_STEROID_DEHYDROGENASE_ACTIVITY | -0.594048395 | -1.781214418 | 0.002420283 | 0.046756736 | HSD3B1/HSD17B11/HSD11B2/HSD17B4/HSD17B12/AKR1C1/HSD17B13/HSD17B2/SRD5A1/DHRS9/SRD5A2/HSD17B10/HSD17B14/NSDHL/SRD5A3/AKR1C3/AKR1C2 |
| GOMF_3_5_CYCLIC_GMP_PHOSPHODIESTERASE_ACTIVITY | 0.765321715 | 1.806538115 | 0.002421882 | 0.046756736 | PDE1A/PDE8A/PDE5A/PDE10A/PDE2A |
| GOBP_MUSCLE_CELL_PROLIFERATION | 0.392844536 | 1.475286919 | 0.002432945 | 0.046837636 | OGN/ITGA2/PDE1A/IGF1/PDGFD/EDN1/FGFR2/TNFAIP3/PDCD4/ZFPM2/CCL5/TGFB3/NR4A3/MIR21/THBS1/ARID2/CTNNB1/GJA1/APLN/BMPR1A/PRKG1/ERBB4/IGFBP3/HBEGF/STAT1/CNN1/MEF2C/PAXBP1/MYOCD/MNAT1/RBP4/PPARG/RPS6KB1/PPARGC1A/MIR27A/ABCC4/ANGPT1/EFEMP2/JUN/YAP1/FGF2/FOXP1/ERN1/MAP3K5 |
| GOBP_REGULATION_OF_MAST_CELL_ACTIVATION_INVOLVED_IN_IMMUNE_RESPONSE | -0.61853277 | -1.845051767 | 0.002441845 | 0.046876545 | VAMP8/FES/LGALS9/SPHK2/SYK/UNC13D/LYN/RAC2/IL4R/CD300A/STXBP2/ADGRE2/FOXF1/GATA2/FGR/HMOX1 |
| GOMF_DOUBLE_STRANDED_RNA_BINDING | 0.491441461 | 1.630317703 | 0.002451803 | 0.046935507 | HNRNPU/DDX58/DDX60/VIM/STRBP/TFRC/DHX36/IFIH1/EIF2AK2/FMR1/AGO3/MBNL1/DHX15/FXR1/SUPV3L1/RC3H1/RFTN1/DHX9/AGO2/RC3H2/DDX3X/STAU2/HSP90AB1/HSPD1/DICER1/LRRFIP1/OASL/ACTN1/HMGB1/DDX21/EIF4B/OAS2/DROSHA/LSM14A |
| GOBP_PEPTIDYL_TYROSINE_MODIFICATION | 0.346434778 | 1.373763771 | 0.002502525 | 0.047772297 | SFRP2/ENPP2/THY1/IGF1/KIT/PDGFD/AREG/BMP6/EPHA3/ITK/ITGB3/WEE1/FGFR2/HGF/CNTN1/EPHA4/CCL5/ERRFI1/FGF7/FER/DDR2/CLK1/FBXW7/TTN/RIPK2/ROS1/ERBB4/SFRP1/HBEGF/BMX/CBLB/MET/ERCC6/SOCS5/MUSK/TEC/EIF2AK2/ABL2/PDGFA/NEK1/EPGN/RICTOR/INSR/DYRK3/HIPK3/LRP4/CD80/CD74/CLK4/ANGPT1/VEGFA/CD44/RYK/RELN/IL7/AFAP1L2/STAP1/ICAM1/PIBF1/PTPN2/IGF2/FGFR1/EFEMP1/NRP1/FGF10/HTR2A/TXK/EFNA1/PARP14/DLG3/ADIPOQ/ACVR1/CSF3/FYN/INPP5F/GGNBP2/MAP2K4/THBS4/LIF/DYRK2/GREM1/PDGFC/PTPRC/ABI2/DYRK1A/CEP43/PTK2/PRLR |
| GOBP_REGULATION_OF_NERVOUS_SYSTEM_DEVELOPMENT | 0.345746475 | 1.388318128 | 0.002548314 | 0.04851051 | THY1/KIT/SEMA3D/MME/CXCL12/SERPINE2/ROBO2/HGF/TRPC6/EPHA4/ETV5/RUFY3/MACF1/SEMA3C/PTPRD/FLRT2/HIF1A/ROBO1/MAP3K13/CXCR4/CAPRIN2/CTNNB1/NF1/PCM1/BMPR1A/ASPA/SEMA6D/NR1D1/RAPGEF2/PRKCI/NPHP3/CLSTN2/PER2/TNR/ELAPOR2/DNM1L/MAN2A1/DOCK7/DHX36/SKIL/EIF2AK3/TNFRSF21/IL1RAP/CHD7/SEMA5A/PPARG/LRRTM2/TMEM98/SLITRK6/GOLGA4/DCC/LRP4/EZH2/LRP2/FLRT3/VEGFA/YAP1/RYK/PPP3CA/RELN/MAP1B/SPEN/PRKCH/FN1/FBXO31/PRTG/HOOK3/DLG1/NLGN1/SLC25A12/NRP1/OPA1/PLXNA4/DICER1/KDM1A/UFL1/FZD3/BHLHB9/SEMA3A/BHLHE40/NIN/DLG5/CX3CL1/LIF/ADCYAP1/PARD3/AMIGO2/SPART/FBXW8/REST/APPL2/MDK/MIR222/MYB/PAK3/KIF14/DUSP10/SERPINF1/IST1/WNT5A/SNW1/DBN1/EIF4G2/IFNG/TIAM1 |
| GOBP_RESPONSE_TO_INTERLEUKIN_1 | 0.405041957 | 1.519445188 | 0.002562895 | 0.048652175 | HAS2/SELE/CCL21/ANKRD1/EGR1/EDN1/PLCB1/RORA/CCL5/GBP1/RPS6KA5/MIR21/HIF1A/CD38/PELI1/GBP3/CCL18/AKAP12/RIPK2/NR1D1/SFRP1/PRKCI/CUL1/PSME4/ADAMTS12/NFKB1/IL1RAP/MAP3K7/FBXW11/BTRC/SKP1/GBP2/MIR27A/YTHDC2/TANK/OTUD4/RC3H1/UBB/CD47/VRK2/ANXA1/ICAM1/CCL19/NOD1/TAB3 |
| KEGG |  |  |  |  |  |
| KEGG_OXIDATIVE_PHOSPHORYLATION | -0.649220863 | -2.488805597 | 1.00E-10 | 1.85E-08 | ATP6V1E1/ATP6V1G1/COX7C/ATP5F1A/NDUFB1/COX11/ATP6V0E1/NDUFA1/NDUFV2/UQCRHL/NDUFS7/ATP6V0C/ATP6V0E2/UQCRFS1/COX10/UQCRC1/NDUFC2/COX8A/COX4I1/ATP6V1C2/ATP6V1A/NDUFA7/NDUFA4/ATP5F1E/NDUFA4L2/SDHC/COX7A2L/ATP6V1B2/NDUFS8/COX17/NDUFAB1/NDUFA6/UQCR10/NDUFB6/SDHD/NDUFV3/UQCRQ/ATP5F1D/COX7A2/UQCR11/COX7A1/SDHB/NDUFB4/TCIRG1/ATP12A/NDUFB9/ATP5PF/ATP6V0D2/NDUFS2/ATP5ME/CYC1/ATP5PO/NDUFA2/ATP5PD/NDUFB3/NDUFB7/ATP5MC1/NDUFV1/ATP6V0B/COX4I2/COX5B/ATP5MF/ATP6V0D1/ATP6AP1/COX5A/ATP6V1F/COX7B/NDUFB10/COX6B1 |
| KEGG_LYSOSOME | -0.537997069 | -2.073784752 | 6.24E-08 | 5.77E-06 | GUSB/M6PR/ARSA/HEXA/SORT1/LIPA/GNPTG/FUCA1/AP1M1/GLB1/PLA2G15/SGSH/SLC17A5/GALNS/AP1S1/NAGLU/AP3D1/TCIRG1/GLA/CTSL/ATP6V0D2/NAGPA/GGA1/DNASE2/CTSD/MAN2B1/CLTA/LAPTM5/LAMP1/ATP6V0B/GGA3/AP1M2/CTSA/ACP5/DNASE2B/ATP6V0D1/ATP6AP1/CD68/CTSB/NEU1/CLTB/TPP1/GM2A/MCOLN1/CLN3/NAGA/SLC11A1/AP1B1/SMPD1/GBA/ACP2/CTSE/CTNS/HYAL1 |
| KEGG_FOCAL_ADHESION | 0.490144754 | 1.847991672 | 1.05E-06 | 6.48E-05 | ITGA2/IGF1/PDGFD/COL6A6/ITGB3/HGF/BIRC3/ITGB6/COL6A3/THBS2/ITGB8/ITGA9/THBS1/COL3A1/ITGA4/CTNNB1/ITGAV/LAMA3/COL1A1/COL5A2/MAPK10/TNXB/SHC3/MAPK8/SOS1/DOCK1/FLNB/BIRC2/MET/COL1A2/TNR/PPP1R12A/FLNC/COL4A1/ARHGAP5/PIK3CG/PDGFA/BCL2/PIK3CA/BRAF/LAMA4/ITGA8/ROCK1/SOS2/ITGA6/RAP1B/CCND2/VEGFA/JUN/LAMB1/VAV2/RELN/RASGRF1/FN1/VCL/ITGB1/MAPK9/CCND1/ROCK2/ITGA7/DIAPH1/COL4A2/ACTN1/FYN |
| KEGG_HUNTINGTONS_DISEASE | -0.462280049 | -1.852346121 | 1.61E-06 | 7.44E-05 | PLCB3/COX7C/ATP5F1A/SLC25A5/DNAL4/TGM2/NDUFB1/DLG4/NDUFA1/POLR2L/DCTN1/NDUFV2/BBC3/UQCRHL/NDUFS7/POLR2F/UQCRFS1/DNAI2/POLR2I/UQCRC1/NDUFC2/COX8A/POLR2K/COX4I1/POLR2C/IFT57/BAX/HIP1/NDUFA7/NDUFA4/ATP5F1E/TP53/NDUFA4L2/SLC25A4/SDHC/COX7A2L/NDUFS8/NDUFAB1/NDUFA6/POLR2J/CREB3/UQCR10/POLR2D/NDUFB6/SDHD/NDUFV3/POLR2G/UQCRQ/ATP5F1D/COX7A2/UQCR11/COX7A1/SDHB/NDUFB4/POLR2A/PLCB2/NDUFB9/ATP5PF/DNAI1/NDUFS2/CYC1/ATP5PO/NDUFA2/CLTA/ATP5PD/NDUFB3/AP2M1/NDUFB7/ATP5MC1/NDUFV1/POLR2E/COX4I2/COX5B/AP2A1/CLTB/COX5A/DNAH3/AP2S1/COX7B/NDUFB10/COX6B1 |
| KEGG_GLUTATHIONE_METABOLISM | -0.665616288 | -2.169715302 | 2.45E-06 | 9.07E-05 | GSS/GCLC/GSTP1/GSTA1/GSTM4/GSTA2/GCLM/IDH2/MGST3/MGST2/GSR/IDH1/GSTT2/MGST1/GSTT1/GPX2/PGD/GSTA4/G6PD/GPX3/ANPEP |
| KEGG_PARKINSONS_DISEASE | -0.497793342 | -1.904798369 | 1.09E-05 | 0.000335373 | COX7C/ATP5F1A/SLC25A5/NDUFB1/NDUFA1/NDUFV2/UQCRHL/NDUFS7/UQCRFS1/UQCRC1/NDUFC2/COX8A/COX4I1/UBE2L3/NDUFA7/NDUFA4/ATP5F1E/NDUFA4L2/SLC25A4/SDHC/UBE2J1/COX7A2L/NDUFS8/NDUFAB1/NDUFA6/PINK1/UQCR10/NDUFB6/SDHD/NDUFV3/UQCRQ/ATP5F1D/COX7A2/UQCR11/COX7A1/SDHB/NDUFB4/UBA1/NDUFB9/ATP5PF/NDUFS2/CYC1/ATP5PO/NDUFA2/ATP5PD/NDUFB3/NDUFB7/ATP5MC1/NDUFV1/UCHL1/COX4I2/COX5B/COX5A/COX7B/NDUFB10/COX6B1 |
| KEGG_ECM_RECEPTOR_INTERACTION | 0.566744101 | 1.917863369 | 2.22E-05 | 0.000587137 | ITGA2/COL6A6/ITGB3/ITGB6/COL6A3/THBS2/ITGB8/ITGA9/THBS1/COL3A1/ITGA4/ITGAV/LAMA3/COL1A1/COL5A2/TNXB/COL1A2/TNR/COL4A1/LAMA4/ITGA8/ITGA6/CD44/LAMB1/CD47/RELN/FN1/SDC4/ITGB1/ITGA7/COL4A2/THBS4/HSPG2 |
| KEGG_ARRHYTHMOGENIC_RIGHT_VENTRICULAR_CARDIOMYOPATHY_ARVC | 0.579626113 | 1.902493129 | 3.02E-05 | 0.000699518 | ITGA2/ITGB3/ITGB6/ITGB8/ITGA9/CACNA2D1/PKP2/ITGA4/RYR2/CTNNB1/ITGAV/GJA1/DSG2/SGCD/CDH2/SGCB/LEF1/CACNA2D2/DMD/ITGA8/CACNB2/ITGA6/ITGB1/LMNA/DES/ITGA7/ACTN1/CACNB4/ACTN2/CACNA1C/CACNB3/SLC8A1/LAMA2 |
| KEGG_WNT_SIGNALING_PATHWAY | 0.474602441 | 1.725104829 | 5.17E-05 | 0.001063016 | SFRP2/WIF1/SFRP4/PLCB1/PLCB4/FZD7/MMP7/CTNNB1/PRKACB/NFATC2/MAPK10/DAAM1/MAPK8/SFRP1/PRICKLE2/CAMK2D/CUL1/TBL1XR1/LRP6/SMAD3/LEF1/MAP3K7/FBXW11/SFRP5/PPP2R1B/BTRC/SKP1/CSNK1A1/ROCK1/CCND2/JUN/PPP3CA/NFAT5/FZD6/SMAD2/MAPK9/CCND1/ROCK2/FZD3 |
| KEGG_AUTOIMMUNE_THYROID_DISEASE | 0.62664699 | 1.96086122 | 0.000116476 | 0.001975861 | HLA-DQA1/CTLA4/HLA-DPB1/HLA-DOA/HLA-DRA/HLA-DPA1/HLA-DQA2/CD80/CD28/HLA-DOB/HLA-DRB1/CD40LG/HLA-DRB3/FAS/HLA-DQB1/HLA-DMA/GZMB/CD86 |
| KEGG_TERPENOID_BACKBONE_BIOSYNTHESIS | -0.806713074 | -2.054303558 | 0.000117484 | 0.001975861 | HMGCR/HMGCS1/MVK/MVD/DHDDS/PDSS1/FDPS/ACAT2 |
| KEGG_CELL_ADHESION_MOLECULES_CAMS | 0.473303114 | 1.704053098 | 0.000186885 | 0.002881136 | VCAM1/SELE/SELP/CNTN1/ITGB8/ITGA9/PDCD1LG2/HLA-DQA1/CTLA4/ITGA4/HLA-DPB1/ITGAV/CLDN18/ICOS/HLA-DOA/NRCAM/CDH2/CD226/NEO1/CD2/CLDN11/HLA-DRA/HLA-DPA1/CADM1/HLA-DQA2/CD80/ITGA8/CD28/HLA-DOB/HLA-DRB1/CD40LG/ITGA6/JAM3/HLA-DRB3/ICAM1/SDC4/ITGB1/NLGN1/NRXN3/HLA-DQB1/NFASC/NECTIN3/HLA-DMA |
| KEGG_ABC_TRANSPORTERS | 0.621905813 | 1.914163715 | 0.000221982 | 0.003158978 | ABCG2/ABCC9/ABCB1/ABCA8/ABCA9/ABCA6/ABCA10/ABCG1/ABCC4/ABCD2/ABCA5/ABCD3/CFTR/ABCB7 |
| KEGG_TYPE_I_DIABETES_MELLITUS | 0.612057628 | 1.863350625 | 0.000369163 | 0.004656178 | HLA-DQA1/CPE/HLA-DPB1/HLA-DOA/HLA-DRA/HLA-DPA1/HLA-DQA2/CD80/CD28/HLA-DOB/HLA-DRB1/HLA-DRB3/FAS/HLA-DQB1/HSPD1/HLA-DMA/GZMB/CD86/IFNG |
| KEGG_TGF_BETA_SIGNALING_PATHWAY | 0.51458649 | 1.753396296 | 0.000393234 | 0.004656178 | LTBP1/INHBA/BMP6/BMP5/THBS2/TGFB3/THBS1/ZFYVE9/BMPR1A/FST/DCN/ID4/SMAD9/SMURF2/ACVR2A/CUL1/SMAD3/ZFYVE16/RPS6KB1/PPP2R1B/SKP1/ROCK1/SMURF1/TGFB2/SMAD2/ROCK2/ACVR1/THBS4/RBL2/SMAD5 |
| KEGG_HYPERTROPHIC_CARDIOMYOPATHY_HCM | 0.515024777 | 1.734321048 | 0.000402696 | 0.004656178 | ITGA2/IGF1/ITGB3/ITGB6/TGFB3/ITGB8/ITGA9/CACNA2D1/ITGA4/RYR2/ITGAV/TTN/SGCD/SGCB/CACNA2D2/DMD/PRKAA2/ITGA8/CACNB2/ITGA6/TGFB2/ITGB1/LMNA/DES/ITGA7 |
| KEGG_METABOLISM_OF_XENOBIOTICS_BY_CYTOCHROME_P450 | -0.533852313 | -1.844442801 | 0.000493778 | 0.005373461 | ADH7/GSTP1/GSTA1/GSTM4/GSTA2/ALDH3B1/MGST3/MGST2/GSTT2/MGST1/GSTT1/ALDH3A1/CYP2F1/AKR1C3/GSTA4/CYP1B1/AKR1C2 |
| KEGG_INTESTINAL_IMMUNE_NETWORK_FOR_IGA_PRODUCTION | 0.589879496 | 1.827136728 | 0.000591035 | 0.006074529 | CXCL12/HLA-DQA1/ITGA4/CXCR4/HLA-DPB1/ICOS/HLA-DOA/TNFSF13B/HLA-DRA/HLA-DPA1/HLA-DQA2/CD80/CD28/HLA-DOB/HLA-DRB1/CD40LG/HLA-DRB3/HLA-DQB1/HLA-DMA |
| KEGG_ALLOGRAFT_REJECTION | 0.633978198 | 1.872283725 | 0.000885037 | 0.008617468 | HLA-DQA1/HLA-DPB1/HLA-DOA/HLA-DRA/HLA-DPA1/HLA-DQA2/CD80/CD28/HLA-DOB/HLA-DRB1/CD40LG/HLA-DRB3/FAS/HLA-DQB1/HLA-DMA/GZMB/CD86/IFNG |
| KEGG_VIRAL_MYOCARDITIS | 0.524885318 | 1.71452675 | 0.001073157 | 0.009726149 | MYH10/HLA-DQA1/HLA-DPB1/MYH11/SGCD/HLA-DOA/CXADR/SGCB/ABL2/HLA-DRA/DMD/HLA-DPA1/HLA-DQA2/CD80/CD28/HLA-DOB/HLA-DRB1/CD40LG/HLA-DRB3/ICAM1/HLA-DQB1/CCND1/HLA-DMA/FYN/CASP3/ABL1/CD86/EIF4G2/LAMA2 |
| KEGG_PATHWAYS_IN_CANCER | 0.363145568 | 1.437073457 | 0.001109621 | 0.009726149 | ITGA2/IGF1/KIT/PTGS2/FGFR2/HGF/BIRC3/FZD7/TGFB3/HIF1A/FGF7/FOS/CTNNB1/ITGAV/LAMA3/TRAF5/MAPK10/MAPK8/HSP90AA1/SOS1/STAT1/FGF14/CDK6/CBLB/PLD1/MECOM/MSH2/BIRC2/MET/SMAD3/NFKB1/LEF1/HHIP/TPR/PPARG/COL4A1/PIK3CG/PDGFA/FGF1/BCL2/PIK3CA/BRAF/LAMA4/DCC/MSH6/MITF/SOS2/ITGA6/VEGFA/JUN/LAMB1/FGF2/RALA/MSH3/FN1/TRAF3/TGFB2/ITGB1/FGFR1/FZD6/SMAD2/FGF10/DAPK2/FAS/HSP90AB1/MAPK9/CCND1/CUL2/APPL1/NFKBIA/FZD3/COL4A2/ETS1/CCNE2/PIAS2/COL4A4/TRAF6/PTK2/APC/SMAD4/CASP3/AKT3/TRAF1/ABL1/MMP2/ARNT2/JAK1/WNT5A/LAMA1 |
| KEGG_STEROID_BIOSYNTHESIS | -0.750152044 | -1.946090173 | 0.001156623 | 0.009726149 | LIPA/SC5D/FDFT1/MSMO1/SOAT1/NSDHL/TM7SF2/DHCR7/LSS/EBP |
| KEGG_GLYCOLYSIS_GLUCONEOGENESIS | -0.488258958 | -1.688130641 | 0.00219289 | 0.016968204 | ADH1C/GPI/LDHA/ADH1A/PGAM2/ALDH3B2/G6PC2/PDHB/PGM1/TPI1/PGAM4/ALDOA/ENO2/PGAM1/ADH7/DLAT/AKR1A1/ENO1/ALDH3B1/PCK2/GAPDH/PKM/ALDOC/ALDH3A1/HK3/ACSS2 |
| KEGG_COLORECTAL_CANCER | 0.516547112 | 1.65028036 | 0.002201281 | 0.016968204 | TGFB3/FOS/CTNNB1/MAPK10/MAPK8/MSH2/SMAD3/LEF1/PIK3CG/BCL2/PIK3CA/BRAF/DCC/MSH6/JUN/MSH3/TGFB2/SMAD2/MAPK9/CCND1/APPL1/APC/SMAD4/CASP3/AKT3 |
| KEGG_DILATED_CARDIOMYOPATHY | 0.475772439 | 1.623523019 | 0.002747079 | 0.019940528 | ITGA2/IGF1/ITGB3/ITGB6/TGFB3/ITGB8/ITGA9/CACNA2D1/ITGA4/RYR2/ITGAV/PRKACB/TTN/SGCD/SGCB/CACNA2D2/DMD/ITGA8/CACNB2/ITGA6/TGFB2/ITGB1/LMNA/DES/ITGA7/ADCY6 |
| KEGG_GRAFT_VERSUS_HOST_DISEASE | 0.586142911 | 1.733279286 | 0.002838359 | 0.019940528 | HLA-DQA1/HLA-DPB1/HLA-DOA/HLA-DRA/HLA-DPA1/HLA-DQA2/CD80/CD28/HLA-DOB/HLA-DRB1/HLA-DRB3/FAS/HLA-DQB1/HLA-DMA/GZMB/CD86/IFNG |
| KEGG_ALZHEIMERS_DISEASE | -0.379512961 | -1.501368393 | 0.002910239 | 0.019940528 | PLCB3/CHP2/COX7C/ATP5F1A/BID/NDUFB1/NDUFA1/NDUFV2/UQCRHL/NDUFS7/UQCRFS1/UQCRC1/NDUFC2/FADD/COX8A/COX4I1/NDUFA7/NDUFA4/ATP5F1E/TNF/APOE/TNFRSF1A/NDUFA4L2/APBB1/SDHC/COX7A2L/NDUFS8/NDUFAB1/NDUFA6/ATP2A3/UQCR10/NDUFB6/SDHD/NDUFV3/UQCRQ/ATP5F1D/COX7A2/UQCR11/COX7A1/SDHB/NDUFB4/PLCB2/NDUFB9/ATP5PF/NDUFS2/CYC1/ATP5PO/NDUFA2/NCSTN/ATP5PD/NDUFB3/NDUFB7/ATP5MC1/NDUFV1/HSD17B10/CDK5/CALM3/COX4I2/PSENEN/COX5B/GAPDH/COX5A/PSEN2/COX7B/NDUFB10/CAPN1/COX6B1 |
| KEGG_NEUROACTIVE_LIGAND_RECEPTOR_INTERACTION | -0.322721122 | -1.375067651 | 0.003323401 | 0.021958187 | GABRP/S1PR4/ADORA2A/NMUR1/PTAFR/FPR2/P2RY1/AGTR2/ADRB1/P2RX4/PTH1R/RXFP1/CHRM1/HRH2/FPR3/P2RY13/F2RL3/C5AR1/ADRA1B/GRIA1/S1PR1/EDNRB/AGTR1/C3AR1/FPR1/ADORA3/ADRA1A/VIPR1 |
| KEGG_CITRATE_CYCLE_TCA_CYCLE | -0.600444113 | -1.815206414 | 0.003457777 | 0.022058234 | PDHB/MDH1/IDH3B/ACO2/SDHC/SDHD/ACLY/SDHB/IDH3G/DLAT/OGDH/IDH2/PCK2/MDH2/IDH1 |
| KEGG_GALACTOSE_METABOLISM | -0.591437254 | -1.729234686 | 0.005106801 | 0.03149194 | AKR1B1/G6PC2/PGM1/B4GALT2/GLB1/GALK1/GLA/GALE/HK3/MGAM |
| KEGG_PRION_DISEASES | 0.586330844 | 1.72111118 | 0.00560118 | 0.033426399 | EGR1/C5/C7/CCL5/C8B/PRKACB/HSPA1A |
| KEGG_MELANOMA | 0.476557047 | 1.56000827 | 0.005910475 | 0.034169932 | IGF1/PDGFD/HGF/FGF7/FGF14/CDK6/MET/PIK3CG/PDGFA/FGF1/PIK3CA/BRAF/MITF/FGF2/FGFR1/FGF10/CCND1 |
| KEGG_T_CELL_RECEPTOR_SIGNALING_PATHWAY | 0.441441174 | 1.550399162 | 0.006314819 | 0.035401256 | RASGRP1/ITK/PRKCQ/FOS/CTLA4/ICOS/NFATC2/SOS1/CBLB/MALT1/TEC/NFKB1/MAP3K7/PIK3CG/PIK3CA/CD28/CD3G/SOS2/CD40LG/JUN/PPP3CA/VAV2/NFAT5/DLG1/GRAP2/MAPK9/NFKBIA/FYN/NCK1/PTPRC/PAK3/AKT3 |
| KEGG_MAPK_SIGNALING_PATHWAY | 0.358122295 | 1.385610185 | 0.006528609 | 0.035523313 | RASGRP1/FGFR2/TGFB3/RASGRF2/RPS6KA5/FGF7/CACNA2D1/FOS/MAP3K13/MAP3K4/NF1/PRKACB/NFATC2/MAPK10/RASGRP3/HSPA1B/HSPA1A/MAP4K4/MAPK8/RAPGEF2/SOS1/RASA2/FGF14/TAOK1/PLA2G4A/FLNB/MECOM/RPS6KA6/RASA1/DUSP16/NR4A1/MAP3K20/RPS6KA3/FLNC/NFKB1/MEF2C/MAP3K7/CACNA2D2/PPM1B/PDGFA/FGF1/BRAF/SOS2/MAP3K2/PLA2G2A/CACNB2/GADD45A/RAP1B/JUN/FGF2/PPP3CA/MAP3K5/GNG12/RASGRF1/TGFB2/PLA2G1B/DUSP1/ATF2/FGFR1/FGF10/FAS/HSPA6/MAPK9/STK3/MAP3K1 |
| KEGG_AMINO_SUGAR_AND_NUCLEOTIDE_SUGAR_METABOLISM | -0.514122644 | -1.650427363 | 0.009437325 | 0.049883002 | PGM1/CYB5R3/HEXA/MPI/AMDHD2/GALK1/CYB5R1/GFUS/GALE/NANS/CHIA/NAGK/GMPPA/RENBP/GNPDA1/HK3/NPL/CHIT1 |
