# Supplementary material for: FAM171B as a Novel Biomarker Mediates Tissue Immune Microenvironment in Pulmonary Arterial Hypertension
Source: Mediators Inflamm. 2022 Sep 22;2022:1878766. doi: 10.1155/2022/1878766 (PMC9553458; doi:10.1155/2022/1878766)
Supplement: Supplementary Materials — Supplementary Table 1: The results of differentially expressed genes (DEGs). Supplementary Table 2: Gene Ontology (GO) enrichment analysis results of differentially expressed genes (DEGs). Supplementary Table 3: Kyoto Encyclopedia of Genes and Genomes (KEGG) enrichment analysis results of differentially expressed genes (DEGs). Supplementary Table 4: Disease Ontology (DO) enrichment analysis results of differentially expressed genes (DEGs). Supplementary Table 5: Metascape function analysis results of differentially expressed genes (DEGs). Supplementary Table 6: results of Gene Set Enrichment Analysis (GSEA) of gene expression matrix. Supplementary Table 7: results of all genes in brown module. Supplementary Table 8: results of key genes in brown module. Supplementary Table 9: results of analyzing the combined data matrix of GSE113439 and GSE117261 using CIBERSORT. Supplementary Table 10: results of the correlation of FAM171B with immune cells. [file 1878766.f1.zip › Supplementary Table7.docx]

| genes |
| --- |
| ABCA10 |
| ABCA5 |
| ABCA8 |
| ABCC9 |
| ABI3BP |
| ABLIM1 |
| ABLIM3 |
| ACADL |
| ACE2 |
| ACOXL |
| ACP2 |
| ACP5 |
| ACSS2 |
| ACSS3 |
| ACTA2 |
| ACTR3C |
| ADA2 |
| ADAM15 |
| ADAMTS12 |
| ADAMTS5 |
| ADAMTS6 |
| ADAMTSL3 |
| ADCY7 |
| ADD3 |
| ADGRB3 |
| ADGRE5 |
| ADGRF5 |
| ADGRG1 |
| ADGRG6 |
| ADGRL2 |
| ADGRV1 |
| AFAP1L1 |
| AFAP1L2 |
| AGBL1 |
| AGTRAP |
| AHCTF1 |
| AHCYL2 |
| AHI1 |
| AKAP11 |
| AKAP9 |
| ALAS2 |
| ALCAM |
| ALDOC |
| ALG3 |
| ALOX5 |
| ALOX5AP |
| ALPL |
| ALYREF |
| AMIGO2 |
| AMY2B |
| ANGPT1 |
| ANK3 |
| ANKMY2 |
| ANKRD1 |
| ANKRD12 |
| ANKRD20A12P |
| ANKRD20A3P |
| ANKRD20A8P |
| ANKRD36 |
| ANKRD36B |
| ANKRD36C |
| ANKRD44 |
| ANKRD50 |
| ANO5 |
| ANTXR1 |
| ANXA6 |
| AP1B1 |
| AP2S1 |
| APC |
| APOE |
| ARAP1 |
| ARAP2 |
| ARFGEF3 |
| ARGLU1 |
| ARHGAP12 |
| ARHGAP18 |
| ARHGAP21 |
| ARHGAP28 |
| ARHGAP29 |
| ARHGAP30 |
| ARHGAP42 |
| ARHGEF28 |
| ARHGEF38 |
| ARHGEF6 |
| ARID4A |
| ARL6 |
| ARMCX1 |
| ARMCX4 |
| ARNTL |
| ARRB2 |
| AS3MT |
| ASPA |
| ASPN |
| ATP13A4 |
| ATP5MC1 |
| ATP6AP1L |
| ATP6V0D1 |
| ATRX |
| B3GALT2 |
| B3GAT3 |
| BAK1 |
| BAZ2B |
| BBS2 |
| BCHE |
| BDH2 |
| BHLHE41 |
| BICC1 |
| BICD1 |
| BMP5 |
| BMPR1A |
| BMPR2 |
| BNC2 |
| BOD1L1 |
| BRWD1 |
| BTAF1 |
| BTBD8 |
| C11orf24 |
| C1QB |
| C1QC |
| C1QTNF3 |
| C1orf162 |
| C1orf54 |
| C21orf91 |
| C4B |
| C4BPA |
| C5 |
| C5AR1 |
| CA1 |
| CACNA2D1 |
| CADPS2 |
| CALHM5 |
| CAMSAP2 |
| CAP2 |
| CAPG |
| CAPN3 |
| CAPN7 |
| CBS |
| CCDC14 |
| CCDC144CP |
| CCDC144NL |
| CCDC171 |
| CCDC68 |
| CCDC82 |
| CCL3 |
| CCL3L3 |
| CCL4 |
| CCL4L2 |
| CCN3 |
| CCRL2 |
| CD101 |
| CD14 |
| CD226 |
| CD300A |
| CD300C |
| CD300LF |
| CD33 |
| CD36 |
| CD68 |
| CD82 |
| CDC25B |
| CDC42BPA |
| CDCA7L |
| CDH6 |
| CDK15 |
| CDK6 |
| CDKL2 |
| CDON |
| CEACAM6 |
| CEBPB |
| CENPC |
| CENPJ |
| CEP112 |
| CEP192 |
| CEP295 |
| CEP57 |
| CFH |
| CFLAR |
| CHD6 |
| CHIT1 |
| CHN1 |
| CHRM3 |
| CIART |
| CLCN7 |
| CLEC1A |
| CLK1 |
| CLK4 |
| CLN6 |
| CMKLR1 |
| CNST |
| CNTLN |
| CNTN1 |
| CNTN4 |
| CNTN6 |
| CNTNAP3B |
| COL12A1 |
| COL6A1 |
| COLEC10 |
| COLEC12 |
| COLGALT1 |
| COPE |
| CORIN |
| COX7B |
| CPED1 |
| CPLANE1 |
| CRBN |
| CREBRF |
| CRYBG3 |
| CSF1R |
| CSGALNACT1 |
| CSRP2 |
| CSTA |
| CTAGE15 |
| CTNNAL1 |
| CTSD |
| CTSK |
| CWF19L2 |
| CXADR |
| CXCL16 |
| CYLD |
| CYP27A1 |
| CYP39A1 |
| CYSLTR2 |
| CYTH4 |
| DCBLD1 |
| DCBLD2 |
| DCN |
| DDR2 |
| DENND1B |
| DENND2C |
| DENND2D |
| DESI2 |
| DHCR7 |
| DIAPH2 |
| DIXDC1 |
| DLC1 |
| DLG2 |
| DMAC2 |
| DMD |
| DMTF1 |
| DNAJB4 |
| DNM1P46 |
| DNM3 |
| DOCK4 |
| DOK2 |
| DOP1A |
| DPH6 |
| DPYSL3 |
| DST |
| DUSP7 |
| DYNC2LI1 |
| EBP |
| ECM2 |
| EDIL3 |
| EFCAB13 |
| EFHD2 |
| EHBP1 |
| ELAPOR2 |
| EMILIN1 |
| EMP3 |
| ENPEP |
| ENPP1 |
| ENPP2 |
| ENTPD1 |
| EPB41L2 |
| EPB41L4A |
| EPCAM |
| EPHA3 |
| EPHA4 |
| ERBB4 |
| ERCC6 |
| ERG |
| ERO1B |
| ERV3-1 |
| ESM1 |
| ESRP1 |
| EXOC6 |
| EXOC6B |
| EXPH5 |
| EYA4 |
| F11 |
| F8 |
| FABP4 |
| FAM111A |
| FAM13C |
| FAM167B |
| FAM171B |
| FAM184A |
| FAM214A |
| FASN |
| FAT3 |
| FAT4 |
| FBN1 |
| FBXW7 |
| FCER1G |
| FER |
| FERMT3 |
| FEZ1 |
| FFAR4 |
| FILIP1 |
| FLRT2 |
| FMNL1 |
| FOXP2 |
| FRAS1 |
| FREM1 |
| FREM2 |
| FRMD4B |
| FRY |
| FUCA1 |
| FURIN |
| G6PC3 |
| G6PD |
| GALNT5 |
| GBA |
| GBGT1 |
| GCC2 |
| GCNT2 |
| GFUS |
| GIMAP7 |
| GIPC2 |
| GJC1 |
| GLIPR2 |
| GLMP |
| GM2A |
| GMFG |
| GNAI1 |
| GOLGA8B |
| GOLGB1 |
| GPC6 |
| GPR160 |
| GPR4 |
| GRINA |
| GSAP |
| GUCY1A2 |
| GULP1 |
| H1-0 |
| H19 |
| H4C14 |
| H4C15 |
| HBA2 |
| HBB |
| HBD |
| HBG2 |
| HBP1 |
| HECW2 |
| HEG1 |
| HERC2P2 |
| HERC2P4 |
| HHIP |
| HIBCH |
| HILPDA |
| HMCN1 |
| HOMER1 |
| HSD17B10 |
| HSDL2 |
| HSPB1 |
| HTRA4 |
| ICAM3 |
| IDH2 |
| IFI35 |
| IFITM1 |
| IGIP |
| IL10RA |
| IL15RA |
| INPP4B |
| INSIG1 |
| IRF8 |
| ISM1 |
| ITGA6 |
| ITGAX |
| ITGB2 |
| ITGB6 |
| JAML |
| JMY |
| KANSL1L |
| KCNAB1 |
| KCNAB2 |
| KCNT2 |
| KCTD16 |
| KDM3A |
| KDR |
| KIAA1109 |
| KIAA1586 |
| KIF13A |
| KIF16B |
| KIF3A |
| KIT |
| KLF12 |
| KLHL3 |
| KLHL4 |
| KLHL5 |
| KRT19 |
| KRT7 |
| LAIR1 |
| LAPTM5 |
| LEPR |
| LFNG |
| LGALS1 |
| LGALS9C |
| LGALSL |
| LGR4 |
| LINC00312 |
| LINC00597 |
| LINC01002 |
| LINC01140 |
| LIPH |
| LMO7 |
| LOC100996333 |
| LOC283788 |
| LOC399975 |
| LOC401261 |
| LOC408186 |
| LOC646358 |
| LPXN |
| LRCH2 |
| LRIG3 |
| LRP6 |
| LRRC1 |
| LRRC17 |
| LRRC70 |
| LRRK2 |
| LRRN4 |
| LSAMP |
| LSP1P4 |
| LST1 |
| LTBP1 |
| LUC7L3 |
| LUM |
| LY96 |
| MACF1 |
| MAFB |
| MAGI1 |
| MAGI3 |
| MAP2 |
| MAP3K13 |
| MAPK10 |
| MARCO |
| MARK1 |
| MATN2 |
| MBD5 |
| MBIP |
| MCEMP1 |
| MCOLN3 |
| MDH2 |
| MEF2C |
| MEG3 |
| MET |
| MFAP5 |
| MFNG |
| MGP |
| MICU3 |
| MIER3 |
| MIPOL1 |
| MIR186 |
| MIR224 |
| MIR29C |
| MIR30C2 |
| MIRLET7F1 |
| MLLT3 |
| MMP14 |
| MMP16 |
| MPDZ |
| MPEG1 |
| MPP5 |
| MPP7 |
| MRPS15 |
| MS4A2 |
| MSLN |
| MTERF2 |
| MTND1P23 |
| MTUS1 |
| MTX3 |
| MVP |
| MYBL1 |
| MYD88 |
| MYH10 |
| MYH11 |
| MYO10 |
| MYO1B |
| MYO1F |
| MYO1G |
| MYO5B |
| MYO5C |
| MYO9A |
| MYOC |
| MYOCD |
| N4BP2 |
| NAALADL2 |
| NAGA |
| NAGK |
| NAPEPLD |
| NBEAL1 |
| NCF1 |
| NCF4 |
| NDNF |
| NDUFB10 |
| NECAB1 |
| NECTIN3 |
| NEK3 |
| NET1 |
| NFIB |
| NKTR |
| NME2 |
| NOP10 |
| NOSTRIN |
| NOTCH3 |
| NPAS2 |
| NPHP3 |
| NPHP3-ACAD11 |
| NPR3 |
| NR1D1 |
| NR1D2 |
| NR3C2 |
| NRM |
| NRROS |
| NT5DC2 |
| NT5E |
| NTS |
| NUAK2 |
| OCLN |
| OFD1 |
| OGFRL1 |
| OGN |
| OLFML1 |
| OSBPL9 |
| OVCH1 |
| OVOS2 |
| OXR1 |
| P2RY12 |
| P2RY14 |
| PAG1 |
| PALMD |
| PAMR1 |
| PAPOLG |
| PARD6B |
| PARVG |
| PATJ |
| PCDH15 |
| PCDHB14 |
| PCF11 |
| PCM1 |
| PCMTD1 |
| PCYT2 |
| PDCD4 |
| PDE10A |
| PDE1A |
| PDE3A |
| PDE4D |
| PDE5A |
| PDE8B |
| PDGFB |
| PDGFD |
| PDGFRL |
| PDZK1IP1 |
| PDZRN3 |
| PEAK1 |
| PER3 |
| PFKFB3 |
| PFN1P2 |
| PGAP1 |
| PHACTR2 |
| PHEX |
| PHF3 |
| PHIP |
| PHOSPHO2 |
| PIK3C2G |
| PIK3CA |
| PILRA |
| PKM |
| PLA2R1 |
| PLBD2 |
| PLCB1 |
| PLCB2 |
| PLCB4 |
| PLCE1 |
| PLCH1 |
| PLD1 |
| PLEKHA1 |
| PLEKHA5 |
| PLK2 |
| PLN |
| PLOD1 |
| PLOD3 |
| PLP2 |
| PLPPR1 |
| PLPPR4 |
| PLSCR4 |
| PLTP |
| PNISR |
| PNPLA6 |
| POSTN |
| PPARGC1A |
| PPFIBP1 |
| PPP1R11 |
| PPP1R12A |
| PPP1R9A |
| PPP2R3A |
| PPP4R4 |
| PRAF2 |
| PRCP |
| PRELID1 |
| PREX1 |
| PREX2 |
| PRG4 |
| PRKAA2 |
| PRKCI |
| PRKD1 |
| PRKG1 |
| PROS1 |
| PRSS23 |
| PRTG |
| PSD3 |
| PSIP1 |
| PSMB10 |
| PSMB3 |
| PSME2 |
| PTCH2 |
| PTGFR |
| PTPN13 |
| PTPN3 |
| PTPN6 |
| PTPRD |
| PUS10 |
| PWAR6 |
| RAB1C |
| RAB23 |
| RAB3IP |
| RAB5IF |
| RABGAP1L |
| RALA |
| RALGAPA2 |
| RANBP17 |
| RAPGEF2 |
| RAPGEF5 |
| RASAL2 |
| RASSF8 |
| RASSF9 |
| RAVER2 |
| RB1CC1 |
| RBM41 |
| RBMS3 |
| RCN3 |
| RDX |
| RELN |
| RENBP |
| RFC1 |
| RGS13 |
| RGS5 |
| RGS6 |
| RHOBTB1 |
| RHOF |
| RHOJ |
| RICTOR |
| RIMKLB |
| RMDN2 |
| RNASE6 |
| RNF133 |
| RNF148 |
| RNF152 |
| RNF180 |
| RNF19A |
| RNU4-1 |
| RNU4-2 |
| RNU5D-1 |
| RNU5E-1 |
| ROBO1 |
| ROBO2 |
| ROS1 |
| RPS23 |
| RPS26 |
| RPS6KA5 |
| RSRP1 |
| RUFY3 |
| RUVBL2 |
| RYR2 |
| S100A11 |
| S100A16 |
| S100A3 |
| SACM1L |
| SAMD12 |
| SAMD5 |
| SCAPER |
| SCARA5 |
| SCARB1 |
| SCEL |
| SCIMP |
| SCIN |
| SCML1 |
| SCNN1G |
| SECISBP2L |
| SECTM1 |
| SELP |
| SELPLG |
| SEMA3D |
| SEMA3E |
| SEMA3F |
| SEMA6D |
| SENP7 |
| SEPTIN11 |
| SERPINB9P1 |
| SESN3 |
| SESTD1 |
| SFRP4 |
| SGCB |
| SGCD |
| SGIP1 |
| SH2D4A |
| SHMT1 |
| SHPRH |
| SHROOM4 |
| SIGLEC1 |
| SIRPA |
| SIRPB1 |
| SLAMF8 |
| SLC11A1 |
| SLC12A2 |
| SLC15A2 |
| SLC1A5 |
| SLC22A3 |
| SLC25A30 |
| SLC26A2 |
| SLC31A2 |
| SLC35A1 |
| SLC35A4 |
| SLC35B1 |
| SLC35C1 |
| SLC38A5 |
| SLC3A2 |
| SLC43A2 |
| SLC47A1 |
| SLC50A1 |
| SLC5A4 |
| SLC6A9 |
| SLC7A7 |
| SLC9A3R1 |
| SLC9A9 |
| SLCO2B1 |
| SLCO4C1 |
| SLK |
| SLU7 |
| SMARCA1 |
| SMCO4 |
| SMIM4 |
| SMURF2 |
| SNCA |
| SNORA14B |
| SNORA21 |
| SNORA24 |
| SNORA3B |
| SNORA64 |
| SNORA65 |
| SNORA70F |
| SNORA71A |
| SNORA71C |
| SNORA71D |
| SNORD113-3 |
| SNORD113-4 |
| SNORD114-2 |
| SNORD116-28 |
| SNORD1C |
| SNORD24 |
| SNORD34 |
| SNORD45B |
| SNORD74 |
| SNRK |
| SNRNP48 |
| SNRPA1 |
| SNX25 |
| SNX30 |
| SOX5 |
| SPARC |
| SPATS2 |
| SPI1 |
| SPICE1 |
| SPN |
| SPTBN1 |
| SPTLC3 |
| SRD5A3 |
| ST14 |
| ST3GAL5 |
| ST3GAL6 |
| STAG2 |
| STK10 |
| STRBP |
| STXBP2 |
| SULT1C2 |
| SYNE1 |
| SYNE2 |
| SYNM |
| SYNPO2 |
| SYTL2 |
| TAGLN |
| TALDO1 |
| TARBP1 |
| TAS2R13 |
| TAS2R14 |
| TAS2R19 |
| TAS2R20 |
| TAS2R31 |
| TAS2R4 |
| TAS2R46 |
| TAS2R50 |
| TBC1D15 |
| TBC1D2 |
| TBC1D4 |
| TCF4 |
| TEK |
| TGM2 |
| THEMIS2 |
| THSD7A |
| TIA1 |
| TJP1 |
| TKT |
| TLCD4 |
| TLL1 |
| TMEM106A |
| TMEM141 |
| TMEM176A |
| TMEM176B |
| TMEM97 |
| TMOD2 |
| TNFRSF1B |
| TNIK |
| TOMM40 |
| TP53 |
| TPP1 |
| TRAK2 |
| TRAM2 |
| TRANK1 |
| TRAPPC1 |
| TREM2 |
| TRIM23 |
| TRPC1 |
| TRPC6 |
| TRPV2 |
| TSPAN2 |
| TTC21B |
| TTLL7 |
| TUBB4B |
| TWF2 |
| TXK |
| TXLNG |
| TYMP |
| TYRP1 |
| UACA |
| UBE2MP1 |
| UCP2 |
| UQCC2 |
| USP31 |
| USP47 |
| UTRN |
| VAV1 |
| VEPH1 |
| VPS13A |
| VSNL1 |
| VTRNA1-1 |
| WBP4 |
| WDFY4 |
| WDR17 |
| WDR19 |
| WDR48 |
| WIF1 |
| WNT5A |
| XKRX |
| ZBED8 |
| ZC3H6 |
| ZDBF2 |
| ZEB1 |
| ZFAND2A |
| ZFPM2 |
| ZFX |
| ZFYVE9 |
| ZNF10 |
| ZNF117 |
| ZNF136 |
| ZNF14 |
| ZNF141 |
| ZNF154 |
| ZNF160 |
| ZNF175 |
| ZNF189 |
| ZNF204P |
| ZNF208 |
| ZNF224 |
| ZNF234 |
| ZNF253 |
| ZNF254 |
| ZNF266 |
| ZNF292 |
| ZNF300P1 |
| ZNF302 |
| ZNF33A |
| ZNF33B |
| ZNF347 |
| ZNF350 |
| ZNF43 |
| ZNF443 |
| ZNF486 |
| ZNF493 |
| ZNF507 |
| ZNF510 |
| ZNF518B |
| ZNF521 |
| ZNF528 |
| ZNF559 |
| ZNF594 |
| ZNF615 |
| ZNF654 |
| ZNF682 |
| ZNF711 |
| ZNF720 |
| ZNF737 |
| ZNF780A |
| ZNF780B |
| ZNF791 |
| ZNF814 |
| ZNF83 |
| ZNF84 |
| ZNF844 |
| ZNF91 |
| ZRANB2 |
| ZSCAN31 |
