# Supplementary material for: FAM171B as a Novel Biomarker Mediates Tissue Immune Microenvironment in Pulmonary Arterial Hypertension
Source: Mediators Inflamm. 2022 Sep 22;2022:1878766. doi: 10.1155/2022/1878766 (PMC9553458; doi:10.1155/2022/1878766)
Supplement: Supplementary Materials — Supplementary Table 1: The results of differentially expressed genes (DEGs). Supplementary Table 2: Gene Ontology (GO) enrichment analysis results of differentially expressed genes (DEGs). Supplementary Table 3: Kyoto Encyclopedia of Genes and Genomes (KEGG) enrichment analysis results of differentially expressed genes (DEGs). Supplementary Table 4: Disease Ontology (DO) enrichment analysis results of differentially expressed genes (DEGs). Supplementary Table 5: Metascape function analysis results of differentially expressed genes (DEGs). Supplementary Table 6: results of Gene Set Enrichment Analysis (GSEA) of gene expression matrix. Supplementary Table 7: results of all genes in brown module. Supplementary Table 8: results of key genes in brown module. Supplementary Table 9: results of analyzing the combined data matrix of GSE113439 and GSE117261 using CIBERSORT. Supplementary Table 10: results of the correlation of FAM171B with immune cells. [file 1878766.f1.zip › Supplementary Table8.docx]

| gene | MMbrown | p.MMbrown | GS.PAH | p.GS.PAH |
| --- | --- | --- | --- | --- |
| ABCC9 | 0.680179075 | 5.69E-16 | 0.601518954 | 5.81E-12 |
| AHI1 | 0.569726737 | 1.24E-10 | 0.579124258 | 5.18E-11 |
| ANKRD36 | 0.700213841 | 3.36E-17 | 0.589938252 | 1.84E-11 |
| ANKRD36B | 0.710967269 | 6.68E-18 | 0.591831226 | 1.53E-11 |
| ANKRD36C | 0.725596389 | 6.55E-19 | 0.66614473 | 3.62E-15 |
| ARHGAP21 | 0.758351442 | 2.02E-21 | 0.581908478 | 3.98E-11 |
| CACNA2D1 | 0.690831411 | 1.30E-16 | 0.55398035 | 4.99E-10 |
| ECM2 | 0.697965455 | 4.67E-17 | 0.630129879 | 2.74E-13 |
| FAM171B | 0.55128641 | 6.30E-10 | 0.581893099 | 3.99E-11 |
| FRMD4B | 0.617020568 | 1.15E-12 | 0.5580846 | 3.49E-10 |
| GLT8D2 | 0.582928579 | 3.61E-11 | 0.574132144 | 8.24E-11 |
| JMY | 0.731049426 | 2.65E-19 | 0.553414387 | 5.24E-10 |
| KLF12 | 0.691219603 | 1.23E-16 | 0.558662582 | 3.32E-10 |
| LUC7L3 | 0.850309547 | 2.55E-31 | 0.582391802 | 3.80E-11 |
| MACF1 | 0.754989343 | 3.81E-21 | 0.550808831 | 6.56E-10 |
| N4BP2 | 0.639027205 | 9.92E-14 | 0.561819535 | 2.51E-10 |
| NBEAL1 | 0.837778467 | 1.28E-29 | 0.583602753 | 3.39E-11 |
| NT5E | 0.621517334 | 7.09E-13 | 0.604581711 | 4.25E-12 |
| PHIP | 0.792922869 | 1.52E-24 | 0.593248593 | 1.33E-11 |
| PNISR | 0.822705824 | 9.33E-28 | 0.559914961 | 2.98E-10 |
| RORA | 0.735429967 | 1.26E-19 | 0.568059841 | 1.44E-10 |
| RPS6KA5 | 0.68991734 | 1.48E-16 | 0.581071458 | 4.31E-11 |
| RUFY3 | 0.690027978 | 1.46E-16 | 0.625568868 | 4.55E-13 |
| SHPRH | 0.818858918 | 2.61E-27 | 0.58461363 | 3.08E-11 |
| WIF1 | 0.611594392 | 2.05E-12 | 0.554285602 | 4.86E-10 |
| ZNF483 | 0.564077159 | 2.06E-10 | 0.580082846 | 4.73E-11 |
| ZNF711 | 0.700259409 | 3.34E-17 | 0.574791418 | 7.76E-11 |
