# Supplementary material for: FAM171B as a Novel Biomarker Mediates Tissue Immune Microenvironment in Pulmonary Arterial Hypertension
Source: Mediators Inflamm. 2022 Sep 22;2022:1878766. doi: 10.1155/2022/1878766 (PMC9553458; doi:10.1155/2022/1878766)
Supplement: Supplementary Materials — Supplementary Table 1: The results of differentially expressed genes (DEGs). Supplementary Table 2: Gene Ontology (GO) enrichment analysis results of differentially expressed genes (DEGs). Supplementary Table 3: Kyoto Encyclopedia of Genes and Genomes (KEGG) enrichment analysis results of differentially expressed genes (DEGs). Supplementary Table 4: Disease Ontology (DO) enrichment analysis results of differentially expressed genes (DEGs). Supplementary Table 5: Metascape function analysis results of differentially expressed genes (DEGs). Supplementary Table 6: results of Gene Set Enrichment Analysis (GSEA) of gene expression matrix. Supplementary Table 7: results of all genes in brown module. Supplementary Table 8: results of key genes in brown module. Supplementary Table 9: results of analyzing the combined data matrix of GSE113439 and GSE117261 using CIBERSORT. Supplementary Table 10: results of the correlation of FAM171B with immune cells. [file 1878766.f1.zip › Supplementary Table9.docx]

| id | B cells naive | B cells memory | Plasma cells | T cells CD8 | T cells CD4 naive | T cells CD4 memory resting | T cells CD4 memory activated | T cells follicular helper | T cells regulatory (Tregs) | T cells gamma delta | NK cells resting | NK cells activated | Monocytes | Macrophages M0 | Macrophages M1 | Macrophages M2 | Dendritic cells resting | Dendritic cells activated | Mast cells resting | Mast cells activated | Eosinophils | Neutrophils |
| --- | --- | --- | --- | --- | --- | --- | --- | --- | --- | --- | --- | --- | --- | --- | --- | --- | --- | --- | --- | --- | --- | --- |
| GSM3106341_con | 0.06956303 | 0 | 0.100991251 | 0.062587797 | 0 | 0.23985493 | 0 | 0 | 0 | 0 | 0 | 0.010401625 | 0.030674066 | 0.036726942 | 0.020492302 | 0.190421638 | 0 | 0.064718321 | 0.153753196 | 0 | 0 | 0.019814901 |
| GSM3106342_con | 0.047149563 | 0 | 0.083319208 | 0.047737187 | 0 | 0.181384697 | 0.010748051 | 0 | 0 | 0 | 0.013430003 | 0.00567984 | 0.030282564 | 0.042702979 | 0.000269185 | 0.218423547 | 0 | 0.032580224 | 0.153110044 | 0 | 0 | 0.13318291 |
| GSM3106343_con | 0.062102347 | 0 | 0.089964587 | 0.089981424 | 0 | 0.184331565 | 0 | 0 | 0 | 0 | 0.017366316 | 0.010153692 | 0.023561334 | 0.105497873 | 0.013511304 | 0.245648012 | 0 | 0.010575758 | 0.129236397 | 0 | 0 | 0.018069391 |
| GSM3106344_con | 0.066257205 | 0 | 0.063256994 | 0.050395829 | 0 | 0.148901543 | 0.007045208 | 0 | 0 | 0 | 0.037474789 | 0 | 0.06301308 | 0.09855181 | 0.013043224 | 0.19627576 | 0 | 0.043815977 | 0.17786526 | 0 | 0 | 0.03410332 |
| GSM3106345_con | 0.080131236 | 0 | 0.063486202 | 0.0632961 | 0 | 0.156938918 | 0.005694362 | 0 | 0 | 0.009679579 | 0.037803564 | 0 | 0.054511717 | 0.076982491 | 0.024881306 | 0.16543096 | 0 | 0.087193046 | 0.120758674 | 0 | 0.003244265 | 0.04996758 |
| GSM3106346_con | 0.067866841 | 0 | 0.038535605 | 0.049004103 | 0 | 0.194877587 | 0 | 0 | 0 | 0 | 0.037077758 | 0 | 0.03957167 | 0.147531566 | 0.017077634 | 0.243202749 | 0 | 0.022899954 | 0.085989854 | 0 | 0.007819528 | 0.048545151 |
| GSM3106347_con | 0.076057498 | 0 | 0.065173754 | 0.101150382 | 0 | 0.136707968 | 0 | 0 | 0 | 0 | 0.060292755 | 0 | 0.077928163 | 0.080431762 | 0.012155905 | 0.240251552 | 0 | 0.00190102 | 0.126793218 | 0 | 0 | 0.021156022 |
| GSM3106348_con | 0.089713691 | 0 | 0.068370004 | 0.018358328 | 0 | 0.117354261 | 0.001981326 | 0 | 0 | 0 | 0.030637697 | 0 | 0.029410001 | 0.108797091 | 0.005381033 | 0.208381711 | 0 | 0.117366027 | 0.111045586 | 0 | 0.013523774 | 0.079679472 |
| GSM3106349_con | 0.120894519 | 0 | 0.037501119 | 0.113977008 | 0 | 0.208518046 | 0 | 0 | 0 | 0 | 0.023624756 | 0 | 0.069325003 | 0.068827016 | 0.004081648 | 0.134526185 | 0 | 0.047730925 | 0.117455271 | 0 | 0 | 0.053538504 |
| GSM3106350_con | 0.114115978 | 0 | 0.112100172 | 0.052631968 | 0 | 0.14727794 | 0 | 0 | 0 | 0 | 0.01704495 | 0 | 0.021532822 | 0.073916872 | 0.005217837 | 0.298994904 | 0 | 0.050911714 | 0.061723005 | 0 | 0 | 0.044531838 |
| GSM3106351_con | 0.066220572 | 0 | 0.065705128 | 0.133757026 | 0 | 0.201474793 | 0 | 0 | 0 | 0 | 0.034633762 | 0.004505791 | 0.045165777 | 0.015406727 | 0.029164053 | 0.248335269 | 0 | 0.017865839 | 0.114277498 | 0 | 0 | 0.023487765 |
| GSM3290083_con | 0.072508431 | 0 | 0.010984933 | 0.037575815 | 0.017713361 | 0.169263005 | 0.05712055 | 0 | 0 | 0 | 0.057317057 | 0 | 0.197400878 | 0.019948961 | 0.088373114 | 0.145403556 | 0 | 0.016975875 | 0.016792196 | 0 | 0 | 0.092622267 |
| GSM3290085_con | 0.14760509 | 0 | 0.035884827 | 0.024069363 | 0 | 0.285569073 | 0 | 0 | 0 | 0 | 0.028063487 | 0 | 0.100605297 | 0 | 0.005176445 | 0.184066593 | 0 | 0.024994653 | 0.11275048 | 0 | 0 | 0.051214694 |
| GSM3290086_con | 0.145547679 | 0 | 0.071689703 | 0.037113169 | 0 | 0.175144989 | 0.000128836 | 0 | 0 | 0 | 0.068890188 | 0 | 0.055411343 | 0.064791563 | 0 | 0.172827679 | 0 | 0.061484568 | 0.009794619 | 0.025118416 | 0.017804433 | 0.094252815 |
| GSM3290088_con | 0.001442528 | 0.009724559 | 0.050231633 | 0.060366144 | 0 | 0.075299085 | 0.003819282 | 0 | 0 | 0 | 0.031704488 | 0 | 0.034970831 | 0.235298871 | 0 | 0.186483119 | 0 | 0.117026899 | 0.031389937 | 0.024294905 | 0.040303291 | 0.097644429 |
| GSM3290091_con | 0.117245787 | 0 | 0.083338584 | 0.030442772 | 0 | 0.157101127 | 0 | 0 | 0 | 0 | 0.023617046 | 0 | 0.043949718 | 0.07531118 | 0.000239249 | 0.191409394 | 0 | 0.060058108 | 0.116786546 | 0 | 0.011685755 | 0.088814733 |
| GSM3290092_con | 0.06153803 | 0 | 0.044391396 | 0.025931355 | 0 | 0.138444182 | 0.01497182 | 0 | 0 | 0 | 0.048389394 | 0 | 0.066320436 | 0.11139399 | 0.01357017 | 0.300359301 | 0 | 0.0032254 | 0.086290913 | 0 | 0 | 0.085173614 |
| GSM3290093_con | 0.064676613 | 0 | 0.067774032 | 0.027748719 | 0 | 0.122108401 | 0 | 0 | 0 | 0 | 0.038974908 | 0 | 0.091179773 | 0.113616362 | 0.003069659 | 0.243702687 | 0 | 0.024166064 | 0.108847023 | 0 | 0.022938528 | 0.071197232 |
| GSM3290094_con | 0.06993013 | 0 | 0.053228555 | 0.023599019 | 0 | 0.149208401 | 0 | 0 | 0 | 0 | 0.024324882 | 0 | 0.001647131 | 0.12960633 | 0.003790071 | 0.20191144 | 0 | 0.071472749 | 0.141864775 | 0 | 0.007138997 | 0.122277521 |
| GSM3290097_con | 0.084898629 | 0 | 0.088699461 | 0.046911267 | 0 | 0.149755492 | 0.000759193 | 0 | 0 | 0 | 0.044059391 | 0 | 0.030260505 | 0.054333675 | 0 | 0.107695827 | 0 | 0.140067974 | 0.119626053 | 0 | 0.044924761 | 0.088007772 |
| GSM3290098_con | 0.08246864 | 0 | 0.036909659 | 0.026766803 | 0 | 0.084841403 | 0 | 0 | 0 | 0 | 0.029041653 | 0.001259144 | 0.111969539 | 0.199849125 | 0 | 0.058721929 | 0 | 0.182102537 | 0.028820667 | 0 | 0 | 0.157248901 |
| GSM3290100_con | 0.095078676 | 0 | 0.054406793 | 0.012178998 | 0 | 0.102111375 | 0 | 0 | 0 | 0 | 0.017398025 | 0 | 0.122641353 | 0.097563006 | 0.007831035 | 0.238384985 | 0 | 0.010921057 | 0.086262329 | 0 | 0 | 0.155222369 |
| GSM3290101_con | 0.043010862 | 0 | 0.07112041 | 0.024891235 | 0 | 0.165770779 | 0 | 0 | 0 | 0 | 0.007639288 | 0.009411211 | 0.130691965 | 0.087014116 | 0.004589828 | 0.187890768 | 0 | 0.063543811 | 0.122068553 | 0 | 0 | 0.082357174 |
| GSM3290102_con | 0.087722155 | 0 | 0.045442428 | 0.027815418 | 0 | 0.099361418 | 0 | 0.002105775 | 0 | 0 | 0.032367324 | 0 | 0.082689637 | 0.079851795 | 0.00471265 | 0.204078773 | 0 | 0.137213354 | 0.1202028 | 0 | 0 | 0.076436473 |
| GSM3290118_con | 0.069268753 | 0 | 0.021691526 | 0.053633138 | 0 | 0.180580853 | 0 | 0 | 0 | 0 | 0.022931674 | 0.016959225 | 0.05684675 | 0.235579304 | 0.004726165 | 0.150464468 | 0.013122331 | 0.01165496 | 0.108624183 | 0 | 0 | 0.053916671 |
| GSM3290119_con | 0.073695623 | 0 | 0.024784406 | 0.030303811 | 0.057700154 | 0.146302895 | 0.001216405 | 0 | 0 | 0 | 0.054563785 | 0 | 0.190983248 | 0 | 0 | 0.134290764 | 0 | 0.010275376 | 0.094217553 | 0 | 0 | 0.18166598 |
| GSM3290122_con | 0.031037773 | 0 | 0.064734245 | 0.06509565 | 0.016410692 | 0.219590611 | 0 | 0 | 0 | 0 | 0.068581506 | 0 | 0.073352235 | 0.062528967 | 0 | 0.213062121 | 0 | 0.032002149 | 0.079575786 | 0 | 0 | 0.074028265 |
| GSM3290123_con | 0.051498987 | 0 | 0.038170364 | 0.017148565 | 0 | 0.108114932 | 0 | 0 | 0 | 0 | 0.044325369 | 0 | 0.238797501 | 0.097054327 | 0 | 0.099853363 | 0 | 0.04322311 | 0 | 0.058391861 | 0.015425999 | 0.18799562 |
| GSM3290124_con | 0.0548918 | 0 | 0.083740788 | 0.023858635 | 0 | 0.0950823 | 0.000519536 | 0 | 0 | 0 | 0.03208397 | 0 | 0.097658554 | 0.190173874 | 0.008306134 | 0.24307502 | 0 | 0 | 0.07591004 | 0 | 0.004216039 | 0.09048331 |
| GSM3290125_con | 0.083772968 | 0 | 0.049063164 | 0.037782799 | 0 | 0.132471825 | 0 | 0 | 0 | 0 | 0.024092514 | 0 | 0.043670929 | 0.184621739 | 0 | 0.221044784 | 0 | 0.040877557 | 0.113806702 | 0 | 0.006087518 | 0.062707503 |
| GSM3290131_con | 0.064606639 | 0 | 0.025948026 | 0.036216837 | 0.036800283 | 0.20020344 | 0 | 0 | 0 | 0 | 0.029745492 | 0.021642353 | 0.067504442 | 0.12498759 | 0 | 0.129471544 | 0 | 0.019044864 | 0.154352769 | 0 | 0 | 0.089475722 |
| GSM3290132_con | 0.058205229 | 0 | 0.046615957 | 0.024905799 | 0 | 0.095067978 | 0 | 0 | 0 | 0 | 0.06086812 | 0 | 0.135707699 | 0.081892292 | 0.003184851 | 0.106712394 | 0 | 0.144701277 | 0.08284137 | 0 | 0 | 0.159297034 |
| GSM3290133_con | 0.061320573 | 0 | 0.095722231 | 0.059918433 | 0 | 0.145767647 | 0.008286088 | 0 | 0 | 0 | 0.028669971 | 0 | 0.019694863 | 0.190326145 | 0 | 0.145535312 | 0 | 0.064088379 | 0.119639006 | 0 | 0.002752965 | 0.058278388 |
| GSM3290134_con | 0.067020832 | 0 | 0.043325847 | 0.029376226 | 0 | 0.090097433 | 0 | 0 | 0 | 0 | 0.026530204 | 0 | 0.17807913 | 0.104535628 | 0.000103926 | 0.211292787 | 0 | 0.037133141 | 0.128881879 | 0 | 0 | 0.083622965 |
| GSM3290146_con | 0.115531557 | 0 | 0.072856178 | 0.077968698 | 0 | 0.142480237 | 0.013357984 | 0 | 0 | 0 | 0.059112389 | 0 | 0.034387944 | 0.124429154 | 0.006097502 | 0.179486592 | 0 | 0.000329824 | 0.118040303 | 0 | 0.002093889 | 0.053827749 |
| GSM3290147_con | 0.065379389 | 0 | 0.031836797 | 0.03400555 | 0 | 0.133285756 | 0.000252678 | 0 | 0 | 0 | 0.052095172 | 0 | 0.119452206 | 0.048158169 | 0.029318336 | 0.230749056 | 0 | 0.111265411 | 0.090486692 | 0 | 0 | 0.053714789 |
| GSM3106326_treat | 0.101176905 | 0 | 0.086987297 | 0.027802611 | 0 | 0.143313135 | 0.001172123 | 0 | 0 | 0 | 0.03431203 | 0 | 0.090182481 | 0.079069477 | 0 | 0.14032883 | 0 | 0.121651186 | 0.095097138 | 0 | 0.017195607 | 0.06171118 |
| GSM3106327_treat | 0.067073791 | 0 | 0.032513754 | 0.003651779 | 0 | 0.147487769 | 0 | 0 | 0 | 0 | 0.061340166 | 0 | 0.062760102 | 0.225589007 | 0.014576421 | 0.118696191 | 0 | 0.112970483 | 0.029487288 | 0 | 0.020129603 | 0.103723646 |
| GSM3106328_treat | 0.102720799 | 0 | 0.024078303 | 0.015135181 | 0 | 0.262877425 | 0.001978463 | 0 | 0 | 0 | 0 | 0.019331123 | 0.039569413 | 0.056328568 | 0.033677523 | 0.168498382 | 0 | 0.033075049 | 0.186760864 | 0 | 0.01195449 | 0.044014415 |
| GSM3106329_treat | 0.105064521 | 0 | 0.100909852 | 0.027759452 | 0 | 0.16291473 | 0 | 0 | 0 | 0 | 0 | 0 | 0.029827737 | 0.04786811 | 0.002681174 | 0.278077657 | 0 | 0.023065759 | 0.183020071 | 0 | 0.017366707 | 0.021444231 |
| GSM3106330_treat | 0.110609069 | 0 | 0.041875838 | 0.052602898 | 0 | 0.285334337 | 0 | 0 | 0 | 0 | 0 | 0.009523748 | 0.023135609 | 0.017262869 | 0 | 0.182233302 | 0 | 0.048713829 | 0.174844063 | 0 | 0.004790401 | 0.049074037 |
| GSM3106331_treat | 0.108128458 | 0 | 0.066493583 | 0.009190952 | 0 | 0.183895076 | 0.003622732 | 0.003364231 | 0 | 0 | 0.048828915 | 0 | 0.021317287 | 0.12717046 | 0.003567409 | 0.141202527 | 0 | 0.102832207 | 0.086180417 | 0.001709298 | 0.03495525 | 0.057541197 |
| GSM3106332_treat | 0.137198457 | 0 | 0.116310806 | 0.011009562 | 0 | 0.168951742 | 0.003062135 | 0 | 0 | 0 | 0.02719617 | 0 | 0.030932484 | 0.056891833 | 0 | 0.236690382 | 0 | 0.058352633 | 0.104636733 | 0 | 0 | 0.048767062 |
| GSM3106333_treat | 0.083203803 | 0 | 0.077738602 | 0.023149577 | 0 | 0.158058239 | 0 | 0 | 0 | 0 | 0.041081292 | 0 | 0.023503273 | 0.144517524 | 0.00219973 | 0.228511472 | 0 | 0.070019353 | 0.084912438 | 0 | 0.034523538 | 0.02858116 |
| GSM3106334_treat | 0.014861541 | 0.070642642 | 0.037823201 | 0 | 0 | 0.29378639 | 0.009850826 | 0 | 0 | 0 | 0.046508449 | 0 | 0.027078746 | 0.082038565 | 0 | 0.135493072 | 0 | 0.127247784 | 0.06077331 | 0 | 0 | 0.093895474 |
| GSM3106335_treat | 0.039547106 | 0 | 0.030073467 | 0.001591201 | 0 | 0.100561737 | 0 | 0 | 0 | 0 | 0.031747329 | 0.000915096 | 0.211871341 | 0.179512738 | 0.018556 | 0.148058492 | 0 | 0.08356256 | 0.088163962 | 0 | 0.008624791 | 0.057214179 |
| GSM3106336_treat | 0.069933478 | 0 | 0.085494117 | 0.033177052 | 0 | 0.167708739 | 0 | 0 | 0 | 0 | 0.040014784 | 0 | 0.035922443 | 0.127420958 | 0.005074511 | 0.18601438 | 0 | 0.104648053 | 0.067153483 | 0 | 0.021297901 | 0.056140102 |
| GSM3106337_treat | 0.109142538 | 0 | 0.065510783 | 0.021898971 | 0 | 0.183840058 | 0 | 0 | 0 | 0 | 0.024364194 | 0 | 0.019088293 | 0.112758215 | 0.009580411 | 0.168130522 | 0 | 0.092948275 | 0.132108116 | 0 | 0.005109215 | 0.055520407 |
| GSM3106338_treat | 0.09903221 | 0 | 0.077035719 | 0.073291842 | 0 | 0.185822685 | 0 | 0.00559294 | 0 | 0 | 0.017900339 | 0 | 0.049725334 | 0.02271628 | 0.002776192 | 0.110005839 | 0 | 0.063915411 | 0.178229023 | 0 | 0.018364587 | 0.095591599 |
| GSM3106339_treat | 0.089762875 | 0 | 0.04720583 | 0.035282357 | 0 | 0.180720626 | 0 | 0 | 0 | 0 | 0.03991955 | 0 | 0.047397706 | 0.103602643 | 0.057516879 | 0.12282785 | 0 | 0.114318955 | 0.092239015 | 0 | 0.006186335 | 0.063019379 |
| GSM3106340_treat | 0.095706948 | 0 | 0.071116328 | 0.051808186 | 0 | 0.191757589 | 0.002263671 | 0 | 0 | 0 | 0.006971763 | 0 | 0.017605931 | 0.077596405 | 0.022326144 | 0.228558472 | 0 | 0.027947681 | 0.117170853 | 0 | 0.010791597 | 0.078378433 |
| GSM3290067_treat | 0.072932231 | 0 | 0.052858884 | 0.060468377 | 0 | 0.23195329 | 0.010074468 | 0 | 0 | 0 | 0.015729241 | 0 | 0.032330707 | 0.019098316 | 0.063773792 | 0.250517365 | 0 | 0.014146222 | 0.138026568 | 0 | 0 | 0.038090539 |
| GSM3290068_treat | 0.088187816 | 0 | 0.109921708 | 0.073470587 | 0 | 0.225865862 | 0 | 0 | 0 | 0 | 0.030595599 | 0 | 0.052635113 | 0 | 7.02E-05 | 0.213055986 | 0 | 0.026256642 | 0.163142222 | 0 | 0 | 0.016798295 |
| GSM3290069_treat | 0.038538229 | 0 | 0.036688901 | 0.048529889 | 0 | 0.112976194 | 0.00544445 | 0.000798785 | 0 | 0 | 0.02261101 | 0.009604257 | 0.060681509 | 0.254725206 | 0.005679259 | 0.203285213 | 0 | 0.049718477 | 0.134529752 | 0 | 0 | 0.016188868 |
| GSM3290070_treat | 0.016648162 | 0.011422923 | 0.075266227 | 0.1107244 | 0 | 0.168965673 | 0 | 0 | 0 | 0 | 0.008992666 | 0.01442716 | 0.031432694 | 0.154583396 | 0.002057881 | 0.162080531 | 0 | 0.043633742 | 0.1565547 | 0 | 0.016628344 | 0.026581499 |
| GSM3290071_treat | 0.054806089 | 0 | 0.061814365 | 0.036285528 | 0 | 0.178920686 | 0 | 0 | 0 | 0 | 0.007206245 | 0 | 0.023770297 | 0.083879705 | 0.016851673 | 0.308685868 | 0 | 0.050127836 | 0.142800694 | 0 | 0 | 0.034851015 |
| GSM3290072_treat | 0.160850258 | 0 | 0.065980115 | 0.121908878 | 0 | 0.129319501 | 0.003983614 | 0 | 0 | 0 | 0.044814584 | 0 | 0.066874338 | 0.019676103 | 0.019271608 | 0.206154825 | 0 | 0.052352525 | 0.054003511 | 0 | 0 | 0.05481014 |
| GSM3290073_treat | 0.04031765 | 0 | 0.024324981 | 0.035193574 | 0 | 0.131968866 | 0.001053828 | 0.006374819 | 0 | 0 | 0.026096527 | 0.006525604 | 0.073997018 | 0.16438516 | 0 | 0.176542922 | 0 | 0.099667652 | 0.155997536 | 0 | 0.013890437 | 0.043663425 |
| GSM3290074_treat | 0.09926399 | 0 | 0.038906234 | 0.029255628 | 0 | 0.170058669 | 0 | 0 | 0 | 0.098762129 | 0.002254451 | 0 | 0.01550906 | 0.097983291 | 0.083580895 | 0.193247621 | 0 | 0.018236795 | 0.074592884 | 0 | 0.005943794 | 0.072404558 |
| GSM3290075_treat | 0.081156541 | 0 | 0.08989418 | 0.017759062 | 0 | 0.183824614 | 0.006546573 | 0 | 0 | 0 | 0 | 0.003072411 | 0.020904236 | 0.048034245 | 0.002640559 | 0.170384242 | 0 | 0.066398639 | 0.308523217 | 0 | 0 | 0.000861479 |
| GSM3290076_treat | 0.072787295 | 0 | 0.065658935 | 0.012657641 | 0 | 0.198745483 | 0 | 0 | 0 | 0.002756981 | 0.027851037 | 0 | 0.028621002 | 0.109113347 | 0.0080414 | 0.195121251 | 0 | 0.123637814 | 0.112932231 | 0 | 0 | 0.042075583 |
| GSM3290077_treat | 0.100124405 | 0 | 0.063555754 | 0.064133383 | 0 | 0.214647622 | 0 | 0.000354354 | 0 | 0 | 0.040884911 | 0 | 0.063884864 | 0.070978567 | 0.000365802 | 0.101796969 | 0 | 0.072311083 | 0.139830451 | 0 | 0 | 0.067131835 |
| GSM3290078_treat | 0.080979918 | 0 | 0.108068154 | 0.04596729 | 0 | 0.203426566 | 0.000314748 | 0 | 0 | 0 | 0.006543539 | 0.000450712 | 0.02243364 | 0.054427243 | 0.014892827 | 0.219054361 | 0 | 0.031539048 | 0.193040604 | 0 | 0 | 0.018861351 |
| GSM3290079_treat | 0.111521788 | 0 | 0.106213284 | 0.010888294 | 0 | 0.16437984 | 0 | 0 | 0 | 0.032501717 | 0.062678176 | 0 | 0.066409802 | 0.03626909 | 0.017061657 | 0.20595708 | 0 | 0.055597153 | 0.07390439 | 0 | 0 | 0.05661773 |
| GSM3290080_treat | 0.10276763 | 0 | 0.10173889 | 0.01346676 | 0 | 0.165127314 | 0 | 0 | 0 | 0 | 0.037802088 | 0 | 0.036181279 | 0.176502354 | 0.00685898 | 0.172869162 | 0 | 0.021019919 | 0.12466926 | 0 | 0 | 0.040996364 |
| GSM3290081_treat | 0.082585874 | 0 | 0.040706958 | 0.035047056 | 0 | 0.172201633 | 0.002482766 | 0 | 0 | 0 | 0.040388425 | 0 | 0.035590614 | 0.147117667 | 0.00475632 | 0.181168173 | 0 | 0.107332765 | 0.05877653 | 0 | 0.01771189 | 0.074133331 |
| GSM3290082_treat | 0.069498027 | 0 | 0.074289436 | 0.034568496 | 0 | 0.118940946 | 0 | 0 | 0 | 0.017072546 | 0.054229087 | 0 | 0.018162222 | 0.154284925 | 0 | 0.225434398 | 0 | 0.11845824 | 0.088903993 | 0 | 0.012267381 | 0.013890304 |
| GSM3290084_treat | 0.080008739 | 0 | 0.034718476 | 0.02652489 | 0.008006631 | 0.155378731 | 0.015610374 | 0 | 0 | 0 | 0.037312536 | 0 | 0.026777866 | 0.056372247 | 0 | 0.182449426 | 0 | 0.192995609 | 0.117138291 | 0 | 0.016542613 | 0.050163573 |
| GSM3290087_treat | 0.049852234 | 0 | 0.081724088 | 0.060149953 | 0 | 0.151211503 | 0.007087037 | 0 | 0 | 0 | 0.011745897 | 0 | 0.014800837 | 0.12229842 | 0.036471799 | 0.176898433 | 0 | 0.024603125 | 0.240432359 | 0 | 0.013315264 | 0.009409052 |
| GSM3290089_treat | 0.056977836 | 0 | 0.127285256 | 0.073817117 | 0 | 0.180718965 | 0 | 0 | 0 | 0.043199588 | 0 | 0.004012627 | 0.007293218 | 0.055083822 | 0.0109333 | 0.175077508 | 0 | 0.078347324 | 0.129853697 | 0 | 0 | 0.057399742 |
| GSM3290090_treat | 0.085549175 | 0 | 0.094478047 | 0.022759113 | 0 | 0.125030672 | 0.003019261 | 0 | 0 | 0 | 0.016621236 | 0 | 0.008603088 | 0.121180422 | 0.00764305 | 0.144312175 | 0 | 0.033522343 | 0.144932965 | 0 | 0.019915285 | 0.172433168 |
| GSM3290095_treat | 0.119064147 | 0 | 0.06083357 | 0.020223311 | 0 | 0.203252411 | 0.005909796 | 0 | 0 | 0.059607694 | 0 | 0 | 0.022825582 | 0.026988847 | 0 | 0.129694434 | 0 | 0.095215677 | 0.165215084 | 0 | 0.018943249 | 0.072226197 |
| GSM3290096_treat | 0.073704534 | 0 | 0.100352414 | 0.021799838 | 0 | 0.243435642 | 0 | 0 | 0 | 0.004408277 | 0.017406367 | 0 | 0.012930295 | 0.110492062 | 0.029668155 | 0.142899042 | 0 | 0.062137524 | 0.162471015 | 0 | 0.010343594 | 0.007951239 |
| GSM3290099_treat | 0.086120581 | 0 | 0.034971635 | 0.040715531 | 0 | 0.252220106 | 0 | 0 | 0 | 0 | 0.054449731 | 0.031214523 | 0.031140925 | 0.073639157 | 0.04156331 | 0.13238539 | 0 | 0.063020288 | 0.114608508 | 0 | 0 | 0.043950315 |
| GSM3290103_treat | 0.093752295 | 0 | 0.109777092 | 0.030906678 | 0 | 0.218174559 | 0 | 0 | 0 | 0 | 0.039297335 | 0 | 0.02941882 | 0.032676668 | 0.007962232 | 0.181575174 | 0 | 0.073891785 | 0.164814242 | 0 | 0 | 0.017753119 |
| GSM3290104_treat | 0.034663721 | 0 | 0.092961751 | 0.02165659 | 0 | 0.169617063 | 0 | 0 | 0 | 0 | 0.007793003 | 0 | 0.017505837 | 0.133047638 | 0.008910907 | 0.293236613 | 0 | 0.047045247 | 0.168769311 | 0 | 0 | 0.00479232 |
| GSM3290105_treat | 0.066160236 | 0 | 0.048994369 | 0.04673372 | 0 | 0.246083694 | 0.001814317 | 0 | 0 | 0 | 0.024469365 | 0 | 0.029552135 | 0.082876357 | 0.007023294 | 0.227669561 | 0 | 0.022528802 | 0.089200555 | 0 | 0 | 0.106893596 |
| GSM3290106_treat | 0.108706085 | 0 | 0.069509539 | 0.036673424 | 0 | 0.154113712 | 0.004911856 | 0 | 0 | 0 | 0.03822629 | 0.00555832 | 0.028232858 | 0.01261992 | 0 | 0.222815079 | 0 | 0.096805548 | 0.202195025 | 0 | 0 | 0.019632345 |
| GSM3290107_treat | 0.09131971 | 0 | 0.091722427 | 0.01299339 | 0 | 0.180340599 | 0 | 0 | 0 | 0 | 0.003451478 | 0.005622138 | 0.009085797 | 0.102099674 | 0.054289269 | 0.227773583 | 0 | 0.051783134 | 0.119415518 | 0 | 0 | 0.050103284 |
| GSM3290108_treat | 0.107257778 | 0 | 0.089155724 | 0.061638149 | 0 | 0.21431826 | 0.005387088 | 0 | 0 | 0 | 0.022691542 | 0 | 0.023865475 | 0.06540925 | 0.021385567 | 0.19071609 | 0 | 0.061115024 | 0.114814669 | 0 | 0 | 0.022245383 |
| GSM3290109_treat | 0.043784215 | 0 | 0.011685426 | 0.395285579 | 0 | 0.131058136 | 0.019508116 | 0.021795385 | 0 | 0.101619159 | 0 | 0 | 0 | 0.058446389 | 0.063973478 | 0.118075261 | 0 | 0 | 0.03251307 | 0 | 0 | 0.002255785 |
| GSM3290110_treat | 0.051085582 | 0 | 0.033753969 | 0.029491562 | 0 | 0.091042916 | 0.003985938 | 0 | 0 | 0 | 0.046815647 | 0 | 0.057171084 | 0.11666162 | 0 | 0.169362796 | 0 | 0.19162402 | 0.14830619 | 0 | 0 | 0.060698676 |
| GSM3290111_treat | 0.082829027 | 0 | 0.027136783 | 0.060607758 | 0 | 0.19683451 | 0 | 0.006356251 | 0 | 0 | 0.006090246 | 0.03619257 | 0.051886418 | 0.145575326 | 0.00848395 | 0.114960455 | 0 | 0.04824573 | 0.156904724 | 0 | 0.001415887 | 0.056480365 |
| GSM3290112_treat | 0.065598327 | 0 | 0.068757086 | 0.038712951 | 0 | 0.199316865 | 0.004400807 | 0 | 0 | 0.025154118 | 0.012744634 | 0.00119587 | 0.016116443 | 0.124636585 | 0 | 0.199007407 | 0 | 0.065852269 | 0.130540151 | 0 | 0.015189394 | 0.032777094 |
| GSM3290113_treat | 0.042005002 | 0.089341861 | 0.138708307 | 0.158370329 | 0 | 0.138069619 | 0.029388052 | 0 | 0 | 0.006663808 | 0.017564808 | 0 | 0.000808138 | 0.122298891 | 0.064050558 | 0.121031711 | 0 | 0.023293532 | 0.041188745 | 0 | 0 | 0.007216637 |
| GSM3290114_treat | 0.061713569 | 0 | 0.066505242 | 0.07746957 | 0 | 0.160676653 | 0 | 0 | 0 | 0 | 0 | 0.009808559 | 0.022914698 | 0.16543071 | 0.008309581 | 0.298559478 | 0 | 0.024880939 | 0.070672011 | 0 | 0.009929955 | 0.023129034 |
| GSM3290115_treat | 0.032537031 | 0 | 0.037912102 | 0.045843554 | 0 | 0.26693231 | 0 | 0 | 0 | 0 | 0.040568285 | 0.008653657 | 0.067672112 | 0.062599053 | 0 | 0.145185926 | 0 | 0.065076811 | 0.203071858 | 0 | 0 | 0.0239473 |
| GSM3290116_treat | 0.102110375 | 0 | 0.062588729 | 0.074961826 | 0 | 0.207916804 | 0 | 0.034723832 | 0 | 0 | 0.002802015 | 0 | 0.035836535 | 0.061499503 | 0.02984949 | 0.139164243 | 0 | 0.060027122 | 0.145109527 | 0 | 0.022493459 | 0.020916539 |
| GSM3290117_treat | 0.074606951 | 0 | 0.022430118 | 0.061707602 | 0 | 0.246082991 | 0 | 0 | 0 | 0 | 0.028239111 | 0 | 0.101430545 | 0.008972396 | 0 | 0.193132928 | 0 | 0.03945135 | 0.186187122 | 0 | 0 | 0.037758886 |
| GSM3290120_treat | 0.041378365 | 0 | 0.048710652 | 0.010917339 | 0 | 0.180154068 | 0 | 0 | 0 | 0.005181553 | 0.024885819 | 0 | 0.042864835 | 0.087965508 | 0.021010049 | 0.174659995 | 0 | 0.166640396 | 0.158505497 | 0 | 0.024645385 | 0.01248054 |
| GSM3290121_treat | 0.09321763 | 0 | 0.04204363 | 0.027296079 | 0 | 0.196409841 | 0.006401955 | 0 | 0 | 0 | 0.053549719 | 0 | 0.033923358 | 0.059741575 | 0.023478283 | 0.136368521 | 0 | 0.127779627 | 0.072282986 | 0 | 0 | 0.127506797 |
| GSM3290126_treat | 0.123526147 | 0 | 0.088210437 | 0.019225958 | 0 | 0.233504317 | 0.004076774 | 0 | 0 | 0 | 0.030889358 | 0 | 0.017339037 | 0.054335219 | 0.065078554 | 0.133485411 | 0 | 0.047210067 | 0.102736087 | 0 | 0.005943926 | 0.074438709 |
| GSM3290127_treat | 0.081186805 | 0 | 0.058510166 | 0.028253149 | 0 | 0.135797981 | 0.005560782 | 0 | 0 | 0 | 0.047528986 | 0 | 0.058073134 | 0.016482068 | 0 | 0.115820718 | 0 | 0.245954121 | 0.134941241 | 0 | 0 | 0.071890849 |
| GSM3290128_treat | 0.078241492 | 0.012860216 | 0.091507315 | 0.037834804 | 0 | 0.12330407 | 0.006254953 | 0 | 0 | 0 | 0 | 0.019092927 | 0.012136058 | 0.003706428 | 0.005960851 | 0.146843405 | 0 | 0.103938306 | 0.329939802 | 0 | 0.028379373 | 0 |
| GSM3290129_treat | 0.074489897 | 0 | 0.057462562 | 0.046053407 | 0 | 0.151791792 | 0 | 0 | 0 | 0 | 0.032119569 | 0 | 0.028949174 | 0.088861879 | 0.009706162 | 0.190958678 | 0 | 0.085171906 | 0.185598226 | 0 | 0.009486182 | 0.039350566 |
| GSM3290130_treat | 0.114802844 | 0 | 0.070480226 | 0.037846305 | 0 | 0.16078559 | 0.006917576 | 0 | 0 | 0 | 0.026813766 | 0 | 0.028607915 | 0.023899619 | 0 | 0.149298999 | 0 | 0.148621117 | 0.174680067 | 0 | 0 | 0.057245975 |
| GSM3290135_treat | 0.161203514 | 0 | 0.031489902 | 0.026404067 | 0 | 0.252531277 | 0 | 0.007335881 | 0 | 0 | 0 | 0.007369368 | 0.04585554 | 0.00135375 | 0 | 0.157723783 | 0 | 0.062109158 | 0.114409363 | 0 | 0.030203448 | 0.102010947 |
| GSM3290136_treat | 0.067212612 | 0 | 0.030087364 | 0.061444511 | 0 | 0.258228277 | 0 | 0 | 0 | 0 | 0.043628644 | 0 | 0.05326326 | 0.016564605 | 0 | 0.148535616 | 0 | 0.060630109 | 0.127367383 | 0 | 0.075092267 | 0.057945352 |
| GSM3290137_treat | 0.092593214 | 0 | 0.071365052 | 0.057208075 | 0 | 0.179724486 | 0.005274223 | 0 | 0 | 0 | 0.001014938 | 0.009925798 | 0.043852618 | 0.091736476 | 0 | 0.17406265 | 0 | 0.01875586 | 0.209714214 | 0 | 0 | 0.044772396 |
| GSM3290138_treat | 0.130778563 | 0 | 0.032682341 | 0.030524244 | 0 | 0.246437515 | 0 | 0 | 0 | 0 | 0.022808276 | 0.02366372 | 0.041778478 | 0 | 0.060701026 | 0.208047013 | 0 | 0.035009676 | 0.108116892 | 0 | 0 | 0.059452254 |
| GSM3290139_treat | 0.036768773 | 0 | 0.079857406 | 0.067159946 | 0 | 0.275877176 | 0 | 0 | 0 | 0 | 0.000671347 | 0.004003801 | 0.007448108 | 0.133714374 | 0.016070163 | 0.094503569 | 0 | 0.117733586 | 0.124911503 | 0 | 0 | 0.041280249 |
| GSM3290140_treat | 0.065129683 | 0 | 0.088531848 | 0.0385735 | 0 | 0.17798904 | 0.008688003 | 0 | 0 | 0 | 0.008688207 | 0 | 0.012916245 | 0.111862209 | 0.023676709 | 0.214921281 | 0 | 0.025273753 | 0.207311313 | 0 | 0.007749179 | 0.008689029 |
| GSM3290141_treat | 0.063295589 | 0 | 0.098412129 | 0.01344111 | 0 | 0.219682686 | 0 | 0 | 0 | 0 | 0.003302484 | 0 | 0.009516141 | 0.046035992 | 0.044312935 | 0.181606949 | 0 | 0.059289209 | 0.210774225 | 0 | 0.02172039 | 0.028610163 |
| GSM3290142_treat | 0.103028315 | 0 | 0.093425736 | 0.090813234 | 0 | 0.109777259 | 0 | 0 | 0 | 0 | 0.045782882 | 0 | 0.090642901 | 0.054514 | 0.002091484 | 0.131364716 | 0 | 0.101907226 | 0.093526918 | 0 | 0.003678297 | 0.079447032 |
| GSM3290143_treat | 0.068829972 | 0 | 0.041265004 | 0.027557106 | 0 | 0.149588399 | 0 | 0 | 0 | 0 | 0.026276942 | 0 | 0.01576023 | 0.093853842 | 0.024303087 | 0.282998624 | 0 | 0.126380051 | 0.099013679 | 0 | 0.010110698 | 0.034062368 |
| GSM3290144_treat | 0.073466246 | 0 | 0.082815172 | 0.036289766 | 0 | 0.167576086 | 0.021915677 | 0 | 0 | 0 | 0.015682511 | 0 | 0.014889461 | 0.181148729 | 0.013327924 | 0.209833317 | 0 | 0.012705722 | 0.166390627 | 0 | 0 | 0.003958764 |
| GSM3290145_treat | 0.037195362 | 0 | 0.046983759 | 0.017814048 | 0 | 0.166727782 | 0 | 0 | 0 | 0 | 0.036613953 | 0 | 0.108492485 | 0.112917566 | 0.010735546 | 0.203463153 | 0 | 0.072083398 | 0.076409017 | 0 | 0 | 0.110563931 |
| GSM3290148_treat | 0.084064751 | 0 | 0.104937854 | 0.040437395 | 0 | 0.135456205 | 0.013077819 | 0 | 0 | 0 | 0.059721757 | 0 | 0.033259977 | 0.148390155 | 0.000915069 | 0.251749859 | 0 | 0.024700567 | 0.07711347 | 0 | 0.013811146 | 0.012363976 |
| GSM3290149_treat | 0.177382112 | 0 | 0.030108048 | 0.075380238 | 0 | 0.17640514 | 0 | 0.024655536 | 0 | 0 | 0.050749195 | 0 | 0.033713938 | 0.058750021 | 0.075888997 | 0.129276388 | 0 | 0.076689583 | 0.071702337 | 0 | 0 | 0.019298469 |
